# ALA‐A2 Is a Novel Anticancer Peptide Inspired by Alpha‐Lactalbumin: A Discovery from a Computational Peptide Library, In Silico Anticancer Peptide Screening and In Vitro Experimental Validation

**DOI:** 10.1002/gch2.202200213

**Published:** 2023-01-12

**Authors:** Tassanee Lerksuthirat, Pasinee On‐yam, Sermsiri Chitphuk, Wasana Stitchantrakul, David S. Newburg, Ardythe L. Morrow, Suradej Hongeng, Wararat Chiangjong, Somchai Chutipongtanate

**Affiliations:** ^1^ Research Center Faculty of Medicine Ramathibodi Hospital Mahidol University Bangkok 10400 Thailand; ^2^ Pediatric Translational Research Unit Department of Pediatrics Faculty of Medicine Ramathibodi Hospital Mahidol University Bangkok 10400 Thailand; ^3^ Faculty of Medicine Ramathibodi Hospital Mahidol University Bangkok 10400 Thailand; ^4^ Division of Epidemiology Department of Environmental and Public Health Sciences University of Cincinnati College of Medicine Cincinnati OH 45267 USA; ^5^ Division of Infectious Diseases Department of Pediatrics Cincinnati Children's Hospital Medical Center University of Cincinnati College of Medicine Cincinnati OH 45267 USA; ^6^ Division of Hematology and Oncology Department of Pediatrics Faculty of Medicine Ramathibodi Hospital Mahidol University Bangkok 10400 Thailand

**Keywords:** anticancer peptides, cytotoxic screening, drug discovery, lung adenocarcinoma, machine learning, peptide library, SWATH‐MS

## Abstract

Anticancer peptides (ACPs) are rising as a new strategy for cancer therapy. However, traditional laboratory screening to find and identify novel ACPs from hundreds to thousands of peptides is costly and time consuming. Here, a sequential procedure is applied to identify candidate ACPs from a computer‐generated peptide library inspired by alpha‐lactalbumin, a milk protein with known anticancer properties. A total of 2688 distinct peptides, 5–25 amino acids in length, are generated from alpha‐lactalbumin. In silico ACP screening using the physicochemical and structural filters and three machine learning models lead to the top candidate peptides ALA‐A1 and ALA‐A2. In vitro screening against five human cancer cell lines supports ALA‐A2 as the positive hit. ALA‐A2 selectively kills A549 lung cancer cells in a dose‐dependent manner, with no hemolytic side effects, and acts as a cell penetrating peptide without membranolytic effects. Sequential window acquisition of all theorical fragment ions‐proteomics and functional validation reveal that ALA‐A2 induces autophagy to mediate lung cancer cell death. This approach to identify ALA‐A2 is time and cost‐effective. Further investigations are warranted to elucidate the exact intracellular targets of ALA‐A2. Moreover, these findings support the use of larger computational peptide libraries built upon multiple proteins to further advance ACP research and development.

## Graphical abstract

This study describes a novel strategy for searching for a new anticancer peptide (ACP) by integrating the computational peptide library of alpha‐lactalbumin with downstream in silico machine learning‐based screening and in vitro experimental validation. The novel ACP, so‐called ALA‐A2, specifically kills lung adenocarcinoma cells through autophagy mediated cell death.

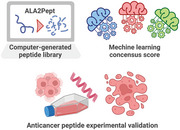

## 1 Introduction

Worldwide, cancer is a leading cause of premature death and a growing public health burden. Global cancer estimates indicate up to 19 million new cases and 10 million cancer deaths in 2020.^[^ ^1^ ^]^ Conventional cancer therapies, such as chemotherapy and radiation, frequently result in cancer resistance and a lack of tumor selectivity. More effective treatment agents are required to improve patient outcomes.

Anticancer peptides (ACP) are sequences of amino acids of fewer than 25 amino acids in length that have a cytotoxic effect on cancer cells.^[^ ^2^ ^]^ The modes of action of ACPs can be divided into three categories: 1) membranolytic peptides that kill cancer cells through pore formation on the cellular membrane; 2) cell penetrating peptides that are rich in basic amino acids (arginine and lysine), leading to translocation and access to intracellular compartments to disturb cellular homeostasis; and 3) tumor‐targeting peptides that directly interact with cancer‐specific molecules to mediate cell death.^[^ ^2^ ^]^ Two major approaches of ACP discovery include activity‐guided purification from biological or natural sources of interest,^[^ ^3^ ^]^ and experimental screening of an established peptide library, that is, antimicrobial peptides.^[^ ^4^ ^]^

In our hands, a new strategy of ACP screening based on bio–physico–chemical features and machine learning (ML) preference of the input peptides proved to be time‐ and cost‐effective for prioritizing candidates for experimental studies.^[^ ^5^ ^]^ A naturally occurring peptide library from human milk, a promising source for therapeutic peptide discovery,^[^ ^5^, ^6^ ^]^ was generated by sequential peptide fractionation coupled with liquid chromatography‐tandem mass spectrometry. Of 142 input peptides, in silico ACP screening with subsequent experimental validation identified an anti‐leukemic peptide that selectively kills four distinct leukemic cell lines in vitro and three patient‐derived leukemic cells ex vivo.^[^ ^5^ ^]^ However, the bottleneck of the overall workflow lies in the relatively small and limited peptide library. Unfortunately, expansion of the peptide library using traditional approaches is associated with significant time and budget constraints. To address this issue, we had proposed a large peptide library generated by a computational method to facilitate further discovery of ACP from human milk.^[^ ^5^ ^]^

Alpha‐lactalbumin, a 16‐kDa milk protein with 142 amino acids, when in a complex with oleic acid, selectively induces cancer cell death in vitro and in vivo. Named “human alpha‐lactalbumin made lethal to tumor cells (HAMLET)”, this complex is currently under investigation in an early phase clinical trial.^[^ ^7^ ^]^ The monomeric form of alpha‐lactalbumin was reported to be inactive for anticancer activity,^[^ ^8^ ^]^ but alpha‐lactalbumin could nevertheless provide a good lead for developing a computer‐generated peptide library, followed by deployment of in silico ACP screening and experimental validation of ACP candidates.

This study aimed to establish a workflow for discovery of novel ACPs from potential alpha‐lactalbumin peptides. An R‐based script was created to generate all possible peptides that could be derived from the amino acid sequence of alpha‐lactalbumin. The ACP physicochemical and structural properties, as well as the ACP probabilities predicted by three different ML models, were used to prioritize the peptide candidates from the 2688‐peptide library. Anticancer activities of the candidate peptides were experimentally measured in vitro against human cancer cell lines derived from lung, breast, colon, brain, and leukemia cancers. Their hemolytic side effects were tested using a red blood cell (RBC) lysis assay. Sequential window acquisition of all theorical fragment ions (SWATH)‐proteomics was performed to determine a potential mechanism of action of the peptide candidate, followed by functional validation. This workflow resulted in discovery of a novel ACP that induces autophagy‐associated cell death in a human lung cancer cell line.

## 2 Results

**Figure 1:**
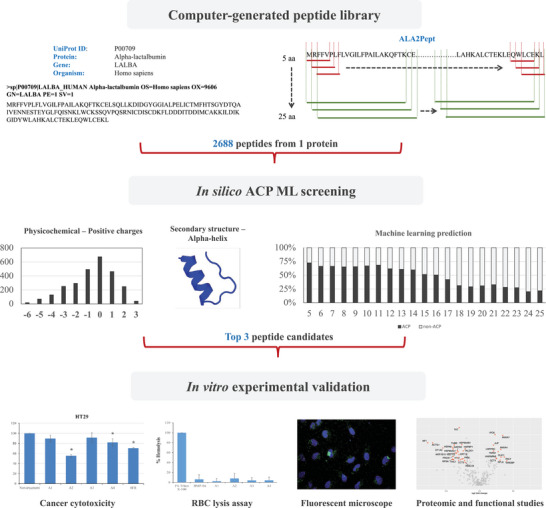
The entire workflow of ACP discovery from a computer‐generated peptide library based on alpha‐lactalbumin, in vivo ACP screening, and in vitro validation experiments. aa: amino acid; ACP: anticancer peptide.

This study investigated the utility of computer‐based peptide library generation coupled with in silico ACP screening^[^ ^5^ ^]^ for prioritizing peptide candidates. The top predicted ACP candidates were tested in vitro for actual anticancer activity against various cancer cell lines and also evaluated for potential toxicity using a RBC lysis assay ex vivo. The overall workflow of this study is illustrated in **Figure** **1**.

### 2.1 Alpha‐Lactalbumin Inspired Peptide Library Generation and In Silico ACP Screening

**Figure 2:**
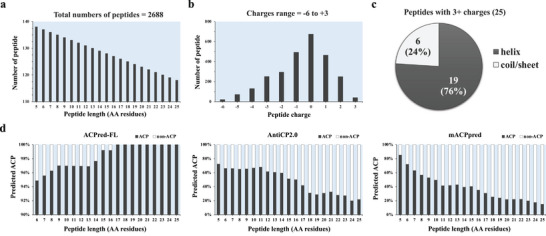
The distributions of various characteristics of the 2688 peptides generated from alpha‐lactalbumin. a) Numbers of peptides by length (5–25 amino acid residues). b) Numbers of peptides by the physicochemical charge property. c) Secondary structure of 25 peptides containing +3 net charges predicted by three ML models. d) ACP probabilities as predicted by three distinct ML models, that is, ACPred‐FL, Anticp2.0, and mACPpred.

Human alpha‐lactalbumin (UniProt ID: P00709) is comprised of 142 amino acids. Here, 2688 unique peptides, ranging from 5 to 25 amino acids per peptide with stepping 1 amino acid each were generated from alpha‐lactalbumin by using the peptide generator ALA2Pept. The distributions of the 2688 peptides based on physicochemical, structural, and ML probabilities are shown in **Figure** **2**. As expected, the numbers of computer‐generated peptides were inverse to the peptide length (Figure 2a). The net charge of peptides ranged from −6 to +3, with the electrically neutral peptides being the majority of this library (Figure 2b). Most known ACPs have a positive charge,^[^ ^2^, ^5^ ^]^ so the initial focus was on the peptides with +3 charge. A secondary structure prediction was performed on this subset of peptides with a +3 net charge. Of the 25 peptides (≈1% of the total 2688 peptides) with +3 net charge, 19 were predicted to have α‐helical conformation (ACP‐preferred structure) and 6 had a coil/sheet structure (Figure 2c). Three distinct ML models (ACPred‐FL, Anticp2.0, and mACPpred) revealed distinct, but complementary, distributions of the predicted ACPs (Figure 2d). The ACP candidates were ranked by the consensus score (geometric mean) of the three ACP probabilities, and filtered against their physicochemical and structural properties. All peptide sequences and the results of in silico ACP screening of 2688 peptides are provided in Table S1, Supporting Information.

### 2.2 Cancer Cytotoxic Screening Indicates ALA‐A2 as the Positive Hit

**Figure 3:**
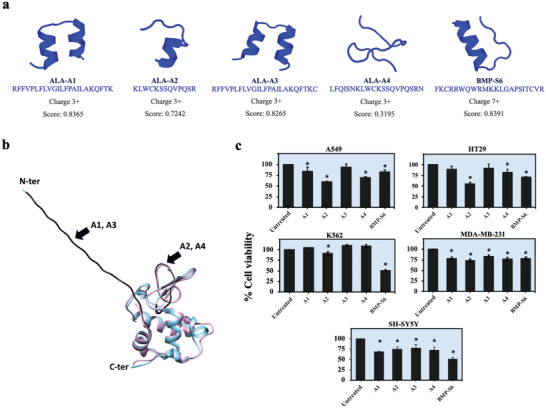
In vitro cytotoxicity of the synthetic peptides against five human cancer cell lines. a) The amino acid sequences with the predicted secondary structures, net charges, and consensus scores of all synthetic peptides included for experimental validations. b) The positions of ALA‐A1, ‐A2, ‐A3, and ‐A4 peptides (black) on the predicted structure (blue) and the crystal structure (pink; PDB:1b9o) of alpha‐lactalbumin as visualized by UCSF chimera 1.15.^[^ ^11^ ^]^ Note that ALA‐A3 and ALA‐A4 have amino acid sequences that partially overlap with ALA‐A1 and ALA‐A2, respectively. c) Cancer cytotoxicity screening against five human cancer cell lines, including lung cancer (A549), colon cancer (HT29), breast cancer (MDA‐MB‐231), neuroblastoma (SH‐SY5Y), and leukemia (K562) cells. The synthetic peptides (200 µm) were incubated with cancer cells at 37 °C for 24 h before measuring cell viability (MTT assay). Only ALA‐A2 exhibited a selective and substantial anticancer effect against A549 and HT29 cells. This experiment was performed in three biological replicates. *p < 0.05; N‐ter: N‐terminal; C‐ter: C‐terminal.

The list of candidates selected for the peptide synthesis and experimental validations is shown in **Table** **1** and **Figure** **3**a. The top two peptide candidates, ALA‐A1 and ALA‐A2, are indicated by the highest consensus score peptides with non‐redundant sequence, having ≥3 positive charges, and containing alpha‐helical secondary structure. Based on the similarity of their amino acid sequences, ALA‐A3 and ALA‐A4 are included as the lower‐ranked peptide comparators of ALA‐A1 and ALA‐A2, respectively. Figure 3b illustrates the positions of these peptides within the 3D structure of alpha‐lactalbumin (PDB: 1b9o) generated by AlphaFold.^[^ ^9^ ^]^ ALA‐A1 and ALA‐A3 peptides are derived from the linear N‐terminal chain, while ALA‐A2 and ALA‐A4 are presented as part of the external surface of the folded alpha‐lactalbumin.

**Table 1:** Physicochemical, structural, and machine learning properties of selected peptide ACP candidates

ID	Amino acid sequence	Length	Charge	Structure	Machine learning model			Consensus score	Prediction
					ACPred‐FL	AntiCp 2.0	mACP pred		
ALA‐A1	RFFVPLFLVGILFPAILAKQFTK	23	3+	coil–helix–coil–helix	0.980	0.620	0.964	0.836	ACP
ALA‐A2	KLWCKSSQVPQSR	13	3+	helix–coil	0.992	0.480	0.798	0.724	ACP
ALA‐A3	RFFVPLFLVGILFPAILAKQFTKC	24	3+	helix–coil–helix	0.980	0.590	0.977	0.826	ACP
ALA‐A4	LFQISNKLWCKSSQVPQSRN	20	3+	coil	0.944	0.460	0.075	0.319	non‐ACP
BMP‐S6 (positive control)^[^ ^5^, ^10^ ^]^	FKCRRWQWRMKKLGAPSITCVR	22	7+	coil–helix	0.606	1.000	0.9848	0.842	ACP

To determine whether the in silico predicted candidates actually exhibit anticancer effects in vitro, the synthetic peptides were tested against five cancer cell lines (Figure 3c). BMP‐S6, a bovine‐derived ACP from previous studies,^[^ ^5^, ^10^ ^]^ was included as the positive control of this study. ALA‐A2 (at 200 µm) selectively induced cancer cytotoxicity of A549 human lung epithelial adenocarcinoma (59.7 ± 1.1% cell viability) and HT29 human colorectal adenocarcinoma (55.3 ± 2.7% cell viability) cell lines, but did not affect K562 human lymphoblastic leukemia, MDA‐MB‐231 human breast cancer, and SH‐SY5Y human neuroblastoma cell lines. ALA‐A1, ALA‐A3, and ALA‐A4 did not exhibit a substantial anticancer effect against the five cell lines examined. BMP‐S6 was the only peptide that exhibited cancer cytotoxicity against K562 leukemic cells, consistent with previous studies.^[^ ^5^, ^10^ ^]^ Accordingly, ALA‐A2 was designated the positive hit from in silico and in vitro ACP screening, and subjected to further validation.

### 2.3 Dose–Response Relationship, Hemolytic Side Effect, and Cell Internalization of ALA‐A2

**Figure 4:**
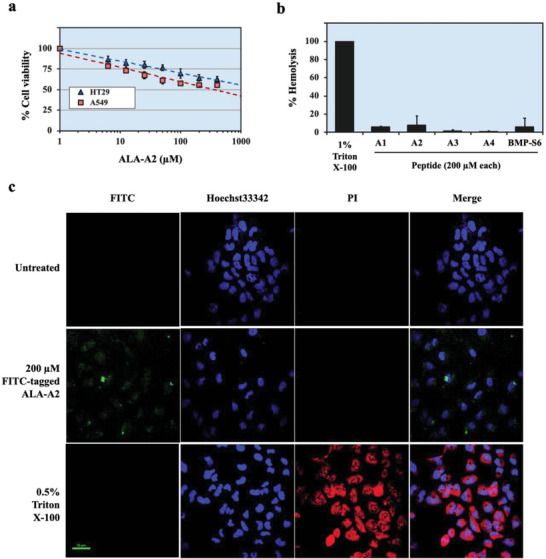
Dose‐dependent cytotoxicity, hemolytic side effect, and cell penetrating capability of ALA‐A2 peptide. a) ALA‐A2 dose–response relationship in A549 lung cancer and HT29 colon cancer cell lines. The cells were incubated with twofold dilutions of ALA‐A2 from 400 to 0 µm for 24 h, and then cell viability was measured by the MTT assay. b) RBC lysis assay for ALA‐A2 peptide toxicity testing. Human red blood cells were incubated with the peptides for 1 h. Triton X‐100 at a final concentration of 1% was used as a positive control. c) Confocal microscopy showed that FITC‐tagged ALA‐A2 peptide (green) could internalize into A549 lung cancer cells without altered membrane integrity, whereas Triton X‐100 (0.5% v/v; the positive control) could permeabilize cellular membrane allowing PI internalization (red). Hoechst 33342 (blue) was used for the nuclear counter staining. Scale bar: 50 µm.

The dose dependency of ALA‐A2 against A549 and HT29 cells was measured at twofold serial dilutions from 0 to 400 µm. Based on this dose testing range, the IC50 of A549 is ≈300 µm and the IC50 of HT29 is extrapolated to be greater than 1000 µm. The higher concentration of ALA‐A2 doses resulted in lower cell viability of A549, and, to a lesser extent, of HT29 cells (**Figure** **4**a).

Hemolysis is a major concern during the development of peptide‐based drugs; hemolytic peptides do not progress to clinical trials.^[^ ^12^ ^]^ Thus, hemolytic activity of the ACP ALA‐A2 was compared with those of other peptides without anticancer activity. Figure 4b illustrates that neither ALA‐A2 (200 µm) nor the other peptides exhibit hemolytic effects against normal RBCs after 1 h incubation. The percent hemolysis was ALA‐A1, 5.6% ± 0.6%; ALA‐A2, 7.8% ± 10.2%; ALA‐A3, 1.3% ± 1.2%; ALA‐A4, 0.7% ± 0.6%; and BMP‐S6, 6.0% ± 9.2%. Note that 1% Triton X‐100, the positive control, completely lysed the normal RBCs within 1 h.

The anticancer mechanism of ALA‐A2 was investigated in A549 lung cancer cells. Membranolysis and cell penetrating peptides are both common, but distinct, modes of ACP action.^[^ ^2^ ^]^ To explore the mode of action of ALA‐A2, A549 lung cancer cells were treated with FITC‐tagged ALA‐A2 peptide (200 µm) for 24 h, followed by confocal microscopy. Figure 4c illustrates that the signals of FITC‐tagged ALA‐A2 were present in the intracellular compartments of A549 cells. ALA‐A2 did not disrupt membrane integrity. Propidium iodide (PI) is internalized following treatment with Triton X‐100, the positive control for a cell permeabilized agent,^[^ ^13^ ^]^ whereas it is not internalized following treatment with ALA‐A2. Thus, ALA‐A2 penetrates the cell and may be the trigger for cell death‐related signaling of A549 lung cancer cells.

### 2.4 ALA‐A2 Triggered Autophagy‐Mediated Cell Death in A549 Lung Adenocarcinoma Cells

**Figure 5:**
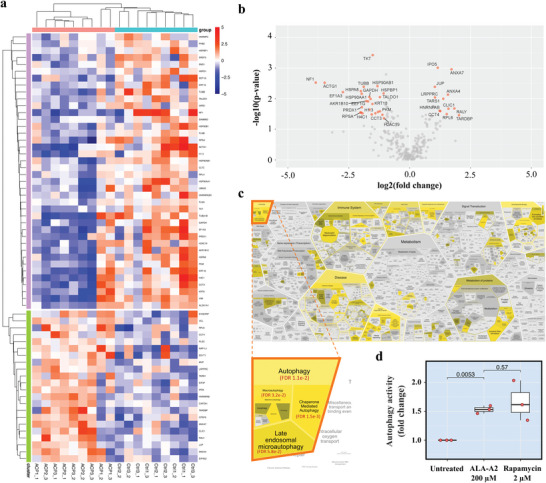
SWATH‐proteomic and functional analyses suggest that cell death is caused by autophagic induction. a) Heatmap and b) volcano plot identified the significantly differentially expressed proteins between ALA‐A2 peptide‐treated versus untreated A549 cells (*n* = 9 per group; three technical replicates of three biological specimens) (full expression datasets in Table S2, Supporting Information). c) This reactome was subjected to biological pathway analyses. The stronger yellow accent indicates lower false‐discovery rate. Chaperone mediated autophagy was predicted as the most significant pathway elicited in ALA‐A2 peptide‐treated A549 cells (details in Table S3, Supporting Information). d) Autophagy activity assay. Rapamycin, an autophagic inducer, was the positive control. This experiment was performed in three biological replicates. *p < 0.05.

SWATH‐proteomics was performed to investigate potential mechanisms of the ALA‐A2 anticancer effect in A549 lung cancer cells. Of 286 proteins detected and quantified across 18 data independent acquisition (DIA) runs (full data sets in Table S2, Supporting Information), heatmap and volcano plot revealed 38 differentially expressed proteins (12 upregulated and 26 downregulated) in ALA‐A2 treated A549 cells (as illustrated in **Figure** **5**a,b and **Table** **2**). Reactome pathway enrichment analysis was performed to deduce biological meaning from this differential protein signature. Figure 5c demonstrates that the autophagy pathways, particularly chaperone‐mediated autophagy, were the best matched pathway with the lowest false discovery rate (details in Table S3, Supporting Information). This functional match mostly reflected four downregulated proteins: ubiquitin‐60S ribosomal protein L40 (fold‐change = 0.41, p = 0.027), heat shock 70 kDa protein 8 (fold‐change = 0.25, *p* = 0.006), and heat shock protein HSP90 alpha (fold‐change = 0.26, *p* = 0.007) and beta (fold‐change = 0.40, *p* = 0.004) (Figure 5a,b and Table 2 and Table S3, Supporting Information). To confirm this deduction, the ability of ALA‐A2 peptide (200 µm) to induce autophagy was tested. As shown in Figure 5d, ALA‐A2 treatment significantly increased the autophagic activity in A549 cells (1.5‐fold of untreated cells) comparable to that of rapamycin, a positive control known to induce autophagy. Taken together, these findings identified ALA‐A2 as a cell penetrating peptide that induces autophagy‐mediated cell death in A549 human lung adenocarcinoma cells.

**Table 2:** Significantly differentially expressed proteins between ALA‐A2 treated versus untreated A549 lung adenocarcinoma cells

SwisProt ID	Gene name	Protein name	%Coverage	Number of matched peptides	MW [kDa]	*p*I	Fold‐change [treated/ untreated]	*p*‐value
Proteins whose expression was upregulated in ALA‐A2 treated cells								
Q13148	*TADBP*	TAR DNA‐binding protein 43	2.90	1	44.74	5.85	4.07	0.0348
Q9UKM9	*RALY*	RNA‐binding protein Raly (Autoantigen p542)	6.86	1	32.463	9.20	3.57	0.0209
P20073	*ANXA7*	Annexin A7	8.61	1	52.739	5.52	3.29	0.0011
O00299	*CLIC1*	Chloride intracellular channel protein 1	11.20	2	26.923	5.09	3.02	0.0212
P09525	*ANXA4*	Annexin A4	18.81	1	35.883	5.84	2.98	0.0074
Q02878	*RL6*	60S ribosomal protein L6	19.10	2	32.728	10.59	2.87	0.0319
P26639	*SYTC*	Threonine‐tRNA ligase 1	4.70	1	83.435	6.23	2.61	0.0100
P50991	*TCPD*	T‐complex protein 1 subunit delta	13.36	3	57.924	8.13	2.39	0.0255
Q99729	*ROAA*	Heterogeneous nuclear ribonucleoprotein A/B	5.42	1	36.225	8.21	2.35	0.0248
O00410	*IPO5*	Importin‐5	6.38	3	123.63	4.83	2.22	0.0010
P42704	*LPPRC*	Leucine‐rich PPR motif‐containing protein	14.92	11	157.905	5.53	2.06	0.0091
P14923	*PLAK*	Junction plakoglobin	7.52	2	81.745	5.75	2.05	0.0041
Proteins whose expression was downregulated in ALA‐A2 treated cells								
Q6FI13	*H2A2A*	Histone H2A type 2‐A	62.31	19	14.095	10.90	0.48	0.0438
Q9NZL4	*HPBP1*	Hsp70‐binding protein 1	8.36	2	39.303	5.13	0.48	0.0067
P49368	*TCPG*	T‐complex protein 1 subunit gamma	10.83	3	60.534	6.10	0.46	0.0336
P14618	*KPYM*	Pyruvate kinase PKM	54.43	32	57.937	7.95	0.43	0.0258
P37837	*TALDO*	Transaldolase	17.80	3	37.54	6.36	0.43	0.0089
P62987	*UBA52*	Ubiquitin‐60S ribosomal protein L40	52.34	4	14.728	6.56	0.41	0.0272
P08238	*HSP90AB1*	Heat shock protein HSP90 beta	37.15	25	83.264	4.96	0.40	0.0039
P14625	*ENPL*	Endoplasmin	17.68	10	92.469	4.73	0.38	0.0301
P29401	*TKT*	Transketolase	39.33	20	67.878	7.58	0.35	0.0004
P05783	*K1C18*	Keratin, type I cytoskeletal 18	53.02	42	48.058	5.34	0.35	0.0149
P08727	*K1C19*	Keratin, type I cytoskeletal 19	23.75	7	44.106	5.05	0.34	0.0327
P84103	*SRSF3*	Serine/arginine‐rich splicing factor 3	12.80	1	19.33	11.64	0.33	0.0102
P16402	*H13*	Histone H1.3	25.34	2	22.35	11.02	0.32	0.0227
P04406	*G3P*	Glyceraldehyde‐3‐phosphate dehydrogenase	60.00	32	36.053	8.58	0.32	0.0088
P26641	*EF1G*	Elongation factor 1‐gamma	20.14	4	50.119	6.27	0.31	0.0123
O60218	*AK1BA*	Aldo‐keto reductase family 1 member B10	71.52	26	36.02	7.66	0.29	0.0120
P07437	*TBB5*	Tubulin beta chain	53.15	39	49.671	4.78	0.27	0.0041
P62805	*H4*	Histone H4	57.28	15	11.367	11.36	0.26	0.0302
Q06830	*PRDX1*	Peroxiredoxin‐1	29.65	10	22.11	8.27	0.26	0.0190
P07900	*HSP90AA1*	Heat shock protein HSP90 alpha	37.02	24	84.66	4.94	0.26	0.0069
P22626	*ROA2*	Heterogeneous nuclear ribonucleoproteins A2/B1	24.93	3	37.43	8.97	0.25	0.0281
P11142	*HSPA8*	Heat shock 70 kDa protein 8	28.79	21	70.898	5.37	0.25	0.0056
P08865	*RSSA*	40S ribosomal protein SA	17.97	3	32.854	4.79	0.25	0.0285
Q5VTE0	*EF1A3*	Elongation factor 1‐alpha‐like 3	35.93	19	50.185	9.15	0.15	0.0061
P63261	*ACTG*	Actin, cytoplasmic 2	63.20	53	41.793	5.31	0.09	0.0030
P21359	*NF1*	Neurofibromin	1.02	1	319.372	7.12	0.07	0.0030

## 3 Discussion

The novel approach to ACP identification described in this study demonstrates that a computer‐generated peptide library with downstream in silico ACP screening and in vitro experimental validation could accelerate anticancer research and development. Alpha‐lactalbumin, a milk protein known for anticancer properties,^[^ ^7^, ^14^ ^]^ inspired this study design. The library of 2688 distinct peptides containing 5–25 amino acids in length were generated from the sequence information of alpha‐lactalbumin rapidly (Figure 2). As different ML models were established upon various data sets, algorithms, and tuning parameters, their predictive performance can vary.^[^ ^15^ ^]^ Rather than relying on only one ML model, we utilized the consensus score, which is a geometric mean of ACP probabilities of three different ML models, plus the physicochemical and structural properties, to obtain better ACP candidates. After in silico ACP screening of 2688 peptides, a few top‐ranked candidates were achieved within days for the custom‐ordered peptide synthesis and in vitro experimental validation (Figures 3, 4, 5). This study identified ALA‐A2 as a novel ACP in a time‐ and cost‐effective manner. ALA‐A2 was relatively selective to lung adenocarcinoma cells, acting as a cell penetrating peptide to induce autophagy‐mediated cell death without provoking hemolysis. The sequence of ALA‐A2 as KLWCKSSQVPQSR was predicted for its half‐life in blood using PlifePred (https://webs.iiitd.edu.in/raghava/plifepred/, accessed on October 30, 2022) that remained ≈20 min in blood.^[^ ^16^ ^]^ Because of a relatively short half‐life of ALA‐A2 peptide, it should be modified, for example, by amidation at N‐ or C‐terminus,^[^ ^4^, ^17^ ^]^ to improve the stability of ALA‐A2 for further clinical applications. Moreover, the 200 µm ALA‐A2 concentration was chosen to observe lung cancer cell responses because the effect of this dose was obvious on A549 cells. This high dose has no hemolytic effect (Figure 4b) while exhibiting lung cancer cytotoxicity through autophagy‐induced cell death (Figure 5).

Brisuda et al.^[^ ^7a^ ^]^ reported that the α‐helical peptide (residues 1–39) derived from alpha‐lactalbumin in complexes with oleate (so‐called alpha1‐oleate) had a tumoricidal effect against bladder cancer, but beta‐sheet‐oleate (residue at 40–80) complexes lacked anticancer properties. Both ALA‐A1 and ALA‐A3 partially overlapped with the alpha1‐oleate, while ALA‐A2 and ALA‐A4 were part of the beta‐sheet‐oleate. However, the data reported herein indicate that only ALA‐A2 exhibits the anticancer effect. This discrepancy may be due to the absence of oleate, the shorter length of ALA‐peptides, and the different cancer cell lines examined in this study. Our computational workflow did not generate any peptide longer than 25 amino acid residues. By default, the bare peptides of alpha1‐oleate (39 amino acids in length) and beta‐sheet‐oleate (41 amino acids in length) were not evaluated in this study. The 5 amino acid peptide length is the minimal number that mass spectrometry can detect and is the shortest peptide length for ACP prediction available (mACPred [≥5 amino acids], Anti‐CP2.0 [4–50 amino acids], and ACPred‐FL [10–50 amino acids]). However, the shortest peptide in the protein data bank is seven amino acids long as this size is needed to form the crystalline structure of the alpha‐helical peptide.^[^ ^18^ ^]^ A peptide length of 25 amino acids or greater may suffer from peptide aggregation and poor solubility. Moreover, the lengths of peptides containing 13–24 amino acid residues were effectively similar in cell uptake.^[^ ^19^ ^]^ Thus, 25 amino acids length fulfilled this requirement.

The mechanism of ALA‐A2 is as a cell penetrating peptide with the ability to induce autophagy‐mediated cancer cell death.^[^ ^20^ ^]^ ALA‐A2 passes through the plasma membrane to the intracellular compartment of A549 lung cancer cells (Figure 4c), and disturbs cancer cell homeostasis. ALA‐A2‐treated A549 cells exhibit downregulation of chaperone proteins involved in autophagic processes, including heat shock 70 kDa protein 8 (*HSPA8*), heat shock protein HSP90 alpha (*HSP90AA1*), and beta (*HSP90AB1*) (Figure 5 and Table 2). Although the exact intracellular targets of ALA‐A2 have yet to be determined, the mode of anticancer actions of ALA‐A2 has shared with previous studies.^[^ ^21^ ^]^

Ding et al.^[^ ^21a^ ^]^ showed that the engineered peptide Trx‐pHLIP‐Beclin1 could induce autophagic cell death in SKOV3 ovarian cancer cells. Pelaz et al.^[^ ^21b^ ^]^ reported that a designed peptide, TAT‐Cx43266‐283, deregulated autophagy and induced glioblastoma stem cell death. Recently, Dent et al.^[^ ^22^ ^]^ reported that the formation of autophagosomes was induced in HCT116 colon cancer following the inhibition of ATPase activities of HSP90 and HSP70. Thus, ACP‐induced autophagy cancer cell death is probably explained, at least in part, by disturbing the expression and function of heat shock proteins. This is consistent with a report by Zhao et al.,^[^ ^21c^ ^]^ in which a novel HSP90 inhibitor, DPB, could inhibit A549 lung cancer growth via inducing apoptosis and autophagy. HSP90 is known as the cancer chaperone which plays a crucial role in maintaining the stability of oncogenic drivers in lung adenocarcinoma.^[^ ^23^ ^]^ Suppression of HSP90 leads to the degradation of oncogenic drivers and a loss of lung cancer cell viability.^[^ ^23^ ^]^ Overexpression of HSP90 has been correlated with a poorer prognosis and chemoresistance in patients with non‐small‐cell lung carcinoma.^[^ ^23^, ^24^ ^]^ The findings reported herein support ALA‐A2 inducing autophagy through downregulation of HSP90 expression in lung adenocarcinoma (Figure 5 and Table 2). Future studies on the molecular interaction between ALA‐A2 and HSP90 would be promising. Ultimately, ALA‐A2 peptide could be tested as an adjuvant therapy to attenuate chemoresistance in non‐small‐cell lung carcinoma.

## 4 Conclusion

This study describes a novel strategy for searching for new ACPs. To validate this strategy, ALA‐A2 was discovered to be a new ACP by integrating the computational peptide library of alpha‐lactalbumin with downstream in silico ACP screening and in vitro experimental validation. ALA‐A2 specifically inhibited lung adenocarcinoma cells through autophagy induction and cancer cell death. This approach, when applied to an expanded computational peptide library based on a large number of proteins, has great potential to minimize the time and cost of therapeutic peptide research.

## 5 Experimental Section

### Cell Culture

All cell lines were obtained from the American Type Culture Collection (ATCC, Manassas, VA, USA). The A549 lung adenocarcinoma (ATCC CCL‐185TM), MDA‐MB‐231 triple‐negative breast adenocarcinoma (ATCC HTB‐26TM), and SH‐SY5Y neuroblastoma (ATCC CRL‐2266TM) cell lines were cultured in Dulbecco's Modified Eagle's Medium (DMEM)‐high glucose (Gibco, Thermo Fisher Scientific, MA, USA). HT29 colon adenocarcinoma (ATCC HTB‐38) cell lines were maintained in DMEM/Nutrient Mixture F‐12 (Gibco). K562 chronic myeloid leukemia (ATCC CRL‐3344) were grown in Roswell Park Memorial Institute Medium 1640 (Gibco). The cells were cultured in medium supplemented with 10% fetal bovine serum (Gibco) and 1× penicillin/streptomycin (Gibco) and incubated in a 5% CO_2_ incubator with saturated humidity.

### Computational Generation of Alpha‐Lactalbumin‐Inspired Peptide Library

The amino acid sequence of human alpha‐lactalbumin retrieved from UniProt ID P00709 (last accessed on 14 September 2020) served as the template for generating the in silico peptide library. A peptide generator, ALA2Peptide, was coded to generate all possible peptides of alpha‐lactalbumin ranging in the optimal length of ACP (5–25 amino acids)^[^ ^2^ ^]^ with stepping 1 amino acid each (from N‐terminal to C‐terminal). This program can be accessed via https://github.com/schuti/ALA2Pept. The output peptide library containing 2688 distinct peptide sequences is provided in Table S1, Supporting Information.

### In Silico ACP Screening

The in silico ACP screening was performed as described^[^ ^5^ ^]^ with a minor modification. Three different ML models: mACPpred (a support vector machine‐based predictor; http://www.thegleelab.org/mACPpred/),^[^ ^25^ ^]^ ACPred‐FL (a sequence‐based predictor; http://server.malab.cn/ACPred-FL/),^[^ ^26^ ^]^ and AntiCP2.0 (an ensemble tree classifier‐based predictor; https://webs.iiitd.edu.in/raghava/anticp2/),^[^ ^27^ ^]^ were used to predict physicochemical and anticancer properties. The geometric mean of ACP probabilities was calculated from the three ML models to serve as the consensus score as well as net charge prediction. The PEP‐FOLD3 de novo peptide structure prediction webserver^[^ ^28^ ^]^ was used to predict the secondary structure of the peptides with ≥3 positive charges (25 out of 2688). Top‐ranked peptide candidates (ALA‐A1, ALA‐A2) were defined as the top ranked peptides by the consensus score with non‐redundant sequence, having ≥3 positive charges, and containing alpha‐helical secondary structure. ALA‐A3 and ALA‐A4, the lower‐ranked positively charged peptides with redundant sequence to ALA‐A1 and ALA‐A2, were included as comparators. BMP‐S6, a known bovine milk‐derived ACP,^[^ ^5^, ^10^ ^]^ served as the positive ACP control, while the untreated condition was the blank (negative) control of this experiment.

### Synthetic Peptides

Five synthetic peptides (purity >98%) were custom‐made by GL Biochem (GL Biochem [Shanghai] Ltd., Shanghai, China). These peptides included ALA‐A1, NH_2_‐RFFVPLFLVGILFPAILAKQFTK‐COOH; ALA‐A2, NH_2_‐KLWCKSSQVPQSR‐COOH; ALA‐A3, NH_2_‐RFFVPLFLVGILFPAILAKQFTKC‐COOH; ALA‐A4, NH_2_‐LFQISNKLWCKSSQVPQSRN‐COOH; and BMP‐S6 (the positive control),^[^ ^5^, ^10^ ^]^ NH_2_‐FKCRRWQWRMKKLGAPSITCVR‐COOH. The peptides were resuspended in fresh media to the final concentration before use.

### In Vitro Cancer Cytotoxicity Screening

The protocol previously described by Chiangjong et al. and Baindara et al.^[^ ^5^, ^29^ ^]^ was modified for use in determining the cytotoxicity of peptides on the various cancer types. Adherent cells were seeded at 1 × 10^4^ cells/well into a 96‐well plate 24 h prior to replacing the media containing the peptide of interest. Suspended cells were seeded at 1 × 10^4^ cells/well in the media containing the peptide of interest. Five human cell lines, including lung cancer (A549), colon cancer (HT29), breast cancer (MDA‐MB‐231), neuroblastoma (SH‐SY5Y), and leukemia (K562) cells, were treated with the culture media containing the peptides at 200 µm for 24 h. Cell viability was then determined using Cell Proliferation Kit I, MTT (Roche Diagnostics GmbH, Mannheim, Germany): MTT labeling reagent (10 µL) was added to each well and incubated at 37 °C in a 5% CO_2_ atmosphere for 4 h. After incubation, 100 µL solubilizing solution was added to each well and incubated at 37 °C in 5% CO_2_ overnight. The absorbance at 570 nm (*A*_570_) was measured for each well. The percentage of cell viability was calculated as:

(1) $$\begin{array}{c} \%\;\text{Cell}\;\text{Viability}=\left[\frac{\text{A570}\;\text{of}\;\text{treated}\;\text{cells}\;-\;\text{A570}\;\text{of}\;\text{media}}{\text{A570}\;\text{of}\;\text{untreated}\;\text{cells}-A570\;\text{of}\;\text{media}}\right]\;\;\times \;100 \end{array}$$

To evaluate dose‐dependency, ≈1 × 10^4^ cells/well were cultured in media containing ALA‐A2 at 0, 6.25, 12.5, 25, 50, 100, 200, and 400 µm in a 96‐well plate for 24 h. Cell viability was determined by the MTT assay, as above.

### Ex Vivo Hemolysis Assay

The hemolysis assay was as described previously.^[^ ^30^ ^]^ After informed consent, peripheral blood (3 mL) was collected from three healthy individuals into heparin tubes (BD Biosciences, NJ, USA). The specimen was centrifuged at 2000 × *g* for 5 min at room temperature, and the plasma was discarded. The RBCs were washed twice with PBS and then resuspended in 0.9% normal saline solution at a volume equivalent to that of the original plasma. Approximately 0.8% v/v RBC suspension was incubated with the peptides at 200 µm (100 µL final volume) in a U‐bottom 96‐well plate at room temperature for 1 h with agitation. 1% v/v Triton X‐100 was applied as a positive control. The untreated condition served as the blank control. After 1 h incubation, the supernatants were collected by centrifuging at 2000 × g for 5 min at room temperature. The absorbance of hemoglobin in the supernatant was measured at 415 nm. The hemolysis percentage was calculated as follows:

(2) $$\begin{array}{c} \%\;\text{Hemolysis}\;=\;\left[\frac{\text{sample}\;\text{absorbance}-\text{blank}\;\text{control}}{\text{positive}\;\text{control}-\text{blank}\;\text{control}}\right]\;\;\times \;100 \end{array}$$

This study was conducted in accordance with the Declaration of Helsinki, and the protocol was approved by the Human Research Ethics Committee, Faculty of Medicine Ramathibodi Hospital, Mahidol University (Protocol ID: MURA2020/759; with approval of amendment on May 5, 2021).

### Confocal Microscopy

In 6‐well plates containing a glass cover slip, A549 lung adenocarcinoma and HT29 colon adenocarcinoma cells were seeded at 2 × 10^5^ cells/2 mL medium/well and incubated for 24 h. The medium was replaced with fresh medium with or without 200 µm FITC‐tagged ALA‐A2 peptide and incubated at 37 °C in 5% CO_2_ for 24 h. All cells were then incubated with PI (1:2000) and Hoechst 33342 (1:5000) in the culture medium at 37 °C in 5% CO_2_ for 15 min. The cells were washed with PBS twice before being fixed with 4% paraformaldehyde (PFA) in PBS. The fixed cells were washed twice with PBS and mounted in 20% glycerol in PBS. After being fixed in PFA, the positive PI staining control cells were permeabilized with 0.5% v/v Triton X‐100 for 10 min. Thereafter, the cells were stained with PI for 15 min at room temperature, washed with PBS, and then mounted with 20% glycerol in PBS. Internalization was visualized by confocal fluorescence microscopy (Nikon Instruments, Inc., Melville, NY, USA).

### SWATH‐Proteomic and Bioinformatic Analysis

Targeted label‐free proteomic analysis using SWATH/DIA was performed as described previously^[^ ^31^ ^]^ to explore the potential mechanisms of ALA‐A2‐induced A549 lung adenocarcinoma cell death. In brief, A549 lung adenocarcinoma cells were seeded at a density of 1 × 10^5^ cells/well in a 24‐well plate. After overnight incubation, the media were changed with fresh media with or without 200 µm ALA‐A2 and incubated at 37 °C in 5%CO_2_ for 24 h. The cells were washed twice with PBS before lysis with Laemmli lysis buffer (62.5 mm Tris‐HCl, pH 6.8, 10% v/v glycerol, 2% w/v SDS, and 2.5% v/v beta‐mercaptoethanol). Total protein was quantified using the Bradford protein assay (Bio‐Rad, Hercules, USA). 10 µg of protein was resolved by 12% SDS–PAGE before staining with Coomassie brilliant blue G‐250, and the gel was cut into small pieces for in‐gel tryptic digestion. An amount of peptide sample corresponding to 2.5 µg of total protein was resolved in an Eksigent nanoLC ultra nanoflow high performance liquid chromatography in tandem with a TripleTOF 6600+ mass spectrometer (ABSciex, Toronto, Canada) set for information‐dependent acquisition (IDA) and DIA modes. The peptides were loaded onto a C18 column trap (Nano Trap RP‐1, 3 µm 120 Å, 10 mm × 0.075 mm; Phenomenex, CA, USA) at a flow rate of 3 µL min^−1^ of 0.1% formic acid in water for 10 min to desalt and concentrate the sample, which was then resolved by HPLC using a stationary phase of a C18 analytical column (bioZen Peptide Polar C18 nanocolumn, 75 µm × 15 cm, particle size 3 µm, 120 Å; Phenomenex) with mobile phase gradients at a flow rate of 300 nL min^−1^ of 3–30% acetonitrile/0.1% formic acid for 60 min, 30–40% acetonitrile/0.1% formic acid for 10 min, 40–80% acetonitrile/0.1% formic acid for 2 min, 80% acetonitrile/0.1% formic acid for 6 min, 80–3% acetonitrile/0.1% formic acid for 2 min, and 3% acetonitrile/0.1% formic acid for 25 min. The eluate was ionized and sprayed into the mass spectrometer using OptiFlow Turbo V Source (Sciex). Ion source gas 1, ion source gas 2, and curtain gas were set at 19, 0, and 25 vendor arbitrary units, respectively. The interface heater temperature was 150 °C and ion spray voltage was 3.3 kV.

Mass spectrometry was operated in the positive ion mode set for 3500 cycles per 105 min gradient elution. Each cycle performed one time of flight (TOF) scan (250 ms accumulation time, 350–1250 *m/z* window with a charge state of +2) followed by IDA of the 100 most intense ions, while the minimum MS signal was set to 150 counts. The MS/MS scan was operated in high sensitivity mode with 50 ms accumulation time and 50 ppm mass tolerance. Former MS/MS candidate ions were excluded for a period of 12 s after their first occurrence to reduce the redundancy of identified peptides. DIA mode was performed in a range of 350–1500 *m/z* using a predefined mass window of 7‐*m/z* with an overlap of 1‐*m/z* for 157 transmissible windows. MS scan was set at 2044 cycles, where each cycle performed one TOF‐MS scan type (50 ms accumulation time across 100–1500 precursor mass range) acquired in every cycle for a total cycle time of 3.08 s. MS spectra of 100–1500 *m/z* were collected with an accumulation time of 96 ms per SWATH window width. Resolution for MS1 was 35 000 and SWATH‐MS2 scan was 30 000. Rolling collision energy mode with collision energy spread of 15 eV was applied. The IDA and DIA data (.wiff) were recorded by Analyst‐TF v.1.8 software (ABSciex).

A total of 18 wiff files of IDA experiments (two groups; three biological replicates per group; three technical replicates per biological sample) were combined and searched using Protein Pilot v.5.0.2.0 software (ABSciex) against the Swiss‐Prot database (UniProtKB 2022_01) Homo sapiens (20385 proteins in database) with the searching parameters as follows; alkylation on cysteine by iodoacetamide, trypsin enzymatic digestion, one missed cleavage allowed, monoisotopic mass, and 1% false discovery rate. The group file (Protein Pilot search result) was loaded into SWATH Acquisition MicroApp v.2.0.1.2133 in PeakView software v.2.2 (Sciex) to generate a spectral library. The maximum number of proteins was set as the number of proteins identified at 1% global FDR from fit. RT alignment was performed by the high abundance endogenous peptides covering the chromatographic range. SWATH data extraction of 18 DIA files (two groups; three biological replicates per group; three technical replicates per biological sample) was performed by SWATH Acquisition MicroApp (Sciex) using the following parameters; 5‐min extraction window, 25 peptides/protein, 6 transitions/peptide, excluding shared peptides, peptide confidence >99%, FDR <1%, and XIC width of 20 ppm. SWATH extraction data, including the identities and quantities of peptides and proteins, was exported into an Excel file for further analysis.

Comparative proteomic analysis was performed by using R program as described previously,^[^ ^31b^ ^]^ including log2 transformation, VSN normalization, missing value imputation by median, differential expression analysis, heatmap, and volcano plot at the thresholds of 2× fold change and *p*‐value <0.05. The pathway enrichment analysis was analyzed by Reactome v.78^[^ ^32^ ^]^ (last accessed on February 2, 2022) using the differential expressed proteins as the input, where a matched pathway with FDR <0.05 was considered statistically significant.

### Autophagy Activity Assay

A549 cells (1 × 10^4^ cells/well in a 96‐well plate) were treated with 200 µm ALA‐A2. Treated cells were maintained for 24 h before determining autophagy activity using an autophagy assay kit (ab139484, Abcam, Milpitas, CA). To prepare for the staining, the media was removed, and the cells were washed once with 1× assay buffer. The dual color detection solution was added to the wells and incubated for 30 min. The cells were washed and 1× assay buffer added to each well. The nuclear signal was excitation of 340 nm and emission of 480, whereas autophagosomes and autophagolysosomes were detected by excitation of 480 nm and emission of 530 nm. Autophagy activity was calculated by normalizing the autophagosome/autophagolysosome signal to the nuclear signal. Rapamycin (2 µm), a known autophagy inducer, was the positive control.^[^ ^33^ ^]^ This experiment was performed in three biological replicates.

### Statistical Analysis

Data were presented as the number, percentage, or mean ± SD as appropriate. One‐way ANOVA with Tukey's post hoc tests for multiple comparisons was performed. A *p*‐value <0.05 was considered statistically significant.

### Ethical Approval Statement

The study was conducted in accordance with the Declaration of Helsinki, and the protocol was approved by the Human Research Ethics Committee, Faculty of Medicine Ramathibodi Hospital, Mahidol University (Protocol ID: MURA2020/759; with approval of amendment on May 5, 2021).

### Informed Consent Statement

Informed consent was obtained from all subjects involved in the study.

## Conflict of Interest

The authors declare no conflict of interest.

## Author Contributions

T.L. and P.O.‐y. contributed equally to this work. Conceptualization: So.C.; Methodology: T.L., P.O.‐y., W.C., So.C.; Validation: W.C., So.C.; Formal analysis: T.L., P.O.‐y., Se.C., W.S., W.C., So.C.; Investigation: T.L., P.O.‐y., Se.C., W.S., W.C., So.C.; Resources: D.S.N., A.L.M., S.H., So.C.; Software: P.O.‐y., So.C.; Writing—original draft preparation: T.L., P.O.‐y.; Writing—review and editing: Se.C., W.S., D.S.N., A.L.M., S.H., W.C., So.C.; Visualization: T.L., P.O.‐y., So.C.; Supervision: D.S.N., A.L.M., S.H., W.C., So.C.; Funding acquisition: So.C. All authors have read and agreed to the published version of the manuscript.

## Supporting information

Supporting Information

## Data Availability

The data that support the findings of this study are available in the supplementary material of this article.
