# Supplementary material for: ALA‐A2 Is a Novel Anticancer Peptide Inspired by Alpha‐Lactalbumin: A Discovery from a Computational Peptide Library, In Silico Anticancer Peptide Screening and In Vitro Experimental Validation
Source: Glob Chall. 2023 Jan 12;7(3):2200213. doi: 10.1002/gch2.202200213 (PMC10000267; doi:10.1002/gch2.202200213)
Supplement: Supplementary file 1 — Supporting Information [file GCH2-7-2200213-s001.pdf]

Global Challenges
Open Access

Supporting Information

for *Global Challenges,* DOI: 10.1002/gch2.202200213

ALA-A2 Is a Novel Anticancer Peptide Inspired by Alpha-Lactalbumin: A Discovery from a Computational Peptide Library, In Silico Anticancer Peptide Screening and In Vitro Experimental Validation

Tassanee Lerksuthirat, Pasinee On-yam, Sermsiri Chitphuk, Wasana Stitchantrakul, David S. Newburg, Ardythe L. Morrow, Suradej Hongeng, Wararat Chiangjong, and Somchai Chutipongtanate**

Supporting Information

ALA-A2 is a novel anticancer peptide inspired by alpha-lactalbumin:
A discovery from a computational peptide library, *in silico* anticancer peptide screening and *in vitro* experimental validation

Tassanee Lerksuthirat,[#] Pasinee On-yam,[#] Sermsiri Chitphuk, Wasana Stitchantrakul, David S. Newburg, Ardythe L. Morrow, Suradej Hongeng, Wararat Chiangjong and Somchai Chutipongtanate**

This PDF file contains three supplementary tables as follows;.

Table S1. ACP prediction and screening of 2688 generated alpha-lactalbumin peptides. *(pages 2-44)*

Table S2. Processed and raw SWATH-intensity proteomic data sets of ALA-A2 treated (ACP) vs. untreated (Ctrl) A549 cells. *(pages 45-47)*

Table S3. Reactome pathway enrichment analysis of the significantly differentially expressed proteins of ALA-A2 treated A549 cells. *(pages 48-56)*

Table S1: Physicochemical properties and ACP probabilities by three machine learning models of 2688 all possible peptides generated in silico from alpha lactalbumin.

No.	peptide sequence	peptide length	Hydrophobicity	Steric hindrance	Sidebulk	Hydropathicity	Amphipathicity	Hydrophilicity	Net Hydrogen	Charge	pI	Molecular weight	ACPred-FL	Anticp2	mACPpred
1	MRFFV	5	0.05	0.71	0.71	1.44	0.49	-0.96	0.8	1	10.11	698.94	no data	0.68	0.98
2	RFFVP	5	-0.01	0.63	0.63	0.74	0.49	-0.7	0.8	1	10.11	664.86	no data	0.74	0.93
3	FFVPL	5	0.44	0.6	0.6	2.4	0	-1.66	0	0	5.88	621.84	no data	0.77	0.95
4	FVPLF	5	0.44	0.6	0.6	2.4	0	-1.66	0	0	5.88	621.84	no data	0.82	0.98
5	VPLFL	5	0.43	0.56	0.56	2.6	0	-1.52	0	0	5.88	587.83	no data	0.79	0.95
6	PLFLV	5	0.43	0.56	0.56	2.6	0	-1.52	0	0	5.88	587.83	no data	0.73	0.94
7	LFLVG	5	0.47	0.63	0.63	2.84	0	-1.52	0	0	5.88	547.77	no data	0.71	0.98
8	FLVGI	5	0.51	0.66	0.66	2.98	0	-1.52	0	0	5.88	547.77	no data	0.67	0.95
9	LVGIL	5	0.5	0.63	0.63	3.18	0	-1.38	0	0	5.88	513.76	no data	0.55	0.98
10	VGILF	5	0.51	0.66	0.66	2.98	0	-1.52	0	0	5.88	547.77	no data	0.65	0.98
11	GILFP	5	0.39	0.59	0.59	1.82	0	-1.22	0	0	5.88	545.75	no data	0.64	0.98
12	ILFPA	5	0.41	0.56	0.56	2.26	0	-1.32	0	0	5.88	559.77	no data	0.73	0.97
13	LFPAI	5	0.41	0.56	0.56	2.26	0	-1.32	0	0	5.88	559.77	no data	0.58	0.93
14	FPAIL	5	0.41	0.56	0.56	2.26	0	-1.32	0	0	5.88	559.77	no data	0.64	0.96
15	PAILA	5	0.34	0.53	0.53	2.06	0	-0.92	0	0	5.88	483.67	no data	0.77	0.97
16	AILAK	5	0.13	0.59	0.59	1.6	0.73	-0.32	0.4	1	9.11	514.73	no data	0.82	0.98
17	ILAKQ	5	-0.06	0.62	0.62	0.54	0.98	-0.18	0.8	1	9.11	571.79	no data	0.89	0.97
18	LAKQF	5	-0.08	0.62	0.62	0.2	0.98	-0.32	0.8	1	9.11	605.8	no data	0.91	0.97
19	AKQFT	5	-0.22	0.62	0.62	-0.7	0.98	-0.04	1	1	9.11	593.74	no data	0.72	0.96
20	KQFTK	5	-0.49	0.65	0.65	-1.84	1.72	0.66	1.4	2	10.02	650.84	no data	0.51	0.98
21	QFTKC	5	-0.26	0.64	0.64	-0.56	0.98	-0.14	1	1	8.57	625.8	no data	0.40	0.92
22	FTKCE	5	-0.25	0.64	0.64	-0.56	0.99	0.42	0.8	0	6.32	626.78	no data	0.40	0.94
23	TKCEL	5	-0.27	0.61	0.61	-0.36	0.99	0.56	0.8	0	6.32	592.77	no data	0.41	0.71
24	KCELS	5	-0.28	0.61	0.61	-0.38	0.99	0.7	0.8	0	6.32	578.74	no data	0.30	0.69
25	CELSQ	5	-0.2	0.61	0.61	-0.3	0.5	0.14	0.8	-1	4	578.7	no data	0.43	0.57
26	ELSQL	5	-0.1	0.59	0.59	-0.04	0.5	-0.02	0.8	-1	4	588.73	no data	0.30	0.67
27	LSQLL	5	0.13	0.56	0.56	1.42	0.25	-0.98	0.6	0	5.88	572.78	no data	0.37	0.98
28	SQLLK	5	-0.2	0.59	0.59	-0.12	0.98	-0.02	1	1	9.11	587.79	no data	0.28	0.98
29	QLLKD	5	-0.29	0.64	0.64	-0.66	0.98	0.52	1	0	6.19	615.8	no data	0.26	0.81
30	LLKDI	5	-0.01	0.64	0.64	0.94	0.73	0.12	0.6	0	6.19	600.83	no data	0.40	0.91
31	LKDID	5	-0.26	0.69	0.69	-0.52	0.73	1.08	0.8	-1	4.21	602.75	no data	0.43	0.80
32	KDIDG	5	-0.33	0.72	0.72	-1.36	0.73	1.44	0.8	-1	4.21	546.64	no data	0.47	0.29
33	DIDGY	5	-0.11	0.72	0.72	-0.84	0	0.38	0.6	-2	3.57	581.64	no data	0.55	0.43
34	IDGYG	5	0.07	0.7	0.7	-0.22	0	-0.22	0.4	-1	3.8	523.61	no data	0.59	0.63
35	DGYGG	5	-0.04	0.7	0.7	-1.2	0	0.14	0.4	-1	3.8	467.5	no data	0.55	0.85
36	GYGGI	5	0.25	0.69	0.69	0.4	0	-0.82	0.2	0	5.88	465.58	no data	0.55	0.98
37	YGGIA	5	0.26	0.66	0.66	0.84	0	-0.92	0.2	0	5.88	479.6	no data	0.60	0.94
38	GGIAL	5	0.37	0.62	0.62	1.86	0	-0.82	0	0	5.88	429.59	no data	0.75	0.98
39	GIALP	5	0.32	0.56	0.56	1.62	0	-0.82	0	0	5.88	469.65	no data	0.78	0.98
40	IALPE	5	0.16	0.56	0.56	1	0.25	-0.22	0.2	-1	4	541.71	no data	0.78	0.90
41	ALPEL	5	0.12	0.52	0.52	0.86	0.25	-0.22	0.2	-1	4	541.71	no data	0.73	0.89
42	LPELI	5	0.22	0.56	0.56	1.4	0.25	-0.48	0.2	-1	4	583.8	no data	0.61	0.63
43	PELIC	5	0.12	0.58	0.58	1.14	0.25	-0.32	0.2	-1	4	573.77	no data	0.51	0.80
44	ELICT	5	0.1	0.61	0.61	1.32	0.25	-0.4	0.4	-1	4	577.76	no data	0.48	0.84
45	LICTM	5	0.28	0.63	0.63	2.4	0	-1.26	0.2	0	5.85	579.84	no data	0.60	0.98
46	ICTMF	5	0.29	0.67	0.67	2.2	0	-1.4	0.2	0	5.85	613.85	no data	0.60	0.97
47	CTMFH	5	0.07	0.53	0.53	0.66	0.29	-1.14	0.4	0.5	7.06	637.83	no data	0.66	0.93
48	TMFHT	5	0.02	0.51	0.51	0.02	0.29	-1.02	0.6	0.5	7.1	635.8	no data	0.70	0.91
49	MFHTS	5	0.01	0.51	0.51	0	0.29	-0.88	0.6	0.5	7.1	621.77	no data	0.56	0.64
50	FHTSG	5	-0.01	0.49	0.49	-0.46	0.29	-0.62	0.6	0.5	7.1	547.63	no data	0.37	0.98
51	HTSGY	5	-0.13	0.49	0.49	-1.28	0.29	-0.58	0.8	0.5	7.09	563.63	no data	0.28	0.87
52	TSGYD	5	-0.2	0.64	0.64	-1.34	0	0.12	0.8	-1	3.8	541.57	no data	0.22	0.28
53	SGYDT	5	-0.2	0.64	0.64	-1.34	0	0.12	0.8	-1	3.8	541.57	no data	0.27	0.19
54	GYDTQ	5	-0.28	0.67	0.67	-1.88	0.25	0.1	1	-1	3.8	582.63	no data	0.59	0.26
55	YDTQA	5	-0.26	0.64	0.64	-1.44	0.25	0	1	-1	3.8	596.65	no data	0.52	0.64
56	DTQAI	5	-0.12	0.64	0.64	-0.28	0.25	0.1	0.8	-1	3.8	546.64	no data	0.37	0.76
57	TQAIV	5	0.13	0.63	0.63	1.26	0.25	-0.8	0.6	0	5.88	530.69	no data	0.48	0.90
58	QAIVE	5	0.04	0.66	0.66	0.7	0.5	-0.12	0.6	-1	4	558.7	no data	0.54	0.22
59	AIVEN	5	0.05	0.67	0.67	0.7	0.25	-0.12	0.6	-1	4	544.67	no data	0.59	0.56
60	IVENN	5	-0.13	0.72	0.72	-0.36	0.25	0.02	1	-1	4	587.7	no data	0.71	0.72
61	VENNE	5	-0.4	0.72	0.72	-1.96	0.51	0.98	1.2	-2	3.8	603.65	no data	0.43	0.61
62	ENNES	5	-0.56	0.68	0.68	-2.96	0.51	1.34	1.4	-2	3.8	591.59	no data	0.35	0.52

63	NNEST	5	-0.47	0.65	0.65	-2.4	0.25	0.66	1.4	-1	4	563.58	no data	0.29	0.42
64	NESTE	5	-0.46	0.64	0.64	-2.4	0.51	1.22	1.2	-2	3.8	578.59	no data	0.30	0.42
65	ESTEY	5	-0.33	0.62	0.62	-1.96	0.51	0.72	1	-2	3.8	627.66	no data	0.43	0.41
66	STEYG	5	-0.18	0.62	0.62	-1.34	0.25	0.12	0.8	-1	4	555.6	no data	0.45	0.29
67	TEYGL	5	-0.02	0.62	0.62	-0.42	0.25	-0.3	0.6	-1	4	581.69	no data	0.46	0.29
68	EYGLF	5	0.14	0.66	0.66	0.28	0.25	-0.72	0.4	-1	4	627.76	no data	0.68	0.57
69	YGLFQ	5	0.13	0.66	0.66	0.28	0.25	-1.28	0.6	0	5.88	626.78	no data	0.65	0.71
70	GLFQI	5	0.27	0.66	0.66	1.44	0.25	-1.18	0.4	0	5.88	576.77	no data	0.65	0.96
71	LFQIS	5	0.18	0.63	0.63	1.36	0.25	-1.12	0.6	0	5.88	606.79	no data	0.68	0.97
72	FQISN	5	-0.05	0.67	0.67	-0.1	0.25	-0.72	1	0	5.88	607.73	no data	0.53	0.54
73	QISNK	5	-0.39	0.67	0.67	-1.44	0.98	0.38	1.4	1	9.11	588.73	no data	0.59	0.24
74	ISNKL	5	-0.15	0.64	0.64	0.02	0.73	-0.02	1	1	9.11	573.76	no data	0.78	0.87
75	SNKLW	5	-0.22	0.6	0.6	-1.06	0.73	-0.34	1.2	1	9.11	646.81	no data	0.76	0.95
76	NKLWC	5	-0.16	0.62	0.62	-0.4	0.73	-0.6	1	1	8.57	662.87	no data	0.79	0.96
77	KLWCK	5	-0.25	0.6	0.6	-0.48	1.47	-0.04	1	2	9.36	676.94	no data	0.71	0.98
78	LWCKS	5	-0.08	0.57	0.57	0.14	0.73	-0.58	0.8	1	8.57	635.84	no data	0.53	0.90
79	WCKSS	5	-0.24	0.57	0.57	-0.78	0.73	-0.16	1	1	8.57	609.75	no data	0.43	0.90
80	CKSSQ	5	-0.45	0.61	0.61	-1.3	0.98	0.56	1.2	1	8.57	551.67	no data	0.42	0.92
81	KSSQV	5	-0.35	0.62	0.62	-0.96	0.98	0.46	1.2	1	9.11	547.67	no data	0.74	0.30
82	SSQVP	5	-0.15	0.56	0.56	-0.5	0.25	-0.14	0.8	0	5.88	516.61	no data	0.68	0.62
83	SQVPQ	5	-0.23	0.59	0.59	-1.04	0.5	-0.16	1	0	5.88	557.67	no data	0.60	0.69
84	QVPQS	5	-0.23	0.59	0.59	-1.04	0.5	-0.16	1	0	5.88	557.67	no data	0.51	0.47
85	VPQSR	5	-0.45	0.59	0.59	-1.24	0.74	0.4	1.4	1	10.11	585.72	no data	0.39	0.34
86	PQSRN	5	-0.68	0.6	0.6	-2.78	0.74	0.74	1.8	1	10.11	600.69	no data	0.35	0.64
87	QSRNI	5	-0.52	0.67	0.67	-1.56	0.74	0.38	1.8	1	10.11	616.74	no data	0.43	0.23
88	SRNIC	5	-0.38	0.66	0.66	-0.36	0.49	0.14	1.4	1	8.6	591.74	no data	0.50	0.98
89	RNICD	5	-0.47	0.7	0.7	-0.9	0.49	0.68	1.4	0	6.16	619.75	no data	0.57	0.93
90	NICDI	5	0.03	0.71	0.71	0.9	0	-0.28	0.6	-1	3.8	576.73	no data	0.52	0.87
91	ICDIS	5	0.1	0.66	0.66	1.44	0	-0.26	0.4	-1	3.8	549.7	no data	0.59	0.92
92	CDISC	5	-0.03	0.65	0.65	1.04	0	-0.1	0.4	-1	3.8	539.67	no data	0.56	0.97
93	DISCD	5	-0.19	0.67	0.67	-0.16	0	0.7	0.6	-2	3.57	551.62	no data	0.56	0.67
94	ISCDK	5	-0.26	0.66	0.66	-0.24	0.73	0.7	0.8	0	6.16	564.71	no data	0.44	0.94
95	SCDKF	5	-0.29	0.66	0.66	-0.58	0.73	0.56	0.8	0	6.16	598.72	no data	0.51	0.93
96	CDKFL	5	-0.13	0.66	0.66	0.34	0.73	0.14	0.6	0	6.16	624.81	no data	0.72	0.98
97	DKFLD	5	-0.28	0.69	0.69	-0.86	0.73	0.94	0.8	-1	4.21	636.76	no data	0.46	0.94
98	KFLDD	5	-0.28	0.69	0.69	-0.86	0.73	0.94	0.8	-1	4.21	636.76	no data	0.53	0.88
99	FLDDD	5	-0.2	0.7	0.7	-0.78	0	0.94	0.6	-3	3.43	623.67	no data	0.49	0.90
100	LDDDI	5	-0.18	0.7	0.7	-0.44	0	1.08	0.6	-3	3.43	589.66	no data	0.30	0.66
101	DDDIT	5	-0.32	0.7	0.7	-1.34	0	1.36	0.8	-3	3.43	577.6	no data	0.54	0.81
102	DDITD	5	-0.32	0.7	0.7	-1.34	0	1.36	0.8	-3	3.43	577.6	no data	0.46	0.84
103	DITDD	5	-0.32	0.7	0.7	-1.34	0	1.36	0.8	-3	3.43	577.6	no data	0.46	0.76
104	ITDDI	5	-0.03	0.69	0.69	0.26	0	0.4	0.6	-2	3.57	575.68	no data	0.46	0.76
105	TDDIM	5	-0.13	0.71	0.71	-0.26	0	0.5	0.6	-2	3.57	593.71	no data	0.38	0.17
106	DDIMC	5	-0.08	0.72	0.72	0.38	0	0.38	0.4	-2	3.57	595.74	no data	0.52	0.65
107	DIMCA	5	0.11	0.68	0.68	1.44	0	-0.32	0.2	-1	3.8	551.73	no data	0.57	0.64
108	IMCAK	5	0.04	0.66	0.66	1.36	0.73	-0.32	0.4	1	8.57	564.82	no data	0.81	0.98
109	MCAKK	5	-0.33	0.66	0.66	-0.32	1.47	0.64	0.8	2	9.36	579.83	no data	0.93	0.98
110	CAKKI	5	-0.24	0.64	0.64	0.2	1.47	0.54	0.8	2	9.36	561.8	no data	0.96	0.98
111	AKKIL	5	-0.14	0.62	0.62	0.46	1.47	0.38	0.8	2	10.02	571.83	no data	0.94	0.98
112	KKILD	5	-0.33	0.67	0.67	-0.6	1.47	1.08	1	1	8.94	615.84	no data	0.68	0.91
113	KILDI	5	0.03	0.67	0.67	1.08	0.73	0.12	0.6	0	6.19	600.83	no data	0.59	0.84
114	ILDIK	5	0.03	0.67	0.67	1.08	0.73	0.12	0.6	0	6.19	600.83	no data	0.51	0.80
115	LDIKG	5	-0.08	0.67	0.67	0.1	0.73	0.48	0.6	0	6.19	544.72	no data	0.36	0.70
116	DIKGI	5	-0.04	0.7	0.7	0.24	0.73	0.48	0.6	0	6.19	544.72	no data	0.39	0.75
117	IKGID	5	-0.04	0.7	0.7	0.24	0.73	0.48	0.6	0	6.19	544.72	no data	0.37	0.75
118	KGIDY	5	-0.18	0.7	0.7	-0.92	0.73	0.38	0.8	0	6.18	594.73	no data	0.31	0.35
119	GIDYW	5	0.11	0.67	0.67	-0.32	0	-0.9	0.6	-1	3.8	652.77	no data	0.71	0.69
120	IDYWL	5	0.19	0.64	0.64	0.52	0	-1.26	0.6	-1	3.8	708.88	no data	0.49	0.92
121	DYWLA	5	0.09	0.6	0.6	-0.02	0	-1	0.6	-1	3.8	666.79	no data	0.72	0.98
122	YWLAH	5	0.15	0.45	0.45	0.04	0.29	-1.7	0.6	0.5	7.09	688.85	no data	0.72	0.98
123	WLAHK	5	-0.07	0.45	0.45	-0.48	1.02	-0.64	0.8	1.5	9.11	653.85	no data	0.60	0.98
124	LAHKA	5	-0.09	0.45	0.45	0.06	1.02	-0.06	0.6	1.5	9.11	538.71	no data	0.87	0.98
125	AHKAL	5	-0.09	0.45	0.45	0.06	1.02	-0.06	0.6	1.5	9.11	538.71	no data	0.94	0.97
126	HKALC	5	-0.14	0.47	0.47	0.2	1.02	-0.16	0.6	1.5	8.57	570.77	no data	0.82	0.97

127	KALCT	5	-0.09	0.58	0.58	0.7	0.73	-0.14	0.6	1	8.57	534.73	no data	0.83	0.97
128	ALCTE	5	0	0.58	0.58	0.78	0.25	-0.14	0.4	-1	4	535.67	no data	0.57	0.99
129	LCTEK	5	-0.27	0.61	0.61	-0.36	0.99	0.56	0.8	0	6.32	592.77	no data	0.21	0.77
130	CTEKL	5	-0.27	0.61	0.61	-0.36	0.99	0.56	0.8	0	6.32	592.77	no data	0.60	0.79
131	TEKLE	5	-0.4	0.62	0.62	-1.56	1.24	1.36	1	-1	4.54	618.75	no data	0.55	0.37
132	EKLEQ	5	-0.5	0.65	0.65	-2.12	1.49	1.48	1.2	-1	4.54	645.78	no data	0.52	0.34
133	KLEQW	5	-0.3	0.61	0.61	-1.6	1.24	0.2	1.2	0	6.35	702.88	no data	0.60	0.68
134	LEQWL	5	0.02	0.58	0.58	-0.06	0.5	-0.76	0.8	-1	4	687.87	no data	0.13	0.94
135	EQWLC	5	-0.07	0.6	0.6	-0.32	0.5	-0.6	0.8	-1	4	677.84	no data	0.12	0.95
136	QWLCE	5	-0.07	0.6	0.6	-0.32	0.5	-0.6	0.8	-1	4	677.84	no data	0.14	0.98
137	WLCEK	5	-0.16	0.6	0.6	-0.4	0.99	-0.04	0.8	0	6.32	677.88	no data	0.10	0.81
138	LCEKL	5	-0.12	0.61	0.61	0.54	0.99	0.28	0.6	0	6.32	604.83	no data	0.45	0.95
139	MRFFVP	6	0.03	0.65	0.65	0.93	0.41	-0.8	0.67	1	10.11	796.07	no data	0.68	0.96
140	RFFVPL	6	0.08	0.61	0.61	1.25	0.41	-0.88	0.67	1	10.11	778.04	no data	0.76	0.91
141	FFVPLF	6	0.47	0.61	0.61	2.47	0	-1.8	0	0	5.88	769.03	no data	0.83	0.98
142	FVPLFL	6	0.46	0.59	0.59	2.63	0	-1.68	0	0	5.88	735.02	no data	0.78	0.98
143	VPLFLV	6	0.45	0.59	0.59	2.87	0	-1.52	0	0	5.88	686.98	no data	0.70	0.91
144	PLFLVG	6	0.38	0.58	0.58	2.1	0	-1.27	0	0	5.88	644.9	no data	0.72	0.98
145	LFLVGI	6	0.52	0.64	0.64	3.12	0	-1.57	0	0	5.88	660.95	no data	0.67	0.86
146	FLVGIL	6	0.52	0.64	0.64	3.12	0	-1.57	0	0	5.88	660.95	no data	0.67	0.98
147	LVGILF	6	0.52	0.64	0.64	3.12	0	-1.57	0	0	5.88	660.95	no data	0.51	0.87
148	VGILFP	6	0.42	0.61	0.61	2.22	0	-1.27	0	0	5.88	644.9	no data	0.62	0.98
149	GILFPA	6	0.37	0.58	0.58	1.82	0	-1.1	0	0	5.88	616.84	no data	0.69	0.98
150	ILFPAI	6	0.46	0.58	0.58	2.63	0	-1.4	0	0	5.88	672.95	no data	0.62	0.98
151	LFPAIL	6	0.43	0.56	0.56	2.52	0	-1.4	0	0	5.88	672.95	no data	0.62	0.91
152	FPAILA	6	0.38	0.56	0.56	2.18	0	-1.18	0	0	5.88	630.86	no data	0.73	0.97
153	PAILAK	6	0.1	0.55	0.55	1.07	0.61	-0.27	0.33	1	9.11	611.86	no data	0.80	0.98
154	AILAKQ	6	-0.01	0.61	0.61	0.75	0.82	-0.23	0.67	1	9.11	642.88	no data	0.71	0.98
155	ILAKQF	6	0.05	0.64	0.64	0.92	0.82	-0.57	0.67	1	9.11	718.98	no data	0.85	0.98
156	LAKQFT	6	-0.1	0.61	0.61	0.05	0.82	-0.33	0.83	1	9.11	706.92	no data	0.87	0.96
157	AKQFTK	6	-0.37	0.63	0.63	-1.23	1.43	0.47	1.17	2	10.02	721.93	no data	0.72	0.95
158	KQFTKC	6	-0.4	0.65	0.65	-1.12	1.43	0.38	1.17	2	9.36	753.99	no data	0.40	0.96
159	QFTKCE	6	-0.32	0.65	0.65	-1.05	1.03	0.38	1	0	6.32	754.93	no data	0.40	0.43
160	FTKCEL	6	-0.12	0.62	0.62	0.17	0.82	0.05	0.67	0	6.32	739.96	no data	0.37	0.79
161	TKCELS	6	-0.26	0.6	0.6	-0.43	0.82	0.52	0.83	0	6.32	679.86	no data	0.37	0.45
162	KCELSQ	6	-0.35	0.62	0.62	-0.9	1.03	0.62	1	0	6.32	706.89	no data	0.37	0.44
163	CELSQL	6	-0.08	0.6	0.6	0.38	0.42	-0.18	0.67	-1	4	691.88	no data	0.25	0.56
164	ELSQLL	6	0	0.58	0.58	0.6	0.42	-0.32	0.67	-1	4	701.91	no data	0.29	0.96
165	LSQLLK	6	-0.08	0.58	0.58	0.53	0.82	-0.32	0.83	1	9.11	700.97	no data	0.26	0.97
166	SQLLKD	6	-0.28	0.62	0.62	-0.68	0.82	0.48	1	0	6.19	702.89	no data	0.26	0.80
167	QLLKDI	6	-0.12	0.65	0.65	0.2	0.82	0.13	0.83	0	6.19	728.98	no data	0.28	0.75
168	LLKDID	6	-0.12	0.66	0.66	0.2	0.61	0.6	0.67	-1	4.21	715.93	no data	0.45	0.94
169	LKDIDG	6	-0.19	0.68	0.68	-0.5	0.61	0.9	0.67	-1	4.21	659.82	no data	0.44	0.18
170	KDIDGY	6	-0.27	0.71	0.71	-1.35	0.61	0.82	0.83	-1	4.21	709.83	no data	0.44	0.05
171	DIDGYG	6	-0.06	0.71	0.71	-0.77	0	0.32	0.5	-2	3.57	638.71	no data	0.55	0.31
172	IDGYGG	6	0.09	0.7	0.7	-0.25	0	-0.18	0.33	-1	3.8	580.68	no data	0.59	0.80
173	DGYGGI	6	0.09	0.7	0.7	-0.25	0	-0.18	0.33	-1	3.8	580.68	no data	0.55	0.79
174	GYGGIA	6	0.25	0.66	0.66	0.63	0	-0.77	0.17	0	5.88	536.67	no data	0.62	0.98
175	YGGIAL	6	0.31	0.63	0.63	1.33	0	-1.07	0.17	0	5.88	592.78	no data	0.74	0.96
176	GGIALP	6	0.29	0.58	0.58	1.28	0	-0.68	0	0	5.88	526.72	no data	0.75	0.98
177	GIALPE	6	0.16	0.58	0.58	0.77	0.21	-0.18	0.17	-1	4	598.78	no data	0.77	0.81
178	IALPEL	6	0.23	0.55	0.55	1.47	0.21	-0.48	0.17	-1	4	654.89	no data	0.69	0.74
179	ALPELI	6	0.23	0.55	0.55	1.47	0.21	-0.48	0.17	-1	4	654.89	no data	0.71	0.71
180	LPELIC	6	0.19	0.57	0.57	1.58	0.21	-0.57	0.17	-1	4	686.95	no data	0.59	0.80
181	PELICT	6	0.07	0.57	0.57	0.83	0.21	-0.33	0.33	-1	4	674.89	no data	0.51	0.72
182	ELICTM	6	0.13	0.64	0.64	1.42	0.21	-0.55	0.33	-1	4	708.97	no data	0.52	0.79
183	LICTMF	6	0.33	0.64	0.64	2.47	0	-1.47	0.17	0	5.85	727.03	no data	0.58	0.97
184	ICTMFH	6	0.18	0.56	0.56	1.3	0.24	-1.25	0.33	0.5	7.06	751.01	no data	0.64	0.96
185	CTMFHT	6	0.02	0.53	0.53	0.43	0.24	-1.02	0.5	0.5	7.06	738.95	no data	0.66	0.87
186	TMFHTS	6	-0.03	0.51	0.51	-0.12	0.24	-0.8	0.67	0.5	7.1	722.89	no data	0.59	0.89
187	MFHTSG	6	0.03	0.54	0.54	-0.07	0.24	-0.73	0.5	0.5	7.1	678.84	no data	0.37	0.38
188	FHTSGY	6	-0.01	0.52	0.52	-0.6	0.24	-0.9	0.67	0.5	7.09	710.82	no data	0.34	0.86
189	HTSGYD	6	-0.23	0.53	0.53	-1.65	0.24	0.02	0.83	-0.5	5.09	678.73	no data	0.27	0.20
190	TSGYDT	6	-0.19	0.62	0.62	-1.23	0	0.03	0.83	-1	3.8	642.69	no data	0.21	0.26

191	SGYDTQ	6	-0.28	0.65	0.65	-1.7	0.21	0.13	1	-1	3.8	669.72	no data	0.31	0.16
192	GYDTQA	6	-0.19	0.65	0.65	-1.27	0.21	0	0.83	-1	3.8	653.72	no data	0.54	0.24
193	YDTQAI	6	-0.1	0.65	0.65	-0.45	0.21	-0.3	0.83	-1	3.8	709.83	no data	0.36	0.43
194	DTQAIV	6	-0.01	0.65	0.65	0.47	0.21	-0.17	0.67	-1	3.8	645.79	no data	0.45	0.47
195	TQAIVE	6	0.01	0.64	0.64	0.47	0.42	-0.17	0.67	-1	4	659.82	no data	0.56	0.44
196	QAIVEN	6	-0.07	0.67	0.67	0	0.42	-0.07	0.83	-1	4	672.82	no data	0.55	0.30
197	AIVENN	6	-0.06	0.69	0.69	0	0.21	-0.07	0.83	-1	4	658.79	no data	0.56	0.55
198	IVENNE	6	-0.21	0.71	0.71	-0.88	0.42	0.52	1	-2	3.8	716.83	no data	0.53	0.38
199	VENNES	6	-0.37	0.68	0.68	-1.77	0.42	0.87	1.17	-2	3.8	690.74	no data	0.42	0.26
200	ENNEST	6	-0.49	0.66	0.66	-2.58	0.42	1.05	1.33	-2	3.8	692.71	no data	0.31	0.39
201	NNESTE	6	-0.49	0.66	0.66	-2.58	0.42	1.05	1.33	-2	3.8	692.71	no data	0.27	0.33
202	NESTEY	6	-0.38	0.65	0.65	-2.22	0.42	0.63	1.17	-2	3.8	741.78	no data	0.30	0.37
203	ESTEYG	6	-0.25	0.63	0.63	-1.7	0.42	0.6	0.83	-2	3.8	684.73	no data	0.44	0.11
204	STEYGL	6	-0.06	0.61	0.61	-0.48	0.21	-0.2	0.67	-1	4	668.78	no data	0.41	0.26
205	TEYGLF	6	0.09	0.64	0.64	0.12	0.21	-0.67	0.5	-1	4	728.88	no data	0.60	0.25
206	EYGLFQ	6	0	0.66	0.66	-0.35	0.42	-0.57	0.67	-1	4	755.91	no data	0.63	0.42
207	YGLFQI	6	0.23	0.67	0.67	0.98	0.21	-1.37	0.5	0	5.88	739.96	no data	0.67	0.65
208	GLFQIS	6	0.18	0.64	0.64	1.07	0.21	-0.93	0.5	0	5.88	663.86	no data	0.67	0.95
209	LFQISN	6	0.05	0.65	0.65	0.55	0.21	-0.9	0.83	0	5.88	720.91	no data	0.64	0.57
210	FQISNK	6	-0.23	0.67	0.67	-0.73	0.82	-0.1	1.17	1	9.11	735.92	no data	0.52	0.37
211	QISNKL	6	-0.24	0.65	0.65	-0.57	0.82	0.02	1.17	1	9.11	701.91	no data	0.73	0.61
212	ISNKLW	6	-0.06	0.62	0.62	-0.13	0.61	-0.58	1	1	9.11	759.99	no data	0.72	0.82
213	SNKLWC	6	-0.18	0.6	0.6	-0.47	0.61	-0.45	1	1	8.57	749.96	no data	0.70	0.96
214	NKLWCK	6	-0.32	0.63	0.63	-0.98	1.22	0	1.17	2	9.36	791.06	no data	0.61	0.95
215	KLWCKS	6	-0.25	0.59	0.59	-0.53	1.22	0.02	1	2	9.36	764.03	no data	0.65	0.98
216	LWCKSS	6	-0.11	0.57	0.57	-0.02	0.61	-0.43	0.83	1	8.57	722.93	no data	0.45	0.89
217	WCKSSQ	6	-0.32	0.59	0.59	-1.23	0.82	-0.1	1.17	1	8.57	737.9	no data	0.44	0.92
218	CKSSQV	6	-0.29	0.62	0.62	-0.38	0.82	0.22	1	1	8.57	650.82	no data	0.44	0.50
219	KSSQVP	6	-0.31	0.58	0.58	-1.07	0.82	0.38	1	1	9.11	644.8	no data	0.69	0.22
220	SSQVPQ	6	-0.24	0.58	0.58	-1	0.42	-0.08	1	0	5.88	644.76	no data	0.55	0.59
221	SQVPQS	6	-0.24	0.58	0.58	-1	0.42	-0.08	1	0	5.88	644.76	no data	0.54	0.51
222	QVPQSR	6	-0.49	0.61	0.61	-1.62	0.83	0.37	1.5	1	10.11	713.87	no data	0.44	0.11
223	VPQSRN	6	-0.48	0.62	0.62	-1.62	0.62	0.37	1.5	1	10.11	699.84	no data	0.41	0.24
224	PQSRNI	6	-0.45	0.62	0.62	-1.57	0.62	0.32	1.5	1	10.11	713.87	no data	0.32	0.29
225	QSRNIC	6	-0.43	0.66	0.66	-0.88	0.62	0.15	1.5	1	8.6	719.89	no data	0.45	0.82
226	SRNICD	6	-0.44	0.67	0.67	-0.88	0.41	0.62	1.33	0	6.16	706.84	no data	0.53	0.78
227	RNICDI	6	-0.27	0.7	0.7	0	0.41	0.27	1.17	0	6.16	732.93	no data	0.55	0.83
228	NICDIS	6	-0.02	0.68	0.68	0.62	0	-0.18	0.67	-1	3.8	663.82	no data	0.53	0.90
229	ICDISC	6	0.09	0.66	0.66	1.62	0	-0.38	0.33	-1	3.8	652.85	no data	0.59	0.98
230	CDISCD	6	-0.15	0.67	0.67	0.28	0	0.42	0.5	-2	3.57	654.77	no data	0.56	0.67
231	DISCDK	6	-0.34	0.67	0.67	-0.78	0.61	1.08	0.83	-1	4.21	679.81	no data	0.43	0.54
232	ISCDKF	6	-0.12	0.67	0.67	0.27	0.61	0.17	0.67	0	6.16	711.9	no data	0.49	0.86
233	SCDKFL	6	-0.15	0.64	0.64	0.15	0.61	0.17	0.67	0	6.16	711.9	no data	0.69	0.96
234	CDKFLD	6	-0.23	0.67	0.67	-0.3	0.61	0.62	0.67	-1	4.21	739.91	no data	0.48	0.89
235	DKFLDD	6	-0.35	0.7	0.7	-1.3	0.61	1.28	0.83	-2	3.94	751.86	no data	0.43	0.83
236	KFLDDD	6	-0.35	0.7	0.7	-1.3	0.61	1.28	0.83	-2	3.94	751.86	no data	0.53	0.72
237	FLDDDI	6	-0.05	0.7	0.7	0.1	0	0.48	0.5	-3	3.43	736.85	no data	0.50	0.77
238	LDDDIT	6	-0.18	0.67	0.67	-0.48	0	0.83	0.67	-3	3.43	690.78	no data	0.32	0.09
239	DDDITD	6	-0.39	0.71	0.71	-1.7	0	1.63	0.83	-4	3.33	692.7	no data	0.46	0.71
240	DDITDD	6	-0.39	0.71	0.71	-1.7	0	1.63	0.83	-4	3.33	692.7	no data	0.46	0.81
241	DITDDI	6	-0.15	0.7	0.7	-0.37	0	0.83	0.67	-3	3.43	690.78	no data	0.46	0.42
242	ITDDIM	6	0.02	0.71	0.71	0.53	0	0.12	0.5	-2	3.57	706.89	no data	0.42	0.23
243	TDDIMC	6	-0.1	0.69	0.69	0.2	0	0.25	0.5	-2	3.57	696.86	no data	0.45	0.15
244	DDIMCA	6	-0.03	0.69	0.69	0.62	0	0.23	0.33	-2	3.57	666.83	no data	0.56	0.27
245	DIMCAK	6	-0.09	0.68	0.68	0.55	0.61	0.23	0.5	0	6.16	679.92	no data	0.77	0.82
246	IMCAKK	6	-0.15	0.66	0.66	0.48	1.22	0.23	0.67	2	9.36	693.01	no data	0.90	0.98
247	MCAKKI	6	-0.15	0.66	0.66	0.48	1.22	0.23	0.67	2	9.36	693.01	no data	0.95	0.98
248	CAKKIL	6	-0.11	0.62	0.62	0.8	1.22	0.15	0.67	2	9.36	674.98	no data	0.92	0.98
249	AKKILD	6	-0.24	0.65	0.65	-0.2	1.22	0.82	0.83	1	8.94	686.93	no data	0.78	0.95
250	KKILDI	6	-0.16	0.67	0.67	0.25	1.22	0.6	0.83	1	8.94	729.02	no data	0.69	0.86
251	KILDIK	6	-0.16	0.67	0.67	0.25	1.22	0.6	0.83	1	8.94	729.02	no data	0.65	0.84
252	ILDIKG	6	0.05	0.67	0.67	0.83	0.61	0.1	0.5	0	6.19	657.9	no data	0.38	0.71
253	LDIKGI	6	0.05	0.67	0.67	0.83	0.61	0.1	0.5	0	6.19	657.9	no data	0.34	0.56
254	DIKGID	6	-0.15	0.71	0.71	-0.38	0.61	0.9	0.67	-1	4.21	659.82	no data	0.40	0.58

255	IKGIDY	6	-0.03	0.7	0.7	-0.02	0.61	0.02	0.67	0	6.18	707.91	no data	0.37	0.50
256	KGIDYW	6	-0.09	0.67	0.67	-0.92	0.61	-0.25	0.83	0	6.18	780.96	no data	0.50	0.33
257	GIDYWL	6	0.18	0.65	0.65	0.37	0	-1.05	0.5	-1	3.8	765.95	no data	0.51	0.66
258	IDYWLA	6	0.2	0.62	0.62	0.73	0	-1.13	0.5	-1	3.8	779.97	no data	0.71	0.90
259	DYWLAH	6	0.01	0.5	0.5	-0.55	0.24	-0.92	0.67	-0.5	5.09	803.95	no data	0.73	0.98
260	YWLAHK	6	-0.06	0.49	0.49	-0.62	0.85	-0.92	0.83	1.5	8.94	817.04	no data	0.73	0.98
261	WLAHKA	6	-0.02	0.46	0.46	-0.1	0.85	-0.62	0.67	1.5	9.11	724.94	no data	0.75	0.98
262	LAHKAL	6	0.01	0.46	0.46	0.68	0.85	-0.35	0.5	1.5	9.11	651.89	no data	0.91	0.96
263	AHKALC	6	-0.07	0.48	0.48	0.47	0.85	-0.22	0.5	1.5	8.57	641.86	no data	0.77	0.96
264	HKALCT	6	-0.14	0.48	0.48	0.05	0.85	-0.2	0.67	1.5	8.57	671.89	no data	0.77	0.91
265	KALCTE	6	-0.18	0.59	0.59	0	0.82	0.38	0.67	0	6.32	663.86	no data	0.75	0.97
266	ALCTEK	6	-0.18	0.59	0.59	0	0.82	0.38	0.67	0	6.32	663.86	no data	0.44	0.92
267	LCTEKL	6	-0.13	0.6	0.6	0.33	0.82	0.17	0.67	0	6.32	705.95	no data	0.37	0.76
268	CTEKLE	6	-0.33	0.62	0.62	-0.88	1.03	0.97	0.83	-1	4.54	721.9	no data	0.48	0.24
269	TEKLEQ	6	-0.45	0.63	0.63	-1.88	1.24	1.17	1.17	-1	4.54	746.9	no data	0.41	0.29
270	EKLEQW	6	-0.35	0.63	0.63	-1.92	1.24	0.67	1.17	-1	4.54	832.01	no data	0.44	0.26
271	KLEQWL	6	-0.16	0.6	0.6	-0.7	1.03	-0.13	1	0	6.35	816.06	no data	0.32	0.88
272	LEQWLC	6	0.03	0.59	0.59	0.37	0.42	-0.8	0.67	-1	4	791.02	no data	0.10	0.88
273	EQWLCE	6	-0.17	0.62	0.62	-0.85	0.63	0	0.83	-2	3.8	806.97	no data	0.10	0.81
274	QWLCEK	6	-0.24	0.62	0.62	-0.92	1.03	0	1	0	6.32	806.03	no data	0.10	0.61
275	WLCEKL	6	-0.04	0.59	0.59	0.3	0.82	-0.33	0.67	0	6.32	791.06	no data	0.22	0.98
276	MRFFVPL	7	0.1	0.64	0.64	1.34	0.35	-0.94	0.57	1	10.11	909.25	0.99	0.70	0.93
277	RFFVPLF	7	0.15	0.62	0.62	1.47	0.35	-1.11	0.57	1	10.11	925.23	0.99	0.82	0.94
278	FFVPLFL	7	0.48	0.6	0.6	2.66	0	-1.8	0	0	5.88	882.21	0.86	0.76	0.98
279	FVPLFLV	7	0.47	0.6	0.6	2.86	0	-1.66	0	0	5.88	834.17	0.86	0.73	0.98
280	VPLFLVG	7	0.41	0.6	0.6	2.4	0	-1.3	0	0	5.88	744.05	0.99	0.67	0.98
281	PLFLVGI	7	0.43	0.6	0.6	2.44	0	-1.34	0	0	5.88	758.08	0.99	0.70	0.93
282	LFLVGIL	7	0.52	0.62	0.62	3.21	0	-1.6	0	0	5.88	774.13	0.97	0.64	0.98
283	FLVGILF	7	0.53	0.65	0.65	3.07	0	-1.7	0	0	5.88	808.14	0.86	0.64	0.98
284	LVGILFP	7	0.43	0.6	0.6	2.44	0	-1.34	0	0	5.88	758.08	0.99	0.49	0.90
285	VGILFPA	7	0.39	0.6	0.6	2.16	0	-1.16	0	0	5.88	715.99	0.99	0.66	0.98
286	GILFPAI	7	0.42	0.6	0.6	2.2	0	-1.2	0	0	5.88	730.02	0.98	0.57	0.98
287	ILFPAIL	7	0.47	0.58	0.58	2.8	0	-1.46	0	0	5.88	786.13	0.99	0.62	0.96
288	LFPAILA	7	0.4	0.55	0.55	2.41	0	-1.27	0	0	5.88	744.04	0.99	0.72	0.89
289	FPAILAK	7	0.17	0.57	0.57	1.31	0.52	-0.59	0.29	1	9.11	759.05	0.86	0.79	0.98
290	PAILAKQ	7	-0.01	0.57	0.57	0.41	0.7	-0.2	0.57	1	9.11	740.01	0.99	0.74	0.98
291	AILAKQF	7	0.08	0.62	0.62	1.04	0.7	-0.56	0.57	1	9.11	790.07	0.61	0.69	0.98
292	ILAKQFT	7	0.02	0.62	0.62	0.69	0.7	-0.54	0.71	1	9.11	820.1	0.99	0.84	0.91
293	LAKQFTK	7	-0.24	0.62	0.62	-0.51	1.23	0.14	1	2	10.02	835.11	0.99	0.85	0.93
294	AKQFTKC	7	-0.31	0.63	0.63	-0.7	1.23	0.26	1	2	9.36	825.08	0.61	0.66	0.97
295	KQFTKCE	7	-0.43	0.65	0.65	-1.46	1.41	0.76	1.14	1	8.54	883.12	0.99	0.40	0.69
296	QFTKCEL	7	-0.2	0.63	0.63	-0.36	0.88	0.07	0.86	0	6.32	868.11	0.94	0.39	0.29
297	FTKCELS	7	-0.14	0.61	0.61	0.03	0.71	0.09	0.71	0	6.32	827.05	0.61	0.36	0.57
298	TKCELSQ	7	-0.33	0.61	0.61	-0.87	0.88	0.47	1	0	6.32	808.01	0.99	0.43	0.53
299	KCELSQL	7	-0.22	0.61	0.61	-0.23	0.88	0.27	0.86	0	6.32	820.07	0.99	0.23	0.48
300	CELSQLL	7	0.01	0.59	0.59	0.87	0.36	-0.41	0.57	-1	4	805.06	0.61	0.30	0.97
301	ELSQLLK	7	-0.15	0.59	0.59	-0.04	0.88	0.16	0.86	0	6.35	830.1	0.61	0.26	0.70
302	LSQLLKD	7	-0.17	0.61	0.61	-0.04	0.7	0.16	0.86	0	6.19	816.07	0.99	0.27	0.77
303	SQLLKDI	7	-0.14	0.63	0.63	0.06	0.7	0.16	0.86	0	6.19	816.07	0.99	0.29	0.60
304	QLLKDID	7	-0.21	0.66	0.66	-0.33	0.7	0.54	0.86	-1	4.21	844.08	0.94	0.33	0.59
305	LLKDIDG	7	-0.08	0.66	0.66	0.11	0.52	0.51	0.57	-1	4.21	773	0.99	0.48	0.38
306	LKDIDGY	7	-0.16	0.69	0.69	-0.61	0.52	0.44	0.71	-1	4.21	823.01	0.99	0.41	0.07
307	KDIDGYG	7	-0.21	0.71	0.71	-1.21	0.52	0.7	0.71	-1	4.21	766.9	0.99	0.44	0.04
308	DIDGYGG	7	-0.03	0.71	0.71	-0.71	0	0.27	0.43	-2	3.57	695.78	0.61	0.54	0.48
309	IDGYGGI	7	0.18	0.7	0.7	0.43	0	-0.41	0.29	-1	3.8	693.86	0.99	0.55	0.68
310	DGYGGIA	7	0.11	0.67	0.67	0.04	0	-0.23	0.29	-1	3.8	651.77	0.61	0.59	0.60
311	GYGGIAL	7	0.29	0.64	0.64	1.09	0	-0.91	0.14	0	5.88	649.85	0.98	0.72	0.97
312	YGGIALP	7	0.25	0.6	0.6	0.91	0	-0.91	0.14	0	5.88	689.91	0.99	0.72	0.94
313	GGIALPE	7	0.16	0.59	0.59	0.6	0.18	-0.16	0.14	-1	4	655.85	0.98	0.73	0.93
314	GIALPEL	7	0.22	0.57	0.57	1.2	0.18	-0.41	0.14	-1	4	711.96	0.98	0.69	0.77
315	IALPELI	7	0.3	0.57	0.57	1.9	0.18	-0.67	0.14	-1	4	768.07	0.99	0.67	0.78
316	ALPELIC	7	0.2	0.56	0.56	1.61	0.18	-0.56	0.14	-1	4	758.04	0.61	0.69	0.81
317	LPELICT	7	0.14	0.56	0.56	1.26	0.18	-0.54	0.29	-1	4	788.07	0.99	0.57	0.53
318	PELICTM	7	0.1	0.6	0.6	0.99	0.18	-0.47	0.29	-1	4	806.1	0.94	0.53	0.58

319	ELICTMF	7	0.2	0.65	0.65	1.61	0.18	-0.83	0.29	-1	4	856.16	0.61	0.50	0.26
320	LICTMFH	7	0.23	0.55	0.55	1.66	0.21	-1.33	0.29	0.5	7.06	864.19	0.99	0.62	0.95
321	ICTMFHT	7	0.13	0.55	0.55	1.01	0.21	-1.13	0.43	0.5	7.06	852.13	0.99	0.64	0.97
322	CTMFHTS	7	-0.02	0.53	0.53	0.26	0.21	-0.83	0.57	0.5	7.06	826.04	0.61	0.58	0.94
323	TMFHTSG	7	0	0.54	0.54	-0.16	0.21	-0.69	0.57	0.5	7.1	779.96	0.99	0.40	0.77
324	MFHTSGY	7	0.03	0.56	0.56	-0.24	0.21	-0.96	0.57	0.5	7.09	842.03	0.99	0.34	0.20
325	FHTSGYD	7	-0.11	0.56	0.56	-1.01	0.21	-0.34	0.71	-0.5	5.09	825.92	0.61	0.33	0.24
326	HTSGYDT	7	-0.22	0.53	0.53	-1.51	0.21	-0.04	0.86	-0.5	5.09	779.85	0.99	0.26	0.21
327	TSGYDTQ	7	-0.26	0.63	0.63	-1.56	0.18	0.06	1	-1	3.8	770.84	0.99	0.24	0.18
328	SGYDTQA	7	-0.2	0.63	0.63	-1.2	0.18	0.04	0.86	-1	3.8	740.81	0.99	0.28	0.14
329	GYDTQAI	7	-0.06	0.65	0.65	-0.44	0.18	-0.26	0.71	-1	3.8	766.9	0.98	0.35	0.12
330	YDTQAIV	7	-0.01	0.66	0.66	0.21	0.18	-0.47	0.71	-1	3.8	808.98	0.99	0.44	0.22
331	DTQAIVE	7	-0.1	0.65	0.65	-0.1	0.36	0.29	0.71	-2	3.67	774.92	0.61	0.52	0.15
332	TQAIVEN	7	-0.09	0.65	0.65	-0.1	0.36	-0.11	0.86	-1	4	773.94	0.99	0.57	0.43
333	QAIVENN	7	-0.15	0.69	0.69	-0.5	0.36	-0.03	1	-1	4	786.94	0.99	0.52	0.32
334	AIVENNE	7	-0.14	0.69	0.69	-0.5	0.36	0.37	0.86	-2	3.8	787.92	0.61	0.44	0.29
335	IVENNES	7	-0.22	0.69	0.69	-0.87	0.36	0.49	1	-2	3.8	803.92	0.94	0.52	0.13
336	VENNEST	7	-0.35	0.66	0.66	-1.61	0.36	0.69	1.14	-2	3.8	791.86	0.99	0.39	0.14
337	ENNESTE	7	-0.51	0.66	0.66	-2.71	0.54	1.33	1.29	-3	3.68	821.84	0.61	0.30	0.36
338	NNESTEY	7	-0.42	0.66	0.66	-2.4	0.36	0.57	1.29	-2	3.8	855.9	0.94	0.28	0.29
339	NESTEYG	7	-0.31	0.65	0.65	-1.96	0.36	0.54	1	-2	3.8	798.85	0.99	0.32	0.07
340	ESTEYGL	7	-0.14	0.62	0.62	-0.91	0.36	0.26	0.71	-2	3.8	797.91	0.61	0.42	0.17
341	STEYGLF	7	0.04	0.62	0.62	-0.01	0.18	-0.53	0.57	-1	4	815.97	0.99	0.54	0.23
342	TEYGLFQ	7	-0.02	0.64	0.64	-0.4	0.36	-0.54	0.71	-1	4	857.03	0.99	0.55	0.25
343	EYGLFQI	7	0.11	0.67	0.67	0.34	0.36	-0.74	0.57	-1	4	869.09	0.61	0.65	0.29
344	YGLFQIS	7	0.16	0.65	0.65	0.73	0.18	-1.13	0.57	0	5.88	827.05	0.99	0.66	0.78
345	GLFQISN	7	0.06	0.65	0.65	0.41	0.18	-0.77	0.71	0	5.88	777.98	0.98	0.62	0.79
346	LFQISNK	7	-0.12	0.65	0.65	-0.09	0.7	-0.34	1	1	9.11	849.1	0.94	0.63	0.70
347	FQISNKL	7	-0.12	0.65	0.65	-0.09	0.7	-0.34	1	1	9.11	849.1	0.61	0.67	0.87
348	QISNKLW	7	-0.15	0.63	0.63	-0.61	0.7	-0.47	1.14	1	9.11	888.14	0.99	0.72	0.85
349	ISNKLWC	7	-0.05	0.62	0.62	0.24	0.52	-0.64	0.86	1	8.57	863.14	0.99	0.72	0.95
350	SNKLWCK	7	-0.31	0.61	0.61	-0.96	1.05	0.04	1.14	2	9.36	878.15	0.99	0.59	0.94
351	NKLWCKS	7	-0.31	0.61	0.61	-0.96	1.05	0.04	1.14	2	9.36	878.15	0.99	0.61	0.91
352	KLWCKSS	7	-0.25	0.58	0.58	-0.57	1.05	0.06	1	2	9.36	851.12	0.99	0.62	0.97
353	LWCKSSQ	7	-0.2	0.58	0.58	-0.51	0.7	-0.34	1	1	8.57	851.08	0.99	0.49	0.91
354	WCKSSQV	7	-0.19	0.61	0.61	-0.46	0.7	-0.3	1	1	8.57	837.05	0.99	0.49	0.85
355	CKSSQVP	7	-0.26	0.59	0.59	-0.56	0.7	0.19	0.86	1	8.57	747.95	0.61	0.48	0.54
356	KSSQVPQ	7	-0.36	0.59	0.59	-1.41	0.88	0.36	1.14	1	9.11	772.95	0.99	0.60	0.16
357	SSQVPQS	7	-0.24	0.57	0.57	-0.97	0.36	-0.03	1	0	5.88	731.85	0.99	0.53	0.45
358	SQVPQSR	7	-0.46	0.59	0.59	-1.5	0.71	0.36	1.43	1	10.11	800.96	0.99	0.46	0.07
359	QVPQSRN	7	-0.51	0.63	0.63	-1.89	0.71	0.34	1.57	1	10.11	827.99	0.99	0.44	0.13
360	VPQSRNI	7	-0.31	0.63	0.63	-0.74	0.53	0.06	1.29	1	10.11	813.02	0.99	0.38	0.16
361	PQSRNIC	7	-0.38	0.62	0.62	-0.99	0.53	0.13	1.29	1	8.6	817.02	0.94	0.34	0.81
362	QSRNICD	7	-0.47	0.68	0.68	-1.26	0.53	0.56	1.43	0	6.16	834.99	0.99	0.48	0.24
363	SRNICDI	7	-0.27	0.68	0.68	-0.11	0.35	0.27	1.14	0	6.16	820.02	0.99	0.52	0.73
364	RNICDIS	7	-0.27	0.68	0.68	-0.11	0.35	0.27	1.14	0	6.16	820.02	0.99	0.56	0.87
365	NICDISC	7	-0.01	0.67	0.67	0.89	0	-0.3	0.57	-1	3.8	766.97	0.99	0.54	0.97
366	ICDISCD	7	-0.02	0.67	0.67	0.89	0	0.1	0.43	-2	3.57	767.95	0.99	0.59	0.86
367	CDISCDK	7	-0.28	0.67	0.67	-0.31	0.52	0.79	0.71	-1	4.21	782.96	0.61	0.43	0.67
368	DISCDKF	7	-0.2	0.68	0.68	-0.27	0.52	0.57	0.71	-1	4.21	827	0.61	0.49	0.46
369	ISCDKFL	7	-0.02	0.65	0.65	0.77	0.52	-0.11	0.57	0	6.16	825.08	0.99	0.67	0.87
370	SCDKFLD	7	-0.23	0.65	0.65	-0.37	0.52	0.57	0.71	-1	4.21	827	0.99	0.47	0.88
371	CDKFLDD	7	-0.3	0.69	0.69	-0.76	0.52	0.96	0.71	-2	3.94	855.01	0.61	0.44	0.63
372	DKFLDDD	7	-0.41	0.71	0.71	-1.61	0.52	1.53	0.86	-3	3.77	866.96	0.61	0.43	0.75
373	KFLDDDI	7	-0.2	0.7	0.7	-0.47	0.52	0.84	0.71	-2	3.94	865.04	0.99	0.53	0.46
374	FLDDDIT	7	-0.07	0.68	0.68	-0.01	0	0.36	0.57	-3	3.43	837.97	0.61	0.51	0.06
375	LDDDITD	7	-0.26	0.69	0.69	-0.91	0	1.14	0.71	-4	3.33	805.88	0.99	0.28	0.16
376	DDDITDD	7	-0.44	0.72	0.72	-1.96	0	1.83	0.86	-5	3.25	807.8	0.61	0.46	0.77
377	DDITDDI	7	-0.23	0.71	0.71	-0.81	0	1.14	0.71	-4	3.33	805.88	0.61	0.46	0.40
378	DITDDIM	7	-0.09	0.71	0.71	-0.04	0	0.53	0.57	-3	3.43	821.99	0.61	0.42	0.13
379	ITDDIMC	7	0.02	0.69	0.69	0.81	0	-0.04	0.43	-2	3.57	810.04	0.99	0.48	0.19
380	TDDIMCA	7	-0.05	0.67	0.67	0.43	0	0.14	0.43	-2	3.57	767.95	0.99	0.49	0.07
381	DDIMCAK	7	-0.18	0.69	0.69	-0.03	0.52	0.63	0.57	-1	4.21	795.02	0.61	0.75	0.17
382	DIMCAKK	7	-0.23	0.68	0.68	-0.09	1.05	0.63	0.71	1	8.54	808.11	0.61	0.86	0.96

383	IMCAKKI	7	-0.03	0.67	0.67	1.06	1.05	-0.06	0.57	2	9.36	806.19	0.99	0.89	0.98
384	MCAKKIL	7	-0.06	0.64	0.64	0.96	1.05	-0.06	0.57	2	9.36	806.19	0.99	0.91	0.98
385	CAKKILD	7	-0.2	0.64	0.64	0.19	1.05	0.56	0.71	1	8.54	790.08	0.83	0.78	0.97
386	AKKILDI	7	-0.1	0.65	0.65	0.47	1.05	0.44	0.71	1	8.94	800.11	0.61	0.77	0.86
387	KKILDIK	7	-0.29	0.68	0.68	-0.34	1.57	0.94	1	2	9.72	857.21	0.99	0.68	0.88
388	KILDIKG	7	-0.11	0.68	0.68	0.16	1.05	0.51	0.71	1	8.94	786.09	0.99	0.49	0.80
389	ILDIKGI	7	0.15	0.68	0.68	1.36	0.52	-0.17	0.43	0	6.19	771.08	0.99	0.33	0.65
390	LDIKGID	7	-0.06	0.69	0.69	0.21	0.52	0.51	0.57	-1	4.21	773	0.99	0.29	0.45
391	DIKGIDY	7	-0.13	0.71	0.71	-0.51	0.52	0.44	0.71	-1	4.21	823.01	0.61	0.38	0.25
392	IKGIDYW	7	0.03	0.67	0.67	-0.14	0.52	-0.47	0.71	0	6.18	894.14	0.99	0.53	0.47
393	KGIDYWL	7	0	0.65	0.65	-0.24	0.52	-0.47	0.71	0	6.18	894.14	0.99	0.42	0.47
394	GIDYWLA	7	0.19	0.63	0.63	0.57	0	-0.97	0.43	-1	3.8	837.04	0.98	0.71	0.75
395	IDYWLAH	7	0.11	0.53	0.53	0.17	0.21	-1.04	0.57	-0.5	5.09	917.13	0.99	0.72	0.84
396	DYWLAHK	7	-0.15	0.53	0.53	-1.03	0.73	-0.36	0.86	0.5	7.09	932.14	0.61	0.74	0.98
397	YWLAHKA	7	-0.01	0.49	0.49	-0.27	0.73	-0.86	0.71	1.5	8.94	888.13	0.99	0.86	0.98
398	WLAHKAL	7	0.06	0.47	0.47	0.46	0.73	-0.79	0.57	1.5	9.11	838.12	0.99	0.83	0.98
399	LAHKALC	7	0.01	0.49	0.49	0.94	0.73	-0.44	0.43	1.5	8.57	755.04	0.99	0.85	0.92
400	AHKALCT	7	-0.09	0.49	0.49	0.3	0.73	-0.24	0.57	1.5	8.57	742.98	0.61	0.75	0.93
401	HKALCTE	7	-0.21	0.51	0.51	-0.46	0.91	0.26	0.71	0.5	7.06	801.02	0.99	0.72	0.94
402	KALCTEK	7	-0.31	0.61	0.61	-0.56	1.23	0.76	0.86	1	8.54	792.05	0.99	0.69	0.82
403	ALCTEKL	7	-0.08	0.58	0.58	0.54	0.71	0.07	0.57	0	6.32	777.04	0.61	0.50	0.93
404	LCTEKLE	7	-0.2	0.61	0.61	-0.21	0.89	0.57	0.71	-1	4.54	835.08	0.99	0.35	0.52
405	CTEKLEQ	7	-0.38	0.63	0.63	-1.26	1.07	0.86	1	-1	4.54	850.05	0.86	0.40	0.25
406	TEKLEQW	7	-0.33	0.61	0.61	-1.74	1.07	0.51	1.14	-1	4.54	933.13	0.99	0.36	0.30
407	EKLEQWL	7	-0.23	0.61	0.61	-1.1	1.07	0.31	1	-1	4.54	945.19	0.61	0.25	0.39
408	KLEQWLC	7	-0.13	0.6	0.6	-0.24	0.88	-0.26	0.86	0	6.32	919.21	0.99	0.20	0.96
409	LEQWLCE	7	-0.07	0.6	0.6	-0.19	0.54	-0.26	0.71	-2	3.8	920.15	0.99	0.10	0.81
410	EQWLCEK	7	-0.3	0.62	0.62	-1.29	1.07	0.43	1	-1	4.54	935.16	0.61	0.08	0.19
411	QWLCEKL	7	-0.13	0.6	0.6	-0.24	0.88	-0.26	0.86	0	6.32	919.21	0.99	0.16	0.96
412	MRFFVPLF	8	0.17	0.64	0.64	1.52	0.31	-1.14	0.5	1	10.11	1056.44	0.99	0.79	0.97
413	RFFVPLFL	8	0.2	0.61	0.61	1.76	0.31	-1.2	0.5	1	10.11	1038.41	0.99	0.76	0.94
414	FFVPLFLV	8	0.49	0.61	0.61	2.85	0	-1.76	0	0	5.88	981.36	0.86	0.75	0.95
415	FVPLFLVG	8	0.43	0.61	0.61	2.45	0	-1.45	0	0	5.88	891.24	0.86	0.72	0.98
416	VPLFLVGI	8	0.45	0.61	0.61	2.66	0	-1.36	0	0	5.88	857.23	0.99	0.67	0.87
417	PLFLVGIL	8	0.45	0.59	0.59	2.61	0	-1.4	0	0	5.88	871.26	0.99	0.72	0.98
418	LFLVGILF	8	0.53	0.63	0.63	3.16	0	-1.71	0	0	5.88	921.32	0.97	0.64	0.80
419	FLVGILFP	8	0.46	0.61	0.61	2.49	0	-1.49	0	0	5.88	905.27	0.86	0.64	0.98
420	LVGILFPA	8	0.41	0.59	0.59	2.36	0	-1.24	0	0	5.88	829.17	0.99	0.55	0.80
421	VGILFPAI	8	0.44	0.61	0.61	2.45	0	-1.24	0	0	5.88	829.17	0.99	0.53	0.98
422	GILFPAIL	8	0.43	0.59	0.59	2.4	0	-1.28	0	0	5.88	843.2	0.98	0.57	0.98
423	ILFPAILA	8	0.44	0.57	0.57	2.68	0	-1.34	0	0	5.88	857.22	0.99	0.72	0.96
424	LFPAILAK	8	0.22	0.57	0.57	1.63	0.46	-0.74	0.25	1	9.11	872.23	0.99	0.81	0.98
425	FPAILAKQ	8	0.06	0.59	0.59	0.71	0.61	-0.49	0.5	1	9.11	887.2	0.86	0.72	0.98
426	PAILAKQF	8	0.06	0.59	0.59	0.71	0.61	-0.49	0.5	1	9.11	887.2	0.99	0.71	0.98
427	AILAKQFT	8	0.05	0.61	0.61	0.82	0.61	-0.54	0.62	1	9.11	891.19	0.61	0.68	0.95
428	ILAKQFTK	8	-0.12	0.63	0.63	0.11	1.07	-0.1	0.88	2	10.02	948.29	0.99	0.83	0.89
429	LAKQFTKC	8	-0.21	0.62	0.62	-0.14	1.07	0	0.88	2	9.36	938.26	0.99	0.80	0.93
430	AKQFTKCE	8	-0.35	0.64	0.64	-1.05	1.23	0.6	1	1	8.54	954.21	0.61	0.66	0.69
431	KQFTKCEL	8	-0.31	0.64	0.64	-0.8	1.23	0.44	1	1	8.54	996.3	0.99	0.41	0.39
432	QFTKCELS	8	-0.21	0.62	0.62	-0.41	0.77	0.1	0.88	0	6.32	955.2	0.94	0.36	0.18
433	FTKCELSQ	8	-0.21	0.62	0.62	-0.41	0.77	0.1	0.88	0	6.32	955.2	0.61	0.39	0.54
434	TKCELSQL	8	-0.22	0.6	0.6	-0.29	0.77	0.19	0.88	0	6.32	921.19	0.99	0.30	0.33
435	KCELSQLL	8	-0.13	0.6	0.6	0.27	0.77	0.01	0.75	0	6.32	933.25	0.99	0.27	0.89
436	CELSQLLK	8	-0.13	0.6	0.6	0.27	0.77	0.01	0.75	0	6.32	933.25	0.61	0.24	0.66
437	ELSQLLKD	8	-0.23	0.61	0.61	-0.48	0.77	0.51	0.88	-1	4.38	945.2	0.61	0.22	0.56
438	LSQLLKDI	8	-0.06	0.62	0.62	0.52	0.61	-0.09	0.75	0	6.19	929.25	0.99	0.27	0.69
439	SQLLKDID	8	-0.21	0.65	0.65	-0.39	0.61	0.51	0.88	-1	4.21	931.17	0.99	0.29	0.48
440	QLLKDIDG	8	-0.16	0.66	0.66	-0.34	0.61	0.47	0.75	-1	4.21	901.15	0.94	0.31	0.15
441	LLKDIDGY	8	-0.07	0.67	0.67	-0.06	0.46	0.16	0.62	-1	4.21	936.19	0.99	0.43	0.14
442	LKDIDGYG	8	-0.12	0.69	0.69	-0.59	0.46	0.39	0.62	-1	4.21	880.08	0.99	0.41	0.04
443	KDIDGYGG	8	-0.16	0.7	0.7	-1.11	0.46	0.61	0.62	-1	4.21	823.97	0.99	0.42	0.06
444	DIDGYGGI	8	0.07	0.71	0.71	-0.06	0	0.01	0.38	-2	3.57	808.96	0.61	0.49	0.24
445	IDGYGGIA	8	0.19	0.68	0.68	0.6	0	-0.42	0.25	-1	3.8	764.95	0.99	0.58	0.54
446	DGYGGIAL	8	0.16	0.66	0.66	0.51	0	-0.42	0.25	-1	3.8	764.95	0.61	0.68	0.53

447	GYGGIALP	8	0.24	0.61	0.61	0.75	0	-0.8	0.12	0	5.88	746.98	0.98	0.70	0.95
448	YGGIALPE	8	0.14	0.61	0.61	0.36	0.16	-0.42	0.25	-1	4	819.04	0.99	0.71	0.59
449	GGIALPEL	8	0.21	0.59	0.59	1	0.16	-0.36	0.12	-1	4	769.03	0.98	0.66	0.91
450	GIALPELI	8	0.28	0.59	0.59	1.61	0.16	-0.59	0.12	-1	4	825.14	0.98	0.68	0.84
451	IALPELIC	8	0.27	0.58	0.58	1.98	0.16	-0.71	0.12	-1	4	871.22	0.99	0.66	0.86
452	ALPELICT	8	0.15	0.56	0.56	1.32	0.16	-0.54	0.25	-1	4	859.16	0.61	0.65	0.55
453	LPELICTM	8	0.15	0.59	0.59	1.34	0.16	-0.64	0.25	-1	4	919.28	0.99	0.60	0.59
454	PELICTMF	8	0.16	0.61	0.61	1.21	0.16	-0.72	0.25	-1	4	953.29	0.94	0.50	0.14
455	ELICTMFH	8	0.12	0.57	0.57	1.01	0.34	-0.79	0.38	-0.5	5.25	993.32	0.61	0.55	0.17
456	LICTMFHT	8	0.18	0.55	0.55	1.36	0.18	-1.21	0.38	0.5	7.06	965.31	0.99	0.63	0.95
457	ICTMFHTS	8	0.08	0.55	0.55	0.79	0.18	-0.95	0.5	0.5	7.06	939.22	0.99	0.58	0.98
458	CTMFHTSG	8	0.01	0.55	0.55	0.17	0.18	-0.73	0.5	0.5	7.06	883.11	0.61	0.39	0.94
459	TMFHTSGY	8	0	0.56	0.56	-0.3	0.18	-0.89	0.62	0.5	7.09	943.15	0.99	0.36	0.33
460	MFHTSGYD	8	-0.06	0.58	0.58	-0.65	0.18	-0.46	0.62	-0.5	5.09	957.13	0.99	0.32	0.08
461	FHTSGYDT	8	-0.12	0.55	0.55	-0.98	0.18	-0.35	0.75	-0.5	5.09	927.04	0.61	0.31	0.13
462	HTSGYDTQ	8	-0.28	0.55	0.55	-1.76	0.34	-0.01	1	-0.5	5.09	908	0.99	0.26	0.18
463	TSGYDTQA	8	-0.2	0.62	0.62	-1.14	0.16	-0.01	0.88	-1	3.8	841.93	0.99	0.23	0.16
464	SGYDTQAI	8	-0.09	0.64	0.64	-0.49	0.16	-0.19	0.75	-1	3.8	853.99	0.99	0.21	0.11
465	GYDTQAIV	8	0.01	0.66	0.66	0.14	0.16	-0.41	0.62	-1	3.8	866.05	0.98	0.41	0.12
466	YDTQAIVE	8	-0.08	0.66	0.66	-0.25	0.32	-0.04	0.75	-2	3.67	938.11	0.99	0.51	0.08
467	DTQAIVEN	8	-0.17	0.67	0.67	-0.53	0.32	0.28	0.88	-2	3.67	889.04	0.61	0.53	0.30
468	TQAIVENN	8	-0.16	0.67	0.67	-0.53	0.32	-0.08	1	-1	4	888.06	0.99	0.54	0.36
469	QAIVENNE	8	-0.21	0.68	0.68	-0.88	0.47	0.35	1	-2	3.8	916.07	0.99	0.41	0.16
470	AIVENNES	8	-0.16	0.67	0.67	-0.54	0.32	0.36	0.88	-2	3.8	875.01	0.61	0.43	0.11
471	IVENNEST	8	-0.21	0.67	0.67	-0.85	0.32	0.38	1	-2	3.8	905.04	0.94	0.48	0.09
472	VENNESTE	8	-0.38	0.66	0.66	-1.85	0.48	0.97	1.12	-3	3.68	920.99	0.99	0.37	0.14
473	ENNESTEY	8	-0.45	0.67	0.67	-2.54	0.48	0.88	1.25	-3	3.68	985.03	0.61	0.30	0.30
474	NNESTEYG	8	-0.35	0.67	0.67	-2.15	0.32	0.5	1.12	-2	3.8	912.97	0.94	0.30	0.06
475	NESTEYGL	8	-0.2	0.64	0.64	-1.24	0.32	0.25	0.88	-2	3.8	912.03	0.99	0.30	0.12
476	ESTEYGLF	8	-0.05	0.63	0.63	-0.45	0.32	-0.09	0.62	-2	3.8	945.1	0.61	0.54	0.18
477	STEYGLFQ	8	-0.05	0.63	0.63	-0.45	0.32	-0.44	0.75	-1	4	944.12	0.99	0.50	0.26
478	TEYGLFQI	8	0.07	0.65	0.65	0.21	0.32	-0.7	0.62	-1	4	970.21	0.99	0.58	0.23
479	EYGLFQIS	8	0.06	0.65	0.65	0.2	0.32	-0.61	0.62	-1	4	956.18	0.61	0.64	0.50
480	YGLFQISN	8	0.06	0.66	0.66	0.2	0.16	-0.96	0.75	0	5.88	941.17	0.99	0.61	0.44
481	GLFQISNK	8	-0.08	0.66	0.66	-0.13	0.61	-0.3	0.88	1	9.11	906.17	0.98	0.61	0.79
482	LFQISNKL	8	-0.04	0.64	0.64	0.4	0.61	-0.52	0.88	1	9.11	962.28	0.94	0.73	0.97
483	FQISNKLW	8	-0.06	0.64	0.64	-0.19	0.61	-0.72	1	1	9.11	1035.33	0.61	0.65	0.97
484	QISNKLWC	8	-0.13	0.62	0.62	-0.22	0.61	-0.54	1	1	8.57	991.29	0.99	0.72	0.93
485	ISNKLWCK	8	-0.18	0.62	0.62	-0.27	0.92	-0.19	1	2	9.36	991.33	0.99	0.62	0.97
486	SNKLWCKS	8	-0.3	0.6	0.6	-0.94	0.92	0.07	1.12	2	9.36	965.24	0.99	0.59	0.86
487	NKLWCKSS	8	-0.3	0.6	0.6	-0.94	0.92	0.07	1.12	2	9.36	965.24	0.99	0.59	0.93
488	KLWCKSSQ	8	-0.31	0.59	0.59	-0.94	1.07	0.07	1.12	2	9.36	979.27	0.99	0.61	0.99
489	LWCKSSQV	8	-0.1	0.6	0.6	0.08	0.61	-0.49	0.88	1	8.57	950.23	0.99	0.52	0.86
490	WCKSSQVP	8	-0.18	0.58	0.58	-0.6	0.61	-0.26	0.88	1	8.57	934.18	0.99	0.51	0.87
491	CKSSQVPQ	8	-0.31	0.6	0.6	-0.93	0.77	0.19	1	1	8.57	876.1	0.61	0.45	0.31
492	KSSQVPQS	8	-0.35	0.59	0.59	-1.34	0.77	0.35	1.12	1	9.11	860.04	0.99	0.57	0.07
493	SSQVPQSR	8	-0.43	0.59	0.59	-1.41	0.62	0.35	1.38	1	10.11	888.05	0.99	0.46	0.08
494	SQVPQSRN	8	-0.48	0.61	0.61	-1.75	0.62	0.34	1.5	1	10.11	915.08	0.99	0.44	0.14
495	QVPQSRNI	8	-0.35	0.64	0.64	-1.09	0.62	0.08	1.38	1	10.11	941.17	0.99	0.41	0.11
496	VPQSRNIC	8	-0.26	0.63	0.63	-0.34	0.46	-0.07	1.12	1	8.6	916.17	0.99	0.40	0.79
497	PQSRNICD	8	-0.42	0.64	0.64	-1.3	0.46	0.49	1.25	0	6.16	932.12	0.94	0.38	0.25
498	QSRNICDI	8	-0.32	0.68	0.68	-0.54	0.46	0.26	1.25	0	6.16	948.17	0.99	0.47	0.27
499	SRNICDIS	8	-0.27	0.66	0.66	-0.2	0.31	0.28	1.12	0	6.16	907.11	0.99	0.53	0.68
500	RNICDISC	8	-0.23	0.67	0.67	0.21	0.31	0.11	1	0	6.13	923.17	0.99	0.55	0.98
501	NICDISCD	8	-0.1	0.68	0.68	0.34	0	0.11	0.62	-2	3.57	882.07	0.99	0.54	0.86
502	ICDISCDK	8	-0.16	0.67	0.67	0.29	0.46	0.46	0.62	-1	4.21	896.14	0.99	0.46	0.87
503	CDISCDKF	8	-0.17	0.67	0.67	0.08	0.46	0.38	0.62	-1	4.21	930.15	0.61	0.49	0.59
504	DISCDKFL	8	-0.11	0.66	0.66	0.24	0.46	0.28	0.62	-1	4.21	940.18	0.61	0.66	0.52
505	ISCDKFLD	8	-0.11	0.66	0.66	0.24	0.46	0.28	0.62	-1	4.21	940.18	0.99	0.43	0.59
506	SCDKFLDD	8	-0.29	0.67	0.67	-0.76	0.46	0.88	0.75	-2	3.94	942.1	0.99	0.42	0.66
507	CDKFLDDD	8	-0.35	0.7	0.7	-1.1	0.46	1.21	0.75	-3	3.77	970.11	0.61	0.43	0.43
508	DKFLDDDI	8	-0.26	0.71	0.71	-0.85	0.46	1.11	0.75	-3	3.77	980.14	0.61	0.43	0.40
509	KFLDDDIT	8	-0.2	0.68	0.68	-0.5	0.46	0.69	0.75	-2	3.94	966.16	0.99	0.52	0.10
510	FLDDDITD	8	-0.15	0.69	0.69	-0.45	0	0.69	0.62	-4	3.33	953.07	0.61	0.46	0.09

511	LDDDITDD	8	-0.31	0.69	0.69	-1.24	0	1.38	0.75	-5	3.25	920.98	0.99	0.28	0.21
512	DDDITDDI	8	-0.29	0.72	0.72	-1.15	0	1.38	0.75	-5	3.25	920.98	0.61	0.46	0.30
513	DDITDDIM	8	-0.17	0.72	0.72	-0.47	0	0.84	0.62	-4	3.33	937.09	0.61	0.42	0.14
514	DITDDIMC	8	-0.07	0.7	0.7	0.27	0	0.34	0.5	-3	3.43	925.14	0.61	0.48	0.09
515	ITDDIMCA	8	0.05	0.67	0.67	0.94	0	-0.1	0.38	-2	3.57	881.13	0.99	0.51	0.12
516	TDDIMCAK	8	-0.18	0.67	0.67	-0.11	0.46	0.5	0.62	-1	4.21	896.14	0.99	0.72	0.06
517	DDIMCAKK	8	-0.3	0.69	0.69	-0.51	0.92	0.93	0.75	0	6.28	923.21	0.61	0.83	0.80
518	DIMCAKKI	8	-0.11	0.68	0.68	0.49	0.92	0.33	0.62	1	8.54	921.29	0.61	0.87	0.78
519	IMCAKKIL	8	0.04	0.65	0.65	1.4	0.92	-0.27	0.5	2	9.36	919.37	0.99	0.87	0.98
520	MCAKKILD	8	-0.14	0.66	0.66	0.4	0.92	0.33	0.62	1	8.54	921.29	0.99	0.77	0.93
521	CAKKILDI	8	-0.08	0.65	0.65	0.72	0.92	0.26	0.62	1	8.54	903.26	0.83	0.78	0.91
522	AKKILDIK	8	-0.22	0.66	0.66	-0.08	1.38	0.76	0.88	2	9.72	928.3	0.61	0.76	0.88
523	KKILDIKG	8	-0.23	0.68	0.68	-0.35	1.38	0.83	0.88	2	9.72	914.28	0.99	0.62	0.75
524	KILDIKGI	8	-0.01	0.68	0.68	0.7	0.92	0.22	0.62	1	8.94	899.27	0.99	0.46	0.66
525	ILDIKGID	8	0.04	0.69	0.69	0.75	0.46	0.22	0.5	-1	4.21	886.18	0.99	0.30	0.60
526	LDIKGIDY	8	-0.05	0.69	0.69	0.02	0.46	0.16	0.62	-1	4.21	936.19	0.99	0.30	0.26
527	DIKGIDYW	8	-0.07	0.69	0.69	-0.56	0.46	-0.04	0.75	-1	4.21	1009.24	0.61	0.54	0.09
528	IKGIDYWL	8	0.09	0.66	0.66	0.35	0.46	-0.64	0.62	0	6.18	1007.32	0.99	0.48	0.49
529	KGIDYWLA	8	0.03	0.63	0.63	0.01	0.46	-0.47	0.62	0	6.18	965.23	0.99	0.56	0.74
530	GIDYWLAH	8	0.12	0.55	0.55	0.1	0.18	-0.91	0.5	-0.5	5.09	974.2	0.98	0.74	0.76
531	IDYWLAHK	8	-0.04	0.55	0.55	-0.34	0.64	-0.54	0.75	0.5	7.09	1045.32	0.99	0.74	0.89
532	DYWLAHKA	8	-0.1	0.53	0.53	-0.68	0.64	-0.38	0.75	0.5	7.09	1003.23	0.86	0.81	0.98
533	YWLAHKAL	8	0.06	0.5	0.5	0.24	0.64	-0.97	0.62	1.5	8.94	1001.31	0.99	0.84	0.98
534	WLAHKALC	8	0.06	0.49	0.49	0.71	0.64	-0.81	0.5	1.5	8.57	941.27	0.99	0.73	0.98
535	LAHKALCT	8	-0.01	0.49	0.49	0.74	0.64	-0.44	0.5	1.5	8.57	856.16	0.99	0.80	0.88
536	AHKALCTE	8	-0.15	0.51	0.51	-0.18	0.8	0.16	0.62	0.5	7.06	872.11	0.61	0.64	0.95
537	HKALCTEK	8	-0.32	0.53	0.53	-0.89	1.26	0.6	0.88	1.5	8.54	929.21	0.99	0.57	0.75
538	KALCTEKL	8	-0.21	0.6	0.6	-0.01	1.08	0.44	0.75	1	8.54	905.23	0.99	0.61	0.84
539	ALCTEKLE	8	-0.15	0.6	0.6	0.04	0.78	0.44	0.62	-1	4.54	906.17	0.61	0.41	0.54
540	LCTEKLEQ	8	-0.26	0.62	0.62	-0.62	0.93	0.53	0.88	-1	4.54	963.23	0.99	0.27	0.22
541	CTEKLEQW	8	-0.28	0.61	0.61	-1.21	0.93	0.33	1	-1	4.54	1036.28	0.61	0.31	0.21
542	TEKLEQWL	8	-0.22	0.6	0.6	-1.05	0.93	0.23	1	-1	4.54	1046.31	0.99	0.20	0.43
543	EKLEQWLC	8	-0.19	0.61	0.61	-0.65	0.93	0.15	0.88	-1	4.54	1048.34	0.61	0.16	0.36
544	KLEQWLCE	8	-0.19	0.61	0.61	-0.65	0.93	0.15	0.88	-1	4.54	1048.34	0.99	0.18	0.35
545	LEQWLCEK	8	-0.2	0.61	0.61	-0.65	0.93	0.15	0.88	-1	4.54	1048.34	0.99	0.10	0.30
546	EQWLCEKL	8	-0.19	0.61	0.61	-0.65	0.93	0.15	0.88	-1	4.54	1048.34	0.61	0.14	0.75
547	MRFFVPLFL	9	0.21	0.63	0.63	1.78	0.27	-1.21	0.44	1	10.11	1169.62	0.97	0.75	0.98
548	RFFVPLFLV	9	0.24	0.62	0.62	2.03	0.27	-1.23	0.44	1	10.11	1137.56	0.99	0.75	0.93
549	FFVPLFLVG	9	0.45	0.62	0.62	2.49	0	-1.57	0	0	5.88	1038.43	0.86	0.73	0.98
550	FVPLFLVGI	9	0.46	0.62	0.62	2.68	0	-1.49	0	0	5.88	1004.42	0.86	0.70	0.97
551	VPLFLVGIL	9	0.46	0.6	0.6	2.79	0	-1.41	0	0	5.88	970.41	0.99	0.69	0.98
552	PLFLVGILF	9	0.46	0.6	0.6	2.63	0	-1.52	0	0	5.88	1018.45	0.99	0.71	0.96
553	LFLVGILFP	9	0.46	0.6	0.6	2.63	0	-1.52	0	0	5.88	1018.45	0.97	0.63	0.89
554	FLVGILFPA	9	0.43	0.6	0.6	2.41	0	-1.38	0	0	5.88	976.36	0.86	0.69	0.98
555	LVGILFPAI	9	0.45	0.6	0.6	2.6	0	-1.3	0	0	5.88	942.35	0.99	0.45	0.89
556	VGILFPAIL	9	0.45	0.6	0.6	2.6	0	-1.3	0	0	5.88	942.35	0.99	0.54	0.97
557	GILFPAILA	9	0.41	0.58	0.58	2.33	0	-1.19	0	0	5.88	914.29	0.98	0.67	0.98
558	ILFPAILAK	9	0.27	0.58	0.58	1.94	0.41	-0.86	0.22	1	9.11	985.41	0.99	0.80	0.98
559	LFPAILAKQ	9	0.12	0.58	0.58	1.06	0.55	-0.63	0.44	1	9.11	1000.38	0.99	0.72	0.97
560	FPAILAKQF	9	0.12	0.6	0.6	0.94	0.55	-0.71	0.44	1	9.11	1034.39	0.61	0.70	0.98
561	PAILAKQFT	9	0.04	0.58	0.58	0.56	0.55	-0.48	0.56	1	9.11	988.32	0.99	0.68	0.95
562	AILAKQFTK	9	-0.08	0.62	0.62	0.3	0.95	-0.14	0.78	2	10.02	1019.38	0.61	0.68	0.97
563	ILAKQFTKC	9	-0.1	0.63	0.63	0.38	0.95	-0.2	0.78	2	9.36	1051.44	0.99	0.79	0.97
564	LAKQFTKCE	9	-0.25	0.62	0.62	-0.51	1.1	0.33	0.89	1	8.54	1067.39	0.99	0.75	0.82
565	AKQFTKCEL	9	-0.25	0.62	0.62	-0.51	1.1	0.33	0.89	1	8.54	1067.39	0.61	0.61	0.56
566	KQFTKCELS	9	-0.31	0.63	0.63	-0.8	1.1	0.42	1	1	8.54	1083.39	99.22%	0.39	0.39
567	QFTKCELSQ	9	-0.26	0.63	0.63	-0.76	0.83	0.11	1	0	6.32	1083.35	0.94	0.40	0.16
568	FTKCELSQL	9	-0.13	0.61	0.61	0.06	0.69	-0.11	0.78	0	6.32	1068.38	0.61	0.29	0.50
569	TKCELSQLL	9	-0.14	0.59	0.59	0.17	0.69	-0.03	0.78	0	6.32	1034.37	0.99	0.36	0.74
570	KCELSQLLK	9	-0.24	0.61	0.61	-0.19	1.1	0.34	0.89	1	8.54	1061.44	0.99	0.28	0.82
571	CELSQLLKD	9	-0.2	0.62	0.62	-0.14	0.69	0.34	0.78	-1	4.38	1048.35	0.61	0.25	0.39
572	ELSQLLKDI	9	-0.12	0.62	0.62	0.08	0.69	0.26	0.78	-1	4.38	1058.38	0.61	0.28	0.29
573	LSQLLKDID	9	-0.13	0.63	0.63	0.08	0.55	0.26	0.78	-1	4.21	1044.35	0.99	0.32	0.62
574	SQLLKDIDG	9	-0.17	0.65	0.65	-0.39	0.55	0.46	0.78	-1	4.21	988.24	0.99	0.31	0.11

575	QLLKDIDGY	9	-0.14	0.67	0.67	-0.44	0.55	0.17	0.78	-1	4.21	1064.34	0.94	0.29	0.06
576	LLKDIDGYG	9	-0.05	0.67	0.67	-0.1	0.41	0.14	0.56	-1	4.21	993.26	0.99	0.44	0.13
577	LKDIDGYGG	9	-0.09	0.69	0.69	-0.57	0.41	0.34	0.56	-1	4.21	937.15	0.99	0.41	0.04
578	KDIDGYGGI	9	-0.06	0.7	0.7	-0.49	0.41	0.34	0.56	-1	4.21	937.15	0.99	0.39	0.06
579	DIDGYGGIA	9	0.09	0.69	0.69	0.14	0	-0.04	0.33	-2	3.57	880.05	0.61	0.55	0.15
580	IDGYGGIAL	9	0.22	0.66	0.66	0.96	0	-0.58	0.22	-1	3.8	878.13	0.99	0.67	0.63
581	DGYGGIALP	9	0.14	0.62	0.62	0.28	0	-0.38	0.22	-1	3.8	862.08	0.86	0.70	0.58
582	GYGGIALPE	9	0.15	0.61	0.61	0.28	0.14	-0.38	0.22	-1	4	876.11	0.98	0.71	0.83
583	YGGIALPEL	9	0.19	0.6	0.6	0.74	0.14	-0.58	0.22	-1	4	932.22	0.99	0.68	0.59
584	GGIALPELI	9	0.27	0.6	0.6	1.39	0.14	-0.52	0.11	-1	4	882.21	0.98	0.68	0.94
585	GIALPELIC	9	0.25	0.59	0.59	1.71	0.14	-0.63	0.11	-1	4	928.29	0.98	0.70	0.85
586	IALPELICT	9	0.22	0.57	0.57	1.68	0.14	-0.68	0.22	-1	4	972.34	0.99	0.66	0.54
587	ALPELICTM	9	0.16	0.58	0.58	1.39	0.14	-0.62	0.22	-1	4	990.37	0.61	0.68	0.55
588	LPELICTMF	9	0.2	0.6	0.6	1.5	0.14	-0.84	0.22	-1	4	1066.47	0.99	0.61	0.16
589	PELICTMFH	9	0.1	0.54	0.54	0.72	0.3	-0.7	0.33	-0.5	5.25	1090.45	0.94	0.56	0.11
590	ELICTMFHT	9	0.09	0.56	0.56	0.82	0.3	-0.74	0.44	-0.5	5.25	1094.44	0.61	0.57	0.19
591	LICTMFHTS	9	0.13	0.55	0.55	1.12	0.16	-1.04	0.44	0.5	7.06	1052.4	0.99	0.57	0.94
592	ICTMFHTSG	9	0.09	0.56	0.56	0.66	0.16	-0.84	0.44	0.5	7.06	996.29	0.99	0.41	0.98
593	CTMFHTSGY	9	0.01	0.56	0.56	0.01	0.16	-0.9	0.56	0.5	7.06	1046.3	0.86	0.37	0.85
594	TMFHTSGYD	9	-0.08	0.58	0.58	-0.66	0.16	-0.46	0.67	-0.5	5.09	1058.25	0.99	0.35	0.11
595	MFHTSGYDT	9	-0.08	0.58	0.58	-0.66	0.16	-0.46	0.67	-0.5	5.09	1058.25	0.99	0.31	0.07
596	FHTSGYDTQ	9	-0.18	0.57	0.57	-1.26	0.3	-0.29	0.89	-0.5	5.09	1055.19	0.61	0.31	0.12
597	HTSGYDTQA	9	-0.22	0.55	0.55	-1.37	0.3	-0.07	0.89	-0.5	5.09	979.09	0.99	0.26	0.17
598	TSGYDTQAI	9	-0.1	0.63	0.63	-0.51	0.14	-0.21	0.78	-1	3.8	955.11	0.99	0.19	0.12
599	SGYDTQAIV	9	-0.02	0.64	0.64	0.03	0.14	-0.33	0.67	-1	3.8	953.14	0.99	0.28	0.13
600	GYDTQAIVE	9	-0.06	0.66	0.66	-0.27	0.28	-0.03	0.67	-2	3.67	995.18	0.98	0.46	0.14
601	YDTQAIVEN	9	-0.15	0.67	0.67	-0.61	0.28	-0.01	0.89	-2	3.67	1052.23	0.99	0.53	0.14
602	DTQAIVENN	9	-0.22	0.68	0.68	-0.86	0.28	0.27	1	-2	3.67	1003.16	0.86	0.51	0.29
603	TQAIVENNE	9	-0.21	0.67	0.67	-0.86	0.42	0.27	1	-2	3.8	1017.19	0.99	0.42	0.21
604	QAIVENNES	9	-0.22	0.67	0.67	-0.87	0.42	0.34	1	-2	3.8	1003.16	0.99	0.40	0.11
605	AIVENNEST	9	-0.16	0.65	0.65	-0.56	0.28	0.28	0.89	-2	3.8	976.13	0.61	0.42	0.11
606	IVENNESTE	9	-0.26	0.67	0.67	-1.14	0.42	0.67	1	-3	3.68	1034.17	0.94	0.44	0.06
607	VENNESTEY	9	-0.34	0.67	0.67	-1.79	0.42	0.61	1.11	-3	3.68	1084.18	0.99	0.38	0.09
608	ENNESTEYG	9	-0.38	0.67	0.67	-2.3	0.42	0.78	1.11	-3	3.68	1042.1	0.61	0.33	0.09
609	NNESTEYGL	9	-0.25	0.65	0.65	-1.49	0.28	0.24	1	-2	3.8	1026.15	0.94	0.28	0.09
610	NESTEYGLF	9	-0.11	0.64	0.64	-0.79	0.28	-0.06	0.78	-2	3.8	1059.22	0.99	0.47	0.14
611	ESTEYGLFQ	9	-0.12	0.63	0.63	-0.79	0.42	-0.06	0.78	-2	3.8	1073.25	0.61	0.49	0.18
612	STEYGLFQI	9	0.03	0.64	0.64	0.1	0.28	-0.59	0.67	-1	4	1057.3	0.99	0.53	0.23
613	TEYGLFQIS	9	0.03	0.64	0.64	0.1	0.28	-0.59	0.67	-1	4	1057.3	0.99	0.58	0.23
614	EYGLFQISN	9	-0.02	0.66	0.66	-0.21	0.28	-0.52	0.78	-1	4	1070.3	0.61	0.58	0.30
615	YGLFQISNK	9	-0.07	0.66	0.66	-0.26	0.55	-0.52	0.89	1	8.94	1069.36	0.99	0.59	0.49
616	GLFQISNKL	9	-0.01	0.64	0.64	0.31	0.55	-0.47	0.78	1	9.11	1019.35	0.98	0.70	0.94
617	LFQISNKLW	9	0.01	0.62	0.62	0.26	0.55	-0.84	0.89	1	9.11	1148.51	0.94	0.70	0.95
618	FQISNKLWC	9	-0.05	0.63	0.63	0.11	0.55	-0.76	0.89	1	8.57	1138.48	0.61	0.64	0.98
619	QISNKLWCK	9	-0.24	0.63	0.63	-0.63	0.95	-0.14	1.11	2	9.36	1119.48	0.99	0.60	0.91
620	ISNKLWCKS	9	-0.19	0.61	0.61	-0.33	0.82	-0.13	1	2	9.36	1078.42	0.99	0.63	0.85
621	SNKLWCKSS	9	-0.3	0.6	0.6	-0.92	0.82	0.1	1.11	2	9.36	1052.33	0.99	0.58	0.82
622	NKLWCKSSQ	9	-0.35	0.61	0.61	-1.22	0.95	0.09	1.22	2	9.36	1093.39	0.99	0.58	0.88
623	KLWCKSSQV	9	-0.21	0.61	0.61	-0.37	0.95	-0.1	1	2	9.36	1078.42	0.99	0.61	0.97
624	LWCKSSQVP	9	-0.1	0.57	0.57	-0.11	0.55	-0.43	0.78	1	8.57	1047.36	0.99	0.53	0.88
625	WCKSSQVPQ	9	-0.24	0.59	0.59	-0.92	0.69	-0.21	1	1	8.57	1062.33	0.99	0.48	0.77
626	CKSSQVPQS	9	-0.31	0.59	0.59	-0.91	0.69	0.2	1	1	8.57	963.19	0.86	0.45	0.06
627	KSSQVPQSR	9	-0.51	0.6	0.6	-1.69	0.96	0.64	1.44	2	11.01	1016.24	0.99	0.50	0.06
628	SSQVPQSRN	9	-0.45	0.61	0.61	-1.64	0.55	0.33	1.44	1	10.11	1002.17	0.99	0.45	0.12
629	SQVPQSRNI	9	-0.34	0.62	0.62	-1.06	0.55	0.1	1.33	1	10.11	1028.26	0.99	0.41	0.13
630	QVPQSRNIC	9	-0.31	0.63	0.63	-0.69	0.55	-0.04	1.22	1	8.6	1044.32	0.99	0.43	0.57
631	VPQSRNICD	9	-0.31	0.64	0.64	-0.69	0.41	0.27	1.11	0	6.16	1031.27	0.99	0.44	0.15
632	PQSRNICDI	9	-0.29	0.64	0.64	-0.66	0.41	0.23	1.11	0	6.16	1045.3	0.94	0.39	0.20
633	QSRNICDIS	9	-0.31	0.66	0.66	-0.57	0.41	0.27	1.22	0	6.16	1035.26	0.99	0.49	0.15
634	SRNICDISC	9	-0.23	0.66	0.66	0.1	0.27	0.13	1	0	6.13	1010.26	0.99	0.52	0.97
635	RNICDISCD	9	-0.28	0.68	0.68	-0.2	0.27	0.43	1	-1	4.21	1038.27	0.99	0.55	0.80
636	NICDISCDK	9	-0.21	0.68	0.68	-0.13	0.41	0.43	0.78	-1	4.21	1010.26	0.99	0.47	0.76
637	ICDISCDKF	9	-0.07	0.67	0.67	0.57	0.41	0.13	0.56	-1	4.21	1043.33	0.99	0.50	0.77
638	CDISCDKFL	9	-0.09	0.66	0.66	0.49	0.41	0.13	0.56	-1	4.21	1043.33	0.61	0.66	0.70

639	DISCDKFLD	9	-0.18	0.67	0.67	-0.18	0.41	0.58	0.67	-2	3.94	1055.28	0.61	0.45	0.36
640	ISCDKFLDD	9	-0.18	0.67	0.67	-0.18	0.41	0.58	0.67	-2	3.94	1055.28	0.99	0.39	0.32
641	SCDKFLDDD	9	-0.34	0.68	0.68	-1.07	0.41	1.11	0.78	-3	3.77	1057.2	0.99	0.42	0.59
642	CDKFLDDDI	9	-0.23	0.7	0.7	-0.48	0.41	0.88	0.67	-3	3.77	1083.29	0.61	0.45	0.25
643	DKFLDDDIT	9	-0.25	0.69	0.69	-0.83	0.41	0.94	0.78	-3	3.77	1081.26	0.61	0.44	0.09
644	KFLDDDITD	9	-0.25	0.69	0.69	-0.83	0.41	0.94	0.78	-3	3.77	1081.26	0.99	0.50	0.08
645	FLDDDITDD	9	-0.21	0.7	0.7	-0.79	0	0.94	0.67	-5	3.25	1068.17	0.61	0.46	0.14
646	LDDDITDDI	9	-0.2	0.7	0.7	-0.6	0	1.02	0.67	-5	3.25	1034.16	0.99	0.29	0.17
647	DDDITDDIM	9	-0.23	0.72	0.72	-0.81	0	1.08	0.67	-5	3.25	1052.19	0.61	0.42	0.16
648	DDITDDIMC	9	-0.14	0.71	0.71	-0.14	0	0.63	0.56	-4	3.33	1040.24	0.61	0.48	0.08
649	DITDDIMCA	9	-0.04	0.68	0.68	0.44	0	0.24	0.44	-3	3.43	996.23	0.61	0.51	0.05
650	ITDDIMCAK	9	-0.08	0.67	0.67	0.4	0.41	0.24	0.56	-1	4.21	1009.32	0.99	0.68	0.08
651	TDDIMCAKK	9	-0.28	0.67	0.67	-0.53	0.82	0.78	0.78	0	6.28	1024.33	0.99	0.77	0.63
652	DDIMCAKKI	9	-0.18	0.69	0.69	0.04	0.82	0.62	0.67	0	6.28	1036.39	0.61	0.80	0.57
653	DIMCAKKIL	9	-0.04	0.66	0.66	0.86	0.82	0.09	0.56	1	8.54	1034.47	0.61	0.88	0.96
654	IMCAKKILD	9	-0.04	0.66	0.66	0.86	0.82	0.09	0.56	1	8.54	1034.47	0.99	0.75	0.98
655	MCAKKILDI	9	-0.04	0.66	0.66	0.86	0.82	0.09	0.56	1	8.54	1034.47	0.99	0.79	0.92
656	CAKKILDIK	9	-0.19	0.65	0.65	0.21	1.22	0.57	0.78	2	9.25	1031.45	0.83	0.79	0.97
657	AKKILDIKG	9	-0.18	0.66	0.66	-0.11	1.22	0.68	0.78	2	9.72	985.37	0.61	0.65	0.82
658	KKILDIKGI	9	-0.13	0.68	0.68	0.19	1.22	0.53	0.78	2	9.72	1027.46	0.99	0.58	0.62
659	KILDIKGID	9	-0.08	0.69	0.69	0.23	0.82	0.53	0.67	0	6.31	1014.37	0.99	0.44	0.45
660	ILDIKGIDY	9	0.04	0.69	0.69	0.52	0.41	-0.06	0.56	-1	4.21	1049.37	0.99	0.31	0.35
661	LDIKGIDYW	9	0	0.67	0.67	-0.08	0.41	-0.23	0.67	-1	4.21	1122.42	0.99	0.46	0.15
662	DIKGIDYWL	9	0	0.67	0.67	-0.08	0.41	-0.23	0.67	-1	4.21	1122.42	0.86	0.51	0.13
663	IKGIDYWLA	9	0.11	0.64	0.64	0.51	0.41	-0.62	0.56	0	6.18	1078.41	0.99	0.54	0.74
664	KGIDYWLAH	9	-0.02	0.56	0.56	-0.34	0.57	-0.48	0.67	0.5	7.09	1102.39	0.99	0.54	0.67
665	GIDYWLAHK	9	-0.02	0.56	0.56	-0.34	0.57	-0.48	0.67	0.5	7.09	1102.39	0.98	0.74	0.86
666	IDYWLAHKA	9	-0.01	0.55	0.55	-0.1	0.57	-0.53	0.67	0.5	7.09	1116.41	0.99	0.77	0.90
667	DYWLAHKAL	9	-0.03	0.53	0.53	-0.18	0.57	-0.53	0.67	0.5	7.09	1116.41	0.86	0.82	0.97
668	YWLAHKALC	9	0.05	0.51	0.51	0.49	0.57	-0.98	0.56	1.5	8.54	1104.46	0.99	0.78	0.98
669	WLAHKALCT	9	0.03	0.49	0.49	0.56	0.57	-0.77	0.56	1.5	8.57	1042.39	0.99	0.74	0.96
670	LAHKALCTE	9	-0.08	0.51	0.51	0.27	0.71	-0.06	0.56	0.5	7.06	985.29	0.99	0.77	0.98
671	AHKALCTEK	9	-0.26	0.53	0.53	-0.59	1.12	0.48	0.78	1.5	8.54	1000.3	0.61	0.58	0.81
672	HKALCTEKL	9	-0.23	0.53	0.53	-0.37	1.12	0.33	0.78	1.5	8.54	1042.39	0.99	0.58	0.84
673	KALCTEKLE	9	-0.25	0.61	0.61	-0.4	1.1	0.72	0.78	0	6.46	1034.36	0.99	0.58	0.41
674	ALCTEKLEQ	9	-0.21	0.61	0.61	-0.36	0.83	0.41	0.78	-1	4.54	1034.32	0.61	0.37	0.25
675	LCTEKLEQW	9	-0.19	0.6	0.6	-0.66	0.83	0.09	0.89	-1	4.54	1149.46	0.99	0.25	0.46
676	CTEKLEQWL	9	-0.19	0.6	0.6	-0.66	0.83	0.09	0.89	-1	4.54	1149.46	0.61	0.22	0.63
677	TEKLEQWLC	9	-0.19	0.6	0.6	-0.66	0.83	0.09	0.89	-1	4.54	1149.46	0.99	0.14	0.45
678	EKLEQWLCE	9	-0.24	0.62	0.62	-0.97	0.97	0.47	0.89	-2	4.26	1177.47	0.61	0.15	0.13
679	KLEQWLCEK	9	-0.3	0.62	0.62	-1.01	1.24	0.47	1	0	6.46	1176.53	0.99	0.15	0.19
680	LEQWLCEKL	9	-0.11	0.6	0.6	-0.16	0.83	-0.07	0.78	-1	4.54	1161.52	0.99	0.15	0.67
681	MRFFVPLFLV	10	0.24	0.64	0.64	2.02	0.25	-1.24	0.4	1	10.11	1268.77	0.97	0.73	0.95
682	RFFVPLFLVG	10	0.23	0.63	0.63	1.79	0.25	-1.11	0.4	1	10.11	1194.63	0.99	0.73	0.99
683	FFVPLFLVGI	10	0.48	0.63	0.63	2.69	0	-1.59	0	0	5.88	1151.61	0.86	0.74	0.92
684	FVPLFLVGIL	10	0.47	0.61	0.61	2.79	0	-1.52	0	0	5.88	1117.6	0.86	0.74	0.98
685	VPLFLVGILF	10	0.47	0.61	0.61	2.79	0	-1.52	0	0	5.88	1117.6	0.97	0.69	0.87
686	PLFLVGILFP	10	0.41	0.58	0.58	2.21	0	-1.37	0	0	5.88	1115.58	0.99	0.71	0.98
687	LFLVGILFPA	10	0.44	0.6	0.6	2.55	0	-1.42	0	0	5.88	1089.54	0.99	0.71	0.83
688	FLVGILFPAI	10	0.46	0.61	0.61	2.62	0	-1.42	0	0	5.88	1089.54	0.86	0.66	0.98
689	LVGILFPAIL	10	0.45	0.6	0.6	2.72	0	-1.35	0	0	5.88	1055.53	0.99	0.47	0.76
690	VGILFPAILA	10	0.43	0.59	0.59	2.52	0	-1.22	0	0	5.88	1013.44	0.99	0.65	0.95
691	GILFPAILAK	10	0.26	0.59	0.59	1.71	0.37	-0.77	0.2	1	9.11	1042.48	0.95	0.80	0.98
692	ILFPAILAKQ	10	0.18	0.59	0.59	1.4	0.49	-0.75	0.4	1	9.11	1113.56	0.99	0.75	0.98
693	LFPAILAKQF	10	0.16	0.59	0.59	1.23	0.49	-0.82	0.4	1	9.11	1147.57	0.98	0.74	0.98
694	FPAILAKQFT	10	0.09	0.59	0.59	0.78	0.49	-0.68	0.5	1	9.11	1135.51	0.80	0.73	0.97
695	PAILAKQFTK	10	-0.08	0.59	0.59	0.11	0.86	-0.13	0.7	2	10.02	1116.51	0.98	0.73	0.98
696	AILAKQFTKC	10	-0.07	0.62	0.62	0.52	0.86	-0.23	0.7	2	9.36	1122.53	0.61	0.71	0.97
697	ILAKQFTKCE	10	-0.15	0.63	0.63	-0.01	0.99	0.12	0.8	1	8.54	1180.57	0.99	0.76	0.68
698	LAKQFTKCEL	10	-0.17	0.61	0.61	-0.08	0.99	0.12	0.8	1	8.54	1180.57	0.99	0.67	0.88
699	AKQFTKCELS	10	-0.25	0.61	0.61	-0.54	0.99	0.33	0.9	1	8.54	1154.48	0.61	0.56	0.42
700	KQFTKCELSQ	10	-0.35	0.63	0.63	-1.07	1.11	0.4	1.1	1	8.54	1211.54	0.99	0.42	0.29
701	QFTKCELSQL	10	-0.18	0.62	0.62	-0.3	0.74	-0.08	0.9	0	6.32	1196.53	0.94	0.30	0.16
702	FTKCELSQLL	10	-0.06	0.6	0.6	0.43	0.62	-0.28	0.7	0	6.32	1181.56	0.61	0.32	0.69

703	TKCELSQLLK	10	-0.23	0.6	0.6	-0.24	0.99	0.27	0.9	1	8.54	1162.56	0.99	0.34	0.82
704	KCELSQLLKD	10	-0.29	0.62	0.62	-0.52	0.99	0.61	0.9	0	6.38	1176.54	0.99	0.27	0.65
705	CELSQLLKDI	10	-0.1	0.62	0.62	0.32	0.62	0.13	0.7	-1	4.38	1161.53	0.61	0.27	0.17
706	ELSQLLKDID	10	-0.18	0.64	0.64	-0.28	0.62	0.53	0.8	-2	4.03	1173.48	0.61	0.29	0.14
707	LSQLLKDIDG	10	-0.1	0.64	0.64	0.03	0.49	0.23	0.7	-1	4.21	1101.42	0.99	0.31	0.26
708	SQLLKDIDGY	10	-0.15	0.66	0.66	-0.48	0.49	0.18	0.8	-1	4.21	1151.43	0.99	0.27	0.07
709	QLLKDIDGYG	10	-0.11	0.67	0.67	-0.44	0.49	0.15	0.7	-1	4.21	1121.41	0.94	0.29	0.05
710	LLKDIDGYGG	10	-0.02	0.67	0.67	-0.13	0.37	0.13	0.5	-1	4.21	1050.33	0.99	0.44	0.20
711	LKDIDGYGGI	10	-0.01	0.69	0.69	-0.06	0.37	0.13	0.5	-1	4.21	1050.33	0.99	0.38	0.05
712	KDIDGYGGIA	10	-0.03	0.69	0.69	-0.26	0.37	0.26	0.5	-1	4.21	1008.24	0.99	0.45	0.06
713	DIDGYGGIAL	10	0.13	0.67	0.67	0.51	0	-0.22	0.3	-2	3.57	993.23	0.61	0.63	0.15
714	IDGYGGIALP	10	0.2	0.63	0.63	0.7	0	-0.52	0.2	-1	3.8	975.26	0.99	0.70	0.62
715	DGYGGIALPE	10	0.06	0.63	0.63	-0.1	0.13	-0.04	0.3	-2	3.67	991.21	0.86	0.70	0.16
716	GYGGIALPEL	10	0.18	0.61	0.61	0.63	0.13	-0.52	0.2	-1	4	989.29	0.98	0.67	0.76
717	YGGIALPELI	10	0.24	0.61	0.61	1.12	0.13	-0.7	0.2	-1	4	1045.4	0.99	0.69	0.63
718	GGIALPELIC	10	0.24	0.6	0.6	1.5	0.13	-0.57	0.1	-1	4	985.36	0.98	0.66	0.93
719	GIALPELICT	10	0.21	0.59	0.59	1.47	0.13	-0.61	0.2	-1	4	1029.41	0.98	0.67	0.71
720	IALPELICTM	10	0.22	0.6	0.6	1.7	0.13	-0.74	0.2	-1	4	1103.55	0.99	0.67	0.67
721	ALPELICTMF	10	0.21	0.6	0.6	1.53	0.13	-0.81	0.2	-1	4	1137.56	0.61	0.67	0.26
722	LPELICTMFH	10	0.14	0.54	0.54	1.03	0.27	-0.81	0.3	-0.5	5.25	1203.63	0.97	0.65	0.23
723	PELICTMFHT	10	0.07	0.54	0.54	0.58	0.27	-0.67	0.4	-0.5	5.25	1191.57	0.94	0.59	0.10
724	ELICTMFHTS	10	0.05	0.56	0.56	0.66	0.27	-0.64	0.5	-0.5	5.25	1181.53	0.61	0.55	0.16
725	LICTMFHTSG	10	0.13	0.56	0.56	0.97	0.14	-0.94	0.4	0.5	7.06	1109.47	0.99	0.43	0.95
726	ICTMFHTSGY	10	0.08	0.58	0.58	0.46	0.14	-0.99	0.5	0.5	7.06	1159.48	0.99	0.39	0.91
727	CTMFHTSGYD	10	-0.07	0.58	0.58	-0.34	0.14	-0.51	0.6	-0.5	5.09	1161.4	0.86	0.37	0.18
728	TMFHTSGYDT	10	-0.09	0.57	0.57	-0.66	0.14	-0.45	0.7	-0.5	5.09	1159.37	0.99	0.34	0.12
729	MFHTSGYDTQ	10	-0.14	0.59	0.59	-0.94	0.27	-0.39	0.8	-0.5	5.09	1186.4	0.98	0.31	0.10
730	FHTSGYDTQA	10	-0.14	0.56	0.56	-0.95	0.27	-0.31	0.8	-0.5	5.09	1126.28	0.80	0.32	0.15
731	HTSGYDTQAI	10	-0.13	0.56	0.56	-0.78	0.27	-0.24	0.8	-0.5	5.09	1092.27	0.99	0.24	0.09
732	TSGYDTQAIV	10	-0.03	0.63	0.63	-0.04	0.12	-0.34	0.7	-1	3.8	1054.26	0.99	0.26	0.12
733	SGYDTQAIVE	10	-0.08	0.65	0.65	-0.32	0.25	0	0.7	-2	3.67	1082.27	0.99	0.32	0.10
734	GYDTQAIVEN	10	-0.11	0.67	0.67	-0.59	0.25	-0.01	0.8	-2	3.67	1109.3	0.98	0.48	0.05
735	YDTQAIVENN	10	-0.19	0.68	0.68	-0.9	0.25	0.01	1	-2	3.67	1166.35	0.99	0.50	0.19
736	DTQAIVENNE	10	-0.26	0.68	0.68	-1.12	0.38	0.54	1	-3	3.58	1132.29	0.86	0.39	0.13
737	TQAIVENNES	10	-0.21	0.65	0.65	-0.85	0.38	0.27	1	-2	3.8	1104.28	0.99	0.41	0.11
738	QAIVENNEST	10	-0.21	0.65	0.65	-0.85	0.38	0.27	1	-2	3.8	1104.28	0.99	0.38	0.11
739	AIVENNESTE	10	-0.21	0.65	0.65	-0.85	0.38	0.55	0.9	-3	3.68	1105.26	0.61	0.41	0.06
740	IVENNESTEY	10	-0.23	0.67	0.67	-1.16	0.38	0.37	1	-3	3.68	1197.36	0.94	0.46	0.05
741	VENNESTEYG	10	-0.29	0.67	0.67	-1.65	0.38	0.55	1	-3	3.68	1141.25	0.99	0.41	0.05
742	ENNESTEYGL	10	-0.29	0.65	0.65	-1.69	0.38	0.52	1	-3	3.68	1155.28	0.61	0.29	0.11
743	NNESTEYGLF	10	-0.16	0.66	0.66	-1.06	0.25	-0.03	0.9	-2	3.8	1173.34	0.94	0.43	0.10
744	NESTEYGLFQ	10	-0.17	0.65	0.65	-1.06	0.38	-0.03	0.9	-2	3.8	1187.37	0.99	0.43	0.16
745	ESTEYGLFQI	10	-0.03	0.64	0.64	-0.26	0.38	-0.23	0.7	-2	3.8	1186.43	0.61	0.52	0.17
746	STEYGLFQIS	10	0	0.63	0.63	0.01	0.25	-0.5	0.7	-1	4	1144.39	0.99	0.52	0.38
747	TEYGLFQISN	10	-0.03	0.65	0.65	-0.26	0.25	-0.51	0.8	-1	4	1171.42	0.99	0.52	0.13
748	EYGLFQISNK	10	-0.13	0.66	0.66	-0.58	0.62	-0.17	0.9	0	6.35	1198.49	0.61	0.56	0.42
749	YGLFQISNKL	10	-0.01	0.65	0.65	0.15	0.49	-0.65	0.8	1	8.94	1182.54	0.99	0.66	0.75
750	GLFQISNKLW	10	0.02	0.63	0.63	0.19	0.49	-0.76	0.8	1	9.11	1205.58	0.98	0.64	0.99
751	LFQISNKLWC	10	0.01	0.62	0.62	0.48	0.49	-0.86	0.8	1	8.57	1251.66	0.94	0.69	0.97
752	FQISNKLWCK	10	-0.15	0.64	0.64	-0.29	0.86	-0.38	1	2	9.36	1266.67	0.61	0.55	0.98
753	QISNKLWCKS	10	-0.24	0.62	0.62	-0.65	0.86	-0.1	1.1	2	9.36	1206.57	0.99	0.61	0.78
754	ISNKLWCKSS	10	-0.2	0.61	0.61	-0.38	0.73	-0.09	1	2	9.36	1165.51	0.99	0.62	0.68
755	SNKLWCKSSQ	10	-0.34	0.6	0.6	-1.18	0.86	0.11	1.2	2	9.36	1180.48	0.99	0.56	0.73
756	NKLWCKSSQV	10	-0.26	0.62	0.62	-0.68	0.86	-0.07	1.1	2	9.36	1192.54	0.99	0.57	0.59
757	KLWCKSSQVP	10	-0.2	0.58	0.58	-0.49	0.86	-0.09	0.9	2	9.36	1175.55	0.99	0.58	0.98
758	LWCKSSQVPQ	10	-0.16	0.58	0.58	-0.45	0.62	-0.37	0.9	1	8.57	1175.51	0.99	0.47	0.78
759	WCKSSQVPQS	10	-0.24	0.58	0.58	-0.91	0.62	-0.16	1	1	8.57	1149.42	0.99	0.47	0.50
760	CKSSQVPQSR	10	-0.45	0.6	0.6	-1.27	0.86	0.48	1.3	2	9.55	1119.39	0.86	0.42	0.05
761	KSSQVPQSRN	10	-0.52	0.61	0.61	-1.87	0.86	0.6	1.5	2	11.01	1130.36	0.99	0.48	0.06
762	SSQVPQSRNI	10	-0.34	0.61	0.61	-1.03	0.49	0.12	1.3	1	10.11	1115.35	0.99	0.42	0.11
763	SQVPQSRNIC	10	-0.31	0.62	0.62	-0.7	0.49	-0.01	1.2	1	8.6	1131.41	0.99	0.44	0.50
764	QVPQSRNICD	10	-0.35	0.65	0.65	-0.97	0.49	0.26	1.2	0	6.16	1159.42	0.99	0.48	0.09
765	VPQSRNICDI	10	-0.21	0.65	0.65	-0.17	0.37	0.06	1	0	6.16	1144.45	0.99	0.47	0.17
766	PQSRNICDIS	10	-0.29	0.63	0.63	-0.67	0.37	0.24	1.1	0	6.16	1132.39	0.94	0.44	0.11

767	QSRNICDISC	10	-0.28	0.66	0.66	-0.26	0.37	0.14	1.1	0	6.13	1138.41	0.99	0.49	0.95
768	SRNICDISCD	10	-0.28	0.67	0.67	-0.26	0.25	0.42	1	-1	4.21	1125.36	0.99	0.52	0.79
769	RNICDISCDK	10	-0.37	0.68	0.68	-0.57	0.61	0.69	1.1	0	6.25	1166.46	0.99	0.48	0.72
770	NICDISCDKF	10	-0.13	0.68	0.68	0.16	0.37	0.14	0.7	-1	4.21	1157.45	0.99	0.50	0.74
771	ICDISCDKFL	10	-0.01	0.66	0.66	0.89	0.37	-0.06	0.5	-1	4.21	1156.51	0.99	0.66	0.79
772	CDISCDKFLD	10	-0.16	0.67	0.67	0.09	0.37	0.42	0.6	-2	3.94	1158.43	0.61	0.46	0.43
773	DISCDKFLDD	10	-0.23	0.68	0.68	-0.51	0.37	0.82	0.7	-3	3.77	1170.38	0.61	0.43	0.19
774	ISCDKFLDDD	10	-0.23	0.68	0.68	-0.51	0.37	0.82	0.7	-3	3.77	1170.38	0.99	0.40	0.24
775	SCDKFLDDDI	10	-0.23	0.68	0.68	-0.51	0.37	0.82	0.7	-3	3.77	1170.38	0.99	0.46	0.44
776	CDKFLDDDIT	10	-0.23	0.68	0.68	-0.5	0.37	0.75	0.7	-3	3.77	1184.41	0.61	0.47	0.06
777	DKFLDDDITD	10	-0.3	0.69	0.69	-1.1	0.37	1.15	0.8	-4	3.66	1196.36	0.61	0.43	0.09
778	KFLDDDITDD	10	-0.3	0.69	0.69	-1.1	0.37	1.15	0.8	-4	3.66	1196.36	0.99	0.51	0.10
779	FLDDDITDDI	10	-0.12	0.7	0.7	-0.26	0	0.67	0.6	-5	3.25	1181.35	0.61	0.48	0.10
780	LDDDITDDIM	10	-0.15	0.7	0.7	-0.35	0	0.79	0.6	-5	3.25	1165.37	0.99	0.28	0.08
781	DDDITDDIMC	10	-0.2	0.71	0.71	-0.48	0	0.87	0.6	-5	3.25	1155.34	0.61	0.48	0.09
782	DDITDDIMCA	10	-0.11	0.69	0.69	0.05	0	0.52	0.5	-4	3.33	1111.33	0.61	0.51	0.04
783	DITDDIMCAK	10	-0.14	0.68	0.68	0.01	0.37	0.52	0.6	-2	3.94	1124.42	0.61	0.66	0.04
784	ITDDIMCAKK	10	-0.18	0.67	0.67	-0.03	0.73	0.52	0.7	0	6.28	1137.51	0.99	0.71	0.48
785	TDDIMCAKKI	10	-0.18	0.67	0.67	-0.03	0.73	0.52	0.7	0	6.28	1137.51	0.99	0.74	0.40
786	DDIMCAKKIL	10	-0.11	0.67	0.67	0.42	0.73	0.38	0.6	0	6.28	1149.57	0.61	0.83	0.93
787	DIMCAKKILD	10	-0.11	0.67	0.67	0.42	0.73	0.38	0.6	0	6.28	1149.57	0.61	0.73	0.83
788	IMCAKKILDI	10	0.03	0.67	0.67	1.22	0.73	-0.1	0.5	1	8.54	1147.65	0.99	0.73	0.91
789	MCAKKILDIK	10	-0.15	0.67	0.67	0.38	1.1	0.38	0.7	2	9.25	1162.66	0.99	0.74	0.93
790	CAKKILDIKG	10	-0.16	0.66	0.66	0.15	1.1	0.51	0.7	2	9.25	1088.52	0.83	0.65	0.94
791	AKKILDIKGI	10	-0.09	0.66	0.66	0.35	1.1	0.43	0.7	2	9.72	1098.55	0.61	0.59	0.70
792	KKILDIKGID	10	-0.19	0.69	0.69	-0.18	1.1	0.78	0.8	1	8.83	1142.56	0.99	0.55	0.55
793	KILDIKGIDY	10	-0.07	0.69	0.69	0.08	0.73	0.25	0.7	0	6.3	1177.56	0.99	0.43	0.30
794	ILDIKGIDYW	10	0.07	0.67	0.67	0.38	0.37	-0.39	0.6	-1	4.21	1235.6	0.99	0.47	0.23
795	LDIKGIDYWL	10	0.05	0.65	0.65	0.31	0.37	-0.39	0.6	-1	4.21	1235.6	0.99	0.45	0.35
796	DIKGIDYWLA	10	0.03	0.65	0.65	0.11	0.37	-0.26	0.6	-1	4.21	1193.51	0.61	0.53	0.34
797	IKGIDYWLAH	10	0.06	0.58	0.58	0.14	0.51	-0.61	0.6	0.5	7.09	1215.57	0.97	0.53	0.61
798	KGIDYWLAHK	10	-0.13	0.57	0.57	-0.7	0.88	-0.13	0.8	1.5	8.84	1230.58	0.99	0.56	0.82
799	GIDYWLAHKA	10	0.01	0.56	0.56	-0.13	0.51	-0.48	0.6	0.5	7.09	1173.48	0.98	0.76	0.90
800	IDYWLAHKAL	10	0.05	0.54	0.54	0.29	0.51	-0.66	0.6	0.5	7.09	1229.59	0.99	0.78	0.86
801	DYWLAHKALC	10	-0.02	0.54	0.54	0.09	0.51	-0.58	0.6	0.5	7.06	1219.56	0.86	0.74	0.97
802	YWLAHKALCT	10	0.03	0.51	0.51	0.37	0.51	-0.92	0.6	1.5	8.54	1205.58	0.99	0.77	0.96
803	WLAHKALCTE	10	-0.03	0.51	0.51	0.15	0.64	-0.39	0.6	0.5	7.06	1171.52	0.99	0.70	0.98
804	LAHKALCTEK	10	-0.18	0.53	0.53	-0.15	1.01	0.25	0.7	1.5	8.54	1113.48	0.99	0.71	0.84
805	AHKALCTEKL	10	-0.18	0.53	0.53	-0.15	1.01	0.25	0.7	1.5	8.54	1113.48	0.61	0.55	0.90
806	HKALCTEKLE	10	-0.27	0.55	0.55	-0.68	1.13	0.6	0.8	0.5	7.07	1171.52	0.99	0.52	0.17
807	KALCTEKLEQ	10	-0.3	0.61	0.61	-0.71	1.11	0.67	0.9	0	6.46	1162.51	0.99	0.49	0.23
808	ALCTEKLEQW	10	-0.15	0.59	0.59	-0.41	0.75	0.03	0.8	-1	4.54	1220.55	0.61	0.33	0.60
809	LCTEKLEQWL	10	-0.12	0.6	0.6	-0.21	0.75	-0.1	0.8	-1	4.54	1262.64	0.99	0.16	0.59
810	CTEKLEQWLC	10	-0.17	0.61	0.61	-0.34	0.75	-0.02	0.8	-1	4.54	1252.61	0.86	0.16	0.77
811	TEKLEQWLCE	10	-0.24	0.61	0.61	-0.94	0.87	0.38	0.9	-2	4.26	1278.59	0.99	0.14	0.11
812	EKLEQWLCEK	10	-0.33	0.63	0.63	-1.26	1.24	0.72	1	-1	4.79	1305.66	0.61	0.15	0.08
813	KLEQWLCEKL	10	-0.21	0.61	0.61	-0.53	1.11	0.24	0.9	0	6.46	1289.71	0.99	0.22	0.79
814	MRFFVPLFLVG	11	0.23	0.64	0.64	1.8	0.22	-1.13	0.36	1	10.11	1325.84	0.93	0.71	0.99
815	RFFVPLFLVGI	11	0.28	0.63	0.63	2.04	0.22	-1.17	0.36	1	10.11	1307.81	0.98	0.74	0.92
816	FFVPLFLVGIL	11	0.48	0.62	0.62	2.79	0	-1.61	0	0	5.88	1264.79	0.94	0.76	0.98
817	FVPLFLVGILF	11	0.48	0.62	0.62	2.79	0	-1.61	0	0	5.88	1264.79	0.94	0.74	0.98
818	VPLFLVGILFP	11	0.42	0.59	0.59	2.39	0	-1.38	0	0	5.88	1214.73	0.99	0.69	0.93
819	PLFLVGILFPA	11	0.4	0.57	0.57	2.17	0	-1.29	0	0	5.88	1186.67	0.98	0.75	0.92
820	LFLVGILFPAI	11	0.47	0.6	0.6	2.73	0	-1.45	0	0	5.88	1202.72	0.97	0.66	0.86
821	FLVGILFPAIL	11	0.47	0.6	0.6	2.73	0	-1.45	0	0	5.88	1202.72	0.94	0.65	0.96
822	LVGILFPAILA	11	0.44	0.59	0.59	2.64	0	-1.27	0	0	5.88	1126.62	0.98	0.57	0.68
823	VGILFPAILAK	11	0.29	0.6	0.6	1.94	0.33	-0.84	0.18	1	9.11	1141.63	0.98	0.76	0.98
824	GILFPAILAKQ	11	0.18	0.6	0.6	1.24	0.45	-0.68	0.36	1	9.11	1170.63	0.95	0.73	0.98
825	ILFPAILAKQF	11	0.22	0.6	0.6	1.53	0.45	-0.91	0.36	1	9.11	1260.75	0.98	0.75	0.98
826	LFPAILAKQFT	11	0.13	0.59	0.59	1.05	0.45	-0.78	0.45	1	9.11	1248.69	0.98	0.73	0.91
827	FPAILAKQFTK	11	-0.01	0.6	0.6	0.35	0.78	-0.35	0.64	2	10.02	1263.7	0.80	0.74	0.98
828	PAILAKQFTKC	11	-0.07	0.59	0.59	0.33	0.78	-0.21	0.64	2	9.36	1219.66	0.93	0.73	0.95
829	AILAKQFTKCE	11	-0.12	0.62	0.62	0.15	0.9	0.06	0.73	1	8.54	1251.66	0.61	0.69	0.93
830	ILAKQFTKCEL	11	-0.09	0.62	0.62	0.34	0.9	-0.05	0.73	1	8.54	1293.75	0.99	0.68	0.68

831	LAKQFTKCELS	11	-0.18	0.61	0.61	-0.15	0.9	0.14	0.82	1	8.54	1267.66	0.99	0.60	0.83
832	AKQFTKCELSQ	11	-0.29	0.62	0.62	-0.81	1.01	0.32	1	1	8.54	1282.63	0.61	0.54	0.31
833	KQFTKCELSQL	11	-0.27	0.62	0.62	-0.63	1.01	0.2	1	1	8.54	1324.72	0.99	0.33	0.28
834	QFTKCELSQLL	11	-0.12	0.61	0.61	0.07	0.68	-0.24	0.82	0	6.32	1309.71	0.94	0.33	0.41
835	FTKCELSQLLK	11	-0.16	0.61	0.61	0.04	0.9	0.02	0.82	1	8.54	1309.75	0.61	0.31	0.84
836	TKCELSQLLKD	11	-0.28	0.61	0.61	-0.54	0.9	0.52	0.91	0	6.38	1277.66	0.99	0.33	0.55
837	KCELSQLLKDI	11	-0.19	0.63	0.63	-0.06	0.9	0.39	0.82	0	6.38	1289.72	0.99	0.30	0.42
838	CELSQLLKDID	11	-0.16	0.64	0.64	-0.03	0.56	0.39	0.73	-2	4.03	1276.63	0.61	0.31	0.08
839	ELSQLLKDIDG	11	-0.15	0.64	0.64	-0.29	0.56	0.48	0.73	-2	4.03	1230.55	0.61	0.32	0.06
840	LSQLLKDIDGY	11	-0.09	0.64	0.64	-0.09	0.45	0	0.73	-1	4.21	1264.61	0.99	0.30	0.10
841	SQLLKDIDGYG	11	-0.12	0.66	0.66	-0.47	0.45	0.16	0.73	-1	4.21	1208.5	0.99	0.30	0.11
842	QLLKDIDGYGG	11	-0.09	0.67	0.67	-0.44	0.45	0.14	0.64	-1	4.21	1178.48	0.94	0.32	0.05
843	LLKDIDGYGGI	11	0.04	0.67	0.67	0.29	0.33	-0.05	0.45	-1	4.21	1163.51	0.99	0.41	0.15
844	LKDIDGYGGIA	11	0.02	0.67	0.67	0.11	0.33	0.07	0.45	-1	4.21	1121.42	0.99	0.42	0.07
845	KDIDGYGGIAL	11	0.02	0.67	0.67	0.11	0.33	0.07	0.45	-1	4.21	1121.42	0.99	0.54	0.09
846	DIDGYGGIALP	11	0.11	0.64	0.64	0.32	0	-0.2	0.27	-2	3.57	1090.36	0.61	0.63	0.34
847	IDGYGGIALPE	11	0.12	0.64	0.64	0.32	0.12	-0.2	0.27	-2	3.67	1104.39	0.99	0.67	0.15
848	DGYGGIALPEL	11	0.1	0.62	0.62	0.25	0.12	-0.2	0.27	-2	3.67	1104.39	0.86	0.66	0.17
849	GYGGIALPELI	11	0.23	0.61	0.61	0.98	0.12	-0.64	0.18	-1	4	1102.47	0.98	0.66	0.82
850	YGGIALPELIC	11	0.22	0.61	0.61	1.25	0.12	-0.73	0.18	-1	4	1148.55	0.99	0.67	0.88
851	GGIALPELICT	11	0.21	0.59	0.59	1.3	0.12	-0.55	0.18	-1	4	1086.48	0.98	0.64	0.87
852	GIALPELICTM	11	0.21	0.6	0.6	1.51	0.12	-0.67	0.18	-1	4	1160.62	0.98	0.68	0.68
853	IALPELICTMF	11	0.26	0.6	0.6	1.8	0.12	-0.9	0.18	-1	4	1250.74	0.97	0.65	0.37
854	ALPELICTMFH	11	0.15	0.54	0.54	1.1	0.25	-0.78	0.27	-0.5	5.25	1274.72	0.61	0.68	0.31
855	LPELICTMFHT	11	0.11	0.54	0.54	0.87	0.25	-0.77	0.36	-0.5	5.25	1304.75	0.99	0.63	0.09
856	PELICTMFHTS	11	0.04	0.54	0.54	0.45	0.25	-0.58	0.45	-0.5	5.25	1278.66	0.94	0.55	0.05
857	ELICTMFHTSG	11	0.06	0.57	0.57	0.56	0.25	-0.58	0.45	-0.5	5.25	1238.6	0.61	0.41	0.15
858	LICTMFHTSGY	11	0.12	0.57	0.57	0.76	0.13	-1.06	0.45	0.5	7.06	1272.66	0.98	0.39	0.79
859	ICTMFHTSGYD	11	0.01	0.59	0.59	0.1	0.13	-0.63	0.55	-0.5	5.09	1274.58	0.99	0.38	0.41
860	CTMFHTSGYDT	11	-0.08	0.58	0.58	-0.37	0.13	-0.5	0.64	-0.5	5.09	1262.52	0.94	0.36	0.27
861	TMFHTSGYDTQ	11	-0.14	0.58	0.58	-0.92	0.25	-0.39	0.82	-0.5	5.09	1287.52	0.98	0.35	0.11
862	MFHTSGYDTQA	11	-0.1	0.58	0.58	-0.69	0.25	-0.4	0.73	-0.5	5.09	1257.49	0.98	0.32	0.08
863	FHTSGYDTQAI	11	-0.06	0.58	0.58	-0.45	0.25	-0.45	0.73	-0.5	5.09	1239.46	0.80	0.28	0.09
864	HTSGYDTQAIV	11	-0.07	0.58	0.58	-0.33	0.25	-0.35	0.73	-0.5	5.09	1191.42	0.99	0.29	0.09
865	TSGYDTQAIVE	11	-0.09	0.64	0.64	-0.35	0.23	-0.04	0.73	-2	3.67	1183.39	0.98	0.31	0.09
866	SGYDTQAIVEN	11	-0.13	0.66	0.66	-0.61	0.23	0.02	0.82	-2	3.67	1196.39	0.93	0.35	0.07
867	GYDTQAIVENN	11	-0.16	0.68	0.68	-0.85	0.23	0.01	0.91	-2	3.67	1223.42	0.98	0.46	0.05
868	YDTQAIVENNE	11	-0.23	0.68	0.68	-1.14	0.34	0.28	1	-3	3.58	1295.48	0.99	0.38	0.08
869	DTQAIVENNES	11	-0.26	0.66	0.66	-1.09	0.34	0.52	1	-3	3.58	1219.38	0.61	0.38	0.07
870	TQAIVENNEST	11	-0.21	0.64	0.64	-0.84	0.34	0.21	1	-2	3.8	1205.4	0.99	0.39	0.21
871	QAIVENNESTE	11	-0.25	0.66	0.66	-1.09	0.46	0.52	1	-3	3.68	1233.41	0.99	0.39	0.06
872	AIVENNESTEY	11	-0.19	0.66	0.66	-0.89	0.35	0.29	0.91	-3	3.68	1268.45	0.61	0.46	0.05
873	IVENNESTEYG	11	-0.19	0.67	0.67	-1.09	0.35	0.34	0.91	-3	3.68	1254.43	0.94	0.51	0.05
874	VENNESTEYGL	11	-0.21	0.66	0.66	-1.15	0.35	0.34	0.91	-3	3.68	1254.43	0.99	0.39	0.09
875	ENNESTEYGLF	11	-0.21	0.66	0.66	-1.28	0.35	0.25	0.91	-3	3.68	1302.47	0.61	0.46	0.12
876	NNESTEYGLFQ	11	-0.21	0.66	0.66	-1.28	0.34	-0.01	1	-2	3.8	1301.49	0.94	0.43	0.11
877	NESTEYGLFQI	11	-0.09	0.65	0.65	-0.55	0.34	-0.19	0.82	-2	3.8	1300.55	0.99	0.48	0.15
878	ESTEYGLFQIS	11	-0.05	0.63	0.63	-0.31	0.34	-0.18	0.73	-2	3.8	1273.52	0.61	0.53	0.17
879	STEYGLFQISN	11	-0.05	0.64	0.64	-0.31	0.23	-0.44	0.82	-1	4	1258.51	0.99	0.49	0.11
880	TEYGLFQISNK	11	-0.13	0.65	0.65	-0.59	0.56	-0.19	0.91	0	6.35	1299.61	0.99	0.54	0.15
881	EYGLFQISNKL	11	-0.07	0.65	0.65	-0.18	0.56	-0.32	0.82	0	6.35	1311.67	0.61	0.65	0.48
882	YGLFQISNKLW	11	0.02	0.64	0.64	0.05	0.45	-0.9	0.82	1	8.94	1368.77	0.99	0.63	0.90
883	GLFQISNKLWC	11	0.03	0.63	0.63	0.4	0.45	-0.78	0.73	1	8.57	1308.73	0.98	0.64	0.97
884	LFQISNKLWCK	11	-0.09	0.63	0.63	0.08	0.78	-0.51	0.91	2	9.36	1379.85	0.94	0.60	0.99
885	FQISNKLWCKS	11	-0.16	0.63	0.63	-0.34	0.78	-0.32	1	2	9.36	1353.76	0.61	0.57	0.96
886	QISNKLWCKSS	11	-0.24	0.61	0.61	-0.66	0.78	-0.06	1.09	2	9.36	1293.66	0.99	0.63	0.71
887	ISNKLWCKSSQ	11	-0.24	0.61	0.61	-0.66	0.78	-0.06	1.09	2	9.36	1293.66	0.99	0.63	0.49
888	SNKLWCKSSQV	11	-0.26	0.61	0.61	-0.69	0.78	-0.04	1.09	2	9.36	1279.63	0.99	0.57	0.22
889	NKLWCKSSQVP	11	-0.24	0.6	0.6	-0.76	0.78	-0.06	1	2	9.36	1289.67	0.99	0.56	0.96
890	KLWCKSSQVPQ	11	-0.24	0.59	0.59	-0.76	0.89	-0.06	1	2	9.36	1303.7	0.99	0.55	0.98
891	LWCKSSQVPQS	11	-0.17	0.58	0.58	-0.48	0.56	-0.31	0.91	1	8.57	1262.6	0.99	0.46	0.56
892	WCKSSQVPQSR	11	-0.38	0.59	0.59	-1.24	0.78	0.13	1.27	2	9.55	1305.62	0.99	0.43	0.41
893	CKSSQVPQSRN	11	-0.47	0.61	0.61	-1.47	0.78	0.45	1.36	2	9.55	1233.51	0.86	0.41	0.05
894	KSSQVPQSRNI	11	-0.41	0.62	0.62	-1.29	0.78	0.38	1.36	2	11.01	1243.54	0.99	0.42	0.07

895	SSQVPQSRNIC	11	-0.3	0.62	0.62	-0.71	0.45	0.02	1.18	1	8.6	1218.5	0.99	0.42	0.25
896	SQVPQSRNICD	11	-0.34	0.64	0.64	-0.95	0.45	0.26	1.18	0	6.16	1246.51	0.99	0.47	0.07
897	QVPQSRNICDI	11	-0.25	0.65	0.65	-0.47	0.45	0.07	1.09	0	6.16	1272.6	0.99	0.49	0.11
898	VPQSRNICDIS	11	-0.21	0.64	0.64	-0.23	0.34	0.08	1	0	6.16	1231.54	0.99	0.51	0.10
899	PQSRNICDISC	11	-0.26	0.63	0.63	-0.38	0.34	0.13	1	0	6.13	1235.54	0.94	0.44	0.94
900	QSRNICDISCD	11	-0.32	0.67	0.67	-0.55	0.34	0.4	1.09	-1	4.21	1253.51	0.99	0.49	0.52
901	SRNICDISCDK	11	-0.36	0.67	0.67	-0.59	0.56	0.65	1.09	0	6.25	1253.55	0.99	0.47	0.50
902	RNICDISCDKF	11	-0.28	0.68	0.68	-0.26	0.56	0.4	1	0	6.25	1313.65	0.99	0.50	0.84
903	NICDISCDKFL	11	-0.07	0.67	0.67	0.49	0.33	-0.04	0.64	-1	4.21	1270.63	0.99	0.68	0.81
904	ICDISCDKFLD	11	-0.08	0.67	0.67	0.49	0.33	0.22	0.55	-2	3.94	1271.61	0.99	0.48	0.59
905	CDISCDKFLDD	11	-0.21	0.67	0.67	-0.24	0.33	0.65	0.64	-3	3.77	1273.53	0.61	0.44	0.22
906	DISCDKFLDDD	11	-0.28	0.69	0.69	-0.78	0.33	1.02	0.73	-4	3.66	1285.48	0.61	0.44	0.18
907	ISCDKFLDDDI	11	-0.15	0.68	0.68	-0.05	0.33	0.58	0.64	-3	3.77	1283.56	0.99	0.44	0.20
908	SCDKFLDDDIT	11	-0.23	0.67	0.67	-0.53	0.33	0.71	0.73	-3	3.77	1271.5	0.99	0.49	0.27
909	CDKFLDDDITD	11	-0.27	0.69	0.69	-0.77	0.33	0.95	0.73	-4	3.66	1299.51	0.61	0.46	0.05
910	DKFLDDDITDD	11	-0.34	0.7	0.7	-1.32	0.33	1.32	0.82	-5	3.57	1311.46	0.61	0.45	0.12
911	KFLDDDITDDI	11	-0.21	0.69	0.69	-0.59	0.33	0.88	0.73	-4	3.66	1309.54	0.99	0.53	0.06
912	FLDDDITDDIM	11	-0.08	0.7	0.7	-0.06	0	0.49	0.55	-5	3.25	1312.56	0.61	0.48	0.04
913	LDDDITDDIMC	11	-0.14	0.7	0.7	-0.09	0	0.63	0.55	-5	3.25	1268.52	0.99	0.31	0.05
914	DDDITDDIMCA	11	-0.16	0.7	0.7	-0.27	0	0.75	0.55	-5	3.25	1226.43	0.61	0.52	0.05
915	DDITDDIMCAK	11	-0.2	0.69	0.69	-0.31	0.33	0.75	0.64	-3	3.77	1239.52	0.61	0.65	0.04
916	DITDDIMCAKK	11	-0.23	0.68	0.68	-0.35	0.67	0.75	0.73	-1	4.43	1252.61	0.86	0.72	0.14
917	ITDDIMCAKKI	11	-0.1	0.68	0.68	0.38	0.67	0.31	0.64	0	6.28	1250.69	0.99	0.74	0.49
918	TDDIMCAKKIL	11	-0.12	0.66	0.66	0.32	0.67	0.31	0.64	0	6.28	1250.69	0.99	0.82	0.79
919	DDIMCAKKILD	11	-0.17	0.68	0.68	0.06	0.67	0.62	0.64	-1	4.43	1264.67	0.61	0.73	0.38
920	DIMCAKKILDI	11	-0.03	0.68	0.68	0.79	0.67	0.18	0.55	0	6.28	1262.75	0.61	0.74	0.68
921	IMCAKKILDIK	11	-0.07	0.67	0.67	0.75	1	0.18	0.64	2	9.25	1275.84	0.99	0.72	0.97
922	MCAKKILDIKG	11	-0.12	0.67	0.67	0.31	1	0.35	0.64	2	9.25	1219.73	0.99	0.61	0.92
923	CAKKILDIKGI	11	-0.08	0.66	0.66	0.55	1	0.3	0.64	2	9.25	1201.7	0.83	0.55	0.89
924	AKKILDIKGID	11	-0.15	0.67	0.67	0	1	0.66	0.73	1	8.83	1213.65	0.61	0.52	0.81
925	KKILDIKGIDY	11	-0.17	0.69	0.69	-0.28	1	0.5	0.82	1	8.76	1305.75	0.99	0.51	0.41
926	KILDIKGIDYW	11	-0.03	0.67	0.67	-0.01	0.67	-0.08	0.73	0	6.3	1363.79	0.99	0.51	0.30
927	ILDIKGIDYWL	11	0.11	0.66	0.66	0.69	0.33	-0.52	0.55	-1	4.21	1348.78	0.99	0.47	0.33
928	LDIKGIDYWLA	11	0.07	0.64	0.64	0.45	0.33	-0.4	0.55	-1	4.21	1306.69	0.99	0.46	0.38
929	DIKGIDYWLAH	11	-0.01	0.59	0.59	-0.19	0.47	-0.28	0.64	-0.5	5.22	1330.67	0.86	0.53	0.13
930	IKGIDYWLAHK	11	-0.05	0.59	0.59	-0.23	0.8	-0.28	0.73	1.5	8.84	1343.76	0.99	0.54	0.78
931	KGIDYWLAHKA	11	-0.09	0.57	0.57	-0.47	0.8	-0.16	0.73	1.5	8.84	1301.67	0.99	0.65	0.94
932	GIDYWLAHKAL	11	0.06	0.56	0.56	0.23	0.47	-0.6	0.55	0.5	7.09	1286.66	0.98	0.80	0.87
933	IDYWLAHKALC	11	0.05	0.55	0.55	0.49	0.47	-0.69	0.55	0.5	7.06	1332.74	0.97	0.72	0.95
934	DYWLAHKALCT	11	-0.04	0.54	0.54	0.02	0.47	-0.56	0.64	0.5	7.06	1320.68	0.86	0.76	0.95
935	YWLAHKALCTE	11	-0.03	0.53	0.53	0.02	0.58	-0.56	0.64	0.5	7.06	1334.71	0.99	0.74	0.98
936	WLAHKALCTEK	11	-0.13	0.53	0.53	-0.22	0.91	-0.08	0.73	1.5	8.54	1299.71	0.99	0.66	0.96
937	LAHKALCTEKL	11	-0.12	0.53	0.53	0.21	0.91	0.06	0.64	1.5	8.54	1226.66	0.99	0.67	0.96
938	AHKALCTEKLE	11	-0.22	0.54	0.54	-0.45	1.03	0.5	0.73	0.5	7.07	1242.61	0.61	0.53	0.47
939	HKALCTEKLEQ	11	-0.31	0.56	0.56	-0.94	1.14	0.56	0.91	0.5	7.07	1299.67	0.99	0.46	0.18
940	KALCTEKLEQW	11	-0.24	0.6	0.6	-0.73	1.01	0.3	0.91	0	6.46	1348.74	0.99	0.47	0.49
941	ALCTEKLEQWL	11	-0.09	0.59	0.59	-0.03	0.68	-0.14	0.73	-1	4.54	1333.73	0.61	0.25	0.76
942	LCTEKLEQWLC	11	-0.11	0.6	0.6	0.04	0.68	-0.18	0.73	-1	4.54	1365.79	0.99	0.19	0.76
943	CTEKLEQWLCE	11	-0.21	0.61	0.61	-0.63	0.79	0.25	0.82	-2	4.26	1381.74	0.61	0.18	0.13
944	TEKLEQWLCEK	11	-0.31	0.62	0.62	-1.21	1.13	0.62	1	-1	4.79	1406.78	0.99	0.16	0.07
945	EKLEQWLCEKL	11	-0.25	0.62	0.62	-0.8	1.13	0.49	0.91	-1	4.79	1418.84	0.61	0.20	0.27
946	MRFFVPLFLVGI	12	0.27	0.65	0.65	2.02	0.2	-1.18	0.33	1	10.11	1439.02	0.93	0.68	0.93
947	RFFVPLFLVGIL	12	0.3	0.63	0.63	2.18	0.2	-1.23	0.33	1	10.11	1420.99	0.98	0.73	0.99
948	FFVPLFLVGILF	12	0.49	0.63	0.63	2.79	0	-1.68	0	0	5.88	1411.98	0.94	0.71	0.98
949	FVPLFLVGILFP	12	0.44	0.6	0.6	2.43	0	-1.48	0	0	5.88	1361.92	0.94	0.69	0.99
950	VPLFLVGILFPA	12	0.41	0.58	0.58	2.34	0	-1.31	0	0	5.88	1285.82	0.98	0.69	0.82
951	PLFLVGILFPAI	12	0.42	0.58	0.58	2.37	0	-1.33	0	0	5.88	1299.85	0.98	0.67	0.92
952	LFLVGILFPAIL	12	0.47	0.6	0.6	2.82	0	-1.48	0	0	5.88	1315.9	0.93	0.62	0.78
953	FLVGILFPAILA	12	0.45	0.6	0.6	2.65	0	-1.38	0	0	5.88	1273.81	0.94	0.69	0.94
954	LVGILFPAILAK	12	0.31	0.6	0.6	2.09	0.31	-0.92	0.17	1	9.11	1254.81	0.98	0.67	0.97
955	VGILFPAILAKQ	12	0.21	0.61	0.61	1.48	0.41	-0.75	0.33	1	9.11	1269.78	0.98	0.68	0.99
956	GILFPAILAKQF	12	0.21	0.61	0.61	1.37	0.41	-0.83	0.33	1	9.11	1317.82	0.95	0.71	0.98
957	ILFPAILAKQFT	12	0.18	0.6	0.6	1.34	0.41	-0.87	0.42	1	9.11	1361.87	0.98	0.72	0.97
958	LFPAILAKQFTK	12	0.03	0.59	0.59	0.64	0.72	-0.47	0.58	2	10.02	1376.88	0.98	0.72	0.98

959	FPAILAKQFTKC	12	-0.01	0.6	0.6	0.53	0.72	-0.4	0.58	2	9.36	1366.85	0.94	0.72	0.98
960	PAILAKQFTKCE	12	-0.11	0.6	0.6	0.01	0.82	0.06	0.67	1	8.54	1348.79	0.93	0.69	0.93
961	AILAKQFTKCEL	12	-0.06	0.61	0.61	0.46	0.82	-0.09	0.67	1	8.54	1364.84	0.61	0.58	0.77
962	ILAKQFTKCELS	12	-0.11	0.62	0.62	0.24	0.82	-0.02	0.75	1	8.54	1380.84	0.99	0.61	0.50
963	LAKQFTKCELSQ	12	-0.22	0.61	0.61	-0.43	0.93	0.14	0.92	1	8.54	1395.81	0.99	0.57	0.80
964	AKQFTKCELSQL	12	-0.22	0.61	0.61	-0.43	0.93	0.14	0.92	1	8.54	1395.81	0.61	0.44	0.28
965	KQFTKCELSQLL	12	-0.2	0.61	0.61	-0.26	0.93	0.03	0.92	1	8.54	1437.9	0.99	0.38	0.59
966	QFTKCELSQLLK	12	-0.2	0.61	0.61	-0.26	0.93	0.03	0.92	1	8.54	1437.9	0.94	0.36	0.42
967	FTKCELSQLLKD	12	-0.2	0.62	0.62	-0.26	0.82	0.27	0.83	0	6.38	1424.85	0.61	0.31	0.73
968	TKCELSQLLKDI	12	-0.19	0.62	0.62	-0.12	0.82	0.32	0.83	0	6.38	1390.84	0.99	0.35	0.24
969	KCELSQLLKDID	12	-0.24	0.64	0.64	-0.35	0.82	0.61	0.83	-1	4.56	1404.82	0.99	0.33	0.33
970	CELSQLLKDIDG	12	-0.13	0.64	0.64	-0.06	0.52	0.36	0.67	-2	4.03	1333.7	0.61	0.32	0.05
971	ELSQLLKDIDGY	12	-0.13	0.65	0.65	-0.38	0.52	0.25	0.75	-2	4.03	1393.74	0.61	0.29	0.04
972	LSQLLKDIDGYG	12	-0.07	0.65	0.65	-0.12	0.41	0	0.67	-1	4.21	1321.68	0.99	0.31	0.13
973	SQLLKDIDGYGG	12	-0.1	0.66	0.66	-0.47	0.41	0.15	0.67	-1	4.21	1265.57	0.99	0.31	0.10
974	QLLKDIDGYGGI	12	-0.02	0.67	0.67	-0.03	0.41	-0.03	0.58	-1	4.21	1291.66	0.94	0.30	0.05
975	LLKDIDGYGGIA	12	0.06	0.66	0.66	0.42	0.31	-0.08	0.42	-1	4.21	1234.6	0.99	0.42	0.24
976	LKDIDGYGGIAL	12	0.06	0.66	0.66	0.42	0.31	-0.08	0.42	-1	4.21	1234.6	0.99	0.49	0.12
977	KDIDGYGGIALP	12	0.01	0.65	0.65	-0.03	0.31	0.07	0.42	-1	4.21	1218.55	0.99	0.57	0.28
978	DIDGYGGIALPE	12	0.05	0.65	0.65	0	0.11	0.07	0.33	-3	3.5	1219.49	0.61	0.63	0.13
979	IDGYGGIALPEL	12	0.15	0.63	0.63	0.61	0.11	-0.33	0.25	-2	3.67	1217.57	0.99	0.65	0.20
980	DGYGGIALPELI	12	0.15	0.63	0.63	0.61	0.11	-0.33	0.25	-2	3.67	1217.57	0.86	0.67	0.17
981	GYGGIALPELIC	12	0.22	0.62	0.62	1.11	0.11	-0.67	0.17	-1	4	1205.62	0.98	0.64	0.85
982	YGGIALPELICT	12	0.19	0.6	0.6	1.08	0.11	-0.7	0.25	-1	4	1249.67	0.99	0.66	0.59
983	GGIALPELICTM	12	0.21	0.61	0.61	1.35	0.11	-0.62	0.17	-1	4	1217.69	0.98	0.64	0.81
984	GIALPELICTMF	12	0.25	0.61	0.61	1.62	0.11	-0.82	0.17	-1	4	1307.81	0.98	0.68	0.59
985	IALPELICTMFH	12	0.2	0.55	0.55	1.38	0.23	-0.87	0.25	-0.5	5.25	1387.9	0.97	0.72	0.38
986	ALPELICTMFHT	12	0.13	0.54	0.54	0.95	0.23	-0.75	0.33	-0.5	5.25	1375.84	0.61	0.66	0.20
987	LPELICTMFHTS	12	0.08	0.54	0.54	0.73	0.23	-0.68	0.42	-0.5	5.25	1391.84	0.99	0.56	0.07
988	PELICTMFHTSG	12	0.05	0.55	0.55	0.38	0.23	-0.53	0.42	-0.5	5.25	1335.73	0.94	0.40	0.06
989	ELICTMFHTSGY	12	0.06	0.58	0.58	0.41	0.23	-0.72	0.5	-0.5	5.25	1401.79	0.61	0.36	0.10
990	LICTMFHTSGYD	12	0.05	0.59	0.59	0.41	0.12	-0.72	0.5	-0.5	5.09	1387.76	0.98	0.40	0.21
991	ICTMFHTSGYDT	12	-0.01	0.59	0.59	0.03	0.12	-0.61	0.58	-0.5	5.09	1375.7	0.98	0.39	0.41
992	CTMFHTSGYDTQ	12	-0.13	0.59	0.59	-0.63	0.23	-0.44	0.75	-0.5	5.09	1390.67	0.94	0.37	0.17
993	TMFHTSGYDTQA	12	-0.11	0.58	0.58	-0.69	0.23	-0.4	0.75	-0.5	5.09	1358.61	0.98	0.38	0.13
994	MFHTSGYDTQAI	12	-0.03	0.59	0.59	-0.26	0.23	-0.52	0.67	-0.5	5.09	1370.67	0.98	0.29	0.12
995	FHTSGYDTQAIV	12	-0.01	0.59	0.59	-0.07	0.23	-0.53	0.67	-0.5	5.09	1338.61	0.80	0.34	0.11
996	HTSGYDTQAIVE	12	-0.11	0.58	0.58	-0.59	0.33	-0.07	0.75	-1.5	4.36	1320.55	0.98	0.34	0.06
997	TSGYDTQAIVEN	12	-0.13	0.65	0.65	-0.62	0.21	-0.02	0.83	-2	3.67	1297.51	0.93	0.33	0.06
998	SGYDTQAIVENN	12	-0.17	0.67	0.67	-0.85	0.21	0.03	0.92	-2	3.67	1310.51	0.93	0.34	0.04
999	GYDTQAIVENNE	12	-0.2	0.68	0.68	-1.07	0.32	0.26	0.92	-3	3.58	1352.55	0.93	0.34	0.03
1000	YDTQAIVENNES	12	-0.24	0.67	0.67	-1.11	0.32	0.28	1	-3	3.58	1382.57	0.99	0.37	0.04
1001	DTQAIVENNEST	12	-0.25	0.65	0.65	-1.06	0.32	0.44	1	-3	3.58	1320.5	0.61	0.37	0.14
1002	TQAIVENNESTE	12	-0.24	0.65	0.65	-1.06	0.42	0.44	1	-3	3.68	1334.53	0.99	0.41	0.10
1003	QAIVENNESTEY	12	-0.23	0.66	0.66	-1.11	0.42	0.28	1	-3	3.68	1396.6	0.99	0.43	0.05
1004	AIVENNESTEYG	12	-0.16	0.66	0.66	-0.85	0.32	0.27	0.83	-3	3.68	1325.52	0.61	0.47	0.04
1005	IVENNESTEYGL	12	-0.13	0.66	0.66	-0.68	0.32	0.16	0.83	-3	3.68	1367.61	0.94	0.44	0.08
1006	VENNESTEYGLF	12	-0.14	0.66	0.66	-0.82	0.32	0.1	0.83	-3	3.68	1401.62	0.99	0.46	0.09
1007	ENNESTEYGLFQ	12	-0.25	0.66	0.66	-1.47	0.42	0.24	1	-3	3.68	1430.62	0.61	0.42	0.13
1008	NNESTEYGLFQI	12	-0.13	0.66	0.66	-0.8	0.32	-0.16	0.92	-2	3.8	1414.67	0.94	0.45	0.10
1009	NESTEYGLFQIS	12	-0.1	0.64	0.64	-0.58	0.32	-0.15	0.83	-2	3.8	1387.64	0.99	0.47	0.16
1010	ESTEYGLFQISN	12	-0.1	0.64	0.64	-0.58	0.32	-0.15	0.83	-2	3.8	1387.64	0.61	0.48	0.09
1011	STEYGLFQISNK	12	-0.14	0.64	0.64	-0.61	0.52	-0.15	0.92	0	6.35	1386.7	0.99	0.49	0.25
1012	TEYGLFQISNKL	12	-0.08	0.64	0.64	-0.23	0.52	-0.32	0.83	0	6.35	1412.79	0.99	0.60	0.22
1013	EYGLFQISNKLW	12	-0.03	0.64	0.64	-0.24	0.52	-0.57	0.83	0	6.35	1497.9	0.61	0.62	0.54
1014	YGLFQISNKLWC	12	0.02	0.63	0.63	0.26	0.41	-0.91	0.75	1	8.54	1471.92	0.99	0.64	0.97
1015	GLFQISNKLWCK	12	-0.07	0.63	0.63	0.04	0.72	-0.47	0.83	2	9.36	1436.92	0.98	0.60	0.98
1016	LFQISNKLWCKS	12	-0.1	0.62	0.62	0.01	0.72	-0.44	0.92	2	9.36	1466.94	0.94	0.61	0.97
1017	FQISNKLWCKSS	12	-0.17	0.62	0.62	-0.38	0.72	-0.27	1	2	9.36	1440.85	0.61	0.59	0.90
1018	QISNKLWCKSSQ	12	-0.28	0.62	0.62	-0.9	0.82	-0.04	1.17	2	9.36	1421.81	0.99	0.62	0.38
1019	ISNKLWCKSSQV	12	-0.18	0.62	0.62	-0.26	0.72	-0.18	1	2	9.36	1392.81	0.99	0.63	0.20
1020	SNKLWCKSSQVP	12	-0.24	0.59	0.59	-0.77	0.72	-0.03	1	2	9.36	1376.76	0.99	0.56	0.30
1021	NKLWCKSSQVPQ	12	-0.28	0.6	0.6	-0.99	0.82	-0.04	1.08	2	9.36	1417.82	0.99	0.54	0.88
1022	KLWCKSSQVPQS	12	-0.25	0.59	0.59	-0.77	0.82	-0.03	1	2	9.36	1390.79	0.99	0.54	0.75

1023	LWCKSSQVPQSR	12	-0.3	0.59	0.59	-0.82	0.72	-0.03	1.17	2	9.55	1418.8	0.99	0.43	0.51
1024	WCKSSQVPQSRN	12	-0.4	0.6	0.6	-1.43	0.72	0.13	1.33	2	9.55	1419.74	0.99	0.43	0.35
1025	CKSSQVPQSRNI	12	-0.37	0.62	0.62	-0.98	0.72	0.27	1.25	2	9.55	1346.69	0.86	0.39	0.06
1026	KSSQVPQSRNIC	12	-0.37	0.62	0.62	-0.98	0.72	0.27	1.25	2	9.55	1346.69	0.99	0.44	0.25
1027	SSQVPQSRNICD	12	-0.34	0.63	0.63	-0.94	0.41	0.27	1.17	0	6.16	1333.6	0.99	0.47	0.06
1028	SQVPQSRNICDI	12	-0.25	0.64	0.64	-0.5	0.41	0.09	1.08	0	6.16	1359.69	0.99	0.50	0.09
1029	QVPQSRNICDIS	12	-0.25	0.64	0.64	-0.5	0.41	0.09	1.08	0	6.16	1359.69	0.99	0.53	0.07
1030	VPQSRNICDISC	12	-0.19	0.64	0.64	0	0.31	-0.01	0.92	0	6.13	1334.69	0.99	0.48	0.95
1031	PQSRNICDISCD	12	-0.3	0.64	0.64	-0.64	0.31	0.37	1	-1	4.21	1350.64	0.94	0.43	0.60
1032	QSRNICDISCDK	12	-0.38	0.67	0.67	-0.83	0.61	0.62	1.17	0	6.25	1381.7	0.99	0.44	0.33
1033	SRNICDISCDKF	12	-0.28	0.67	0.67	-0.31	0.51	0.39	1	0	6.25	1400.74	0.99	0.51	0.63
1034	RNICDISCDKFL	12	-0.21	0.67	0.67	0.07	0.51	0.22	0.92	0	6.25	1426.83	0.99	0.66	0.89
1035	NICDISCDKFLD	12	-0.12	0.68	0.68	0.16	0.31	0.22	0.67	-2	3.94	1385.73	0.99	0.46	0.53
1036	ICDISCDKFLDD	12	-0.13	0.68	0.68	0.16	0.31	0.45	0.58	-3	3.77	1386.71	0.99	0.43	0.32
1037	CDISCDKFLDDD	12	-0.25	0.68	0.68	-0.51	0.31	0.85	0.67	-4	3.66	1388.63	0.61	0.42	0.18
1038	DISCDKFLDDDI	12	-0.19	0.69	0.69	-0.34	0.31	0.78	0.67	-4	3.66	1398.66	0.61	0.42	0.13
1039	ISCDKFLDDDIT	12	-0.15	0.67	0.67	-0.11	0.31	0.5	0.67	-3	3.77	1384.68	0.99	0.44	0.10
1040	SCDKFLDDDITD	12	-0.27	0.67	0.67	-0.78	0.31	0.9	0.75	-4	3.66	1386.6	0.99	0.44	0.19
1041	CDKFLDDDITDD	12	-0.31	0.69	0.69	-1	0.31	1.12	0.75	-5	3.57	1414.61	0.61	0.44	0.06
1042	DKFLDDDITDDI	12	-0.25	0.7	0.7	-0.83	0.31	1.06	0.75	-5	3.57	1424.64	0.61	0.43	0.07
1043	KFLDDDITDDIM	12	-0.17	0.7	0.7	-0.38	0.31	0.7	0.67	-4	3.66	1440.75	0.99	0.50	0.03
1044	FLDDDITDDIMC	12	-0.07	0.7	0.7	0.15	0	0.37	0.5	-5	3.25	1415.71	0.61	0.49	0.05
1045	LDDDITDDIMCA	12	-0.1	0.68	0.68	0.07	0	0.53	0.5	-5	3.25	1339.61	0.99	0.32	0.03
1046	DDDITDDIMCAK	12	-0.24	0.69	0.69	-0.57	0.31	0.93	0.67	-4	3.66	1354.62	0.61	0.64	0.03
1047	DDITDDIMCAKK	12	-0.27	0.69	0.69	-0.61	0.61	0.93	0.75	-2	4.14	1367.71	0.61	0.70	0.10
1048	DITDDIMCAKKI	12	-0.15	0.68	0.68	0.06	0.61	0.53	0.67	-1	4.43	1365.79	0.86	0.71	0.23
1049	ITDDIMCAKKIL	12	-0.05	0.66	0.66	0.67	0.61	0.13	0.58	0	6.28	1363.87	0.99	0.77	0.95
1050	TDDIMCAKKILD	12	-0.17	0.67	0.67	0	0.61	0.53	0.67	-1	4.43	1365.79	0.99	0.66	0.16
1051	DDIMCAKKILDI	12	-0.09	0.68	0.68	0.43	0.61	0.42	0.58	-1	4.43	1377.85	0.61	0.69	0.46
1052	DIMCAKKILDIK	12	-0.12	0.68	0.68	0.4	0.92	0.42	0.67	1	8.51	1390.94	0.61	0.70	0.93
1053	IMCAKKILDIKG	12	-0.05	0.67	0.67	0.66	0.92	0.17	0.58	2	9.25	1332.91	0.99	0.57	0.96
1054	MCAKKILDIKGI	12	-0.05	0.67	0.67	0.66	0.92	0.17	0.58	2	9.25	1332.91	0.99	0.52	0.93
1055	CAKKILDIKGID	12	-0.13	0.67	0.67	0.21	0.92	0.53	0.67	1	8.51	1316.8	0.83	0.51	0.96
1056	AKKILDIKGIDY	12	-0.13	0.67	0.67	-0.11	0.92	0.42	0.75	1	8.76	1376.84	0.61	0.51	0.79
1057	KKILDIKGIDYW	12	-0.12	0.67	0.67	-0.33	0.92	0.18	0.83	1	8.76	1491.98	0.99	0.54	0.54
1058	KILDIKGIDYWL	12	0.01	0.66	0.66	0.31	0.61	-0.23	0.67	0	6.3	1476.97	0.99	0.49	0.44
1059	ILDIKGIDYWLA	12	0.13	0.65	0.65	0.78	0.31	-0.52	0.5	-1	4.21	1419.87	0.99	0.49	0.45
1060	LDIKGIDYWLAH	12	0.03	0.59	0.59	0.14	0.43	-0.41	0.58	-0.5	5.22	1443.85	0.99	0.45	0.25
1061	DIKGIDYWLAHK	12	-0.1	0.6	0.6	-0.5	0.73	-0.01	0.75	0.5	7.09	1458.86	0.86	0.53	0.27
1062	IKGIDYWLAHKA	12	-0.02	0.58	0.58	-0.06	0.73	-0.3	0.67	1.5	8.84	1414.85	0.97	0.59	0.87
1063	KGIDYWLAHKAL	12	-0.04	0.57	0.57	-0.12	0.73	-0.3	0.67	1.5	8.84	1414.85	0.99	0.64	0.89
1064	GIDYWLAHKALC	12	0.06	0.56	0.56	0.42	0.43	-0.63	0.5	0.5	7.06	1389.81	0.98	0.68	0.93
1065	IDYWLAHKALCT	12	0.03	0.55	0.55	0.39	0.43	-0.67	0.58	0.5	7.06	1433.86	0.97	0.71	0.92
1066	DYWLAHKALCTE	12	-0.09	0.55	0.55	-0.28	0.53	-0.27	0.67	-0.5	5.33	1449.81	0.86	0.72	0.98
1067	YWLAHKALCTEK	12	-0.12	0.54	0.54	-0.31	0.84	-0.27	0.75	1.5	8.51	1462.9	0.99	0.67	0.95
1068	WLAHKALCTEKL	12	-0.08	0.53	0.53	0.12	0.84	-0.23	0.67	1.5	8.54	1412.89	0.99	0.59	0.99
1069	LAHKALCTEKLE	12	-0.16	0.54	0.54	-0.1	0.94	0.31	0.67	0.5	7.07	1355.79	0.99	0.62	0.69
1070	AHKALCTEKLEQ	12	-0.26	0.55	0.55	-0.71	1.05	0.48	0.83	0.5	7.07	1370.76	0.61	0.47	0.32
1071	HKALCTEKLEQW	12	-0.25	0.55	0.55	-0.93	1.05	0.23	0.92	0.5	7.07	1485.9	0.99	0.42	0.43
1072	KALCTEKLEQWL	12	-0.17	0.6	0.6	-0.35	0.93	0.13	0.83	0	6.46	1461.92	0.99	0.39	0.70
1073	ALCTEKLEQWLC	12	-0.08	0.59	0.59	0.18	0.62	-0.21	0.67	-1	4.54	1436.88	0.61	0.26	0.75
1074	LCTEKLEQWLCE	12	-0.15	0.6	0.6	-0.26	0.73	0.08	0.75	-2	4.26	1494.92	0.99	0.19	0.54
1075	CTEKLEQWLCEK	12	-0.28	0.62	0.62	-0.9	1.03	0.48	0.92	-1	4.79	1509.93	0.86	0.18	0.07
1076	TEKLEQWLCEKL	12	-0.24	0.61	0.61	-0.79	1.03	0.42	0.92	-1	4.79	1519.96	0.99	0.20	0.30
1077	MRFFVPLFLVGIL	13	0.29	0.64	0.64	2.16	0.19	-1.23	0.31	1	10.11	1552.2	0.93	0.66	0.99
1078	RFFVPLFLVGILF	13	0.32	0.63	0.63	2.23	0.19	-1.32	0.31	1	10.11	1568.18	0.93	0.67	0.95
1079	FFVPLFLVGILFP	13	0.45	0.61	0.61	2.45	0	-1.55	0	0	5.88	1509.11	0.94	0.68	0.98
1080	FVPLFLVGILFPA	13	0.42	0.59	0.59	2.38	0	-1.4	0	0	5.88	1433.01	0.94	0.71	0.97
1081	VPLFLVGILFPAI	13	0.43	0.59	0.59	2.51	0	-1.35	0	0	5.88	1399	0.98	0.66	0.79
1082	PLFLVGILFPAIL	13	0.43	0.58	0.58	2.48	0	-1.37	0	0	5.88	1413.03	0.98	0.66	0.87
1083	LFLVGILFPAILA	13	0.46	0.59	0.59	2.74	0	-1.41	0	0	5.88	1386.99	0.98	0.66	0.71
1084	FLVGILFPAILAK	13	0.33	0.6	0.6	2.15	0.28	-1.04	0.15	1	9.11	1402	0.94	0.74	0.98
1085	LVGILFPAILAKQ	13	0.23	0.6	0.6	1.66	0.38	-0.83	0.31	1	9.11	1382.96	0.98	0.59	0.94
1086	VGILFPAILAKQF	13	0.24	0.62	0.62	1.58	0.38	-0.88	0.31	1	9.11	1416.97	0.98	0.66	0.98

1087	GILFPAILAKQFT	13	0.18	0.6	0.6	1.21	0.38	-0.8	0.38	1	9.11	1418.94	0.95	0.66	0.99
1088	ILFPAILAKQFTK	13	0.08	0.6	0.6	0.94	0.66	-0.57	0.54	2	10.02	1490.06	0.98	0.69	0.99
1089	LFPAILAKQFTKC	13	0.03	0.6	0.6	0.78	0.66	-0.51	0.54	2	9.36	1480.03	0.93	0.67	0.98
1090	FPAILAKQFTKCE	13	-0.06	0.61	0.61	0.22	0.76	-0.14	0.62	1	8.54	1495.98	0.94	0.66	0.99
1091	PAILAKQFTKCEL	13	-0.06	0.59	0.59	0.3	0.76	-0.08	0.62	1	8.54	1461.97	0.98	0.58	0.79
1092	AILAKQFTKCELS	13	-0.08	0.61	0.61	0.36	0.76	-0.06	0.69	1	8.54	1451.93	0.61	0.53	0.80
1093	ILAKQFTKCELSQ	13	-0.15	0.62	0.62	-0.05	0.85	-0.01	0.85	1	8.54	1508.99	0.99	0.58	0.41
1094	LAKQFTKCELSQL	13	-0.17	0.61	0.61	-0.1	0.85	-0.01	0.85	1	8.54	1508.99	0.99	0.49	0.71
1095	AKQFTKCELSQLL	13	-0.17	0.61	0.61	-0.1	0.85	-0.01	0.85	1	8.54	1508.99	0.61	0.47	0.72
1096	KQFTKCELSQLLK	13	-0.27	0.62	0.62	-0.54	1.14	0.26	1	2	9.25	1566.09	0.99	0.41	0.80
1097	QFTKCELSQLLKD	13	-0.24	0.63	0.63	-0.51	0.85	0.26	0.92	0	6.38	1553	0.94	0.37	0.28
1098	FTKCELSQLLKDI	13	-0.13	0.63	0.63	0.11	0.76	0.11	0.77	0	6.38	1538.03	0.61	0.34	0.41
1099	TKCELSQLLKDID	13	-0.23	0.63	0.63	-0.38	0.76	0.53	0.85	-1	4.56	1505.94	0.99	0.39	0.15
1100	KCELSQLLKDIDG	13	-0.21	0.64	0.64	-0.35	0.76	0.56	0.77	-1	4.56	1461.89	0.99	0.36	0.07
1101	CELSQLLKDIDGY	13	-0.12	0.64	0.64	-0.15	0.48	0.15	0.69	-2	4.03	1496.89	0.61	0.31	0.05
1102	ELSQLLKDIDGYG	13	-0.11	0.65	0.65	-0.38	0.48	0.23	0.69	-2	4.03	1450.81	0.61	0.33	0.03
1103	LSQLLKDIDGYGG	13	-0.05	0.65	0.65	-0.14	0.38	0	0.62	-1	4.21	1378.75	0.99	0.34	0.16
1104	SQLLKDIDGYGGI	13	-0.04	0.66	0.66	-0.08	0.38	0	0.62	-1	4.21	1378.75	0.99	0.34	0.08
1105	QLLKDIDGYGGIA	13	0	0.66	0.66	0.12	0.38	-0.06	0.54	-1	4.21	1362.75	0.94	0.37	0.07
1106	LLKDIDGYGGIAL	13	0.1	0.65	0.65	0.68	0.28	-0.22	0.38	-1	4.21	1347.78	0.99	0.53	0.51
1107	LKDIDGYGGIALP	13	0.05	0.64	0.64	0.26	0.28	-0.08	0.38	-1	4.21	1331.73	0.99	0.56	0.33
1108	KDIDGYGGIALPE	13	-0.04	0.65	0.65	-0.3	0.38	0.29	0.46	-2	4.03	1347.68	0.99	0.58	0.06
1109	DIDGYGGIALPEL	13	0.09	0.64	0.64	0.29	0.1	-0.08	0.31	-3	3.5	1332.67	0.61	0.62	0.12
1110	IDGYGGIALPELI	13	0.2	0.63	0.63	0.91	0.1	-0.45	0.23	-2	3.67	1330.75	0.97	0.63	0.24
1111	DGYGGIALPELIC	13	0.15	0.63	0.63	0.75	0.1	-0.38	0.23	-2	3.67	1320.72	0.86	0.63	0.29
1112	GYGGIALPELICT	13	0.19	0.61	0.61	0.97	0.1	-0.65	0.23	-1	4	1306.74	0.98	0.61	0.76
1113	YGGIALPELICTM	13	0.2	0.62	0.62	1.15	0.1	-0.75	0.23	-1	4	1380.88	0.99	0.64	0.56
1114	GGIALPELICTMF	13	0.24	0.62	0.62	1.46	0.1	-0.76	0.15	-1	4	1364.88	0.98	0.61	0.71
1115	GIALPELICTMFH	13	0.2	0.56	0.56	1.25	0.21	-0.8	0.23	-0.5	5.25	1444.97	0.98	0.70	0.55
1116	IALPELICTMFHT	13	0.17	0.55	0.55	1.22	0.21	-0.83	0.31	-0.5	5.25	1489.02	0.97	0.66	0.16
1117	ALPELICTMFHTS	13	0.1	0.54	0.54	0.82	0.21	-0.67	0.38	-0.5	5.25	1462.93	0.61	0.54	0.19
1118	LPELICTMFHTSG	13	0.09	0.55	0.55	0.65	0.21	-0.63	0.38	-0.5	5.25	1448.91	0.99	0.43	0.08
1119	PELICTMFHTSGY	13	0.05	0.56	0.56	0.25	0.21	-0.67	0.46	-0.5	5.25	1498.92	0.94	0.33	0.06
1120	ELICTMFHTSGYD	13	0	0.6	0.6	0.11	0.21	-0.44	0.54	-1.5	4.36	1516.89	0.80	0.33	0.05
1121	LICTMFHTSGYDT	13	0.03	0.58	0.58	0.32	0.11	-0.7	0.54	-0.5	5.09	1488.88	0.98	0.37	0.10
1122	ICTMFHTSGYDTQ	13	-0.06	0.6	0.6	-0.24	0.21	-0.55	0.69	-0.5	5.09	1503.85	0.93	0.39	0.38
1123	CTMFHTSGYDTQA	13	-0.1	0.58	0.58	-0.45	0.21	-0.45	0.69	-0.5	5.09	1461.76	0.94	0.38	0.17
1124	TMFHTSGYDTQAI	13	-0.04	0.59	0.59	-0.29	0.21	-0.51	0.69	-0.5	5.09	1471.79	0.98	0.32	0.12
1125	MFHTSGYDTQAIV	13	0.01	0.6	0.6	0.08	0.21	-0.59	0.62	-0.5	5.09	1469.82	0.98	0.35	0.12
1126	FHTSGYDTQAIVE	13	-0.06	0.59	0.59	-0.33	0.31	-0.26	0.69	-1.5	4.36	1467.74	0.94	0.38	0.13
1127	HTSGYDTQAIVEN	13	-0.15	0.6	0.6	-0.82	0.31	-0.05	0.85	-1.5	4.36	1434.67	0.98	0.37	0.05
1128	TSGYDTQAIVENN	13	-0.17	0.66	0.66	-0.84	0.19	0	0.92	-2	3.67	1411.63	0.93	0.32	0.05
1129	SGYDTQAIVENNE	13	-0.21	0.67	0.67	-1.05	0.29	0.26	0.92	-3	3.58	1439.64	0.93	0.32	0.04
1130	GYDTQAIVENNES	13	-0.21	0.67	0.67	-1.05	0.29	0.26	0.92	-3	3.58	1439.64	0.83	0.33	0.03
1131	YDTQAIVENNEST	13	-0.23	0.66	0.66	-1.08	0.29	0.23	1	-3	3.58	1483.69	0.99	0.35	0.10
1132	DTQAIVENNESTE	13	-0.28	0.65	0.65	-1.25	0.39	0.64	1	-4	3.51	1449.63	0.61	0.38	0.06
1133	TQAIVENNESTEY	13	-0.22	0.65	0.65	-1.08	0.39	0.23	1	-3	3.68	1497.72	0.99	0.43	0.09
1134	QAIVENNESTEYG	13	-0.2	0.66	0.66	-1.05	0.39	0.26	0.92	-3	3.68	1453.67	0.99	0.42	0.04
1135	AIVENNESTEYGL	13	-0.1	0.65	0.65	-0.49	0.29	0.11	0.77	-3	3.68	1438.7	0.61	0.42	0.07
1136	IVENNESTEYGLF	13	-0.08	0.66	0.66	-0.42	0.29	-0.05	0.77	-3	3.68	1514.8	0.94	0.48	0.09
1137	VENNESTEYGLFQ	13	-0.19	0.66	0.66	-1.03	0.39	0.11	0.92	-3	3.68	1529.77	0.99	0.43	0.09
1138	ENNESTEYGLFQI	13	-0.17	0.66	0.66	-1.01	0.39	0.08	0.92	-3	3.68	1543.8	0.61	0.44	0.13
1139	NNESTEYGLFQIS	13	-0.14	0.65	0.65	-0.8	0.29	-0.12	0.92	-2	3.8	1501.76	0.94	0.45	0.17
1140	NESTEYGLFQISN	13	-0.14	0.65	0.65	-0.8	0.29	-0.12	0.92	-2	3.8	1501.76	0.99	0.42	0.06
1141	ESTEYGLFQISNK	13	-0.18	0.64	0.64	-0.83	0.57	0.09	0.92	-1	4.54	1515.83	0.61	0.48	0.10
1142	STEYGLFQISNKL	13	-0.09	0.63	0.63	-0.27	0.48	-0.28	0.85	0	6.35	1499.88	0.99	0.54	0.35
1143	TEYGLFQISNKLW	13	-0.04	0.63	0.63	-0.28	0.48	-0.56	0.85	0	6.35	1599.02	0.99	0.56	0.20
1144	EYGLFQISNKLWC	13	-0.02	0.64	0.64	-0.03	0.48	-0.61	0.77	0	6.32	1601.05	0.61	0.61	0.75
1145	YGLFQISNKLWCK	13	-0.06	0.64	0.64	-0.06	0.66	-0.61	0.85	2	9.25	1600.11	0.99	0.57	0.98
1146	GLFQISNKLWCKS	13	-0.08	0.62	0.62	-0.02	0.66	-0.41	0.85	2	9.36	1524.01	0.98	0.59	0.98
1147	LFQISNKLWCKSS	13	-0.12	0.61	0.61	-0.05	0.66	-0.38	0.92	2	9.36	1554.03	0.94	0.62	0.94
1148	FQISNKLWCKSSQ	13	-0.21	0.62	0.62	-0.62	0.76	-0.23	1.08	2	9.36	1569	0.61	0.58	0.71
1149	QISNKLWCKSSQV	13	-0.21	0.62	0.62	-0.51	0.76	-0.15	1.08	2	9.36	1520.96	0.99	0.63	0.10
1150	ISNKLWCKSSQVP	13	-0.17	0.6	0.6	-0.36	0.66	-0.17	0.92	2	9.36	1489.94	0.99	0.63	0.19

1151	SNKLWCKSSQVPQ	13	-0.28	0.6	0.6	-0.98	0.76	-0.02	1.08	2	9.36	1504.91	0.99	0.52	0.18
1152	NKLWCKSSQVPQS	13	-0.28	0.6	0.6	-0.98	0.76	-0.02	1.08	2	9.36	1504.91	0.99	0.51	0.43
1153	KLWCKSSQVPQSR	13	-0.36	0.59	0.59	-1.05	0.95	0.2	1.23	3	10.07	1546.99	0.99	0.48	0.80
1154	LWCKSSQVPQSRN	13	-0.33	0.6	0.6	-1.02	0.66	-0.02	1.23	2	9.55	1532.92	0.99	0.42	0.41
1155	WCKSSQVPQSRNI	13	-0.31	0.61	0.61	-0.97	0.66	-0.02	1.23	2	9.55	1532.92	0.97	0.41	0.19
1156	CKSSQVPQSRNIC	13	-0.34	0.62	0.62	-0.71	0.66	0.17	1.15	2	9.03	1449.84	0.86	0.40	0.56
1157	KSSQVPQSRNICD	13	-0.4	0.63	0.63	-1.17	0.66	0.48	1.23	1	8.57	1461.79	0.99	0.48	0.06
1158	SSQVPQSRNICDI	13	-0.25	0.63	0.63	-0.52	0.38	0.11	1.08	0	6.16	1446.78	0.99	0.49	0.06
1159	SQVPQSRNICDIS	13	-0.25	0.63	0.63	-0.52	0.38	0.11	1.08	0	6.16	1446.78	0.99	0.53	0.13
1160	QVPQSRNICDISC	13	-0.23	0.64	0.64	-0.27	0.38	0.01	1	0	6.13	1462.84	0.99	0.51	0.62
1161	VPQSRNICDISCD	13	-0.23	0.65	0.65	-0.27	0.28	0.22	0.92	-1	4.21	1449.79	0.99	0.48	0.50
1162	PQSRNICDISCDK	13	-0.36	0.64	0.64	-0.89	0.57	0.57	1.08	0	6.25	1478.83	0.94	0.41	0.32
1163	QSRNICDISCDKF	13	-0.31	0.67	0.67	-0.55	0.57	0.38	1.08	0	6.25	1528.89	0.99	0.50	0.47
1164	SRNICDISCDKFL	13	-0.21	0.66	0.66	0.01	0.47	0.22	0.92	0	6.25	1513.92	0.99	0.65	0.66
1165	RNICDISCDKFLD	13	-0.25	0.68	0.68	-0.2	0.47	0.43	0.92	-1	4.43	1541.93	0.99	0.47	0.64
1166	NICDISCDKFLDD	13	-0.17	0.68	0.68	-0.12	0.28	0.43	0.69	-3	3.77	1500.83	0.99	0.43	0.25
1167	ICDISCDKFLDDD	13	-0.18	0.68	0.68	-0.12	0.28	0.65	0.62	-4	3.66	1501.81	0.99	0.43	0.21
1168	CDISCDKFLDDDI	13	-0.18	0.68	0.68	-0.12	0.28	0.65	0.62	-4	3.66	1501.81	0.61	0.42	0.17
1169	DISCDKFLDDDIT	13	-0.19	0.68	0.68	-0.37	0.28	0.69	0.69	-4	3.66	1499.78	0.61	0.44	0.05
1170	ISCDKFLDDDITD	13	-0.19	0.68	0.68	-0.37	0.28	0.69	0.69	-4	3.66	1499.78	0.99	0.42	0.07
1171	SCDKFLDDDITDD	13	-0.3	0.68	0.68	-0.98	0.28	1.06	0.77	-5	3.57	1501.7	0.99	0.43	0.08
1172	CDKFLDDDITDDI	13	-0.23	0.69	0.69	-0.58	0.28	0.9	0.69	-5	3.57	1527.79	0.61	0.42	0.04
1173	DKFLDDDITDDIM	13	-0.21	0.71	0.71	-0.62	0.28	0.88	0.69	-5	3.57	1555.85	0.61	0.39	0.04
1174	KFLDDDITDDIMC	13	-0.15	0.7	0.7	-0.16	0.28	0.57	0.62	-4	3.66	1543.9	0.99	0.49	0.03
1175	FLDDDITDDIMCA	13	-0.05	0.68	0.68	0.28	0	0.3	0.46	-5	3.25	1486.8	0.61	0.48	0.02
1176	LDDDITDDIMCAK	13	-0.18	0.68	0.68	-0.24	0.28	0.72	0.62	-4	3.66	1467.8	0.99	0.41	0.03
1177	DDDITDDIMCAKK	13	-0.31	0.69	0.69	-0.83	0.56	1.09	0.77	-3	3.98	1482.81	0.61	0.64	0.07
1178	DDITDDIMCAKKI	13	-0.19	0.69	0.69	-0.22	0.56	0.72	0.69	-2	4.14	1480.89	0.86	0.68	0.10
1179	DITDDIMCAKKIL	13	-0.1	0.67	0.67	0.35	0.56	0.35	0.62	-1	4.43	1478.97	0.86	0.73	0.35
1180	ITDDIMCAKKILD	13	-0.1	0.67	0.67	0.35	0.56	0.35	0.62	-1	4.43	1478.97	0.99	0.63	0.20
1181	TDDIMCAKKILDI	13	-0.1	0.67	0.67	0.35	0.56	0.35	0.62	-1	4.43	1478.97	0.99	0.63	0.20
1182	DDIMCAKKILDIK	13	-0.17	0.68	0.68	0.1	0.85	0.62	0.69	0	6.35	1506.04	0.61	0.63	0.71
1183	DIMCAKKILDIKG	13	-0.1	0.68	0.68	0.34	0.85	0.38	0.62	1	8.51	1448.01	0.61	0.51	0.93
1184	IMCAKKILDIKGI	13	0.01	0.67	0.67	0.95	0.85	0.02	0.54	2	9.25	1446.09	0.99	0.46	0.95
1185	MCAKKILDIKGID	13	-0.1	0.68	0.68	0.34	0.85	0.38	0.62	1	8.51	1448.01	0.99	0.44	0.94
1186	CAKKILDIKGIDY	13	-0.12	0.67	0.67	0.09	0.85	0.31	0.69	1	8.48	1479.99	0.54	0.46	0.95
1187	AKKILDIKGIDYW	13	-0.09	0.66	0.66	-0.17	0.85	0.12	0.77	1	8.76	1563.07	0.61	0.54	0.92
1188	KKILDIKGIDYWL	13	-0.07	0.66	0.66	-0.02	0.85	0.02	0.77	1	8.76	1605.16	0.99	0.48	0.67
1189	KILDIKGIDYWLA	13	0.03	0.65	0.65	0.42	0.56	-0.25	0.62	0	6.3	1548.06	0.99	0.48	0.65
1190	ILDIKGIDYWLAH	13	0.09	0.6	0.6	0.48	0.39	-0.52	0.54	-0.5	5.22	1557.03	0.99	0.48	0.27
1191	LDIKGIDYWLAHK	13	-0.06	0.6	0.6	-0.17	0.68	-0.15	0.69	0.5	7.09	1572.04	0.99	0.46	0.35
1192	DIKGIDYWLAHKA	13	-0.08	0.59	0.59	-0.32	0.68	-0.05	0.69	0.5	7.09	1529.95	0.86	0.59	0.67
1193	IKGIDYWLAHKAL	13	0.02	0.58	0.58	0.24	0.68	-0.42	0.62	1.5	8.84	1528.03	0.99	0.60	0.90
1194	KGIDYWLAHKALC	13	-0.03	0.57	0.57	0.08	0.68	-0.35	0.62	1.5	8.51	1518	0.98	0.63	0.94
1195	GIDYWLAHKALCT	13	0.04	0.56	0.56	0.33	0.39	-0.62	0.54	0.5	7.06	1490.93	0.98	0.68	0.91
1196	IDYWLAHKALCTE	13	-0.02	0.56	0.56	0.09	0.49	-0.38	0.62	-0.5	5.33	1562.99	0.97	0.67	0.89
1197	DYWLAHKALCTEK	13	-0.16	0.56	0.56	-0.55	0.77	-0.02	0.77	0.5	7.06	1578	0.61	0.66	0.93
1198	YWLAHKALCTEKL	13	-0.07	0.54	0.54	0.01	0.77	-0.38	0.69	1.5	8.51	1576.08	0.99	0.62	0.98
1199	WLAHKALCTEKLE	13	-0.12	0.54	0.54	-0.16	0.87	0.02	0.69	0.5	7.07	1542.02	0.99	0.57	0.85
1200	LAHKALCTEKLEQ	13	-0.2	0.55	0.55	-0.36	0.97	0.3	0.77	0.5	7.07	1483.94	0.99	0.57	0.38
1201	AHKALCTEKLEQW	13	-0.21	0.55	0.55	-0.72	0.97	0.18	0.85	0.5	7.07	1556.99	0.86	0.44	0.60
1202	HKALCTEKLEQWL	13	-0.19	0.55	0.55	-0.57	0.97	0.08	0.85	0.5	7.07	1599.08	0.99	0.33	0.82
1203	KALCTEKLEQWLC	13	-0.16	0.6	0.6	-0.13	0.86	0.04	0.77	0	6.43	1565.07	0.99	0.35	0.76
1204	ALCTEKLEQWLCE	13	-0.12	0.6	0.6	-0.1	0.67	0.04	0.69	-2	4.26	1566.01	0.61	0.23	0.57
1205	LCTEKLEQWLCEK	13	-0.22	0.61	0.61	-0.54	0.95	0.31	0.85	-1	4.79	1623.11	0.99	0.18	0.29
1206	CTEKLEQWLCEKL	13	-0.22	0.61	0.61	-0.54	0.95	0.31	0.85	-1	4.79	1623.11	0.61	0.20	0.57
1207	MRFFVPLFLVGILF	14	0.32	0.64	0.64	2.21	0.18	-1.32	0.29	1	10.11	1699.39	0.93	0.65	0.98
1208	RFFVPLFLVGILFP	14	0.29	0.61	0.61	1.96	0.18	-1.23	0.29	1	10.11	1665.31	0.93	0.67	0.96
1209	FFVPLFLVGILFPA	14	0.44	0.6	0.6	2.41	0	-1.48	0	0	5.88	1580.2	0.94	0.70	0.98
1210	FVPLFLVGILFPAI	14	0.45	0.6	0.6	2.53	0	-1.43	0	0	5.88	1546.19	0.94	0.65	0.95
1211	VPLFLVGILFPAIL	14	0.44	0.59	0.59	2.6	0	-1.38	0	0	5.88	1512.18	0.93	0.59	0.74
1212	PLFLVGILFPAILA	14	0.42	0.58	0.58	2.43	0	-1.31	0	0	5.88	1484.12	0.98	0.60	0.82
1213	LFLVGILFPAILAK	14	0.35	0.6	0.6	2.26	0.26	-1.09	0.14	1	9.11	1515.18	0.98	0.67	0.95
1214	FLVGILFPAILAKQ	14	0.26	0.61	0.61	1.74	0.35	-0.95	0.29	1	9.11	1530.15	0.80	0.61	0.99

1215	LVGILFPAILAKQF	14	0.26	0.61	0.61	1.74	0.35	-0.95	0.29	1	9.11	1530.15	0.98	0.53	0.98
1216	VGILFPAILAKQFT	14	0.21	0.61	0.61	1.42	0.35	-0.85	0.36	1	9.11	1518.09	0.98	0.56	0.98
1217	GILFPAILAKQFTK	14	0.09	0.61	0.61	0.84	0.61	-0.53	0.5	2	10.02	1547.13	0.95	0.62	0.98
1218	ILFPAILAKQFTKC	14	0.08	0.6	0.6	1.05	0.61	-0.6	0.5	2	9.36	1593.21	0.93	0.67	0.98
1219	LFPAILAKQFTKCE	14	-0.02	0.6	0.6	0.48	0.7	-0.26	0.57	1	8.54	1609.16	0.93	0.64	0.97
1220	FPAILAKQFTKCEL	14	-0.02	0.6	0.6	0.48	0.7	-0.26	0.57	1	8.54	1609.16	0.94	0.59	0.94
1221	PAILAKQFTKCELS	14	-0.08	0.59	0.59	0.22	0.7	-0.06	0.64	1	8.54	1549.06	0.98	0.58	0.78
1222	AILAKQFTKCELSQ	14	-0.12	0.61	0.61	0.09	0.79	-0.04	0.79	1	8.54	1580.08	0.80	0.53	0.61
1223	ILAKQFTKCELSQL	14	-0.1	0.61	0.61	0.23	0.79	-0.14	0.79	1	8.54	1622.17	0.99	0.51	0.36
1224	LAKQFTKCELSQLL	14	-0.12	0.6	0.6	0.18	0.79	-0.14	0.79	1	8.54	1622.17	0.99	0.48	0.85
1225	AKQFTKCELSQLLK	14	-0.23	0.61	0.61	-0.37	1.06	0.21	0.93	2	9.25	1637.18	0.61	0.44	0.84
1226	KQFTKCELSQLLKD	14	-0.3	0.63	0.63	-0.75	1.06	0.46	1	1	8.51	1681.19	0.99	0.37	0.67
1227	QFTKCELSQLLKDI	14	-0.17	0.63	0.63	-0.15	0.79	0.11	0.86	0	6.38	1666.18	0.94	0.35	0.19
1228	FTKCELSQLLKDID	14	-0.17	0.64	0.64	-0.15	0.7	0.31	0.79	-1	4.56	1653.13	0.61	0.31	0.27
1229	TKCELSQLLKDIDG	14	-0.21	0.64	0.64	-0.38	0.7	0.49	0.79	-1	4.56	1563.01	0.99	0.37	0.05
1230	KCELSQLLKDIDGY	14	-0.19	0.65	0.65	-0.42	0.7	0.36	0.79	-1	4.56	1625.08	0.99	0.29	0.04
1231	CELSQLLKDIDGYG	14	-0.1	0.65	0.65	-0.17	0.44	0.14	0.64	-2	4.03	1553.96	0.61	0.30	0.04
1232	ELSQLLKDIDGYGG	14	-0.09	0.65	0.65	-0.38	0.44	0.21	0.64	-2	4.03	1507.88	0.61	0.31	0.03
1233	LSQLLKDIDGYGGI	14	0	0.65	0.65	0.19	0.35	-0.13	0.57	-1	4.21	1491.93	0.99	0.31	0.11
1234	SQLLKDIDGYGGIA	14	-0.02	0.65	0.65	0.05	0.35	-0.04	0.57	-1	4.21	1449.84	0.99	0.32	0.11
1235	QLLKDIDGYGGIAL	14	0.04	0.65	0.65	0.38	0.35	-0.19	0.5	-1	4.21	1475.93	0.94	0.42	0.09
1236	LLKDIDGYGGIALP	14	0.09	0.63	0.63	0.51	0.26	-0.2	0.36	-1	4.21	1444.91	0.99	0.55	0.61
1237	LKDIDGYGGIALPE	14	0	0.64	0.64	-0.01	0.35	0.14	0.43	-2	4.03	1460.86	0.99	0.53	0.07
1238	KDIDGYGGIALPEL	14	0	0.64	0.64	-0.01	0.35	0.14	0.43	-2	4.03	1460.86	0.99	0.55	0.06
1239	DIDGYGGIALPELI	14	0.13	0.64	0.64	0.59	0.09	-0.2	0.29	-3	3.5	1445.85	0.61	0.58	0.10
1240	IDGYGGIALPELIC	14	0.19	0.63	0.63	1.02	0.09	-0.49	0.21	-2	3.67	1433.9	0.97	0.59	0.47
1241	DGYGGIALPELICT	14	0.12	0.62	0.62	0.65	0.09	-0.39	0.29	-2	3.67	1421.84	0.86	0.59	0.16
1242	GYGGIALPELICTM	14	0.19	0.62	0.62	1.04	0.09	-0.69	0.21	-1	4	1437.95	0.98	0.58	0.67
1243	YGGIALPELICTMF	14	0.23	0.62	0.62	1.26	0.09	-0.87	0.21	-1	4	1528.07	0.99	0.59	0.19
1244	GGIALPELICTMFH	14	0.2	0.57	0.57	1.13	0.19	-0.74	0.21	-0.5	5.25	1502.04	0.93	0.63	0.65
1245	GIALPELICTMFHT	14	0.17	0.56	0.56	1.11	0.19	-0.77	0.29	-0.5	5.25	1546.09	0.98	0.64	0.27
1246	IALPELICTMFHTS	14	0.14	0.55	0.55	1.08	0.19	-0.75	0.36	-0.5	5.25	1576.11	0.97	0.54	0.25
1247	ALPELICTMFHTSG	14	0.1	0.55	0.55	0.73	0.19	-0.62	0.36	-0.5	5.25	1520	0.61	0.42	0.16
1248	LPELICTMFHTSGY	14	0.08	0.56	0.56	0.51	0.19	-0.75	0.43	-0.5	5.25	1612.1	0.98	0.37	0.07
1249	PELICTMFHTSGYD	14	-0.01	0.58	0.58	-0.01	0.19	-0.41	0.5	-1.5	4.36	1614.02	0.87	0.32	0.05
1250	ELICTMFHTSGYDT	14	-0.01	0.59	0.59	0.05	0.19	-0.44	0.57	-1.5	4.36	1618.01	0.80	0.32	0.04
1251	LICTMFHTSGYDTQ	14	-0.02	0.59	0.59	0.05	0.19	-0.64	0.64	-0.5	5.09	1617.03	0.93	0.37	0.17
1252	ICTMFHTSGYDTQA	14	-0.04	0.59	0.59	-0.09	0.19	-0.54	0.64	-0.5	5.09	1574.94	0.93	0.38	0.38
1253	CTMFHTSGYDTQAI	14	-0.04	0.59	0.59	-0.09	0.19	-0.54	0.64	-0.5	5.09	1574.94	0.94	0.31	0.13
1254	TMFHTSGYDTQAIV	14	0	0.6	0.6	0.03	0.19	-0.58	0.64	-0.5	5.09	1570.94	0.98	0.37	0.11
1255	MFHTSGYDTQAIVE	14	-0.03	0.61	0.61	-0.17	0.28	-0.34	0.64	-1.5	4.36	1598.95	0.98	0.37	0.09
1256	FHTSGYDTQAIVEN	14	-0.1	0.6	0.6	-0.56	0.28	-0.23	0.79	-1.5	4.36	1581.86	0.94	0.39	0.06
1257	HTSGYDTQAIVENN	14	-0.19	0.61	0.61	-1.01	0.28	-0.04	0.93	-1.5	4.36	1548.79	0.93	0.36	0.04
1258	TSGYDTQAIVENNE	14	-0.2	0.66	0.66	-1.03	0.27	0.21	0.93	-3	3.58	1540.76	0.93	0.29	0.03
1259	SGYDTQAIVENNES	14	-0.21	0.66	0.66	-1.04	0.27	0.26	0.93	-3	3.58	1526.73	0.93	0.31	0.04
1260	GYDTQAIVENNEST	14	-0.2	0.66	0.66	-1.03	0.27	0.21	0.93	-3	3.58	1540.76	0.83	0.33	0.03
1261	YDTQAIVENNESTE	14	-0.26	0.66	0.66	-1.25	0.36	0.43	1	-4	3.51	1612.82	0.98	0.37	0.05
1262	DTQAIVENNESTEY	14	-0.26	0.66	0.66	-1.25	0.36	0.43	1	-4	3.51	1612.82	0.94	0.39	0.06
1263	TQAIVENNESTEYG	14	-0.2	0.65	0.65	-1.03	0.36	0.21	0.93	-3	3.68	1554.79	0.98	0.42	0.04
1264	QAIVENNESTEYGL	14	-0.15	0.65	0.65	-0.71	0.36	0.11	0.86	-3	3.68	1566.85	0.99	0.38	0.07
1265	AIVENNESTEYGLF	14	-0.05	0.65	0.65	-0.26	0.27	-0.08	0.71	-3	3.68	1585.89	0.61	0.45	0.08
1266	IVENNESTEYGLFQ	14	-0.12	0.66	0.66	-0.64	0.36	-0.03	0.86	-3	3.68	1642.95	0.94	0.45	0.09
1267	VENNESTEYGLFQI	14	-0.12	0.66	0.66	-0.64	0.36	-0.03	0.86	-3	3.68	1642.95	0.99	0.45	0.09
1268	ENNESTEYGLFQIS	14	-0.18	0.65	0.65	-0.99	0.36	0.1	0.93	-3	3.68	1630.89	0.61	0.45	0.12
1269	NNESTEYGLFQISN	14	-0.18	0.66	0.66	-0.99	0.27	-0.1	1	-2	3.8	1615.88	0.94	0.41	0.05
1270	NESTEYGLFQISNK	14	-0.21	0.65	0.65	-1.02	0.53	0.1	1	-1	4.54	1629.95	0.99	0.42	0.07
1271	ESTEYGLFQISNKL	14	-0.13	0.64	0.64	-0.5	0.53	-0.04	0.86	-1	4.54	1629.01	0.61	0.54	0.14
1272	STEYGLFQISNKLW	14	-0.06	0.62	0.62	-0.31	0.44	-0.5	0.86	0	6.35	1686.11	0.99	0.52	0.26
1273	TEYGLFQISNKLWC	14	-0.04	0.63	0.63	-0.08	0.44	-0.59	0.79	0	6.32	1702.17	0.97	0.56	0.29
1274	EYGLFQISNKLWCK	14	-0.1	0.64	0.64	-0.31	0.7	-0.35	0.86	1	8.51	1729.24	0.61	0.53	0.87
1275	YGLFQISNKLWCKS	14	-0.08	0.63	0.63	-0.11	0.61	-0.54	0.86	2	9.25	1687.2	0.99	0.57	0.98
1276	GLFQISNKLWCKSS	14	-0.1	0.62	0.62	-0.08	0.61	-0.36	0.86	2	9.36	1611.1	0.98	0.61	0.99
1277	LFQISNKLWCKSSQ	14	-0.16	0.62	0.62	-0.3	0.7	-0.34	1	2	9.36	1682.18	0.94	0.62	0.87
1278	FQISNKLWCKSSQV	14	-0.16	0.63	0.63	-0.27	0.7	-0.32	1	2	9.36	1668.15	0.61	0.58	0.27

1279	QISNKLWCKSSQVP	14	-0.2	0.61	0.61	-0.59	0.7	-0.14	1	2	9.36	1618.09	0.99	0.61	0.12
1280	ISNKLWCKSSQVPQ	14	-0.2	0.61	0.61	-0.59	0.7	-0.14	1	2	9.36	1618.09	0.99	0.57	0.12
1281	SNKLWCKSSQVPQS	14	-0.27	0.59	0.59	-0.96	0.7	0.01	1.07	2	9.36	1592	0.99	0.50	0.08
1282	NKLWCKSSQVPQSR	14	-0.38	0.6	0.6	-1.23	0.88	0.2	1.29	3	10.07	1661.11	0.99	0.46	0.58
1283	KLWCKSSQVPQSRN	14	-0.38	0.6	0.6	-1.23	0.88	0.2	1.29	3	10.07	1661.11	0.99	0.47	0.68
1284	LWCKSSQVPQSRNI	14	-0.25	0.61	0.61	-0.63	0.62	-0.14	1.14	2	9.55	1646.1	0.99	0.40	0.28
1285	WCKSSQVPQSRNIC	14	-0.29	0.61	0.61	-0.72	0.62	-0.09	1.14	2	9.03	1636.07	0.97	0.41	0.73
1286	CKSSQVPQSRNICD	14	-0.36	0.63	0.63	-0.91	0.62	0.37	1.14	1	8.38	1564.94	0.86	0.42	0.09
1287	KSSQVPQSRNICDI	14	-0.31	0.64	0.64	-0.76	0.62	0.31	1.14	1	8.57	1574.97	0.99	0.48	0.06
1288	SSQVPQSRNICDIS	14	-0.26	0.63	0.63	-0.54	0.35	0.12	1.07	0	6.16	1533.87	0.99	0.51	0.06
1289	SQVPQSRNICDISC	14	-0.23	0.63	0.63	-0.31	0.35	0.03	1	0	6.13	1549.93	0.99	0.51	0.81
1290	QVPQSRNICDISCD	14	-0.27	0.65	0.65	-0.5	0.35	0.22	1	-1	4.21	1577.94	0.99	0.50	0.24
1291	VPQSRNICDISCDK	14	-0.3	0.65	0.65	-0.53	0.53	0.42	1	0	6.25	1577.98	0.99	0.45	0.22
1292	PQSRNICDISCDKF	14	-0.29	0.65	0.65	-0.63	0.53	0.35	1	0	6.25	1626.02	0.94	0.43	0.47
1293	QSRNICDISCDKFL	14	-0.25	0.66	0.66	-0.24	0.53	0.22	1	0	6.25	1642.07	0.99	0.60	0.56
1294	SRNICDISCDKFLD	14	-0.25	0.67	0.67	-0.24	0.44	0.42	0.93	-1	4.43	1629.02	0.99	0.44	0.48
1295	RNICDISCDKFLDD	14	-0.28	0.68	0.68	-0.44	0.44	0.61	0.93	-2	4.14	1657.03	0.99	0.41	0.32
1296	NICDISCDKFLDDD	14	-0.21	0.69	0.69	-0.36	0.26	0.61	0.71	-4	3.66	1615.93	0.99	0.39	0.14
1297	ICDISCDKFLDDDI	14	-0.11	0.68	0.68	0.21	0.26	0.47	0.57	-4	3.66	1614.99	0.99	0.39	0.30
1298	CDISCDKFLDDDIT	14	-0.18	0.67	0.67	-0.16	0.26	0.57	0.64	-4	3.66	1602.93	0.80	0.40	0.06
1299	DISCDKFLDDDITD	14	-0.23	0.68	0.68	-0.59	0.26	0.86	0.71	-5	3.57	1614.88	0.61	0.38	0.03
1300	ISCDKFLDDDITDD	14	-0.23	0.68	0.68	-0.59	0.26	0.86	0.71	-5	3.57	1614.88	0.99	0.38	0.03
1301	SCDKFLDDDITDDI	14	-0.23	0.68	0.68	-0.59	0.26	0.86	0.71	-5	3.57	1614.88	0.99	0.39	0.06
1302	CDKFLDDDITDDIM	14	-0.19	0.7	0.7	-0.4	0.26	0.74	0.64	-5	3.57	1659	0.61	0.36	0.03
1303	DKFLDDDITDDIMC	14	-0.19	0.7	0.7	-0.4	0.26	0.74	0.64	-5	3.57	1659	0.61	0.35	0.04
1304	KFLDDDITDDIMCA	14	-0.12	0.68	0.68	-0.02	0.26	0.49	0.57	-4	3.66	1614.99	0.99	0.46	0.03
1305	FLDDDITDDIMCAK	14	-0.12	0.68	0.68	-0.02	0.26	0.49	0.57	-4	3.66	1614.99	0.61	0.46	0.06
1306	LDDDITDDIMCAKK	14	-0.25	0.68	0.68	-0.5	0.52	0.89	0.71	-3	3.98	1595.99	0.99	0.49	0.06
1307	DDDITDDIMCAKKI	14	-0.23	0.69	0.69	-0.45	0.52	0.89	0.71	-3	3.98	1595.99	0.61	0.64	0.07
1308	DDITDDIMCAKKIL	14	-0.14	0.68	0.68	0.07	0.52	0.54	0.64	-2	4.14	1594.07	0.86	0.69	0.12
1309	DITDDIMCAKKILD	14	-0.14	0.68	0.68	0.07	0.52	0.54	0.64	-2	4.14	1594.07	0.86	0.57	0.08
1310	ITDDIMCAKKILDI	14	-0.04	0.67	0.67	0.64	0.52	0.2	0.57	-1	4.43	1592.15	0.99	0.57	0.34
1311	TDDIMCAKKILDIK	14	-0.17	0.67	0.67	0.04	0.79	0.54	0.71	0	6.35	1607.16	0.99	0.59	0.40
1312	DDIMCAKKILDIKG	14	-0.15	0.68	0.68	0.06	0.79	0.57	0.64	0	6.35	1563.11	0.61	0.49	0.88
1313	DIMCAKKILDIKGI	14	-0.04	0.68	0.68	0.64	0.79	0.23	0.57	1	8.51	1561.19	0.61	0.46	0.80
1314	IMCAKKILDIKGID	14	-0.04	0.68	0.68	0.64	0.79	0.23	0.57	1	8.51	1561.19	0.99	0.43	0.96
1315	MCAKKILDIKGIDY	14	-0.09	0.68	0.68	0.22	0.79	0.19	0.64	1	8.48	1611.2	0.99	0.45	0.93
1316	CAKKILDIKGIDYW	14	-0.08	0.66	0.66	0.02	0.79	0.04	0.71	1	8.48	1666.22	0.54	0.53	0.98
1317	AKKILDIKGIDYWL	14	-0.05	0.65	0.65	0.11	0.79	-0.01	0.71	1	8.76	1676.25	0.61	0.51	0.96
1318	KKILDIKGIDYWLA	14	-0.05	0.65	0.65	0.11	0.79	-0.01	0.71	1	8.76	1676.25	0.99	0.48	0.88
1319	KILDIKGIDYWLAH	14	0	0.6	0.6	0.16	0.63	-0.26	0.64	0.5	7.09	1685.22	0.99	0.45	0.41
1320	ILDIKGIDYWLAHK	14	0	0.6	0.6	0.16	0.63	-0.26	0.64	0.5	7.09	1685.22	0.99	0.46	0.45
1321	LDIKGIDYWLAHKA	14	-0.03	0.59	0.59	-0.03	0.63	-0.17	0.64	0.5	7.09	1643.13	0.99	0.49	0.64
1322	DIKGIDYWLAHKAL	14	-0.03	0.59	0.59	-0.03	0.63	-0.17	0.64	0.5	7.09	1643.13	0.86	0.53	0.81
1323	IKGIDYWLAHKALC	14	0.02	0.58	0.58	0.4	0.63	-0.46	0.57	1.5	8.51	1631.18	0.97	0.53	0.94
1324	KGIDYWLAHKALCT	14	-0.04	0.57	0.57	0.03	0.63	-0.36	0.64	1.5	8.51	1619.12	0.99	0.58	0.84
1325	GIDYWLAHKALCTE	14	-0.01	0.57	0.57	0.06	0.46	-0.36	0.57	-0.5	5.33	1620.06	0.98	0.57	0.73
1326	IDYWLAHKALCTEK	14	-0.1	0.57	0.57	-0.19	0.72	-0.14	0.71	0.5	7.06	1691.18	0.99	0.56	0.86
1327	DYWLAHKALCTEKL	14	-0.11	0.56	0.56	-0.24	0.72	-0.14	0.71	0.5	7.06	1691.18	0.61	0.54	0.97
1328	YWLAHKALCTEKLE	14	-0.11	0.55	0.55	-0.24	0.81	-0.14	0.71	0.5	7.06	1705.21	0.99	0.54	0.87
1329	WLAHKALCTEKLEQ	14	-0.16	0.55	0.55	-0.4	0.9	0.04	0.79	0.5	7.07	1670.17	0.99	0.43	0.60
1330	LAHKALCTEKLEQW	14	-0.16	0.55	0.55	-0.4	0.9	0.04	0.79	0.5	7.07	1670.17	0.99	0.47	0.75
1331	AHKALCTEKLEQWL	14	-0.16	0.55	0.55	-0.4	0.9	0.04	0.79	0.5	7.07	1670.17	0.86	0.33	0.84
1332	HKALCTEKLEQWLC	14	-0.17	0.56	0.56	-0.35	0.9	0	0.79	0.5	7.04	1702.23	0.99	0.27	0.86
1333	KALCTEKLEQWLCE	14	-0.19	0.6	0.6	-0.37	0.89	0.25	0.79	-1	4.79	1694.2	0.99	0.27	0.48
1334	ALCTEKLEQWLCEK	14	-0.19	0.6	0.6	-0.37	0.89	0.25	0.79	-1	4.79	1694.2	0.61	0.19	0.37
1335	LCTEKLEQWLCEKL	14	-0.17	0.6	0.6	-0.23	0.89	0.16	0.79	-1	4.79	1736.29	0.99	0.16	0.62
1336	MRFFVPLFLVGILFP	15	0.29	0.62	0.62	1.95	0.16	-1.23	0.27	1	10.11	1796.52	0.93	0.64	0.98
1337	RFFVPLFLVGILFPA	15	0.29	0.61	0.61	1.95	0.16	-1.18	0.27	1	10.11	1736.4	0.98	0.70	0.96
1338	FFVPLFLVGILFPAI	15	0.46	0.61	0.61	2.55	0	-1.5	0	0	5.88	1693.38	0.94	0.69	0.97
1339	FVPLFLVGILFPAIL	15	0.45	0.6	0.6	2.61	0	-1.45	0	0	5.88	1659.37	0.94	0.69	0.93
1340	VPLFLVGILFPAILA	15	0.43	0.58	0.58	2.55	0	-1.32	0	0	5.88	1583.27	0.98	0.61	0.61
1341	PLFLVGILFPAILAK	15	0.32	0.58	0.58	2.01	0.24	-1.02	0.13	1	9.11	1612.31	0.98	0.70	0.98
1342	LFLVGILFPAILAKQ	15	0.28	0.6	0.6	1.88	0.33	-1.01	0.27	1	9.11	1643.33	0.98	0.63	0.92

1343	FLVGILFPAILAKQF	15	0.28	0.62	0.62	1.81	0.33	-1.05	0.27	1	9.11	1677.34	0.94	0.60	0.98
1344	LVGILFPAILAKQFT	15	0.23	0.6	0.6	1.58	0.33	-0.91	0.33	1	9.11	1631.27	0.98	0.50	0.83
1345	VGILFPAILAKQFTK	15	0.12	0.61	0.61	1.07	0.57	-0.59	0.47	2	10.02	1646.28	0.98	0.56	0.99
1346	GILFPAILAKQFTKC	15	0.09	0.61	0.61	0.95	0.57	-0.56	0.47	2	9.36	1650.28	0.95	0.63	0.98
1347	ILFPAILAKQFTKCE	15	0.03	0.61	0.61	0.75	0.66	-0.36	0.53	1	8.54	1722.34	0.93	0.65	0.97
1348	LFPAILAKQFTKCEL	15	0.02	0.6	0.6	0.7	0.66	-0.36	0.53	1	8.54	1722.34	0.93	0.56	0.87
1349	FPAILAKQFTKCELS	15	-0.03	0.6	0.6	0.39	0.66	-0.22	0.6	1	8.54	1696.25	0.94	0.58	0.93
1350	PAILAKQFTKCELSQ	15	-0.12	0.6	0.6	-0.03	0.74	-0.04	0.73	1	8.54	1677.21	0.98	0.58	0.54
1351	AILAKQFTKCELSQL	15	-0.08	0.61	0.61	0.33	0.74	-0.16	0.73	1	8.54	1693.26	0.80	0.48	0.50
1352	ILAKQFTKCELSQLL	15	-0.06	0.61	0.61	0.47	0.74	-0.25	0.73	1	8.54	1735.35	0.99	0.49	0.77
1353	LAKQFTKCELSQLLK	15	-0.18	0.61	0.61	-0.09	0.99	0.07	0.87	2	9.25	1750.36	0.99	0.45	0.92
1354	AKQFTKCELSQLLKD	15	-0.26	0.62	0.62	-0.58	0.99	0.39	0.93	1	8.51	1752.28	0.86	0.37	0.59
1355	KQFTKCELSQLLKDI	15	-0.23	0.63	0.63	-0.4	0.99	0.31	0.93	1	8.51	1794.37	0.99	0.35	0.49
1356	QFTKCELSQLLKDID	15	-0.21	0.64	0.64	-0.37	0.74	0.31	0.87	-1	4.56	1781.28	0.94	0.33	0.10
1357	FTKCELSQLLKDIDG	15	-0.15	0.64	0.64	-0.17	0.66	0.29	0.73	-1	4.56	1710.2	0.61	0.32	0.12
1358	TKCELSQLLKDIDGY	15	-0.19	0.64	0.64	-0.44	0.66	0.31	0.8	-1	4.56	1726.2	0.99	0.32	0.04
1359	KCELSQLLKDIDGYG	15	-0.17	0.65	0.65	-0.42	0.66	0.33	0.73	-1	4.56	1682.15	0.99	0.30	0.11
1360	CELSQLLKDIDGYGG	15	-0.08	0.65	0.65	-0.19	0.41	0.13	0.6	-2	4.03	1611.03	0.61	0.29	0.04
1361	ELSQLLKDIDGYGGI	15	-0.04	0.65	0.65	-0.05	0.41	0.08	0.6	-2	4.03	1621.06	0.61	0.29	0.03
1362	LSQLLKDIDGYGGIA	15	0.02	0.64	0.64	0.3	0.33	-0.15	0.53	-1	4.21	1563.02	0.99	0.32	0.10
1363	SQLLKDIDGYGGIAL	15	0.02	0.64	0.64	0.3	0.33	-0.15	0.53	-1	4.21	1563.02	0.99	0.37	0.14
1364	QLLKDIDGYGGIALP	15	0.03	0.63	0.63	0.25	0.33	-0.17	0.47	-1	4.21	1573.06	0.94	0.43	0.11
1365	LLKDIDGYGGIALPE	15	0.04	0.63	0.63	0.25	0.33	0.01	0.4	-2	4.03	1574.04	0.99	0.51	0.09
1366	LKDIDGYGGIALPEL	15	0.04	0.63	0.63	0.25	0.33	0.01	0.4	-2	4.03	1574.04	0.99	0.48	0.06
1367	KDIDGYGGIALPELI	15	0.05	0.64	0.64	0.29	0.33	0.01	0.4	-2	4.03	1574.04	0.99	0.48	0.04
1368	DIDGYGGIALPELIC	15	0.13	0.64	0.64	0.72	0.08	-0.25	0.27	-3	3.5	1549	0.86	0.53	0.11
1369	IDGYGGIALPELICT	15	0.16	0.62	0.62	0.91	0.08	-0.48	0.27	-2	3.67	1535.02	0.97	0.54	0.17
1370	DGYGGIALPELICTM	15	0.13	0.63	0.63	0.73	0.08	-0.45	0.27	-2	3.67	1553.05	0.86	0.56	0.06
1371	GYGGIALPELICTMF	15	0.22	0.63	0.63	1.15	0.08	-0.81	0.2	-1	4	1585.14	0.98	0.53	0.56
1372	YGGIALPELICTMFH	15	0.18	0.58	0.58	0.97	0.18	-0.85	0.27	-0.5	5.25	1665.23	0.97	0.61	0.25
1373	GGIALPELICTMFHT	15	0.17	0.57	0.57	1.01	0.18	-0.72	0.27	-0.5	5.25	1603.16	0.98	0.59	0.27
1374	GIALPELICTMFHTS	15	0.14	0.56	0.56	0.98	0.18	-0.7	0.33	-0.5	5.25	1633.18	0.98	0.51	0.46
1375	IALPELICTMFHTSG	15	0.14	0.56	0.56	0.98	0.18	-0.7	0.33	-0.5	5.25	1633.18	0.97	0.42	0.21
1376	ALPELICTMFHTSGY	15	0.09	0.56	0.56	0.59	0.18	-0.73	0.4	-0.5	5.25	1683.19	0.80	0.36	0.11
1377	LPELICTMFHTSGYD	15	0.03	0.58	0.58	0.24	0.18	-0.5	0.47	-1.5	4.36	1727.2	0.98	0.36	0.06
1378	PELICTMFHTSGYDT	15	-0.02	0.58	0.58	-0.06	0.18	-0.41	0.53	-1.5	4.36	1715.14	0.87	0.31	0.04
1379	ELICTMFHTSGYDTQ	15	-0.06	0.6	0.6	-0.19	0.26	-0.39	0.67	-1.5	4.36	1746.16	0.80	0.32	0.03
1380	LICTMFHTSGYDTQA	15	0	0.59	0.59	0.17	0.18	-0.63	0.6	-0.5	5.09	1688.12	0.98	0.36	0.26
1381	ICTMFHTSGYDTQAI	15	0.01	0.6	0.6	0.21	0.18	-0.63	0.6	-0.5	5.09	1688.12	0.93	0.33	0.29
1382	CTMFHTSGYDTQAIV	15	0	0.6	0.6	0.19	0.18	-0.61	0.6	-0.5	5.09	1674.09	0.94	0.39	0.11
1383	TMFHTSGYDTQAIVE	15	-0.04	0.6	0.6	-0.21	0.26	-0.34	0.67	-1.5	4.36	1700.07	0.98	0.38	0.13
1384	MFHTSGYDTQAIVEN	15	-0.07	0.62	0.62	-0.39	0.26	-0.3	0.73	-1.5	4.36	1713.07	0.98	0.39	0.07
1385	FHTSGYDTQAIVENN	15	-0.13	0.62	0.62	-0.75	0.26	-0.2	0.87	-1.5	4.36	1695.98	0.94	0.39	0.05
1386	HTSGYDTQAIVENNE	15	-0.22	0.61	0.61	-1.17	0.35	0.17	0.93	-2.5	4.14	1677.92	0.93	0.31	0.03
1387	TSGYDTQAIVENNES	15	-0.21	0.65	0.65	-1.01	0.25	0.22	0.93	-3	3.58	1627.85	0.93	0.26	0.03
1388	SGYDTQAIVENNEST	15	-0.21	0.65	0.65	-1.01	0.25	0.22	0.93	-3	3.58	1627.85	0.93	0.31	0.03
1389	GYDTQAIVENNESTE	15	-0.23	0.66	0.66	-1.19	0.34	0.4	0.93	-4	3.51	1669.89	0.83	0.35	0.03
1390	YDTQAIVENNESTEY	15	-0.24	0.66	0.66	-1.25	0.34	0.25	1	-4	3.51	1776.01	0.98	0.39	0.06
1391	DTQAIVENNESTEYG	15	-0.23	0.66	0.66	-1.19	0.34	0.4	0.93	-4	3.51	1669.89	0.94	0.39	0.04
1392	TQAIVENNESTEYGL	15	-0.15	0.64	0.64	-0.71	0.34	0.08	0.87	-3	3.68	1667.97	0.98	0.40	0.06
1393	QAIVENNESTEYGLF	15	-0.1	0.66	0.66	-0.47	0.34	-0.06	0.8	-3	3.68	1714.04	0.99	0.43	0.08
1394	AIVENNESTEYGLFQ	15	-0.1	0.66	0.66	-0.47	0.34	-0.06	0.8	-3	3.68	1714.04	0.80	0.47	0.08
1395	IVENNESTEYGLFQI	15	-0.06	0.67	0.67	-0.29	0.34	-0.15	0.8	-3	3.68	1756.13	0.94	0.49	0.08
1396	VENNESTEYGLFQIS	15	-0.13	0.66	0.66	-0.65	0.34	-0.01	0.87	-3	3.68	1730.04	0.99	0.48	0.08
1397	ENNESTEYGLFQISN	15	-0.21	0.66	0.66	-1.16	0.34	0.11	1	-3	3.68	1745.01	0.61	0.42	0.07
1398	NNESTEYGLFQISNK	15	-0.24	0.66	0.66	-1.19	0.5	0.11	1.07	-1	4.54	1744.07	0.94	0.40	0.05
1399	NESTEYGLFQISNKL	15	-0.16	0.64	0.64	-0.7	0.5	-0.03	0.93	-1	4.54	1743.13	0.99	0.43	0.12
1400	ESTEYGLFQISNKLW	15	-0.09	0.63	0.63	-0.53	0.5	-0.27	0.87	-1	4.54	1815.24	0.61	0.49	0.11
1401	STEYGLFQISNKLWC	15	-0.05	0.62	0.62	-0.13	0.41	-0.53	0.8	0	6.32	1789.26	0.97	0.50	0.58
1402	TEYGLFQISNKLWCK	15	-0.11	0.63	0.63	-0.33	0.66	-0.35	0.87	1	8.51	1830.36	0.97	0.48	0.60
1403	EYGLFQISNKLWCKS	15	-0.11	0.63	0.63	-0.34	0.66	-0.31	0.87	1	8.51	1816.33	0.61	0.52	0.84
1404	YGLFQISNKLWCKSS	15	-0.09	0.62	0.62	-0.16	0.57	-0.49	0.87	2	9.25	1774.29	0.99	0.58	0.95
1405	GLFQISNKLWCKSSQ	15	-0.14	0.62	0.62	-0.31	0.66	-0.32	0.93	2	9.36	1739.25	0.98	0.58	0.97
1406	LFQISNKLWCKSSQV	15	-0.11	0.62	0.62	0	0.66	-0.42	0.93	2	9.36	1781.33	0.94	0.59	0.51

1407	FQISNKLWCKSSQVP	15	-0.15	0.61	0.61	-0.36	0.66	-0.3	0.93	2	9.36	1765.28	0.61	0.53	0.29
1408	QISNKLWCKSSQVPQ	15	-0.24	0.61	0.61	-0.78	0.74	-0.12	1.07	2	9.36	1746.24	0.99	0.54	0.09
1409	ISNKLWCKSSQVPQS	15	-0.21	0.6	0.6	-0.6	0.66	-0.11	1	2	9.36	1705.18	0.99	0.52	0.05
1410	SNKLWCKSSQVPQSR	15	-0.37	0.6	0.6	-1.2	0.82	0.21	1.27	3	10.07	1748.2	0.99	0.43	0.10
1411	NKLWCKSSQVPQSRN	15	-0.4	0.61	0.61	-1.38	0.82	0.2	1.33	3	10.07	1775.23	0.99	0.43	0.46
1412	KLWCKSSQVPQSRNI	15	-0.31	0.61	0.61	-0.85	0.82	0.07	1.2	3	10.07	1774.29	0.99	0.43	0.65
1413	LWCKSSQVPQSRNIC	15	-0.23	0.61	0.61	-0.42	0.57	-0.2	1.07	2	9.03	1749.25	0.99	0.41	0.67
1414	WCKSSQVPQSRNICD	15	-0.32	0.62	0.62	-0.91	0.57	0.12	1.13	1	8.38	1751.17	0.97	0.43	0.61
1415	CKSSQVPQSRNICDI	15	-0.29	0.64	0.64	-0.55	0.57	0.23	1.07	1	8.38	1678.12	0.86	0.43	0.11
1416	KSSQVPQSRNICDIS	15	-0.31	0.63	0.63	-0.77	0.57	0.31	1.13	1	8.57	1662.06	0.99	0.51	0.04
1417	SSQVPQSRNICDISC	15	-0.24	0.63	0.63	-0.34	0.33	0.05	1	0	6.13	1637.02	0.99	0.49	0.42
1418	SQVPQSRNICDISCD	15	-0.27	0.64	0.64	-0.52	0.33	0.23	1	-1	4.21	1665.03	0.99	0.49	0.23
1419	QVPQSRNICDISCDK	15	-0.32	0.65	0.65	-0.73	0.57	0.41	1.07	0	6.25	1706.13	0.99	0.47	0.13
1420	VPQSRNICDISCDKF	15	-0.24	0.65	0.65	-0.31	0.49	0.23	0.93	0	6.25	1725.17	0.99	0.49	0.47
1421	PQSRNICDISCDKFL	15	-0.24	0.64	0.64	-0.33	0.49	0.21	0.93	0	6.25	1739.2	0.87	0.54	0.59
1422	QSRNICDISCDKFLD	15	-0.28	0.67	0.67	-0.46	0.49	0.41	1	-1	4.43	1757.17	0.99	0.41	0.36
1423	SRNICDISCDKFLDD	15	-0.28	0.67	0.67	-0.46	0.41	0.59	0.93	-2	4.14	1744.12	0.99	0.42	0.28
1424	RNICDISCDKFLDDD	15	-0.31	0.69	0.69	-0.64	0.41	0.77	0.93	-3	3.98	1772.13	0.99	0.41	0.19
1425	NICDISCDKFLDDDI	15	-0.15	0.69	0.69	-0.04	0.24	0.45	0.67	-4	3.66	1729.11	0.98	0.39	0.19
1426	ICDISCDKFLDDDIT	15	-0.12	0.67	0.67	0.15	0.24	0.41	0.6	-4	3.66	1716.11	0.98	0.39	0.09
1427	CDISCDKFLDDDITD	15	-0.21	0.68	0.68	-0.39	0.24	0.73	0.67	-5	3.57	1718.03	0.61	0.37	0.03
1428	DISCDKFLDDDITDD	15	-0.26	0.69	0.69	-0.79	0.24	1	0.73	-6	3.5	1729.98	0.61	0.37	0.03
1429	ISCDKFLDDDITDDI	15	-0.17	0.68	0.68	-0.25	0.24	0.68	0.67	-5	3.57	1728.06	0.99	0.37	0.03
1430	SCDKFLDDDITDDIM	15	-0.2	0.69	0.69	-0.43	0.24	0.71	0.67	-5	3.57	1746.09	0.99	0.36	0.12
1431	CDKFLDDDITDDIMC	15	-0.18	0.69	0.69	-0.21	0.24	0.63	0.6	-5	3.57	1762.15	0.61	0.35	0.05
1432	DKFLDDDITDDIMCA	15	-0.16	0.69	0.69	-0.25	0.24	0.66	0.6	-5	3.57	1730.09	0.61	0.35	0.02
1433	KFLDDDITDDIMCAK	15	-0.19	0.68	0.68	-0.28	0.49	0.66	0.67	-3	3.98	1743.18	0.99	0.45	0.03
1434	FLDDDITDDIMCAKK	15	-0.19	0.68	0.68	-0.28	0.49	0.66	0.67	-3	3.98	1743.18	0.61	0.48	0.15
1435	LDDDITDDIMCAKKI	15	-0.18	0.68	0.68	-0.17	0.49	0.71	0.67	-3	3.98	1709.17	0.99	0.47	0.03
1436	DDDITDDIMCAKKIL	15	-0.18	0.68	0.68	-0.17	0.49	0.71	0.67	-3	3.98	1709.17	0.61	0.65	0.10
1437	DDITDDIMCAKKILD	15	-0.18	0.68	0.68	-0.17	0.49	0.71	0.67	-3	3.98	1709.17	0.86	0.52	0.05
1438	DITDDIMCAKKILDI	15	-0.08	0.68	0.68	0.37	0.49	0.39	0.6	-2	4.14	1707.25	0.61	0.52	0.13
1439	ITDDIMCAKKILDIK	15	-0.11	0.67	0.67	0.34	0.73	0.39	0.67	0	6.35	1720.34	0.99	0.53	0.45
1440	TDDIMCAKKILDIKG	15	-0.15	0.67	0.67	0.01	0.73	0.51	0.67	0	6.35	1664.23	0.99	0.43	0.57
1441	DDIMCAKKILDIKGI	15	-0.09	0.68	0.68	0.36	0.73	0.41	0.6	0	6.35	1676.29	0.61	0.43	0.65
1442	DIMCAKKILDIKGID	15	-0.09	0.68	0.68	0.36	0.73	0.41	0.6	0	6.35	1676.29	0.61	0.41	0.88
1443	IMCAKKILDIKGIDY	15	-0.04	0.68	0.68	0.51	0.73	0.06	0.6	1	8.48	1724.38	0.99	0.42	0.96
1444	MCAKKILDIKGIDYW	15	-0.06	0.67	0.67	0.15	0.73	-0.05	0.67	1	8.48	1797.43	0.98	0.51	0.97
1445	CAKKILDIKGIDYWL	15	-0.04	0.65	0.65	0.27	0.73	-0.08	0.67	1	8.48	1779.4	0.69	0.50	0.98
1446	AKKILDIKGIDYWLA	15	-0.03	0.64	0.64	0.23	0.73	-0.05	0.67	1	8.76	1747.34	0.80	0.51	0.98
1447	KKILDIKGIDYWLAH	15	-0.07	0.61	0.61	-0.11	0.83	-0.05	0.73	1.5	8.76	1813.41	0.98	0.47	0.78
1448	KILDIKGIDYWLAHK	15	-0.07	0.61	0.61	-0.11	0.83	-0.05	0.73	1.5	8.76	1813.41	0.99	0.45	0.70
1449	ILDIKGIDYWLAHKA	15	0.02	0.6	0.6	0.27	0.59	-0.28	0.6	0.5	7.09	1756.31	0.99	0.49	0.56
1450	LDIKGIDYWLAHKAL	15	0	0.59	0.59	0.23	0.59	-0.28	0.6	0.5	7.09	1756.31	0.99	0.44	0.58
1451	DIKGIDYWLAHKALC	15	-0.03	0.59	0.59	0.14	0.59	-0.23	0.6	0.5	7.06	1746.28	0.94	0.51	0.85
1452	IKGIDYWLAHKALCT	15	0.01	0.58	0.58	0.33	0.59	-0.45	0.6	1.5	8.51	1732.3	0.97	0.53	0.83
1453	KGIDYWLAHKALCTE	15	-0.08	0.58	0.58	-0.21	0.67	-0.13	0.67	0.5	7.06	1748.25	0.99	0.52	0.60
1454	GIDYWLAHKALCTEK	15	-0.08	0.58	0.58	-0.21	0.67	-0.13	0.67	0.5	7.06	1748.25	0.98	0.50	0.69
1455	IDYWLAHKALCTEKL	15	-0.06	0.57	0.57	0.07	0.67	-0.25	0.67	0.5	7.06	1804.36	0.99	0.49	0.90
1456	DYWLAHKALCTEKLE	15	-0.15	0.56	0.56	-0.46	0.76	0.07	0.73	-0.5	5.46	1820.31	0.86	0.48	0.50
1457	YWLAHKALCTEKLEQ	15	-0.15	0.56	0.56	-0.46	0.84	-0.12	0.8	0.5	7.06	1833.36	0.99	0.43	0.57
1458	WLAHKALCTEKLEQW	15	-0.12	0.55	0.55	-0.43	0.84	-0.19	0.8	0.5	7.07	1856.4	0.99	0.37	0.83
1459	LAHKALCTEKLEQWL	15	-0.11	0.55	0.55	-0.12	0.84	-0.09	0.73	0.5	7.07	1783.35	0.99	0.37	0.90
1460	AHKALCTEKLEQWLC	15	-0.14	0.55	0.55	-0.21	0.84	-0.03	0.73	0.5	7.04	1773.32	0.86	0.28	0.85
1461	HKALCTEKLEQWLCE	15	-0.2	0.56	0.56	-0.56	0.92	0.2	0.8	-0.5	5.51	1831.36	0.98	0.21	0.21
1462	KALCTEKLEQWLCEK	15	-0.25	0.61	0.61	-0.61	1.07	0.43	0.87	0	6.51	1822.39	0.99	0.23	0.36
1463	ALCTEKLEQWLCEKL	15	-0.14	0.6	0.6	-0.09	0.83	0.11	0.73	-1	4.79	1807.38	0.61	0.19	0.65
1464	MRFFVPLFLVGILFPA	16	0.29	0.62	0.62	1.94	0.15	-1.19	0.25	1	10.11	1867.61	0.93	0.67	0.97
1465	RFFVPLFLVGILFPAI	16	0.32	0.61	0.61	2.11	0.15	-1.22	0.25	1	10.11	1849.58	0.93	0.66	0.97
1466	FFVPLFLVGILFPAIL	16	0.46	0.6	0.6	2.62	0	-1.52	0	0	5.88	1806.56	0.94	0.68	0.97
1467	FVPLFLVGILFPAILA	16	0.44	0.59	0.59	2.56	0	-1.39	0	0	5.88	1730.46	0.94	0.67	0.89
1468	VPLFLVGILFPAILAK	16	0.33	0.59	0.59	2.14	0.23	-1.05	0.12	1	9.11	1711.46	0.98	0.69	0.98
1469	PLFLVGILFPAILAKQ	16	0.25	0.59	0.59	1.66	0.31	-0.94	0.25	1	9.11	1740.46	0.98	0.65	0.98
1470	LFLVGILFPAILAKQF	16	0.3	0.61	0.61	1.94	0.31	-1.1	0.25	1	9.11	1790.52	0.98	0.64	0.98

1471	FLVGILFPAILAKQFT	16	0.25	0.61	0.61	1.66	0.31	-1.01	0.31	1	9.11	1778.46	0.94	0.54	0.95
1472	LVGILFPAILAKQFTK	16	0.15	0.61	0.61	1.24	0.54	-0.67	0.44	2	10.02	1759.46	0.98	0.51	0.97
1473	VGILFPAILAKQFTKC	16	0.12	0.61	0.61	1.16	0.54	-0.62	0.44	2	9.36	1749.43	0.93	0.58	0.98
1474	GILFPAILAKQFTKCE	16	0.04	0.61	0.61	0.68	0.62	-0.34	0.5	1	8.54	1779.41	0.95	0.61	0.99
1475	ILFPAILAKQFTKCEL	16	0.07	0.6	0.6	0.94	0.62	-0.45	0.5	1	8.54	1835.52	0.93	0.58	0.87
1476	LFPAILAKQFTKCELS	16	0	0.59	0.59	0.61	0.62	-0.32	0.56	1	8.54	1809.43	0.93	0.57	0.79
1477	FPAILAKQFTKCELSQ	16	-0.07	0.6	0.6	0.15	0.69	-0.19	0.69	1	8.54	1824.4	0.94	0.59	0.72
1478	PAILAKQFTKCELSQL	16	-0.08	0.59	0.59	0.21	0.69	-0.15	0.69	1	8.54	1790.39	0.98	0.54	0.37
1479	AILAKQFTKCELSQLL	16	-0.04	0.6	0.6	0.55	0.69	-0.26	0.69	1	8.54	1806.44	0.80	0.47	0.81
1480	ILAKQFTKCELSQLLK	16	-0.12	0.61	0.61	0.19	0.92	-0.04	0.81	2	9.25	1863.54	0.99	0.52	0.84
1481	LAKQFTKCELSQLLKD	16	-0.22	0.62	0.62	-0.31	0.92	0.26	0.88	1	8.51	1865.46	0.99	0.43	0.88
1482	AKQFTKCELSQLLKDI	16	-0.2	0.63	0.63	-0.26	0.92	0.26	0.88	1	8.51	1865.46	0.86	0.35	0.44
1483	KQFTKCELSQLLKDID	16	-0.26	0.64	0.64	-0.59	0.92	0.48	0.94	0	6.43	1909.47	0.99	0.34	0.31
1484	QFTKCELSQLLKDIDG	16	-0.18	0.64	0.64	-0.38	0.69	0.29	0.81	-1	4.56	1838.35	0.94	0.33	0.05
1485	FTKCELSQLLKDIDGY	16	-0.14	0.64	0.64	-0.24	0.62	0.13	0.75	-1	4.56	1873.39	0.61	0.28	0.14
1486	TKCELSQLLKDIDGYG	16	-0.17	0.64	0.64	-0.44	0.62	0.29	0.75	-1	4.56	1783.27	0.99	0.32	0.08
1487	KCELSQLLKDIDGYGG	16	-0.15	0.65	0.65	-0.42	0.62	0.31	0.69	-1	4.56	1739.22	0.99	0.30	0.05
1488	CELSQLLKDIDGYGGI	16	-0.03	0.65	0.65	0.11	0.39	0.01	0.56	-2	4.03	1724.21	0.61	0.28	0.03
1489	ELSQLLKDIDGYGGIA	16	-0.02	0.65	0.65	0.06	0.39	0.04	0.56	-2	4.03	1692.15	0.61	0.30	0.03
1490	LSQLLKDIDGYGGIAL	16	0.05	0.64	0.64	0.52	0.31	-0.26	0.5	-1	4.21	1676.2	0.99	0.37	0.18
1491	SQLLKDIDGYGGIALP	16	0.01	0.63	0.63	0.18	0.31	-0.14	0.5	-1	4.21	1660.15	0.99	0.41	0.21
1492	QLLKDIDGYGGIALPE	16	-0.01	0.64	0.64	0.01	0.39	0.02	0.5	-2	4.03	1702.19	0.94	0.42	0.04
1493	LLKDIDGYGGIALPEL	16	0.07	0.63	0.63	0.47	0.31	-0.1	0.38	-2	4.03	1687.22	0.99	0.49	0.08
1494	LKDIDGYGGIALPELI	16	0.08	0.64	0.64	0.51	0.31	-0.1	0.38	-2	4.03	1687.22	0.99	0.46	0.05
1495	KDIDGYGGIALPELIC	16	0.05	0.64	0.64	0.43	0.31	-0.05	0.38	-2	4.03	1677.19	0.99	0.48	0.08
1496	DIDGYGGIALPELICT	16	0.11	0.63	0.63	0.63	0.08	-0.26	0.31	-3	3.5	1650.12	0.61	0.52	0.09
1497	IDGYGGIALPELICTM	16	0.17	0.63	0.63	0.97	0.08	-0.53	0.25	-2	3.67	1666.23	0.97	0.54	0.13
1498	DGYGGIALPELICTMF	16	0.16	0.63	0.63	0.86	0.08	-0.57	0.25	-2	3.67	1700.24	0.86	0.52	0.06
1499	GYGGIALPELICTMFH	16	0.18	0.59	0.59	0.88	0.17	-0.79	0.25	-0.5	5.25	1722.3	0.98	0.53	0.53
1500	YGGIALPELICTMFHT	16	0.16	0.58	0.58	0.86	0.17	-0.82	0.31	-0.5	5.25	1766.35	0.97	0.58	0.12
1501	GGIALPELICTMFHTS	16	0.14	0.57	0.57	0.89	0.17	-0.66	0.31	-0.5	5.25	1690.25	0.98	0.50	0.51
1502	GIALPELICTMFHTSG	16	0.14	0.57	0.57	0.89	0.17	-0.66	0.31	-0.5	5.25	1690.25	0.98	0.39	0.50
1503	IALPELICTMFHTSGY	16	0.13	0.57	0.57	0.84	0.17	-0.8	0.38	-0.5	5.25	1796.37	0.99	0.34	0.15
1504	ALPELICTMFHTSGYD	16	0.04	0.57	0.57	0.34	0.17	-0.5	0.44	-1.5	4.36	1798.29	0.80	0.34	0.09
1505	LPELICTMFHTSGYDT	16	0.02	0.57	0.57	0.18	0.17	-0.49	0.5	-1.5	4.36	1828.32	0.98	0.34	0.05
1506	PELICTMFHTSGYDTQ	16	-0.06	0.58	0.58	-0.28	0.25	-0.37	0.62	-1.5	4.36	1843.29	0.87	0.31	0.03
1507	ELICTMFHTSGYDTQA	16	-0.04	0.59	0.59	-0.06	0.25	-0.4	0.62	-1.5	4.36	1817.25	0.80	0.31	0.03
1508	LICTMFHTSGYDTQAI	16	0.05	0.59	0.59	0.44	0.17	-0.7	0.56	-0.5	5.09	1801.3	0.93	0.34	0.08
1509	ICTMFHTSGYDTQAIV	16	0.05	0.6	0.6	0.46	0.17	-0.68	0.56	-0.5	5.09	1787.27	0.93	0.40	0.30
1510	CTMFHTSGYDTQAIVE	16	-0.04	0.6	0.6	-0.04	0.25	-0.38	0.62	-1.5	4.36	1803.22	0.94	0.40	0.10
1511	TMFHTSGYDTQAIVEN	16	-0.08	0.61	0.61	-0.41	0.25	-0.31	0.75	-1.5	4.36	1814.19	0.98	0.40	0.09
1512	MFHTSGYDTQAIVENN	16	-0.11	0.63	0.63	-0.59	0.25	-0.27	0.81	-1.5	4.36	1827.19	0.93	0.38	0.05
1513	FHTSGYDTQAIVENNE	16	-0.17	0.62	0.62	-0.93	0.33	0	0.88	-2.5	4.14	1825.11	0.94	0.33	0.03
1514	HTSGYDTQAIVENNES	16	-0.22	0.61	0.61	-1.15	0.33	0.17	0.94	-2.5	4.14	1765.01	0.93	0.29	0.03
1515	TSGYDTQAIVENNEST	16	-0.21	0.64	0.64	-0.99	0.24	0.18	0.94	-3	3.58	1728.97	0.93	0.24	0.05
1516	SGYDTQAIVENNESTE	16	-0.23	0.65	0.65	-1.17	0.32	0.39	0.94	-4	3.51	1756.98	0.93	0.29	0.04
1517	GYDTQAIVENNESTEY	16	-0.22	0.66	0.66	-1.2	0.32	0.23	0.94	-4	3.51	1833.08	0.83	0.34	0.03
1518	YDTQAIVENNESTEYG	16	-0.22	0.66	0.66	-1.2	0.32	0.23	0.94	-4	3.51	1833.08	0.98	0.36	0.03
1519	DTQAIVENNESTEYGL	16	-0.18	0.65	0.65	-0.88	0.32	0.26	0.88	-4	3.51	1783.07	0.94	0.35	0.07
1520	TQAIVENNESTEYGLF	16	-0.1	0.65	0.65	-0.49	0.32	-0.08	0.81	-3	3.68	1815.16	0.98	0.43	0.08
1521	QAIVENNESTEYGLFQ	16	-0.13	0.66	0.66	-0.66	0.39	-0.04	0.88	-3	3.68	1842.19	0.98	0.41	0.08
1522	AIVENNESTEYGLFQI	16	-0.04	0.66	0.66	-0.16	0.32	-0.17	0.75	-3	3.68	1827.22	0.80	0.49	0.08
1523	IVENNESTEYGLFQIS	16	-0.08	0.66	0.66	-0.33	0.32	-0.12	0.81	-3	3.68	1843.22	0.94	0.50	0.08
1524	VENNESTEYGLFQISN	16	-0.16	0.66	0.66	-0.82	0.32	0.01	0.94	-3	3.68	1844.16	0.99	0.46	0.05
1525	ENNESTEYGLFQISNK	16	-0.26	0.66	0.66	-1.33	0.55	0.29	1.06	-2	4.26	1873.2	0.61	0.42	0.06
1526	NNESTEYGLFQISNKL	16	-0.19	0.65	0.65	-0.87	0.47	-0.01	1	-1	4.54	1857.25	0.94	0.43	0.13
1527	NESTEYGLFQISNKLW	16	-0.13	0.64	0.64	-0.71	0.47	-0.24	0.94	-1	4.54	1929.36	0.99	0.44	0.09
1528	ESTEYGLFQISNKLWC	16	-0.09	0.63	0.63	-0.34	0.47	-0.31	0.81	-1	4.54	1918.39	0.61	0.52	0.11
1529	STEYGLFQISNKLWCK	16	-0.12	0.63	0.63	-0.36	0.62	-0.31	0.88	1	8.51	1917.45	0.97	0.47	0.74
1530	TEYGLFQISNKLWCKS	16	-0.12	0.63	0.63	-0.36	0.62	-0.31	0.88	1	8.51	1917.45	0.97	0.51	0.26
1531	EYGLFQISNKLWCKSS	16	-0.12	0.63	0.63	-0.37	0.62	-0.27	0.88	1	8.51	1903.42	0.61	0.57	0.63
1532	YGLFQISNKLWCKSSQ	16	-0.13	0.63	0.63	-0.37	0.61	-0.44	0.94	2	9.25	1902.44	0.99	0.57	0.70
1533	GLFQISNKLWCKSSQV	16	-0.09	0.63	0.63	-0.03	0.61	-0.39	0.88	2	9.36	1838.4	0.98	0.58	0.93
1534	LFQISNKLWCKSSQVP	16	-0.11	0.61	0.61	-0.1	0.61	-0.39	0.88	2	9.36	1878.46	0.94	0.57	0.39

1535	FQISNKLWCKSSQVPQ	16	-0.18	0.62	0.62	-0.56	0.69	-0.27	1	2	9.36	1893.43	0.61	0.49	0.13
1536	QISNKLWCKSSQVPQS	16	-0.24	0.61	0.61	-0.78	0.69	-0.09	1.06	2	9.36	1833.33	0.99	0.51	0.04
1537	ISNKLWCKSSQVPQSR	16	-0.3	0.61	0.61	-0.84	0.77	0.08	1.19	3	10.07	1861.38	0.99	0.46	0.06
1538	SNKLWCKSSQVPQSRN	16	-0.39	0.61	0.61	-1.34	0.77	0.21	1.31	3	10.07	1862.32	0.99	0.42	0.07
1539	NKLWCKSSQVPQSRNI	16	-0.33	0.62	0.62	-1.01	0.77	0.07	1.25	3	10.07	1888.41	0.99	0.41	0.18
1540	KLWCKSSQVPQSRNIC	16	-0.29	0.61	0.61	-0.64	0.77	0	1.12	3	9.53	1877.44	0.99	0.43	0.86
1541	LWCKSSQVPQSRNICD	16	-0.26	0.62	0.62	-0.61	0.54	0	1.06	1	8.38	1864.35	0.97	0.42	0.50
1542	WCKSSQVPQSRNICDI	16	-0.25	0.63	0.63	-0.57	0.54	0	1.06	1	8.38	1864.35	0.97	0.43	0.23
1543	CKSSQVPQSRNICDIS	16	-0.29	0.63	0.63	-0.56	0.54	0.23	1.06	1	8.38	1765.21	0.86	0.48	0.08
1544	KSSQVPQSRNICDISC	16	-0.29	0.63	0.63	-0.56	0.54	0.23	1.06	1	8.38	1765.21	0.99	0.49	0.27
1545	SSQVPQSRNICDISCD	16	-0.27	0.63	0.63	-0.54	0.31	0.23	1	-1	4.21	1752.12	0.99	0.48	0.15
1546	SQVPQSRNICDISCDK	16	-0.32	0.64	0.64	-0.73	0.54	0.4	1.06	0	6.25	1793.22	0.99	0.47	0.08
1547	QVPQSRNICDISCDKF	16	-0.26	0.65	0.65	-0.51	0.54	0.23	1	0	6.25	1853.32	0.99	0.51	0.23
1548	VPQSRNICDISCDKFL	16	-0.19	0.64	0.64	-0.05	0.46	0.1	0.88	0	6.25	1838.35	0.99	0.54	0.63
1549	PQSRNICDISCDKFLD	16	-0.27	0.65	0.65	-0.53	0.46	0.38	0.94	-1	4.43	1854.3	0.94	0.37	0.33
1550	QSRNICDISCDKFLDD	16	-0.31	0.67	0.67	-0.65	0.46	0.57	1	-2	4.14	1872.27	0.99	0.39	0.22
1551	SRNICDISCDKFLDDD	16	-0.31	0.68	0.68	-0.65	0.38	0.74	0.94	-3	3.98	1859.22	0.99	0.43	0.16
1552	RNICDISCDKFLDDDI	16	-0.25	0.69	0.69	-0.32	0.38	0.61	0.88	-3	3.98	1885.31	0.98	0.42	0.22
1553	NICDISCDKFLDDDIT	16	-0.15	0.68	0.68	-0.08	0.23	0.4	0.69	-4	3.66	1830.23	0.98	0.41	0.06
1554	ICDISCDKFLDDDITD	16	-0.15	0.68	0.68	-0.08	0.23	0.57	0.62	-5	3.57	1831.21	0.99	0.37	0.05
1555	CDISCDKFLDDDITDD	16	-0.24	0.68	0.68	-0.58	0.23	0.87	0.69	-6	3.5	1833.13	0.61	0.37	0.03
1556	DISCDKFLDDDITDDI	16	-0.2	0.69	0.69	-0.46	0.23	0.82	0.69	-6	3.5	1843.16	0.61	0.37	0.04
1557	ISCDKFLDDDITDDIM	16	-0.14	0.69	0.69	-0.12	0.23	0.56	0.62	-5	3.57	1859.27	0.99	0.35	0.09
1558	SCDKFLDDDITDDIMC	16	-0.18	0.68	0.68	-0.24	0.23	0.61	0.62	-5	3.57	1849.24	0.99	0.36	0.14
1559	CDKFLDDDITDDIMCA	16	-0.15	0.68	0.68	-0.08	0.23	0.56	0.56	-5	3.57	1833.24	0.61	0.35	0.03
1560	DKFLDDDITDDIMCAK	16	-0.22	0.69	0.69	-0.48	0.46	0.81	0.69	-4	3.86	1858.28	0.61	0.38	0.04
1561	KFLDDDITDDIMCAKK	16	-0.25	0.68	0.68	-0.51	0.69	0.81	0.75	-2	4.29	1871.37	0.99	0.50	0.11
1562	FLDDDITDDIMCAKKI	16	-0.13	0.68	0.68	0.02	0.46	0.51	0.62	-3	3.98	1856.36	0.61	0.47	0.05
1563	LDDDITDDIMCAKKIL	16	-0.14	0.67	0.67	0.08	0.46	0.55	0.62	-3	3.98	1822.35	0.99	0.48	0.06
1564	DDDITDDIMCAKKILD	16	-0.21	0.69	0.69	-0.37	0.46	0.85	0.69	-4	3.86	1824.27	0.61	0.48	0.04
1565	DDITDDIMCAKKILDI	16	-0.12	0.68	0.68	0.13	0.46	0.55	0.62	-3	3.98	1822.35	0.86	0.48	0.07
1566	DITDDIMCAKKILDIK	16	-0.15	0.68	0.68	0.1	0.69	0.55	0.69	-1	4.58	1835.44	0.61	0.49	0.13
1567	ITDDIMCAKKILDIKG	16	-0.09	0.67	0.67	0.29	0.69	0.36	0.62	0	6.35	1777.41	0.99	0.39	0.60
1568	TDDIMCAKKILDIKGI	16	-0.09	0.67	0.67	0.29	0.69	0.36	0.62	0	6.35	1777.41	0.99	0.38	0.40
1569	DDIMCAKKILDIKGID	16	-0.13	0.69	0.69	0.12	0.69	0.57	0.62	-1	4.58	1791.39	0.61	0.39	0.31
1570	DIMCAKKILDIKGIDY	16	-0.08	0.68	0.68	0.26	0.69	0.24	0.62	0	6.35	1839.48	0.61	0.40	0.54
1571	IMCAKKILDIKGIDYW	16	-0.01	0.67	0.67	0.42	0.69	-0.16	0.62	1	8.48	1910.61	0.99	0.49	0.98
1572	MCAKKILDIKGIDYWL	16	-0.02	0.66	0.66	0.38	0.69	-0.16	0.62	1	8.48	1910.61	0.93	0.49	0.98
1573	CAKKILDIKGIDYWLA	16	-0.03	0.64	0.64	0.37	0.69	-0.11	0.62	1	8.48	1850.49	0.69	0.48	0.98
1574	AKKILDIKGIDYWLAH	16	-0.05	0.6	0.6	0.01	0.78	-0.07	0.69	1.5	8.76	1884.5	0.94	0.49	0.93
1575	KKILDIKGIDYWLAHK	16	-0.14	0.61	0.61	-0.34	1.01	0.14	0.81	2.5	9.43	1941.6	0.99	0.47	0.89
1576	KILDIKGIDYWLAHKA	16	-0.05	0.6	0.6	0.01	0.78	-0.08	0.69	1.5	8.76	1884.5	0.99	0.50	0.82
1577	ILDIKGIDYWLAHKAL	16	0.05	0.59	0.59	0.49	0.55	-0.38	0.56	0.5	7.09	1869.49	0.97	0.46	0.54
1578	LDIKGIDYWLAHKALC	16	0.01	0.59	0.59	0.37	0.55	-0.33	0.56	0.5	7.06	1859.46	0.98	0.45	0.69
1579	DIKGIDYWLAHKALCT	16	-0.04	0.59	0.59	0.09	0.55	-0.24	0.62	0.5	7.06	1847.4	0.86	0.53	0.50
1580	IKGIDYWLAHKALCTE	16	-0.03	0.58	0.58	0.09	0.63	-0.24	0.62	0.5	7.06	1861.43	0.97	0.50	0.64
1581	KGIDYWLAHKALCTEK	16	-0.15	0.58	0.58	-0.44	0.86	0.06	0.75	1.5	8.48	1876.44	0.99	0.51	0.75
1582	GIDYWLAHKALCTEKL	16	-0.04	0.57	0.57	0.04	0.63	-0.24	0.62	0.5	7.06	1861.43	0.95	0.48	0.80
1583	IDYWLAHKALCTEKLE	16	-0.09	0.57	0.57	-0.15	0.71	-0.05	0.69	-0.5	5.46	1933.49	0.99	0.47	0.10
1584	DYWLAHKALCTEKLEQ	16	-0.18	0.57	0.57	-0.65	0.79	0.07	0.81	-0.5	5.46	1948.46	0.86	0.43	0.17
1585	YWLAHKALCTEKLEQW	16	-0.11	0.56	0.56	-0.49	0.79	-0.33	0.81	0.5	7.06	2019.59	0.99	0.42	0.81
1586	WLAHKALCTEKLEQWL	16	-0.08	0.54	0.54	-0.17	0.79	-0.29	0.75	0.5	7.07	1969.58	0.99	0.34	0.90
1587	LAHKALCTEKLEQWLC	16	-0.1	0.55	0.55	0.04	0.79	-0.14	0.69	0.5	7.04	1886.5	0.99	0.36	0.87
1588	AHKALCTEKLEQWLCE	16	-0.17	0.56	0.56	-0.41	0.87	0.16	0.75	-0.5	5.51	1902.45	0.94	0.27	0.33
1589	HKALCTEKLEQWLCEK	16	-0.26	0.57	0.57	-0.77	1.09	0.38	0.88	0.5	7.04	1959.55	0.99	0.21	0.20
1590	KALCTEKLEQWLCEKL	16	-0.2	0.6	0.6	-0.33	1	0.29	0.81	0	6.51	1935.57	0.99	0.25	0.68
1591	MRFFVPLFLVGILFPAI	17	0.31	0.62	0.62	2.09	0.14	-1.22	0.24	1	10.11	1980.79	0.93	0.65	0.98
1592	RFFVPLFLVGILFPAIL	17	0.33	0.61	0.61	2.21	0.14	-1.25	0.24	1	10.11	1962.76	0.93	0.67	0.93
1593	FFVPLFLVGILFPAILA	17	0.45	0.6	0.6	2.58	0	-1.46	0	0	5.88	1877.65	0.94	0.65	0.95
1594	FVPLFLVGILFPAILAK	17	0.35	0.6	0.6	2.18	0.22	-1.14	0.12	1	9.11	1858.65	0.94	0.72	0.98
1595	VPLFLVGILFPAILAKQ	17	0.27	0.6	0.6	1.81	0.29	-0.98	0.24	1	9.11	1839.61	0.98	0.63	0.93
1596	PLFLVGILFPAILAKQF	17	0.28	0.6	0.6	1.73	0.29	-1.04	0.24	1	9.11	1887.65	0.98	0.64	0.98
1597	LFLVGILFPAILAKQFT	17	0.27	0.61	0.61	1.78	0.29	-1.06	0.29	1	9.11	1891.64	0.98	0.57	0.80
1598	FLVGILFPAILAKQFTK	17	0.17	0.61	0.61	1.33	0.51	-0.78	0.41	2	10.02	1906.65	0.94	0.54	0.99

1599	LVGILFPAILAKQFTKC	17	0.14	0.61	0.61	1.31	0.51	-0.69	0.41	2	9.36	1862.61	0.93	0.51	0.99
1600	VGILFPAILAKQFTKCE	17	0.07	0.62	0.62	0.88	0.58	-0.41	0.47	1	8.54	1878.56	0.93	0.57	0.98
1601	GILFPAILAKQFTKCEL	17	0.07	0.61	0.61	0.86	0.58	-0.42	0.47	1	8.54	1892.59	0.83	0.55	0.97
1602	ILFPAILAKQFTKCELS	17	0.05	0.6	0.6	0.84	0.58	-0.41	0.53	1	8.54	1922.61	0.93	0.57	0.89
1603	LFPAILAKQFTKCELSQ	17	-0.04	0.6	0.6	0.36	0.65	-0.29	0.65	1	8.54	1937.58	0.93	0.56	0.54
1604	FPAILAKQFTKCELSQL	17	-0.04	0.6	0.6	0.36	0.65	-0.29	0.65	1	8.54	1937.58	0.80	0.54	0.52
1605	PAILAKQFTKCELSQLL	17	-0.04	0.59	0.59	0.42	0.65	-0.25	0.65	1	8.54	1903.57	0.98	0.52	0.77
1606	AILAKQFTKCELSQLLK	17	-0.1	0.61	0.61	0.29	0.87	-0.07	0.76	2	9.25	1934.63	0.80	0.48	0.86
1607	ILAKQFTKCELSQLLKD	17	-0.16	0.62	0.62	-0.02	0.87	0.14	0.82	1	8.51	1978.64	0.97	0.50	0.66
1608	LAKQFTKCELSQLLKDI	17	-0.16	0.62	0.62	-0.02	0.87	0.14	0.82	1	8.51	1978.64	0.99	0.41	0.81
1609	AKQFTKCELSQLLKDID	17	-0.23	0.63	0.63	-0.45	0.87	0.42	0.88	0	6.43	1980.56	0.86	0.32	0.24
1610	KQFTKCELSQLLKDIDG	17	-0.24	0.64	0.64	-0.58	0.87	0.45	0.88	0	6.43	1966.54	0.99	0.34	0.07
1611	QFTKCELSQLLKDIDGY	17	-0.17	0.65	0.65	-0.43	0.65	0.14	0.82	-1	4.56	2001.54	0.94	0.31	0.04
1612	FTKCELSQLLKDIDGYG	17	-0.12	0.65	0.65	-0.25	0.58	0.12	0.71	-1	4.56	1930.46	0.61	0.30	0.27
1613	TKCELSQLLKDIDGYGG	17	-0.15	0.64	0.64	-0.44	0.58	0.27	0.71	-1	4.56	1840.34	0.99	0.35	0.05
1614	KCELSQLLKDIDGYGGI	17	-0.1	0.65	0.65	-0.13	0.58	0.19	0.65	-1	4.56	1852.4	0.99	0.30	0.03
1615	CELSQLLKDIDGYGGIA	17	-0.02	0.64	0.64	0.21	0.36	-0.02	0.53	-2	4.03	1795.3	0.61	0.28	0.03
1616	ELSQLLKDIDGYGGIAL	17	0.01	0.64	0.64	0.28	0.36	-0.06	0.53	-2	4.03	1805.33	0.61	0.34	0.03
1617	LSQLLKDIDGYGGIALP	17	0.05	0.62	0.62	0.39	0.29	-0.24	0.47	-1	4.21	1773.33	0.99	0.40	0.23
1618	SQLLKDIDGYGGIALPE	17	-0.02	0.63	0.63	-0.04	0.36	0.04	0.53	-2	4.03	1789.28	0.99	0.39	0.11
1619	QLLKDIDGYGGIALPEL	17	0.02	0.63	0.63	0.24	0.36	-0.08	0.47	-2	4.03	1815.37	0.94	0.40	0.04
1620	LLKDIDGYGGIALPELI	17	0.11	0.63	0.63	0.71	0.29	-0.2	0.35	-2	4.03	1800.4	0.99	0.46	0.07
1621	LKDIDGYGGIALPELIC	17	0.08	0.64	0.64	0.63	0.29	-0.15	0.35	-2	4.03	1790.37	0.99	0.47	0.05
1622	KDIDGYGGIALPELICT	17	0.04	0.64	0.64	0.36	0.29	-0.07	0.41	-2	4.03	1778.31	0.99	0.46	0.05
1623	DIDGYGGIALPELICTM	17	0.12	0.64	0.64	0.71	0.07	-0.32	0.29	-3	3.5	1781.33	0.86	0.52	0.03
1624	IDGYGGIALPELICTMF	17	0.2	0.64	0.64	1.08	0.07	-0.65	0.24	-2	3.67	1813.42	0.97	0.53	0.05
1625	DGYGGIALPELICTMFH	17	0.13	0.6	0.6	0.62	0.16	-0.57	0.29	-1.5	4.36	1837.4	0.86	0.54	0.08
1626	GYGGIALPELICTMFHT	17	0.16	0.58	0.58	0.79	0.16	-0.77	0.29	-0.5	5.25	1823.42	0.93	0.52	0.17
1627	YGGIALPELICTMFHTS	17	0.14	0.57	0.57	0.76	0.16	-0.75	0.35	-0.5	5.25	1853.44	0.97	0.49	0.13
1628	GGIALPELICTMFHTSG	17	0.14	0.57	0.57	0.82	0.16	-0.62	0.29	-0.5	5.25	1747.32	0.98	0.37	0.58
1629	GIALPELICTMFHTSGY	17	0.14	0.57	0.57	0.76	0.16	-0.75	0.35	-0.5	5.25	1853.44	0.98	0.33	0.41
1630	IALPELICTMFHTSGYD	17	0.08	0.58	0.58	0.58	0.16	-0.58	0.41	-1.5	4.36	1911.47	0.97	0.31	0.11
1631	ALPELICTMFHTSGYDT	17	0.03	0.57	0.57	0.28	0.16	-0.49	0.47	-1.5	4.36	1899.41	0.80	0.31	0.08
1632	LPELICTMFHTSGYDTQ	17	-0.02	0.58	0.58	-0.04	0.23	-0.45	0.59	-1.5	4.36	1956.47	0.98	0.33	0.03
1633	PELICTMFHTSGYDTQA	17	-0.04	0.58	0.58	-0.15	0.23	-0.38	0.59	-1.5	4.36	1914.38	0.87	0.29	0.03
1634	ELICTMFHTSGYDTQAI	17	0.01	0.6	0.6	0.21	0.23	-0.48	0.59	-1.5	4.36	1930.43	0.80	0.29	0.02
1635	LICTMFHTSGYDTQAIV	17	0.07	0.6	0.6	0.66	0.16	-0.75	0.53	-0.5	5.09	1900.45	0.93	0.37	0.14
1636	ICTMFHTSGYDTQAIVE	17	0.01	0.61	0.61	0.23	0.23	-0.46	0.59	-1.5	4.36	1916.4	0.93	0.39	0.29
1637	CTMFHTSGYDTQAIVEN	17	-0.07	0.61	0.61	-0.24	0.23	-0.35	0.71	-1.5	4.36	1917.34	0.94	0.40	0.09
1638	TMFHTSGYDTQAIVENN	17	-0.11	0.62	0.62	-0.59	0.23	-0.28	0.82	-1.5	4.36	1928.31	0.93	0.37	0.07
1639	MFHTSGYDTQAIVENNE	17	-0.14	0.63	0.63	-0.76	0.31	-0.08	0.82	-2.5	4.14	1956.32	0.93	0.31	0.04
1640	FHTSGYDTQAIVENNES	17	-0.17	0.61	0.61	-0.92	0.31	0.02	0.88	-2.5	4.14	1912.2	0.94	0.31	0.04
1641	HTSGYDTQAIVENNEST	17	-0.22	0.6	0.6	-1.12	0.31	0.14	0.94	-2.5	4.14	1866.13	0.93	0.28	0.04
1642	TSGYDTQAIVENNESTE	17	-0.23	0.64	0.64	-1.14	0.3	0.35	0.94	-4	3.51	1858.1	0.93	0.24	0.04
1643	SGYDTQAIVENNESTEY	17	-0.22	0.65	0.65	-1.18	0.3	0.24	0.94	-4	3.51	1920.17	0.93	0.31	0.04
1644	GYDTQAIVENNESTEYG	17	-0.19	0.66	0.66	-1.15	0.3	0.22	0.88	-4	3.51	1890.15	0.83	0.34	0.03
1645	YDTQAIVENNESTEYGL	17	-0.17	0.65	0.65	-0.91	0.3	0.11	0.88	-4	3.51	1946.26	0.98	0.35	0.04
1646	DTQAIVENNESTEYGLF	17	-0.14	0.65	0.65	-0.66	0.3	0.1	0.82	-4	3.51	1930.26	0.94	0.40	0.08
1647	TQAIVENNESTEYGLFQ	17	-0.14	0.65	0.65	-0.66	0.37	-0.06	0.88	-3	3.68	1943.31	0.98	0.41	0.07
1648	QAIVENNESTEYGLFQI	17	-0.08	0.66	0.66	-0.36	0.37	-0.15	0.82	-3	3.68	1955.37	0.98	0.42	0.08
1649	AIVENNESTEYGLFQIS	17	-0.06	0.65	0.65	-0.2	0.3	-0.14	0.76	-3	3.68	1914.31	0.80	0.51	0.07
1650	IVENNESTEYGLFQISN	17	-0.11	0.66	0.66	-0.51	0.3	-0.1	0.88	-3	3.68	1957.34	0.94	0.47	0.05
1651	VENNESTEYGLFQISNK	17	-0.22	0.66	0.66	-1.01	0.51	0.18	1	-2	4.26	1972.35	0.99	0.42	0.04
1652	ENNESTEYGLFQISNKL	17	-0.22	0.65	0.65	-1.03	0.51	0.16	1	-2	4.26	1986.38	0.61	0.42	0.10
1653	NNESTEYGLFQISNKLW	17	-0.16	0.64	0.64	-0.88	0.44	-0.21	1	-1	4.54	2043.48	0.94	0.41	0.07
1654	NESTEYGLFQISNKLWC	17	-0.12	0.63	0.63	-0.52	0.44	-0.28	0.88	-1	4.54	2032.51	0.99	0.45	0.10
1655	ESTEYGLFQISNKLWCK	17	-0.15	0.63	0.63	-0.55	0.65	-0.12	0.88	0	6.45	2046.58	0.61	0.47	0.23
1656	STEYGLFQISNKLWCKS	17	-0.12	0.62	0.62	-0.39	0.58	-0.28	0.88	1	8.51	2004.54	0.97	0.48	0.63
1657	TEYGLFQISNKLWCKSS	17	-0.12	0.62	0.62	-0.39	0.58	-0.28	0.88	1	8.51	2004.54	0.97	0.55	0.13
1658	EYGLFQISNKLWCKSSQ	17	-0.15	0.63	0.63	-0.55	0.65	-0.24	0.94	1	8.51	2031.57	0.61	0.56	0.33
1659	YGLFQISNKLWCKSSQV	17	-0.09	0.63	0.63	-0.1	0.58	-0.51	0.88	2	9.25	2001.59	0.99	0.57	0.20
1660	GLFQISNKLWCKSSQVP	17	-0.09	0.61	0.61	-0.12	0.58	-0.37	0.82	2	9.36	1935.53	0.98	0.57	0.91
1661	LFQISNKLWCKSSQVPQ	17	-0.14	0.61	0.61	-0.3	0.65	-0.36	0.94	2	9.36	2006.61	0.94	0.55	0.23
1662	FQISNKLWCKSSQVPQS	17	-0.19	0.61	0.61	-0.57	0.65	-0.24	1	2	9.36	1980.52	0.61	0.49	0.05

1663	QISNKLWCKSSQVPQSR	17	-0.33	0.61	0.61	-1	0.8	0.09	1.24	3	10.07	1989.53	0.99	0.46	0.05
1664	ISNKLWCKSSQVPQSRN	17	-0.32	0.61	0.61	-1	0.72	0.09	1.24	3	10.07	1975.5	0.99	0.46	0.05
1665	SNKLWCKSSQVPQSRNI	17	-0.32	0.61	0.61	-1	0.72	0.09	1.24	3	10.07	1975.5	0.99	0.40	0.09
1666	NKLWCKSSQVPQSRNIC	17	-0.31	0.62	0.62	-0.81	0.72	0.01	1.18	3	9.53	1991.56	0.99	0.42	0.80
1667	KLWCKSSQVPQSRNICD	17	-0.31	0.62	0.62	-0.81	0.72	0.18	1.12	2	8.98	1992.54	0.99	0.45	0.69
1668	LWCKSSQVPQSRNICDI	17	-0.2	0.62	0.62	-0.31	0.51	-0.11	1	1	8.38	1977.53	0.97	0.42	0.23
1669	WCKSSQVPQSRNICDIS	17	-0.25	0.62	0.62	-0.58	0.51	0.02	1.06	1	8.38	1951.44	0.97	0.47	0.46
1670	CKSSQVPQSRNICDISC	17	-0.27	0.63	0.63	-0.38	0.51	0.16	1	1	8.26	1868.36	0.86	0.44	0.71
1671	KSSQVPQSRNICDISCD	17	-0.31	0.64	0.64	-0.74	0.51	0.39	1.06	0	6.25	1880.31	0.99	0.45	0.05
1672	SSQVPQSRNICDISCDK	17	-0.31	0.64	0.64	-0.74	0.51	0.39	1.06	0	6.25	1880.31	0.99	0.43	0.06
1673	SQVPQSRNICDISCDKF	17	-0.26	0.65	0.65	-0.52	0.51	0.23	1	0	6.25	1940.41	0.99	0.48	0.19
1674	QVPQSRNICDISCDKFL	17	-0.22	0.65	0.65	-0.25	0.51	0.11	0.94	0	6.25	1966.5	0.99	0.51	0.36
1675	VPQSRNICDISCDKFLD	17	-0.22	0.65	0.65	-0.25	0.43	0.27	0.88	-1	4.43	1953.45	0.99	0.37	0.29
1676	PQSRNICDISCDKFLDD	17	-0.29	0.65	0.65	-0.71	0.43	0.54	0.94	-2	4.14	1969.4	0.87	0.33	0.19
1677	QSRNICDISCDKFLDDD	17	-0.33	0.68	0.68	-0.82	0.43	0.71	1	-3	3.98	1987.37	0.98	0.38	0.13
1678	SRNICDISCDKFLDDDI	17	-0.25	0.68	0.68	-0.35	0.36	0.59	0.88	-3	3.98	1972.4	0.99	0.42	0.17
1679	RNICDISCDKFLDDDIT	17	-0.24	0.68	0.68	-0.34	0.36	0.55	0.88	-3	3.98	1986.43	0.98	0.42	0.08
1680	NICDISCDKFLDDDITD	17	-0.18	0.68	0.68	-0.28	0.22	0.55	0.71	-5	3.57	1945.33	0.98	0.39	0.04
1681	ICDISCDKFLDDDITDD	17	-0.19	0.68	0.68	-0.28	0.22	0.72	0.65	-6	3.5	1946.31	0.99	0.37	0.03
1682	CDISCDKFLDDDITDDI	17	-0.19	0.68	0.68	-0.28	0.22	0.72	0.65	-6	3.5	1946.31	0.61	0.37	0.04
1683	DISCDKFLDDDITDDIM	17	-0.17	0.69	0.69	-0.32	0.22	0.7	0.65	-6	3.5	1974.37	0.61	0.35	0.03
1684	ISCDKFLDDDITDDIMC	17	-0.13	0.69	0.69	0.04	0.22	0.46	0.59	-5	3.57	1962.42	0.99	0.35	0.13
1685	SCDKFLDDDITDDIMCA	17	-0.16	0.67	0.67	-0.12	0.22	0.54	0.59	-5	3.57	1920.33	0.99	0.35	0.09
1686	CDKFLDDDITDDIMCAK	17	-0.21	0.68	0.68	-0.31	0.43	0.7	0.65	-4	3.86	1961.43	0.61	0.37	0.03
1687	DKFLDDDITDDIMCAKK	17	-0.27	0.69	0.69	-0.68	0.65	0.94	0.76	-3	4.12	1986.47	0.61	0.40	0.12
1688	KFLDDDITDDIMCAKKI	17	-0.19	0.68	0.68	-0.21	0.65	0.65	0.71	-2	4.29	1984.55	0.99	0.49	0.06
1689	FLDDDITDDIMCAKKIL	17	-0.09	0.67	0.67	0.24	0.43	0.37	0.59	-3	3.98	1969.54	0.61	0.48	0.11
1690	LDDDITDDIMCAKKILD	17	-0.17	0.68	0.68	-0.13	0.43	0.69	0.65	-4	3.86	1937.45	0.99	0.43	0.03
1691	DDDITDDIMCAKKILDI	17	-0.16	0.69	0.69	-0.09	0.43	0.69	0.65	-4	3.86	1937.45	0.86	0.43	0.05
1692	DDITDDIMCAKKILDIK	17	-0.18	0.68	0.68	-0.11	0.65	0.69	0.71	-2	4.29	1950.54	0.61	0.43	0.07
1693	DITDDIMCAKKILDIKG	17	-0.13	0.68	0.68	0.07	0.65	0.52	0.65	-1	4.58	1892.51	0.61	0.38	0.15
1694	ITDDIMCAKKILDIKGI	17	-0.04	0.68	0.68	0.54	0.65	0.24	0.59	0	6.35	1890.59	0.99	0.36	0.40
1695	TDDIMCAKKILDIKGID	17	-0.13	0.68	0.68	0.07	0.65	0.52	0.65	-1	4.58	1892.51	0.99	0.34	0.11
1696	DDIMCAKKILDIKGIDY	17	-0.12	0.69	0.69	0.04	0.65	0.41	0.65	-1	4.58	1954.58	0.61	0.37	0.11
1697	DIMCAKKILDIKGIDYW	17	-0.05	0.67	0.67	0.19	0.65	0.03	0.65	0	6.35	2025.71	0.86	0.44	0.75
1698	IMCAKKILDIKGIDYWL	17	0.02	0.66	0.66	0.62	0.65	-0.25	0.59	1	8.48	2023.79	0.97	0.47	0.98
1699	MCAKKILDIKGIDYWLA	17	-0.01	0.65	0.65	0.46	0.65	-0.18	0.59	1	8.48	1981.7	0.93	0.46	0.98
1700	CAKKILDIKGIDYWLAH	17	-0.05	0.6	0.6	0.16	0.73	-0.13	0.65	1.5	8.48	1987.65	0.69	0.46	0.98
1701	AKKILDIKGIDYWLAHK	17	-0.11	0.61	0.61	-0.22	0.95	0.11	0.76	2.5	9.43	2012.69	0.94	0.46	0.97
1702	KKILDIKGIDYWLAHKA	17	-0.11	0.61	0.61	-0.22	0.95	0.11	0.76	2.5	9.43	2012.69	0.99	0.48	0.95
1703	KILDIKGIDYWLAHKAL	17	-0.02	0.6	0.6	0.24	0.73	-0.18	0.65	1.5	8.76	1997.68	0.99	0.44	0.79
1704	ILDIKGIDYWLAHKALC	17	0.05	0.59	0.59	0.61	0.52	-0.41	0.53	0.5	7.06	1972.64	0.93	0.42	0.61
1705	LDIKGIDYWLAHKALCT	17	0	0.58	0.58	0.31	0.52	-0.33	0.59	0.5	7.06	1960.58	0.93	0.43	0.29
1706	DIKGIDYWLAHKALCTE	17	-0.07	0.59	0.59	-0.12	0.59	-0.05	0.65	-0.5	5.39	1976.53	0.94	0.46	0.13
1707	IKGIDYWLAHKALCTEK	17	-0.09	0.59	0.59	-0.15	0.81	-0.05	0.71	1.5	8.48	1989.62	0.97	0.47	0.73
1708	KGIDYWLAHKALCTEKL	17	-0.11	0.58	0.58	-0.19	0.81	-0.05	0.71	1.5	8.48	1989.62	0.98	0.47	0.78
1709	GIDYWLAHKALCTEKLE	17	-0.08	0.58	0.58	-0.16	0.67	-0.05	0.65	-0.5	5.46	1990.56	0.95	0.44	0.10
1710	IDYWLAHKALCTEKLEQ	17	-0.13	0.58	0.58	-0.35	0.74	-0.04	0.76	-0.5	5.46	2061.64	0.99	0.39	0.11
1711	DYWLAHKALCTEKLEQW	17	-0.15	0.57	0.57	-0.66	0.74	-0.13	0.82	-0.5	5.46	2134.69	0.86	0.39	0.55
1712	YWLAHKALCTEKLEQWL	17	-0.08	0.55	0.55	-0.24	0.74	-0.41	0.76	0.5	7.06	2132.77	0.99	0.38	0.91
1713	WLAHKALCTEKLEQWLC	17	-0.07	0.55	0.55	-0.01	0.74	-0.34	0.71	0.5	7.04	2072.73	0.97	0.33	0.90
1714	LAHKALCTEKLEQWLCE	17	-0.13	0.56	0.56	-0.16	0.81	0.04	0.71	-0.5	5.51	2015.63	0.93	0.34	0.51
1715	AHKALCTEKLEQWLCEK	17	-0.23	0.57	0.57	-0.62	1.03	0.32	0.82	0.5	7.04	2030.64	0.86	0.27	0.32
1716	HKALCTEKLEQWLCEKL	17	-0.21	0.57	0.57	-0.5	1.03	0.25	0.82	0.5	7.04	2072.73	0.98	0.23	0.77
1717	MRFFVPLFLVGILFPAIL	18	0.33	0.62	0.62	2.19	0.14	-1.26	0.22	1	10.11	2093.97	0.93	0.66	0.95
1718	RFFVPLFLVGILFPAILA	18	0.33	0.6	0.6	2.18	0.14	-1.21	0.22	1	10.11	2033.85	0.93	0.65	0.90
1719	FFVPLFLVGILFPAILAK	18	0.36	0.6	0.6	2.22	0.2	-1.21	0.11	1	9.11	2005.84	0.94	0.71	0.99
1720	FVPLFLVGILFPAILAKQ	18	0.29	0.6	0.6	1.87	0.27	-1.06	0.22	1	9.11	1986.8	0.94	0.65	0.98
1721	VPLFLVGILFPAILAKQF	18	0.29	0.6	0.6	1.87	0.27	-1.06	0.22	1	9.11	1986.8	0.98	0.62	0.98
1722	PLFLVGILFPAILAKQFT	18	0.25	0.59	0.59	1.59	0.27	-1	0.28	1	9.11	1988.77	0.98	0.56	0.89
1723	LFLVGILFPAILAKQFTK	18	0.19	0.61	0.61	1.47	0.48	-0.83	0.39	2	10.02	2019.83	0.98	0.55	0.95
1724	FLVGILFPAILAKQFTKC	18	0.17	0.61	0.61	1.39	0.48	-0.79	0.39	2	9.36	2009.8	0.94	0.55	0.98
1725	LVGILFPAILAKQFTKCE	18	0.1	0.61	0.61	1.04	0.55	-0.48	0.44	1	8.54	1991.74	0.93	0.50	0.89
1726	VGILFPAILAKQFTKCEL	18	0.1	0.61	0.61	1.04	0.55	-0.48	0.44	1	8.54	1991.74	0.93	0.49	0.96

1727	GILFPAILAKQFTKCELS	18	0.05	0.6	0.6	0.77	0.55	-0.38	0.5	1	8.54	1979.68	0.83	0.51	0.97
1728	ILFPAILAKQFTKCELSQ	18	0.01	0.6	0.6	0.59	0.62	-0.37	0.61	1	8.54	2050.76	0.93	0.53	0.63
1729	LFPAILAKQFTKCELSQL	18	-0.01	0.59	0.59	0.56	0.62	-0.37	0.61	1	8.54	2050.76	0.93	0.49	0.37
1730	FPAILAKQFTKCELSQLL	18	-0.01	0.59	0.59	0.56	0.62	-0.37	0.61	1	8.54	2050.76	0.94	0.46	0.85
1731	PAILAKQFTKCELSQLLK	18	-0.1	0.59	0.59	0.18	0.82	-0.07	0.72	2	9.25	2031.76	0.93	0.47	0.85
1732	AILAKQFTKCELSQLLKD	18	-0.14	0.62	0.62	0.08	0.82	0.1	0.78	1	8.51	2049.73	0.94	0.41	0.74
1733	ILAKQFTKCELSQLLKDI	18	-0.11	0.63	0.63	0.23	0.82	0.03	0.78	1	8.51	2091.82	0.99	0.41	0.55
1734	LAKQFTKCELSQLLKDID	18	-0.19	0.63	0.63	-0.22	0.82	0.29	0.83	0	6.43	2093.74	0.99	0.39	0.68
1735	AKQFTKCELSQLLKDIDG	18	-0.21	0.64	0.64	-0.45	0.82	0.39	0.83	0	6.43	2037.63	0.61	0.31	0.07
1736	KQFTKCELSQLLKDIDGY	18	-0.22	0.65	0.65	-0.62	0.82	0.29	0.89	0	6.43	2129.73	0.99	0.31	0.04
1737	QFTKCELSQLLKDIDGYG	18	-0.15	0.65	0.65	-0.43	0.62	0.13	0.78	-1	4.56	2058.61	0.94	0.31	0.04
1738	FTKCELSQLLKDIDGYGG	18	-0.11	0.65	0.65	-0.26	0.55	0.12	0.67	-1	4.56	1987.53	0.61	0.29	0.18
1739	TKCELSQLLKDIDGYGGI	18	-0.1	0.65	0.65	-0.16	0.55	0.16	0.67	-1	4.56	1953.52	0.99	0.33	0.03
1740	KCELSQLLKDIDGYGGIA	18	-0.08	0.65	0.65	-0.02	0.55	0.15	0.61	-1	4.56	1923.49	0.99	0.29	0.10
1741	CELSQLLKDIDGYGGIAL	18	0.01	0.64	0.64	0.41	0.34	-0.12	0.5	-2	4.03	1908.48	0.61	0.29	0.03
1742	ELSQLLKDIDGYGGIALP	18	0.01	0.62	0.62	0.18	0.34	-0.06	0.5	-2	4.03	1902.46	0.61	0.35	0.03
1743	LSQLLKDIDGYGGIALPE	18	0.01	0.62	0.62	0.18	0.34	-0.06	0.5	-2	4.03	1902.46	0.99	0.36	0.06
1744	SQLLKDIDGYGGIALPEL	18	0.01	0.62	0.62	0.18	0.34	-0.06	0.5	-2	4.03	1902.46	0.99	0.35	0.04
1745	QLLKDIDGYGGIALPELI	18	0.06	0.63	0.63	0.47	0.34	-0.18	0.44	-2	4.03	1928.55	0.94	0.34	0.03
1746	LLKDIDGYGGIALPELIC	18	0.1	0.63	0.63	0.81	0.27	-0.24	0.33	-2	4.03	1903.55	0.97	0.42	0.17
1747	LKDIDGYGGIALPELICT	18	0.06	0.63	0.63	0.56	0.27	-0.17	0.39	-2	4.03	1891.49	0.99	0.41	0.04
1748	KDIDGYGGIALPELICTM	18	0.05	0.64	0.64	0.45	0.27	-0.14	0.39	-2	4.03	1909.52	0.97	0.42	0.06
1749	DIDGYGGIALPELICTMF	18	0.14	0.64	0.64	0.82	0.07	-0.44	0.28	-3	3.5	1928.52	0.86	0.46	0.04
1750	IDGYGGIALPELICTMFH	18	0.16	0.6	0.6	0.84	0.15	-0.64	0.28	-1.5	4.36	1950.58	0.97	0.52	0.07
1751	DGYGGIALPELICTMFHT	18	0.11	0.59	0.59	0.55	0.15	-0.56	0.33	-1.5	4.36	1938.52	0.86	0.52	0.06
1752	GYGGIALPELICTMFHTS	18	0.14	0.58	0.58	0.7	0.15	-0.71	0.33	-0.5	5.25	1910.51	0.93	0.44	0.38
1753	YGGIALPELICTMFHTSG	18	0.14	0.58	0.58	0.7	0.15	-0.71	0.33	-0.5	5.25	1910.51	0.97	0.37	0.13
1754	GGIALPELICTMFHTSGY	18	0.14	0.58	0.58	0.7	0.15	-0.71	0.33	-0.5	5.25	1910.51	0.98	0.33	0.41
1755	GIALPELICTMFHTSGYD	18	0.09	0.58	0.58	0.53	0.15	-0.54	0.39	-1.5	4.36	1968.54	0.98	0.33	0.22
1756	IALPELICTMFHTSGYDT	18	0.07	0.58	0.58	0.51	0.15	-0.57	0.44	-1.5	4.36	2012.59	0.93	0.30	0.08
1757	ALPELICTMFHTSGYDTQ	18	-0.01	0.58	0.58	0.07	0.22	-0.46	0.56	-1.5	4.36	2027.56	0.80	0.31	0.06
1758	LPELICTMFHTSGYDTQA	18	-0.01	0.58	0.58	0.07	0.22	-0.46	0.56	-1.5	4.36	2027.56	0.93	0.32	0.02
1759	PELICTMFHTSGYDTQAI	18	0	0.58	0.58	0.11	0.22	-0.46	0.56	-1.5	4.36	2027.56	0.87	0.29	0.03
1760	ELICTMFHTSGYDTQAIV	18	0.04	0.6	0.6	0.43	0.22	-0.54	0.56	-1.5	4.36	2029.58	0.80	0.33	0.02
1761	LICTMFHTSGYDTQAIVE	18	0.04	0.6	0.6	0.43	0.22	-0.54	0.56	-1.5	4.36	2029.58	0.93	0.39	0.22
1762	ICTMFHTSGYDTQAIVEN	18	-0.03	0.62	0.62	0.02	0.22	-0.43	0.67	-1.5	4.36	2030.52	0.93	0.42	0.18
1763	CTMFHTSGYDTQAIVENN	18	-0.11	0.62	0.62	-0.42	0.22	-0.32	0.78	-1.5	4.36	2031.46	0.94	0.39	0.08
1764	TMFHTSGYDTQAIVENNE	18	-0.14	0.62	0.62	-0.76	0.29	-0.09	0.83	-2.5	4.14	2057.44	0.93	0.33	0.06
1765	MFHTSGYDTQAIVENNES	18	-0.15	0.62	0.62	-0.76	0.29	-0.06	0.83	-2.5	4.14	2043.41	0.93	0.32	0.04
1766	FHTSGYDTQAIVENNEST	18	-0.17	0.61	0.61	-0.91	0.29	-0.01	0.89	-2.5	4.14	2013.32	0.94	0.34	0.04
1767	HTSGYDTQAIVENNESTE	18	-0.24	0.61	0.61	-1.26	0.36	0.3	0.94	-3.5	4	1995.26	0.93	0.29	0.04
1768	TSGYDTQAIVENNESTEY	18	-0.22	0.65	0.65	-1.15	0.28	0.2	0.94	-4	3.51	2021.29	0.93	0.25	0.04
1769	SGYDTQAIVENNESTEYG	18	-0.2	0.66	0.66	-1.13	0.28	0.22	0.89	-4	3.51	1977.24	0.93	0.35	0.04
1770	GYDTQAIVENNESTEYGL	18	-0.15	0.66	0.66	-0.88	0.28	0.11	0.83	-4	3.51	2003.33	0.83	0.33	0.05
1771	YDTQAIVENNESTEYGLF	18	-0.13	0.66	0.66	-0.7	0.28	-0.03	0.83	-4	3.51	2093.45	0.98	0.38	0.05
1772	DTQAIVENNESTEYGLFQ	18	-0.17	0.66	0.66	-0.82	0.35	0.11	0.89	-4	3.51	2058.41	0.94	0.37	0.08
1773	TQAIVENNESTEYGLFQI	18	-0.09	0.65	0.65	-0.38	0.35	-0.16	0.83	-3	3.68	2056.49	0.98	0.41	0.08
1774	QAIVENNESTEYGLFQIS	18	-0.09	0.65	0.65	-0.38	0.35	-0.12	0.83	-3	3.68	2042.46	0.98	0.43	0.07
1775	AIVENNESTEYGLFQISN	18	-0.09	0.66	0.66	-0.38	0.28	-0.12	0.83	-3	3.68	2028.43	0.80	0.47	0.04
1776	IVENNESTEYGLFQISNK	18	-0.16	0.67	0.67	-0.7	0.49	0.07	0.94	-2	4.26	2085.53	0.94	0.42	0.04
1777	VENNESTEYGLFQISNKL	18	-0.18	0.66	0.66	-0.74	0.49	0.07	0.94	-2	4.26	2085.53	0.98	0.41	0.07
1778	ENNESTEYGLFQISNKLW	18	-0.18	0.64	0.64	-1.02	0.49	-0.03	1	-2	4.26	2172.61	0.61	0.38	0.10
1779	NNESTEYGLFQISNKLWC	18	-0.15	0.64	0.64	-0.69	0.41	-0.26	0.94	-1	4.54	2146.63	0.87	0.40	0.12
1780	NESTEYGLFQISNKLWCK	18	-0.17	0.64	0.64	-0.71	0.62	-0.1	0.94	0	6.45	2160.7	0.99	0.40	0.19
1781	ESTEYGLFQISNKLWCKS	18	-0.15	0.62	0.62	-0.56	0.62	-0.09	0.89	0	6.45	2133.67	0.61	0.46	0.10
1782	STEYGLFQISNKLWCKSS	18	-0.13	0.62	0.62	-0.41	0.55	-0.24	0.89	1	8.51	2091.63	0.97	0.50	0.24
1783	TEYGLFQISNKLWCKSSQ	18	-0.16	0.62	0.62	-0.56	0.62	-0.25	0.94	1	8.51	2132.69	0.97	0.53	0.06
1784	EYGLFQISNKLWCKSSQV	18	-0.12	0.63	0.63	-0.29	0.62	-0.31	0.89	1	8.51	2130.72	0.61	0.54	0.09
1785	YGLFQISNKLWCKSSQVP	18	-0.09	0.62	0.62	-0.18	0.55	-0.48	0.83	2	9.25	2098.72	0.99	0.54	0.28
1786	GLFQISNKLWCKSSQVPQ	18	-0.12	0.61	0.61	-0.31	0.62	-0.34	0.89	2	9.36	2063.68	0.98	0.53	0.78
1787	LFQISNKLWCKSSQVPQS	18	-0.15	0.61	0.61	-0.33	0.62	-0.32	0.94	2	9.36	2093.7	0.94	0.49	0.10
1788	FQISNKLWCKSSQVPQSR	18	-0.28	0.61	0.61	-0.79	0.75	-0.06	1.17	3	10.07	2136.72	0.61	0.43	0.07
1789	QISNKLWCKSSQVPQSRN	18	-0.34	0.62	0.62	-1.14	0.75	0.09	1.28	3	10.07	2103.65	0.99	0.44	0.04
1790	ISNKLWCKSSQVPQSRNI	18	-0.27	0.62	0.62	-0.69	0.68	-0.02	1.17	3	10.07	2088.68	0.99	0.42	0.07

1791	SNKLWCKSSQVPQSRNIC	18	-0.3	0.61	0.61	-0.81	0.68	0.03	1.17	3	9.53	2078.65	0.99	0.37	0.58
1792	NKLWCKSSQVPQSRNICD	18	-0.33	0.63	0.63	-0.96	0.68	0.18	1.17	2	8.98	2106.66	0.99	0.42	0.42
1793	KLWCKSSQVPQSRNICDI	18	-0.25	0.62	0.62	-0.51	0.68	0.07	1.06	2	8.98	2105.72	0.99	0.43	0.39
1794	LWCKSSQVPQSRNICDIS	18	-0.21	0.62	0.62	-0.34	0.48	-0.08	1	1	8.38	2064.62	0.97	0.46	0.43
1795	WCKSSQVPQSRNICDISC	18	-0.23	0.62	0.62	-0.41	0.48	-0.04	1	1	8.26	2054.59	0.97	0.43	0.81
1796	CKSSQVPQSRNICDISCD	18	-0.29	0.64	0.64	-0.56	0.48	0.32	1	0	6.23	1983.46	0.86	0.42	0.13
1797	KSSQVPQSRNICDISCDK	18	-0.36	0.64	0.64	-0.91	0.68	0.54	1.11	1	8.36	2008.5	0.99	0.43	0.06
1798	SSQVPQSRNICDISCDKF	18	-0.26	0.64	0.64	-0.54	0.48	0.23	1	0	6.25	2027.5	0.99	0.47	0.13
1799	SQVPQSRNICDISCDKFL	18	-0.22	0.64	0.64	-0.28	0.48	0.12	0.94	0	6.25	2053.59	0.99	0.49	0.47
1800	QVPQSRNICDISCDKFLD	18	-0.24	0.65	0.65	-0.43	0.48	0.27	0.94	-1	4.43	2081.6	0.99	0.37	0.22
1801	VPQSRNICDISCDKFLDD	18	-0.25	0.66	0.66	-0.43	0.41	0.42	0.89	-2	4.14	2068.55	0.98	0.34	0.14
1802	PQSRNICDISCDKFLDDD	18	-0.32	0.66	0.66	-0.86	0.41	0.67	0.94	-3	3.98	2084.5	0.87	0.33	0.11
1803	QSRNICDISCDKFLDDDI	18	-0.27	0.68	0.68	-0.52	0.41	0.57	0.94	-3	3.98	2100.55	0.98	0.38	0.15
1804	SRNICDISCDKFLDDDIT	18	-0.24	0.67	0.67	-0.37	0.34	0.54	0.89	-3	3.98	2073.52	0.98	0.40	0.08
1805	RNICDISCDKFLDDDITD	18	-0.27	0.68	0.68	-0.52	0.34	0.69	0.89	-4	3.86	2101.53	0.98	0.37	0.06
1806	NICDISCDKFLDDDITDD	18	-0.21	0.69	0.69	-0.46	0.2	0.69	0.72	-6	3.5	2060.43	0.99	0.37	0.03
1807	ICDISCDKFLDDDITDDI	18	-0.14	0.68	0.68	-0.02	0.2	0.58	0.61	-6	3.5	2059.49	0.99	0.35	0.07
1808	CDISCDKFLDDDITDDIM	18	-0.16	0.69	0.69	-0.16	0.2	0.61	0.61	-6	3.5	2077.52	0.61	0.33	0.03
1809	DISCDKFLDDDITDDIMC	18	-0.16	0.69	0.69	-0.16	0.2	0.61	0.61	-6	3.5	2077.52	0.61	0.34	0.06
1810	ISCDKFLDDDITDDIMCA	18	-0.11	0.68	0.68	0.13	0.2	0.41	0.56	-5	3.57	2033.51	0.99	0.34	0.06
1811	SCDKFLDDDITDDIMCAK	18	-0.21	0.67	0.67	-0.33	0.41	0.68	0.67	-4	3.86	2048.52	0.99	0.38	0.14
1812	CDKFLDDDITDDIMCAKK	18	-0.26	0.68	0.68	-0.51	0.61	0.83	0.72	-3	4.12	2089.62	0.61	0.39	0.16
1813	DKFLDDDITDDIMCAKKI	18	-0.22	0.69	0.69	-0.39	0.61	0.78	0.72	-3	4.12	2099.65	0.61	0.38	0.05
1814	KFLDDDITDDIMCAKKIL	18	-0.15	0.67	0.67	0.01	0.61	0.52	0.67	-2	4.29	2097.73	0.99	0.53	0.16
1815	FLDDDITDDIMCAKKILD	18	-0.13	0.68	0.68	0.03	0.41	0.52	0.61	-4	3.86	2084.64	0.99	0.49	0.04
1816	LDDDITDDIMCAKKILDI	18	-0.12	0.68	0.68	0.13	0.41	0.56	0.61	-4	3.86	2050.63	0.99	0.43	0.04
1817	DDDITDDIMCAKKILDIK	18	-0.21	0.69	0.69	-0.3	0.61	0.82	0.72	-3	4.12	2065.64	0.61	0.43	0.05
1818	DDITDDIMCAKKILDIKG	18	-0.16	0.68	0.68	-0.13	0.61	0.66	0.67	-2	4.29	2007.61	0.61	0.40	0.08
1819	DITDDIMCAKKILDIKGI	18	-0.08	0.68	0.68	0.32	0.61	0.39	0.61	-1	4.58	2005.69	0.61	0.38	0.18
1820	ITDDIMCAKKILDIKGID	18	-0.08	0.68	0.68	0.32	0.61	0.39	0.61	-1	4.58	2005.69	0.99	0.36	0.17
1821	TDDIMCAKKILDIKGIDY	18	-0.12	0.68	0.68	-0.01	0.61	0.36	0.67	-1	4.58	2055.7	0.99	0.37	0.05
1822	DDIMCAKKILDIKGIDYW	18	-0.09	0.68	0.68	-0.02	0.61	0.19	0.67	-1	4.58	2140.81	0.86	0.45	0.12
1823	DIMCAKKILDIKGIDYWL	18	-0.02	0.67	0.67	0.39	0.61	-0.07	0.61	0	6.35	2138.89	0.94	0.44	0.79
1824	IMCAKKILDIKGIDYWLA	18	0.03	0.65	0.65	0.68	0.61	-0.27	0.56	1	8.48	2094.88	0.93	0.44	0.97
1825	MCAKKILDIKGIDYWLAH	18	-0.03	0.61	0.61	0.26	0.69	-0.19	0.61	1.5	8.48	2118.86	0.93	0.45	0.98
1826	CAKKILDIKGIDYWLAHK	18	-0.11	0.61	0.61	-0.07	0.9	0.04	0.72	2.5	9.1	2115.84	0.69	0.46	0.98
1827	AKKILDIKGIDYWLAHKA	18	-0.09	0.6	0.6	-0.11	0.9	0.07	0.72	2.5	9.43	2083.78	0.80	0.52	0.98
1828	KKILDIKGIDYWLAHKAL	18	-0.08	0.6	0.6	0.01	0.9	0	0.72	2.5	9.43	2125.87	0.98	0.48	0.92
1829	KILDIKGIDYWLAHKALC	18	-0.02	0.6	0.6	0.36	0.69	-0.22	0.61	1.5	8.48	2100.83	0.98	0.41	0.87
1830	ILDIKGIDYWLAHKALCT	18	0.04	0.59	0.59	0.54	0.49	-0.41	0.56	0.5	7.06	2073.76	0.93	0.43	0.30
1831	LDIKGIDYWLAHKALCTE	18	-0.04	0.59	0.59	0.09	0.56	-0.14	0.61	-0.5	5.39	2089.71	0.93	0.41	0.08
1832	DIKGIDYWLAHKALCTEK	18	-0.13	0.6	0.6	-0.33	0.76	0.12	0.72	0.5	7.06	2104.72	0.94	0.46	0.30
1833	IKGIDYWLAHKALCTEKL	18	-0.06	0.59	0.59	0.07	0.76	-0.14	0.67	1.5	8.48	2102.8	0.98	0.47	0.75
1834	KGIDYWLAHKALCTEKLE	18	-0.14	0.58	0.58	-0.37	0.83	0.12	0.72	0.5	7.06	2118.75	0.98	0.47	0.10
1835	GIDYWLAHKALCTEKLEQ	18	-0.11	0.58	0.58	-0.35	0.7	-0.03	0.72	-0.5	5.46	2118.71	0.83	0.42	0.06
1836	IDYWLAHKALCTEKLEQW	18	-0.1	0.57	0.57	-0.38	0.7	-0.22	0.78	-0.5	5.46	2247.87	0.99	0.42	0.25
1837	DYWLAHKALCTEKLEQWL	18	-0.11	0.56	0.56	-0.42	0.7	-0.22	0.78	-0.5	5.46	2247.87	0.86	0.41	0.74
1838	YWLAHKALCTEKLEQWLC	18	-0.07	0.56	0.56	-0.08	0.7	-0.44	0.72	0.5	7.03	2235.92	0.97	0.41	0.91
1839	WLAHKALCTEKLEQWLCE	18	-0.1	0.56	0.56	-0.21	0.77	-0.15	0.72	-0.5	5.51	2201.86	0.97	0.35	0.69
1840	LAHKALCTEKLEQWLCEK	18	-0.19	0.57	0.57	-0.37	0.97	0.21	0.78	0.5	7.04	2143.82	0.99	0.37	0.42
1841	AHKALCTEKLEQWLCEKL	18	-0.19	0.57	0.57	-0.37	0.97	0.21	0.78	0.5	7.04	2143.82	0.94	0.30	0.77
1842	MRFFVPLFLVGILFPAILA	19	0.32	0.61	0.61	2.17	0.13	-1.22	0.21	1	10.11	2165.06	0.93	0.65	0.91
1843	RFFVPLFLVGILFPAILAK	19	0.25	0.61	0.61	1.86	0.32	-0.99	0.32	2	11.01	2162.04	0.98	0.72	0.98
1844	FFVPLFLVGILFPAILAKQ	19	0.31	0.61	0.61	1.92	0.26	-1.14	0.21	1	9.11	2133.99	0.94	0.66	0.98
1845	FVPLFLVGILFPAILAKQF	19	0.31	0.61	0.61	1.92	0.26	-1.14	0.21	1	9.11	2133.99	0.94	0.65	0.98
1846	VPLFLVGILFPAILAKQFT	19	0.27	0.6	0.6	1.73	0.26	-1.03	0.26	1	9.11	2087.92	0.98	0.57	0.75
1847	PLFLVGILFPAILAKQFTK	19	0.18	0.6	0.6	1.31	0.45	-0.79	0.37	2	10.02	2116.96	0.98	0.57	0.98
1848	LFLVGILFPAILAKQFTKC	19	0.18	0.61	0.61	1.52	0.45	-0.84	0.37	2	9.36	2122.98	0.93	0.58	0.99
1849	FLVGILFPAILAKQFTKCE	19	0.12	0.62	0.62	1.14	0.52	-0.59	0.42	1	8.54	2138.93	0.94	0.52	0.98
1850	LVGILFPAILAKQFTKCEL	19	0.12	0.61	0.61	1.19	0.52	-0.55	0.42	1	8.54	2104.92	0.93	0.44	0.70
1851	VGILFPAILAKQFTKCELS	19	0.08	0.61	0.61	0.95	0.52	-0.44	0.47	1	8.54	2078.83	0.93	0.47	0.93
1852	GILFPAILAKQFTKCELSQ	19	0.01	0.61	0.61	0.54	0.58	-0.35	0.58	1	8.54	2107.83	0.83	0.49	0.88
1853	ILFPAILAKQFTKCELSQL	19	0.03	0.6	0.6	0.76	0.58	-0.45	0.58	1	8.54	2163.94	0.93	0.50	0.42
1854	LFPAILAKQFTKCELSQLL	19	0.02	0.59	0.59	0.73	0.58	-0.45	0.58	1	8.54	2163.94	0.93	0.44	0.87

1855	FPAILAKQFTKCELSQLLK	19	-0.06	0.6	0.6	0.32	0.78	-0.19	0.68	2	9.25	2178.95	0.94	0.44	0.92
1856	PAILAKQFTKCELSQLLKD	19	-0.13	0.6	0.6	-0.01	0.78	0.09	0.74	1	8.51	2146.86	0.93	0.44	0.76
1857	AILAKQFTKCELSQLLKDI	19	-0.09	0.62	0.62	0.31	0.78	0	0.74	1	8.51	2162.91	0.94	0.38	0.62
1858	ILAKQFTKCELSQLLKDID	19	-0.14	0.63	0.63	0.03	0.78	0.18	0.79	0	6.43	2206.92	0.99	0.41	0.42
1859	LAKQFTKCELSQLLKDIDG	19	-0.17	0.63	0.63	-0.23	0.78	0.28	0.79	0	6.43	2150.81	0.99	0.39	0.35
1860	AKQFTKCELSQLLKDIDGY	19	-0.2	0.64	0.64	-0.49	0.78	0.25	0.84	0	6.43	2200.82	0.61	0.29	0.04
1861	KQFTKCELSQLLKDIDGYG	19	-0.2	0.65	0.65	-0.61	0.78	0.28	0.84	0	6.43	2186.8	0.99	0.32	0.10
1862	QFTKCELSQLLKDIDGYGG	19	-0.14	0.65	0.65	-0.43	0.58	0.12	0.74	-1	4.56	2115.68	0.94	0.30	0.05
1863	FTKCELSQLLKDIDGYGGI	19	-0.06	0.65	0.65	-0.01	0.52	0.02	0.63	-1	4.56	2100.71	0.61	0.28	0.12
1864	TKCELSQLLKDIDGYGGIA	19	-0.08	0.64	0.64	-0.06	0.52	0.12	0.63	-1	4.56	2024.61	0.99	0.31	0.06
1865	KCELSQLLKDIDGYGGIAL	19	-0.04	0.64	0.64	0.18	0.52	0.05	0.58	-1	4.56	2036.67	0.99	0.30	0.13
1866	CELSQLLKDIDGYGGIALP	19	0.01	0.62	0.62	0.3	0.33	-0.11	0.47	-2	4.03	2005.61	0.61	0.35	0.03
1867	ELSQLLKDIDGYGGIALPE	19	-0.02	0.63	0.63	-0.02	0.39	0.1	0.53	-3	3.92	2031.59	0.61	0.36	0.03
1868	LSQLLKDIDGYGGIALPEL	19	0.04	0.62	0.62	0.37	0.33	-0.15	0.47	-2	4.03	2015.64	0.99	0.36	0.04
1869	SQLLKDIDGYGGIALPELI	19	0.05	0.63	0.63	0.41	0.33	-0.15	0.47	-2	4.03	2015.64	0.99	0.34	0.04
1870	QLLKDIDGYGGIALPELIC	19	0.06	0.63	0.63	0.58	0.33	-0.22	0.42	-2	4.03	2031.7	0.94	0.34	0.04
1871	LLKDIDGYGGIALPELICT	19	0.09	0.62	0.62	0.73	0.26	-0.25	0.37	-2	4.03	2004.67	0.99	0.38	0.05
1872	LKDIDGYGGIALPELICTM	19	0.07	0.64	0.64	0.63	0.26	-0.23	0.37	-2	4.03	2022.7	0.97	0.43	0.03
1873	KDIDGYGGIALPELICTMF	19	0.08	0.65	0.65	0.57	0.26	-0.26	0.37	-2	4.03	2056.71	0.99	0.42	0.03
1874	DIDGYGGIALPELICTMFH	19	0.12	0.61	0.61	0.61	0.14	-0.45	0.32	-2.5	4.03	2065.68	0.86	0.50	0.06
1875	IDGYGGIALPELICTMFHT	19	0.14	0.6	0.6	0.76	0.14	-0.63	0.32	-1.5	4.36	2051.7	0.97	0.53	0.05
1876	DGYGGIALPELICTMFHTS	19	0.09	0.59	0.59	0.48	0.14	-0.52	0.37	-1.5	4.36	2025.61	0.86	0.44	0.07
1877	GYGGIALPELICTMFHTSG	19	0.14	0.59	0.59	0.64	0.14	-0.67	0.32	-0.5	5.25	1967.58	0.98	0.34	0.41
1878	YGGIALPELICTMFHTSGY	19	0.13	0.59	0.59	0.59	0.14	-0.79	0.37	-0.5	5.25	2073.7	0.99	0.34	0.11
1879	GGIALPELICTMFHTSGYD	19	0.09	0.59	0.59	0.48	0.14	-0.52	0.37	-1.5	4.36	2025.61	0.98	0.32	0.20
1880	GIALPELICTMFHTSGYDT	19	0.07	0.58	0.58	0.46	0.14	-0.54	0.42	-1.5	4.36	2069.66	0.95	0.30	0.09
1881	IALPELICTMFHTSGYDTQ	19	0.03	0.58	0.58	0.3	0.21	-0.53	0.53	-1.5	4.36	2140.74	0.93	0.29	0.05
1882	ALPELICTMFHTSGYDTQA	19	0	0.57	0.57	0.16	0.21	-0.46	0.53	-1.5	4.36	2098.65	0.80	0.29	0.03
1883	LPELICTMFHTSGYDTQAI	19	0.03	0.58	0.58	0.3	0.21	-0.53	0.53	-1.5	4.36	2140.74	0.98	0.29	0.03
1884	PELICTMFHTSGYDTQAIV	19	0.03	0.59	0.59	0.32	0.21	-0.51	0.53	-1.5	4.36	2126.71	0.87	0.29	0.04
1885	ELICTMFHTSGYDTQAIVE	19	0	0.61	0.61	0.22	0.28	-0.35	0.58	-2.5	4.14	2158.71	0.80	0.34	0.03
1886	LICTMFHTSGYDTQAIVEN	19	0	0.61	0.61	0.22	0.21	-0.5	0.63	-1.5	4.36	2143.7	0.93	0.40	0.05
1887	ICTMFHTSGYDTQAIVENN	19	-0.06	0.62	0.62	-0.16	0.21	-0.39	0.74	-1.5	4.36	2144.64	0.93	0.40	0.22
1888	CTMFHTSGYDTQAIVENNE	19	-0.13	0.62	0.62	-0.58	0.28	-0.14	0.79	-2.5	4.14	2160.59	0.94	0.34	0.08
1889	TMFHTSGYDTQAIVENNES	19	-0.15	0.62	0.62	-0.76	0.28	-0.07	0.84	-2.5	4.14	2144.53	0.93	0.34	0.06
1890	MFHTSGYDTQAIVENNEST	19	-0.15	0.62	0.62	-0.76	0.28	-0.07	0.84	-2.5	4.14	2144.53	0.93	0.34	0.04
1891	FHTSGYDTQAIVENNESTE	19	-0.19	0.61	0.61	-1.04	0.34	0.15	0.89	-3.5	4	2142.45	0.94	0.34	0.03
1892	HTSGYDTQAIVENNESTEY	19	-0.23	0.61	0.61	-1.26	0.34	0.16	0.95	-3.5	4	2158.45	0.98	0.30	0.04
1893	TSGYDTQAIVENNESTEYG	19	-0.2	0.65	0.65	-1.11	0.27	0.19	0.89	-4	3.51	2078.36	0.93	0.29	0.03
1894	SGYDTQAIVENNESTEYGL	19	-0.16	0.65	0.65	-0.87	0.27	0.12	0.84	-4	3.51	2090.42	0.93	0.32	0.04
1895	GYDTQAIVENNESTEYGLF	19	-0.11	0.66	0.66	-0.68	0.27	-0.03	0.79	-4	3.51	2150.52	0.83	0.34	0.03
1896	YDTQAIVENNESTEYGLFQ	19	-0.16	0.66	0.66	-0.85	0.33	-0.02	0.89	-4	3.51	2221.6	0.93	0.34	0.05
1897	DTQAIVENNESTEYGLFQI	19	-0.12	0.66	0.66	-0.54	0.33	0.01	0.84	-4	3.51	2171.59	0.94	0.37	0.08
1898	TQAIVENNESTEYGLFQIS	19	-0.1	0.65	0.65	-0.4	0.33	-0.14	0.84	-3	3.68	2143.58	0.98	0.42	0.15
1899	QAIVENNESTEYGLFQISN	19	-0.12	0.66	0.66	-0.55	0.33	-0.11	0.89	-3	3.68	2156.58	0.98	0.40	0.05
1900	AIVENNESTEYGLFQISNK	19	-0.14	0.66	0.66	-0.57	0.46	0.04	0.89	-2	4.26	2156.62	0.80	0.44	0.04
1901	IVENNESTEYGLFQISNKL	19	-0.13	0.66	0.66	-0.46	0.46	-0.03	0.89	-2	4.26	2198.71	0.87	0.44	0.07
1902	VENNESTEYGLFQISNKLW	19	-0.15	0.65	0.65	-0.75	0.46	-0.11	0.95	-2	4.26	2271.76	0.98	0.40	0.07
1903	ENNESTEYGLFQISNKLWC	19	-0.17	0.64	0.64	-0.84	0.46	-0.08	0.95	-2	4.26	2275.76	0.80	0.39	0.08
1904	NNESTEYGLFQISNKLWCK	19	-0.2	0.64	0.64	-0.86	0.59	-0.08	1	0	6.45	2274.82	0.94	0.37	0.23
1905	NESTEYGLFQISNKLWCKS	19	-0.18	0.63	0.63	-0.72	0.59	-0.08	0.95	0	6.45	2247.79	0.99	0.40	0.10
1906	ESTEYGLFQISNKLWCKSS	19	-0.16	0.62	0.62	-0.57	0.59	-0.07	0.89	0	6.45	2220.76	0.61	0.49	0.08
1907	STEYGLFQISNKLWCKSSQ	19	-0.16	0.62	0.62	-0.57	0.58	-0.22	0.95	1	8.51	2219.78	0.97	0.50	0.11
1908	TEYGLFQISNKLWCKSSQV	19	-0.12	0.63	0.63	-0.31	0.58	-0.32	0.89	1	8.51	2231.84	0.99	0.53	0.05
1909	EYGLFQISNKLWCKSSQVP	19	-0.11	0.62	0.62	-0.36	0.58	-0.29	0.84	1	8.51	2227.85	0.61	0.54	0.16
1910	YGLFQISNKLWCKSSQVPQ	19	-0.12	0.62	0.62	-0.36	0.58	-0.44	0.89	2	9.25	2226.87	0.99	0.53	0.13
1911	GLFQISNKLWCKSSQVPQS	19	-0.13	0.61	0.61	-0.33	0.58	-0.31	0.89	2	9.36	2150.77	0.98	0.50	0.57
1912	LFQISNKLWCKSSQVPQSR	19	-0.23	0.61	0.61	-0.55	0.71	-0.15	1.11	3	10.07	2249.9	0.94	0.48	0.10
1913	FQISNKLWCKSSQVPQSRN	19	-0.29	0.62	0.62	-0.93	0.71	-0.04	1.21	3	10.07	2250.84	0.61	0.45	0.06
1914	QISNKLWCKSSQVPQSRNI	19	-0.29	0.62	0.62	-0.84	0.71	-0.01	1.21	3	10.07	2216.83	0.99	0.46	0.06
1915	ISNKLWCKSSQVPQSRNIC	19	-0.25	0.62	0.62	-0.53	0.65	-0.07	1.11	3	9.53	2191.83	0.99	0.46	0.45
1916	SNKLWCKSSQVPQSRNICD	19	-0.33	0.62	0.62	-0.95	0.65	0.18	1.16	2	8.98	2193.75	0.99	0.44	0.18
1917	NKLWCKSSQVPQSRNICDI	19	-0.27	0.63	0.63	-0.67	0.65	0.07	1.11	2	8.98	2219.84	0.99	0.45	0.16
1918	KLWCKSSQVPQSRNICDIS	19	-0.25	0.62	0.62	-0.53	0.65	0.08	1.05	2	8.98	2192.81	0.99	0.47	0.52

1919	LWCKSSQVPQSRNICDISC	19	-0.19	0.62	0.62	-0.19	0.45	-0.13	0.95	1	8.26	2167.77	0.97	0.44	0.75
1920	WCKSSQVPQSRNICDISCD	19	-0.26	0.63	0.63	-0.57	0.45	0.12	1	0	6.23	2169.69	0.97	0.43	0.56
1921	CKSSQVPQSRNICDISCDK	19	-0.34	0.64	0.64	-0.73	0.65	0.46	1.05	1	8.24	2111.65	0.86	0.40	0.11
1922	KSSQVPQSRNICDISCDKF	19	-0.31	0.64	0.64	-0.72	0.65	0.38	1.05	1	8.36	2155.69	0.99	0.46	0.11
1923	SSQVPQSRNICDISCDKFL	19	-0.22	0.63	0.63	-0.31	0.45	0.13	0.95	0	6.25	2140.68	0.99	0.47	0.27
1924	SQVPQSRNICDISCDKFLD	19	-0.25	0.65	0.65	-0.45	0.45	0.27	0.95	-1	4.43	2168.69	0.99	0.36	0.18
1925	QVPQSRNICDISCDKFLDD	19	-0.27	0.66	0.66	-0.59	0.45	0.41	0.95	-2	4.14	2196.7	0.99	0.34	0.10
1926	VPQSRNICDISCDKFLDDD	19	-0.27	0.66	0.66	-0.59	0.39	0.56	0.89	-3	3.98	2183.65	0.98	0.33	0.07
1927	PQSRNICDISCDKFLDDDI	19	-0.26	0.66	0.66	-0.58	0.39	0.54	0.89	-3	3.98	2197.68	0.87	0.33	0.13
1928	QSRNICDISCDKFLDDDIT	19	-0.27	0.67	0.67	-0.53	0.39	0.52	0.95	-3	3.98	2201.67	0.98	0.37	0.09
1929	SRNICDISCDKFLDDDITD	19	-0.27	0.68	0.68	-0.53	0.32	0.67	0.89	-4	3.86	2188.62	0.98	0.36	0.06
1930	RNICDISCDKFLDDDITDD	19	-0.29	0.69	0.69	-0.67	0.32	0.81	0.89	-5	3.77	2216.63	0.99	0.36	0.04
1931	NICDISCDKFLDDDITDDI	19	-0.16	0.69	0.69	-0.2	0.19	0.56	0.68	-6	3.5	2173.61	0.99	0.36	0.04
1932	ICDISCDKFLDDDITDDIM	19	-0.11	0.69	0.69	0.08	0.19	0.48	0.58	-6	3.5	2190.7	0.99	0.32	0.08
1933	CDISCDKFLDDDITDDIMC	19	-0.15	0.69	0.69	-0.02	0.19	0.52	0.58	-6	3.5	2180.67	0.61	0.33	0.05
1934	DISCDKFLDDDITDDIMCA	19	-0.14	0.68	0.68	-0.06	0.19	0.55	0.58	-6	3.5	2148.61	0.61	0.33	0.03
1935	ISCDKFLDDDITDDIMCAK	19	-0.16	0.68	0.68	-0.08	0.39	0.55	0.63	-4	3.86	2161.7	0.99	0.34	0.14
1936	SCDKFLDDDITDDIMCAKK	19	-0.26	0.68	0.68	-0.52	0.58	0.8	0.74	-3	4.12	2176.71	0.98	0.36	0.28
1937	CDKFLDDDITDDIMCAKKI	19	-0.2	0.68	0.68	-0.24	0.58	0.69	0.68	-3	4.12	2202.8	0.61	0.35	0.05
1938	DKFLDDDITDDIMCAKKIL	19	-0.18	0.68	0.68	-0.17	0.58	0.65	0.68	-3	4.12	2212.83	0.61	0.39	0.08
1939	KFLDDDITDDIMCAKKILD	19	-0.18	0.68	0.68	-0.17	0.58	0.65	0.68	-3	4.12	2212.83	0.98	0.48	0.03
1940	FLDDDITDDIMCAKKILDI	19	-0.08	0.68	0.68	0.27	0.39	0.39	0.58	-4	3.86	2197.82	0.61	0.46	0.03
1941	LDDDITDDIMCAKKILDIK	19	-0.17	0.68	0.68	-0.08	0.58	0.68	0.68	-3	4.12	2178.82	0.99	0.38	0.04
1942	DDDITDDIMCAKKILDIKG	19	-0.19	0.69	0.69	-0.31	0.58	0.78	0.68	-3	4.12	2122.71	0.61	0.36	0.05
1943	DDITDDIMCAKKILDIKGI	19	-0.12	0.68	0.68	0.12	0.58	0.53	0.63	-2	4.29	2120.79	0.61	0.34	0.09
1944	DITDDIMCAKKILDIKGID	19	-0.12	0.68	0.68	0.12	0.58	0.53	0.63	-2	4.29	2120.79	0.61	0.32	0.08
1945	ITDDIMCAKKILDIKGIDY	19	-0.08	0.68	0.68	0.23	0.58	0.25	0.63	-1	4.58	2168.88	0.99	0.33	0.08
1946	TDDIMCAKKILDIKGIDYW	19	-0.1	0.67	0.67	-0.05	0.58	0.16	0.68	-1	4.58	2241.93	0.98	0.42	0.05
1947	DDIMCAKKILDIKGIDYWL	19	-0.06	0.67	0.67	0.18	0.58	0.09	0.63	-1	4.58	2253.99	0.94	0.44	0.11
1948	DIMCAKKILDIKGIDYWLA	19	-0.01	0.66	0.66	0.46	0.58	-0.09	0.58	0	6.35	2209.98	0.94	0.45	0.92
1949	IMCAKKILDIKGIDYWLAH	19	0.01	0.62	0.62	0.48	0.66	-0.28	0.58	1.5	8.48	2232.04	0.93	0.47	0.98
1950	MCAKKILDIKGIDYWLAHK	19	-0.09	0.62	0.62	0.04	0.85	-0.03	0.68	2.5	9.1	2247.05	0.93	0.48	0.97
1951	CAKKILDIKGIDYWLAHKA	19	-0.09	0.6	0.6	0.03	0.85	0.02	0.68	2.5	9.1	2186.93	0.69	0.54	0.98
1952	AKKILDIKGIDYWLAHKAL	19	-0.06	0.6	0.6	0.1	0.85	-0.03	0.68	2.5	9.43	2196.96	0.86	0.53	0.98
1953	KKILDIKGIDYWLAHKALC	19	-0.07	0.6	0.6	0.14	0.85	-0.05	0.68	2.5	9.1	2229.02	0.98	0.43	0.97
1954	KILDIKGIDYWLAHKALCT	19	-0.02	0.6	0.6	0.31	0.66	-0.23	0.63	1.5	8.48	2201.95	0.98	0.44	0.60
1955	ILDIKGIDYWLAHKALCTE	19	0	0.6	0.6	0.33	0.53	-0.23	0.58	-0.5	5.39	2202.89	0.98	0.43	0.10
1956	LDIKGIDYWLAHKALCTEK	19	-0.09	0.59	0.59	-0.12	0.72	0.02	0.68	0.5	7.06	2217.9	0.98	0.42	0.25
1957	DIKGIDYWLAHKALCTEKL	19	-0.09	0.59	0.59	-0.12	0.72	0.02	0.68	0.5	7.06	2217.9	0.94	0.48	0.26
1958	IKGIDYWLAHKALCTEKLE	19	-0.09	0.59	0.59	-0.12	0.79	0.02	0.68	0.5	7.06	2231.93	0.98	0.46	0.10
1959	KGIDYWLAHKALCTEKLEQ	19	-0.16	0.59	0.59	-0.54	0.86	0.13	0.79	0.5	7.06	2246.9	0.98	0.43	0.09
1960	GIDYWLAHKALCTEKLEQW	19	-0.09	0.58	0.58	-0.38	0.66	-0.21	0.74	-0.5	5.46	2304.94	0.83	0.42	0.16
1961	IDYWLAHKALCTEKLEQWL	19	-0.07	0.57	0.57	-0.16	0.66	-0.31	0.74	-0.5	5.46	2361.05	0.98	0.41	0.45
1962	DYWLAHKALCTEKLEQWLC	19	-0.1	0.57	0.57	-0.26	0.66	-0.26	0.74	-0.5	5.46	2351.02	0.86	0.41	0.74
1963	YWLAHKALCTEKLEQWLCE	19	-0.1	0.56	0.56	-0.26	0.73	-0.26	0.74	-0.5	5.51	2365.05	0.98	0.39	0.74
1964	WLAHKALCTEKLEQWLCEK	19	-0.16	0.56	0.56	-0.4	0.92	0.02	0.79	0.5	7.04	2330.05	0.99	0.35	0.68
1965	LAHKALCTEKLEQWLCEKL	19	-0.15	0.56	0.56	-0.15	0.92	0.1	0.74	0.5	7.04	2257	0.98	0.38	0.79
1966	MRFFVPLFLVGILFPAILAK	20	0.25	0.61	0.61	1.86	0.31	-1.01	0.3	2	11.01	2293.25	0.93	0.70	0.99
1967	RFFVPLFLVGILFPAILAKQ	20	0.2	0.61	0.61	1.59	0.37	-0.93	0.4	2	11.01	2290.19	0.98	0.66	0.97
1968	FFVPLFLVGILFPAILAKQF	20	0.32	0.61	0.61	1.96	0.25	-1.21	0.2	1	9.11	2281.18	0.94	0.65	0.99
1969	FVPLFLVGILFPAILAKQFT	20	0.28	0.6	0.6	1.78	0.25	-1.1	0.25	1	9.11	2235.11	0.94	0.59	0.95
1970	VPLFLVGILFPAILAKQFTK	20	0.2	0.6	0.6	1.45	0.43	-0.82	0.35	2	10.02	2216.11	0.98	0.56	0.98
1971	PLFLVGILFPAILAKQFTKC	20	0.17	0.6	0.6	1.37	0.43	-0.8	0.35	2	9.36	2220.11	0.98	0.56	0.98
1972	LFLVGILFPAILAKQFTKCE	20	0.14	0.61	0.61	1.27	0.49	-0.65	0.4	1	8.54	2252.11	0.93	0.55	0.85
1973	FLVGILFPAILAKQFTKCEL	20	0.14	0.61	0.61	1.27	0.49	-0.65	0.4	1	8.54	2252.11	0.94	0.46	0.92
1974	LVGILFPAILAKQFTKCELS	20	0.1	0.6	0.6	1.09	0.49	-0.51	0.45	1	8.54	2192.01	0.93	0.44	0.56
1975	VGILFPAILAKQFTKCELSQ	20	0.04	0.61	0.61	0.73	0.56	-0.41	0.55	1	8.54	2206.98	0.93	0.48	0.74
1976	GILFPAILAKQFTKCELSQL	20	0.04	0.6	0.6	0.71	0.56	-0.42	0.55	1	8.54	2221.01	0.83	0.49	0.78
1977	ILFPAILAKQFTKCELSQLL	20	0.06	0.6	0.6	0.92	0.56	-0.52	0.55	1	8.54	2277.12	0.93	0.47	0.83
1978	LFPAILAKQFTKCELSQLLK	20	-0.03	0.6	0.6	0.49	0.74	-0.28	0.65	2	9.25	2292.13	0.93	0.44	0.90
1979	FPAILAKQFTKCELSQLLKD	20	-0.1	0.61	0.61	0.13	0.74	-0.03	0.7	1	8.51	2294.05	0.94	0.44	0.84
1980	PAILAKQFTKCELSQLLKDI	20	-0.09	0.61	0.61	0.21	0.74	0	0.7	1	8.51	2260.04	0.93	0.42	0.54
1981	AILAKQFTKCELSQLLKDID	20	-0.12	0.63	0.63	0.12	0.74	0.15	0.75	0	6.43	2278.01	0.94	0.41	0.47
1982	ILAKQFTKCELSQLLKDIDG	20	-0.13	0.63	0.63	0.01	0.74	0.18	0.75	0	6.43	2263.99	0.99	0.43	0.11

1983	LAKQFTKCELSQLLKDIDGY	20	-0.16	0.63	0.63	-0.28	0.74	0.15	0.8	0	6.43	2314	0.99	0.36	0.26
1984	AKQFTKCELSQLLKDIDGYG	20	-0.18	0.64	0.64	-0.49	0.74	0.24	0.8	0	6.43	2257.89	0.61	0.31	0.04
1985	KQFTKCELSQLLKDIDGYGG	20	-0.19	0.65	0.65	-0.6	0.74	0.27	0.8	0	6.43	2243.87	0.99	0.33	0.05
1986	QFTKCELSQLLKDIDGYGGI	20	-0.09	0.65	0.65	-0.18	0.56	0.03	0.7	-1	4.56	2228.86	0.94	0.31	0.03
1987	FTKCELSQLLKDIDGYGGIA	20	-0.05	0.64	0.64	0.08	0.49	-0.01	0.6	-1	4.56	2171.8	0.61	0.29	0.22
1988	TKCELSQLLKDIDGYGGIAL	20	-0.05	0.63	0.63	0.13	0.49	0.02	0.6	-1	4.56	2137.79	0.99	0.35	0.05
1989	KCELSQLLKDIDGYGGIALP	20	-0.05	0.63	0.63	0.09	0.49	0.05	0.55	-1	4.56	2133.8	0.98	0.37	0.12
1990	CELSQLLKDIDGYGGIALPE	20	-0.02	0.63	0.63	0.11	0.37	0.04	0.5	-3	3.92	2134.74	0.61	0.38	0.03
1991	ELSQLLKDIDGYGGIALPEL	20	0	0.62	0.62	0.17	0.37	0	0.5	-3	3.92	2144.77	0.61	0.39	0.03
1992	LSQLLKDIDGYGGIALPELI	20	0.07	0.62	0.62	0.57	0.31	-0.23	0.45	-2	4.03	2128.82	0.99	0.38	0.03
1993	SQLLKDIDGYGGIALPELIC	20	0.05	0.63	0.63	0.51	0.31	-0.2	0.45	-2	4.03	2118.79	0.99	0.37	0.10
1994	QLLKDIDGYGGIALPELICT	20	0.05	0.63	0.63	0.51	0.31	-0.23	0.45	-2	4.03	2132.82	0.94	0.35	0.04
1995	LLKDIDGYGGIALPELICTM	20	0.1	0.63	0.63	0.79	0.25	-0.31	0.35	-2	4.03	2135.88	0.97	0.43	0.12
1996	LKDIDGYGGIALPELICTMF	20	0.1	0.64	0.64	0.73	0.25	-0.34	0.35	-2	4.03	2169.89	0.97	0.43	0.03
1997	KDIDGYGGIALPELICTMFH	20	0.06	0.61	0.61	0.38	0.32	-0.27	0.4	-1.5	4.54	2193.87	0.97	0.46	0.03
1998	DIDGYGGIALPELICTMFHT	20	0.1	0.61	0.61	0.55	0.14	-0.45	0.35	-2.5	4.03	2166.8	0.86	0.52	0.05
1999	IDGYGGIALPELICTMFHTS	20	0.12	0.6	0.6	0.68	0.14	-0.58	0.35	-1.5	4.36	2138.79	0.97	0.44	0.05
2000	DGYGGIALPELICTMFHTSG	20	0.1	0.59	0.59	0.44	0.14	-0.49	0.35	-1.5	4.36	2082.68	0.86	0.32	0.07
2001	GYGGIALPELICTMFHTSGY	20	0.13	0.59	0.59	0.55	0.14	-0.75	0.35	-0.5	5.25	2130.77	0.98	0.32	0.37
2002	YGGIALPELICTMFHTSGYD	20	0.09	0.6	0.6	0.39	0.14	-0.6	0.4	-1.5	4.36	2188.8	0.98	0.32	0.10
2003	GGIALPELICTMFHTSGYDT	20	0.08	0.59	0.59	0.42	0.14	-0.51	0.4	-1.5	4.36	2126.73	0.95	0.29	0.09
2004	GIALPELICTMFHTSGYDTQ	20	0.04	0.59	0.59	0.26	0.2	-0.5	0.5	-1.5	4.36	2197.81	0.95	0.29	0.16
2005	IALPELICTMFHTSGYDTQA	20	0.04	0.58	0.58	0.38	0.2	-0.52	0.5	-1.5	4.36	2211.83	0.93	0.27	0.04
2006	ALPELICTMFHTSGYDTQAI	20	0.04	0.58	0.58	0.38	0.2	-0.53	0.5	-1.5	4.36	2211.83	0.80	0.27	0.03
2007	LPELICTMFHTSGYDTQAIV	20	0.06	0.59	0.59	0.49	0.2	-0.57	0.5	-1.5	4.36	2239.89	0.93	0.29	0.03
2008	PELICTMFHTSGYDTQAIVE	20	0	0.6	0.6	0.13	0.26	-0.33	0.55	-2.5	4.14	2255.84	0.87	0.30	0.03
2009	ELICTMFHTSGYDTQAIVEN	20	-0.03	0.62	0.62	0.04	0.26	-0.32	0.65	-2.5	4.14	2272.83	0.80	0.33	0.03
2010	LICTMFHTSGYDTQAIVENN	20	-0.03	0.62	0.62	0.03	0.2	-0.47	0.7	-1.5	4.36	2257.82	0.93	0.37	0.08
2011	ICTMFHTSGYDTQAIVENNE	20	-0.09	0.63	0.63	-0.33	0.26	-0.23	0.75	-2.5	4.14	2273.77	0.93	0.34	0.17
2012	CTMFHTSGYDTQAIVENNES	20	-0.14	0.62	0.62	-0.6	0.26	-0.12	0.8	-2.5	4.14	2247.68	0.94	0.32	0.07
2013	TMFHTSGYDTQAIVENNEST	20	-0.15	0.61	0.61	-0.76	0.26	-0.09	0.85	-2.5	4.14	2245.65	0.93	0.35	0.05
2014	MFHTSGYDTQAIVENNESTE	20	-0.17	0.62	0.62	-0.9	0.33	0.08	0.85	-3.5	4	2273.66	0.93	0.33	0.04
2015	FHTSGYDTQAIVENNESTEY	20	-0.18	0.62	0.62	-1.06	0.33	0.03	0.9	-3.5	4	2305.64	0.94	0.33	0.04
2016	HTSGYDTQAIVENNESTEYG	20	-0.21	0.62	0.62	-1.21	0.33	0.16	0.9	-3.5	4	2215.52	0.98	0.31	0.03
2017	TSGYDTQAIVENNESTEYGL	20	-0.16	0.64	0.64	-0.86	0.25	0.09	0.85	-4	3.51	2191.54	0.93	0.26	0.03
2018	SGYDTQAIVENNESTEYGLF	20	-0.12	0.65	0.65	-0.69	0.25	-0.01	0.8	-4	3.51	2237.61	0.93	0.31	0.05
2019	GYDTQAIVENNESTEYGLFQ	20	-0.14	0.66	0.66	-0.82	0.32	-0.02	0.85	-4	3.51	2278.67	0.83	0.30	0.05
2020	YDTQAIVENNESTEYGLFQI	20	-0.11	0.66	0.66	-0.58	0.32	-0.11	0.85	-4	3.51	2334.78	0.93	0.36	0.06
2021	DTQAIVENNESTEYGLFQIS	20	-0.13	0.65	0.65	-0.55	0.32	0.02	0.85	-4	3.51	2258.68	0.94	0.39	0.08
2022	TQAIVENNESTEYGLFQISN	20	-0.12	0.65	0.65	-0.56	0.32	-0.12	0.9	-3	3.68	2257.7	0.98	0.37	0.04
2023	QAIVENNESTEYGLFQISNK	20	-0.17	0.66	0.66	-0.72	0.5	0.05	0.95	-2	4.26	2284.77	0.98	0.35	0.04
2024	AIVENNESTEYGLFQISNKL	20	-0.11	0.65	0.65	-0.35	0.44	-0.05	0.85	-2	4.26	2269.8	0.80	0.43	0.06
2025	IVENNESTEYGLFQISNKLW	20	-0.1	0.65	0.65	-0.49	0.44	-0.19	0.9	-2	4.26	2384.94	0.94	0.40	0.06
2026	VENNESTEYGLFQISNKLWC	20	-0.14	0.65	0.65	-0.58	0.44	-0.15	0.9	-2	4.26	2374.91	0.98	0.39	0.06
2027	ENNESTEYGLFQISNKLWCK	20	-0.22	0.65	0.65	-0.99	0.62	0.07	1	-1	4.79	2403.95	0.61	0.37	0.09
2028	NNESTEYGLFQISNKLWCKS	20	-0.2	0.64	0.64	-0.85	0.56	-0.06	1	0	6.45	2361.91	0.94	0.39	0.13
2029	NESTEYGLFQISNKLWCKSS	20	-0.18	0.63	0.63	-0.72	0.56	-0.06	0.95	0	6.45	2334.88	0.99	0.45	0.05
2030	ESTEYGLFQISNKLWCKSSQ	20	-0.18	0.62	0.62	-0.72	0.62	-0.06	0.95	0	6.45	2348.91	0.61	0.50	0.04
2031	STEYGLFQISNKLWCKSSQV	20	-0.13	0.62	0.62	-0.34	0.56	-0.29	0.9	1	8.51	2318.93	0.97	0.52	0.04
2032	TEYGLFQISNKLWCKSSQVP	20	-0.12	0.61	0.61	-0.38	0.56	-0.3	0.85	1	8.51	2328.97	0.99	0.52	0.04
2033	EYGLFQISNKLWCKSSQVPQ	20	-0.14	0.62	0.62	-0.52	0.62	-0.27	0.9	1	8.51	2356	0.61	0.52	0.09
2034	YGLFQISNKLWCKSSQVPQS	20	-0.12	0.61	0.61	-0.38	0.55	-0.4	0.9	2	9.25	2313.96	0.99	0.49	0.05
2035	GLFQISNKLWCKSSQVPQSR	20	-0.21	0.61	0.61	-0.54	0.68	-0.14	1.05	3	10.07	2306.97	0.98	0.48	0.61
2036	LFQISNKLWCKSSQVPQSRN	20	-0.25	0.62	0.62	-0.69	0.68	-0.13	1.15	3	10.07	2364.02	0.94	0.46	0.08
2037	FQISNKLWCKSSQVPQSRNI	20	-0.24	0.63	0.63	-0.66	0.68	-0.13	1.15	3	10.07	2364.02	0.61	0.44	0.07
2038	QISNKLWCKSSQVPQSRNIC	20	-0.27	0.62	0.62	-0.68	0.68	-0.05	1.15	3	9.53	2319.98	0.99	0.47	0.49
2039	ISNKLWCKSSQVPQSRNICD	20	-0.27	0.63	0.63	-0.68	0.61	0.08	1.1	2	8.98	2306.93	0.97	0.48	0.14
2040	SNKLWCKSSQVPQSRNICDI	20	-0.27	0.63	0.63	-0.68	0.61	0.08	1.1	2	8.98	2306.93	0.99	0.44	0.13
2041	NKLWCKSSQVPQSRNICDIS	20	-0.27	0.63	0.63	-0.68	0.61	0.09	1.1	2	8.98	2306.93	0.99	0.49	0.25
2042	KLWCKSSQVPQSRNICDISC	20	-0.24	0.62	0.62	-0.38	0.61	0.03	1	2	8.77	2295.96	0.99	0.44	0.82
2043	LWCKSSQVPQSRNICDISCD	20	-0.22	0.62	0.62	-0.36	0.43	0.02	0.95	0	6.23	2282.87	0.97	0.43	0.41
2044	WCKSSQVPQSRNICDISCDK	20	-0.3	0.63	0.63	-0.74	0.61	0.26	1.05	1	8.24	2297.88	0.97	0.40	0.66
2045	CKSSQVPQSRNICDISCDKF	20	-0.29	0.64	0.64	-0.56	0.61	0.31	1	1	8.24	2258.84	0.86	0.41	0.22
2046	KSSQVPQSRNICDISCDKFL	20	-0.27	0.64	0.64	-0.49	0.61	0.27	1	1	8.36	2268.87	0.99	0.47	0.34

2047	SSQVPQSRNICDISCDKFLD	20	-0.25	0.64	0.64	-0.47	0.43	0.27	0.95	-1	4.43	2255.78	0.99	0.34	0.15
2048	SQVPQSRNICDISCDKFLDD	20	-0.27	0.65	0.65	-0.6	0.43	0.41	0.95	-2	4.14	2283.79	0.99	0.32	0.10
2049	QVPQSRNICDISCDKFLDDD	20	-0.29	0.66	0.66	-0.74	0.43	0.54	0.95	-3	3.98	2311.8	0.99	0.32	0.06
2050	VPQSRNICDISCDKFLDDDI	20	-0.22	0.66	0.66	-0.34	0.37	0.44	0.85	-3	3.98	2296.83	0.98	0.32	0.08
2051	PQSRNICDISCDKFLDDDIT	20	-0.26	0.66	0.66	-0.58	0.37	0.49	0.9	-3	3.98	2298.8	0.87	0.32	0.09
2052	QSRNICDISCDKFLDDDITD	20	-0.29	0.68	0.68	-0.68	0.37	0.65	0.95	-4	3.86	2316.77	0.98	0.34	0.09
2053	SRNICDISCDKFLDDDITDD	20	-0.29	0.68	0.68	-0.68	0.31	0.78	0.9	-5	3.77	2303.72	0.99	0.34	0.04
2054	RNICDISCDKFLDDDITDDI	20	-0.24	0.69	0.69	-0.42	0.31	0.68	0.85	-5	3.77	2329.81	0.99	0.34	0.07
2055	NICDISCDKFLDDDITDDIM	20	-0.14	0.69	0.69	-0.1	0.18	0.46	0.65	-6	3.5	2304.82	0.99	0.32	0.06
2056	ICDISCDKFLDDDITDDIMC	20	-0.11	0.69	0.69	0.2	0.18	0.4	0.55	-6	3.5	2293.85	0.99	0.33	0.20
2057	CDISCDKFLDDDITDDIMCA	20	-0.13	0.68	0.68	0.07	0.18	0.47	0.55	-6	3.5	2251.76	0.61	0.33	0.04
2058	DISCDKFLDDDITDDIMCAK	20	-0.19	0.68	0.68	-0.25	0.37	0.67	0.65	-5	3.77	2276.8	0.61	0.33	0.04
2059	ISCDKFLDDDITDDIMCAKK	20	-0.21	0.68	0.68	-0.27	0.55	0.67	0.7	-3	4.12	2289.89	0.99	0.34	0.29
2060	SCDKFLDDDITDDIMCAKKI	20	-0.21	0.68	0.68	-0.27	0.55	0.67	0.7	-3	4.12	2289.89	0.98	0.35	0.29
2061	CDKFLDDDITDDIMCAKKIL	20	-0.17	0.68	0.68	-0.04	0.55	0.56	0.65	-3	4.12	2315.98	0.80	0.38	0.27
2062	DKFLDDDITDDIMCAKKILD	20	-0.21	0.68	0.68	-0.34	0.55	0.76	0.7	-4	4	2327.93	0.86	0.38	0.03
2063	KFLDDDITDDIMCAKKILDI	20	-0.13	0.68	0.68	0.06	0.55	0.52	0.65	-3	4.12	2326.01	0.98	0.47	0.04
2064	FLDDDITDDIMCAKKILDIK	20	-0.13	0.68	0.68	0.06	0.55	0.53	0.65	-3	4.12	2326.01	0.61	0.45	0.08
2065	LDDDITDDIMCAKKILDIKG	20	-0.16	0.68	0.68	-0.1	0.55	0.65	0.65	-3	4.12	2235.89	0.99	0.38	0.05
2066	DDDITDDIMCAKKILDIKGI	20	-0.15	0.69	0.69	-0.06	0.55	0.65	0.65	-3	4.12	2235.89	0.61	0.37	0.06
2067	DDITDDIMCAKKILDIKGID	20	-0.15	0.69	0.69	-0.06	0.55	0.65	0.65	-3	4.12	2235.89	0.61	0.35	0.06
2068	DITDDIMCAKKILDIKGIDY	20	-0.11	0.68	0.68	0.05	0.55	0.39	0.65	-2	4.29	2283.98	0.61	0.36	0.05
2069	ITDDIMCAKKILDIKGIDYW	20	-0.05	0.67	0.67	0.17	0.55	0.07	0.65	-1	4.58	2355.11	0.99	0.46	0.07
2070	TDDIMCAKKILDIKGIDYWL	20	-0.06	0.66	0.66	0.14	0.55	0.07	0.65	-1	4.58	2355.11	0.93	0.46	0.04
2071	DDIMCAKKILDIKGIDYWLA	20	-0.04	0.66	0.66	0.27	0.55	0.06	0.6	-1	4.58	2325.08	0.94	0.50	0.22
2072	DIMCAKKILDIKGIDYWLAH	20	-0.03	0.62	0.62	0.28	0.62	-0.11	0.6	0.5	7.06	2347.14	0.94	0.51	0.84
2073	IMCAKKILDIKGIDYWLAHK	20	-0.05	0.62	0.62	0.26	0.81	-0.11	0.65	2.5	9.1	2360.23	0.93	0.53	0.97
2074	MCAKKILDIKGIDYWLAHKA	20	-0.07	0.61	0.61	0.12	0.81	-0.05	0.65	2.5	9.1	2318.14	0.93	0.56	0.98
2075	CAKKILDIKGIDYWLAHKAL	20	-0.06	0.6	0.6	0.22	0.81	-0.07	0.65	2.5	9.1	2300.11	0.69	0.55	0.98
2076	AKKILDIKGIDYWLAHKALC	20	-0.06	0.6	0.6	0.22	0.81	-0.07	0.65	2.5	9.1	2300.11	0.94	0.49	0.98
2077	KKILDIKGIDYWLAHKALCT	20	-0.08	0.6	0.6	0.09	0.81	-0.07	0.7	2.5	9.1	2330.14	0.98	0.46	0.82
2078	KILDIKGIDYWLAHKALCTE	20	-0.05	0.6	0.6	0.11	0.69	-0.07	0.65	0.5	7.06	2331.08	0.98	0.43	0.23
2079	ILDIKGIDYWLAHKALCTEK	20	-0.05	0.6	0.6	0.12	0.69	-0.07	0.65	0.5	7.06	2331.08	0.93	0.45	0.35
2080	LDIKGIDYWLAHKALCTEKL	20	-0.06	0.59	0.59	0.08	0.69	-0.07	0.65	0.5	7.06	2331.08	0.98	0.42	0.36
2081	DIKGIDYWLAHKALCTEKLE	20	-0.12	0.6	0.6	-0.29	0.75	0.17	0.7	-0.5	5.5	2347.03	0.94	0.47	0.02
2082	IKGIDYWLAHKALCTEKLEQ	20	-0.12	0.59	0.59	-0.29	0.81	0.03	0.75	0.5	7.06	2360.08	0.98	0.41	0.07
2083	KGIDYWLAHKALCTEKLEQW	20	-0.14	0.58	0.58	-0.55	0.81	-0.05	0.8	0.5	7.06	2433.13	0.98	0.43	0.20
2084	GIDYWLAHKALCTEKLEQWL	20	-0.06	0.58	0.58	-0.17	0.63	-0.29	0.7	-0.5	5.46	2418.12	0.83	0.41	0.36
2085	IDYWLAHKALCTEKLEQWLC	20	-0.06	0.57	0.57	-0.03	0.63	-0.34	0.7	-0.5	5.46	2464.2	0.93	0.41	0.51
2086	DYWLAHKALCTEKLEQWLCE	20	-0.13	0.57	0.57	-0.43	0.69	-0.1	0.75	-1.5	4.84	2480.15	0.94	0.39	0.38
2087	YWLAHKALCTEKLEQWLCEK	20	-0.15	0.57	0.57	-0.45	0.88	-0.1	0.8	0.5	7.04	2493.24	0.99	0.40	0.56
2088	WLAHKALCTEKLEQWLCEKL	20	-0.12	0.56	0.56	-0.19	0.88	-0.08	0.75	0.5	7.04	2443.23	0.99	0.36	0.87
2089	MRFFVPLFLVGILFPAILAKQ	21	0.21	0.62	0.62	1.61	0.35	-0.95	0.38	2	11.01	2421.4	0.93	0.66	0.97
2090	RFFVPLFLVGILFPAILAKQF	21	0.22	0.61	0.61	1.65	0.35	-1	0.38	2	11.01	2437.38	0.98	0.67	0.99
2091	FFVPLFLVGILFPAILAKQFT	21	0.3	0.61	0.61	1.83	0.23	-1.17	0.24	1	9.11	2382.3	0.94	0.61	0.94
2092	FVPLFLVGILFPAILAKQFTK	21	0.22	0.61	0.61	1.51	0.41	-0.9	0.33	2	10.02	2363.3	0.80	0.61	0.98
2093	VPLFLVGILFPAILAKQFTKC	21	0.19	0.6	0.6	1.5	0.41	-0.83	0.33	2	9.36	2319.26	0.93	0.57	0.98
2094	PLFLVGILFPAILAKQFTKCE	21	0.13	0.6	0.6	1.13	0.47	-0.62	0.38	1	8.54	2349.24	0.93	0.57	0.95
2095	LFLVGILFPAILAKQFTKCEL	21	0.16	0.61	0.61	1.39	0.47	-0.7	0.38	1	8.54	2365.29	0.93	0.51	0.72
2096	FLVGILFPAILAKQFTKCELS	21	0.13	0.61	0.61	1.17	0.47	-0.6	0.43	1	8.54	2339.2	0.94	0.46	0.91
2097	LVGILFPAILAKQFTKCELSQ	21	0.06	0.61	0.61	0.87	0.53	-0.48	0.52	1	8.54	2320.16	0.93	0.46	0.30
2098	VGILFPAILAKQFTKCELSQL	21	0.06	0.61	0.61	0.87	0.53	-0.48	0.52	1	8.54	2320.16	0.93	0.47	0.48
2099	GILFPAILAKQFTKCELSQLL	21	0.06	0.6	0.6	0.85	0.53	-0.49	0.52	1	8.54	2334.19	0.83	0.46	0.89
2100	ILFPAILAKQFTKCELSQLLK	21	0	0.6	0.6	0.69	0.7	-0.35	0.62	2	9.25	2405.31	0.93	0.46	0.92
2101	LFPAILAKQFTKCELSQLLKD	21	-0.07	0.6	0.6	0.3	0.7	-0.12	0.67	1	8.51	2407.23	0.93	0.44	0.73
2102	FPAILAKQFTKCELSQLLKDI	21	-0.06	0.61	0.61	0.34	0.7	-0.12	0.67	1	8.51	2407.23	0.94	0.40	0.65
2103	PAILAKQFTKCELSQLLKDID	21	-0.12	0.61	0.61	0.04	0.7	0.14	0.71	0	6.43	2375.14	0.93	0.42	0.54
2104	AILAKQFTKCELSQLLKDIDG	21	-0.11	0.63	0.63	0.1	0.7	0.14	0.71	0	6.43	2335.08	0.80	0.40	0.15
2105	ILAKQFTKCELSQLLKDIDGY	21	-0.12	0.64	0.64	-0.05	0.7	0.06	0.76	0	6.43	2427.18	0.98	0.36	0.05
2106	LAKQFTKCELSQLLKDIDGYG	21	-0.15	0.64	0.64	-0.29	0.7	0.14	0.76	0	6.43	2371.07	0.99	0.35	0.29
2107	AKQFTKCELSQLLKDIDGYGG	21	-0.16	0.64	0.64	-0.49	0.7	0.23	0.76	0	6.43	2314.96	0.61	0.31	0.05
2108	KQFTKCELSQLLKDIDGYGGI	21	-0.14	0.65	0.65	-0.36	0.7	0.17	0.76	0	6.43	2357.05	0.99	0.33	0.04
2109	QFTKCELSQLLKDIDGYGGIA	21	-0.08	0.65	0.65	-0.09	0.53	0	0.67	-1	4.56	2299.95	0.94	0.32	0.03
2110	FTKCELSQLLKDIDGYGGIAL	21	-0.02	0.64	0.64	0.26	0.47	-0.1	0.57	-1	4.56	2284.98	0.61	0.33	0.22

2111	TKCELSQLLKDIDGYGGIALP	21	-0.05	0.62	0.62	0.05	0.47	0.02	0.57	-1	4.56	2234.92	0.99	0.40	0.06
2112	KCELSQLLKDIDGYGGIALPE	21	-0.07	0.63	0.63	-0.08	0.53	0.19	0.57	-2	4.32	2262.93	0.99	0.38	0.09
2113	CELSQLLKDIDGYGGIALPEL	21	0	0.62	0.62	0.29	0.36	-0.04	0.48	-3	3.92	2247.92	0.61	0.39	0.03
2114	ELSQLLKDIDGYGGIALPELI	21	0.04	0.63	0.63	0.38	0.36	-0.08	0.48	-3	3.92	2257.95	0.61	0.39	0.03
2115	LSQLLKDIDGYGGIALPELIC	21	0.07	0.62	0.62	0.67	0.29	-0.27	0.43	-2	4.03	2231.97	0.99	0.39	0.07
2116	SQLLKDIDGYGGIALPELICT	21	0.04	0.62	0.62	0.45	0.29	-0.2	0.48	-2	4.03	2219.91	0.99	0.35	0.04
2117	QLLKDIDGYGGIALPELICTM	21	0.06	0.63	0.63	0.58	0.29	-0.28	0.43	-2	4.03	2264.03	0.94	0.36	0.04
2118	LLKDIDGYGGIALPELICTMF	21	0.12	0.64	0.64	0.88	0.24	-0.41	0.33	-2	4.03	2283.07	0.97	0.41	0.04
2119	LKDIDGYGGIALPELICTMFH	21	0.08	0.61	0.61	0.55	0.3	-0.35	0.38	-1.5	4.54	2307.05	0.97	0.45	0.03
2120	KDIDGYGGIALPELICTMFHT	21	0.04	0.61	0.61	0.33	0.3	-0.28	0.43	-1.5	4.54	2294.99	0.97	0.47	0.03
2121	DIDGYGGIALPELICTMFHTS	21	0.08	0.6	0.6	0.48	0.13	-0.41	0.38	-2.5	4.03	2253.89	0.86	0.43	0.06
2122	IDGYGGIALPELICTMFHTSG	21	0.13	0.6	0.6	0.63	0.13	-0.55	0.33	-1.5	4.36	2195.86	0.97	0.34	0.05
2123	DGYGGIALPELICTMFHTSGY	21	0.09	0.6	0.6	0.35	0.13	-0.58	0.38	-1.5	4.36	2245.87	0.94	0.33	0.08
2124	GYGGIALPELICTMFHTSGYD	21	0.09	0.6	0.6	0.35	0.13	-0.58	0.38	-1.5	4.36	2245.87	0.83	0.33	0.21
2125	YGGIALPELICTMFHTSGYDT	21	0.08	0.59	0.59	0.34	0.13	-0.6	0.43	-1.5	4.36	2289.92	0.93	0.29	0.11
2126	GGIALPELICTMFHTSGYDTQ	21	0.04	0.59	0.59	0.23	0.19	-0.48	0.48	-1.5	4.36	2254.88	0.83	0.29	0.18
2127	GIALPELICTMFHTSGYDTQA	21	0.05	0.58	0.58	0.34	0.19	-0.5	0.48	-1.5	4.36	2268.9	0.95	0.28	0.19
2128	IALPELICTMFHTSGYDTQAI	21	0.07	0.58	0.58	0.57	0.19	-0.59	0.48	-1.5	4.36	2325.01	0.93	0.26	0.04
2129	ALPELICTMFHTSGYDTQAIV	21	0.06	0.58	0.58	0.56	0.19	-0.57	0.48	-1.5	4.36	2310.98	0.80	0.28	0.03
2130	LPELICTMFHTSGYDTQAIVE	21	0.02	0.59	0.59	0.3	0.25	-0.4	0.52	-2.5	4.14	2369.02	0.93	0.32	0.03
2131	PELICTMFHTSGYDTQAIVEN	21	-0.03	0.6	0.6	-0.04	0.25	-0.31	0.62	-2.5	4.14	2369.96	0.87	0.31	0.04
2132	ELICTMFHTSGYDTQAIVENN	21	-0.06	0.62	0.62	-0.13	0.25	-0.3	0.71	-2.5	4.14	2386.95	0.94	0.33	0.03
2133	LICTMFHTSGYDTQAIVENNE	21	-0.06	0.62	0.62	-0.13	0.25	-0.3	0.71	-2.5	4.14	2386.95	0.93	0.34	0.07
2134	ICTMFHTSGYDTQAIVENNES	21	-0.1	0.62	0.62	-0.35	0.25	-0.2	0.76	-2.5	4.14	2360.86	0.93	0.33	0.07
2135	CTMFHTSGYDTQAIVENNEST	21	-0.14	0.61	0.61	-0.6	0.25	-0.13	0.81	-2.5	4.14	2348.8	0.94	0.34	0.08
2136	TMFHTSGYDTQAIVENNESTE	21	-0.17	0.62	0.62	-0.89	0.31	0.06	0.86	-3.5	4	2374.78	0.93	0.35	0.03
2137	MFHTSGYDTQAIVENNESTEY	21	-0.16	0.63	0.63	-0.91	0.31	-0.03	0.86	-3.5	4	2436.85	0.93	0.34	0.04
2138	FHTSGYDTQAIVENNESTEYG	21	-0.17	0.62	0.62	-1.02	0.31	0.03	0.86	-3.5	4	2362.71	0.94	0.36	0.03
2139	HTSGYDTQAIVENNESTEYGL	21	-0.17	0.61	0.61	-0.98	0.31	0.06	0.86	-3.5	4	2328.7	0.93	0.30	0.03
2140	TSGYDTQAIVENNESTEYGLF	21	-0.12	0.65	0.65	-0.69	0.24	-0.03	0.81	-4	3.51	2338.73	0.93	0.29	0.04
2141	SGYDTQAIVENNESTEYGLFQ	21	-0.15	0.65	0.65	-0.82	0.3	0	0.86	-4	3.51	2365.76	0.93	0.30	0.04
2142	GYDTQAIVENNESTEYGLFQI	21	-0.1	0.66	0.66	-0.57	0.3	-0.1	0.81	-4	3.51	2391.85	0.83	0.34	0.04
2143	YDTQAIVENNESTEYGLFQIS	21	-0.12	0.65	0.65	-0.59	0.3	-0.09	0.86	-4	3.51	2421.87	0.98	0.39	0.08
2144	DTQAIVENNESTEYGLFQISN	21	-0.15	0.66	0.66	-0.7	0.3	0.03	0.9	-4	3.51	2372.8	0.94	0.37	0.05
2145	TQAIVENNESTEYGLFQISNK	21	-0.17	0.65	0.65	-0.71	0.48	0.03	0.95	-2	4.26	2385.89	0.98	0.35	0.06
2146	QAIVENNESTEYGLFQISNKL	21	-0.14	0.65	0.65	-0.5	0.48	-0.04	0.9	-2	4.26	2397.95	0.98	0.38	0.07
2147	AIVENNESTEYGLFQISNKLW	21	-0.09	0.64	0.64	-0.38	0.42	-0.21	0.86	-2	4.26	2456.03	0.80	0.42	0.06
2148	IVENNESTEYGLFQISNKLWC	21	-0.1	0.65	0.65	-0.34	0.42	-0.23	0.86	-2	4.26	2488.09	0.87	0.41	0.08
2149	VENNESTEYGLFQISNKLWCK	21	-0.18	0.65	0.65	-0.74	0.59	0	0.95	-1	4.79	2503.1	0.98	0.38	0.08
2150	ENNESTEYGLFQISNKLWCKS	21	-0.22	0.64	0.64	-0.98	0.59	0.08	1	-1	4.79	2491.04	0.86	0.39	0.05
2151	NNESTEYGLFQISNKLWCKSS	21	-0.2	0.63	0.63	-0.85	0.53	-0.05	1	0	6.45	2449	0.94	0.42	0.04
2152	NESTEYGLFQISNKLWCKSSQ	21	-0.21	0.63	0.63	-0.85	0.59	-0.05	1	0	6.45	2463.03	0.93	0.44	0.03
2153	ESTEYGLFQISNKLWCKSSQV	21	-0.15	0.63	0.63	-0.49	0.59	-0.13	0.9	0	6.45	2448.06	0.61	0.50	0.04
2154	STEYGLFQISNKLWCKSSQVP	21	-0.12	0.61	0.61	-0.4	0.53	-0.27	0.86	1	8.51	2416.06	0.99	0.50	0.05
2155	TEYGLFQISNKLWCKSSQVPQ	21	-0.14	0.62	0.62	-0.52	0.59	-0.28	0.9	1	8.51	2457.12	0.99	0.49	0.04
2156	EYGLFQISNKLWCKSSQVPQS	21	-0.15	0.62	0.62	-0.53	0.59	-0.24	0.9	1	8.51	2443.09	0.61	0.48	0.07
2157	YGLFQISNKLWCKSSQVPQSR	21	-0.2	0.62	0.62	-0.58	0.64	-0.24	1.05	3	9.8	2470.16	0.99	0.47	0.08
2158	GLFQISNKLWCKSSQVPQSRN	21	-0.23	0.62	0.62	-0.68	0.64	-0.12	1.1	3	10.07	2421.09	0.93	0.45	0.56
2159	LFQISNKLWCKSSQVPQSRNI	21	-0.21	0.62	0.62	-0.45	0.64	-0.21	1.1	3	10.07	2477.2	0.94	0.41	0.10
2160	FQISNKLWCKSSQVPQSRNIC	21	-0.23	0.63	0.63	-0.51	0.64	-0.17	1.1	3	9.53	2467.17	0.61	0.43	0.53
2161	QISNKLWCKSSQVPQSRNICD	21	-0.29	0.63	0.63	-0.81	0.64	0.09	1.14	2	8.98	2435.08	0.99	0.47	0.12
2162	ISNKLWCKSSQVPQSRNICDI	21	-0.23	0.63	0.63	-0.43	0.59	0	1.05	2	8.98	2420.11	0.97	0.47	0.12
2163	SNKLWCKSSQVPQSRNICDIS	21	-0.27	0.62	0.62	-0.68	0.59	0.1	1.1	2	8.98	2394.02	0.99	0.47	0.11
2164	NKLWCKSSQVPQSRNICDISC	21	-0.26	0.63	0.63	-0.52	0.59	0.03	1.05	2	8.77	2410.08	0.99	0.45	0.76
2165	KLWCKSSQVPQSRNICDISCD	21	-0.26	0.63	0.63	-0.52	0.59	0.17	1	1	8.24	2411.06	0.99	0.42	0.69
2166	LWCKSSQVPQSRNICDISCDK	21	-0.26	0.63	0.63	-0.52	0.59	0.17	1	1	8.24	2411.06	0.97	0.38	0.61
2167	WCKSSQVPQSRNICDISCDKF	21	-0.26	0.63	0.63	-0.57	0.59	0.13	1	1	8.24	2445.07	0.99	0.39	0.71
2168	CKSSQVPQSRNICDISCDKFL	21	-0.25	0.64	0.64	-0.35	0.59	0.21	0.95	1	8.24	2372.02	0.86	0.40	0.52
2169	KSSQVPQSRNICDISCDKFLD	21	-0.29	0.64	0.64	-0.63	0.59	0.4	1	0	6.33	2383.97	0.99	0.32	0.14
2170	SSQVPQSRNICDISCDKFLDD	21	-0.27	0.65	0.65	-0.61	0.41	0.4	0.95	-2	4.14	2370.88	0.99	0.29	0.10
2171	SQVPQSRNICDISCDKFLDDD	21	-0.29	0.66	0.66	-0.74	0.41	0.53	0.95	-3	3.98	2398.89	0.99	0.30	0.06
2172	QVPQSRNICDISCDKFLDDDI	21	-0.24	0.67	0.67	-0.49	0.41	0.43	0.9	-3	3.98	2424.98	0.99	0.31	0.07
2173	VPQSRNICDISCDKFLDDDIT	21	-0.22	0.66	0.66	-0.36	0.35	0.4	0.86	-3	3.98	2397.95	0.98	0.30	0.06
2174	PQSRNICDISCDKFLDDDITD	21	-0.28	0.66	0.66	-0.72	0.35	0.61	0.9	-4	3.86	2413.9	0.87	0.27	0.10

2175	QSRNICDISCDKFLDDDITDD	21	-0.31	0.68	0.68	-0.81	0.35	0.76	0.95	-5	3.77	2431.87	0.99	0.32	0.08
2176	SRNICDISCDKFLDDDITDDI	21	-0.24	0.68	0.68	-0.43	0.29	0.66	0.86	-5	3.77	2416.9	0.99	0.32	0.07
2177	RNICDISCDKFLDDDITDDIM	21	-0.22	0.69	0.69	-0.3	0.29	0.59	0.81	-5	3.77	2461.02	0.99	0.30	0.08
2178	NICDISCDKFLDDDITDDIMC	21	-0.13	0.69	0.69	0.03	0.17	0.4	0.62	-6	3.5	2407.97	0.99	0.31	0.16
2179	ICDISCDKFLDDDITDDIMCA	21	-0.09	0.68	0.68	0.28	0.17	0.36	0.52	-6	3.5	2364.94	0.98	0.31	0.08
2180	CDISCDKFLDDDITDDIMCAK	21	-0.18	0.68	0.68	-0.12	0.35	0.59	0.62	-5	3.77	2379.95	0.61	0.30	0.04
2181	DISCDKFLDDDITDDIMCAKK	21	-0.23	0.68	0.68	-0.42	0.52	0.78	0.71	-4	4	2404.99	0.61	0.32	0.14
2182	ISCDKFLDDDITDDIMCAKKI	21	-0.16	0.68	0.68	-0.04	0.52	0.55	0.67	-3	4.12	2403.07	0.99	0.33	0.30
2183	SCDKFLDDDITDDIMCAKKIL	21	-0.17	0.67	0.67	-0.08	0.52	0.55	0.67	-3	4.12	2403.07	0.98	0.37	0.42
2184	CDKFLDDDITDDIMCAKKILD	21	-0.19	0.68	0.68	-0.2	0.52	0.68	0.67	-4	4	2431.08	0.80	0.37	0.04
2185	DKFLDDDITDDIMCAKKILDI	21	-0.16	0.68	0.68	-0.11	0.52	0.64	0.67	-4	4	2441.11	0.80	0.37	0.03
2186	KFLDDDITDDIMCAKKILDIK	21	-0.18	0.68	0.68	-0.13	0.7	0.64	0.71	-2	4.4	2454.2	0.99	0.44	0.06
2187	FLDDDITDDIMCAKKILDIKG	21	-0.12	0.68	0.68	0.04	0.52	0.5	0.62	-3	4.12	2383.08	0.61	0.43	0.11
2188	LDDDITDDIMCAKKILDIKGI	21	-0.11	0.68	0.68	0.12	0.52	0.53	0.62	-3	4.12	2349.07	0.99	0.37	0.05
2189	DDDITDDIMCAKKILDIKGID	21	-0.17	0.69	0.69	-0.23	0.52	0.76	0.67	-4	4	2350.99	0.61	0.35	0.05
2190	DDITDDIMCAKKILDIKGIDY	21	-0.14	0.69	0.69	-0.12	0.52	0.51	0.67	-3	4.12	2399.08	0.61	0.36	0.04
2191	DITDDIMCAKKILDIKGIDYW	21	-0.09	0.68	0.68	0	0.52	0.2	0.67	-2	4.29	2470.21	0.80	0.46	0.05
2192	ITDDIMCAKKILDIKGIDYWL	21	-0.03	0.67	0.67	0.35	0.52	-0.02	0.62	-1	4.58	2468.29	0.93	0.47	0.06
2193	TDDIMCAKKILDIKGIDYWLA	21	-0.05	0.66	0.66	0.22	0.52	0.04	0.62	-1	4.58	2426.2	0.98	0.51	0.07
2194	DDIMCAKKILDIKGIDYWLAH	21	-0.06	0.63	0.63	0.1	0.59	0.03	0.62	-0.5	5.37	2462.24	0.94	0.54	0.12
2195	DIMCAKKILDIKGIDYWLAHK	21	-0.08	0.63	0.63	0.08	0.77	0.03	0.67	1.5	8.46	2475.33	0.94	0.54	0.93
2196	IMCAKKILDIKGIDYWLAHKA	21	-0.03	0.62	0.62	0.33	0.77	-0.13	0.62	2.5	9.1	2431.32	0.93	0.59	0.98
2197	MCAKKILDIKGIDYWLAHKAL	21	-0.04	0.61	0.61	0.3	0.77	-0.13	0.62	2.5	9.1	2431.32	0.93	0.58	0.98
2198	CAKKILDIKGIDYWLAHKALC	21	-0.05	0.6	0.6	0.33	0.77	-0.12	0.62	2.5	8.87	2403.26	0.69	0.51	0.98
2199	AKKILDIKGIDYWLAHKALCT	21	-0.06	0.6	0.6	0.18	0.77	-0.09	0.67	2.5	9.1	2401.23	0.94	0.54	0.91
2200	KKILDIKGIDYWLAHKALCTE	21	-0.1	0.6	0.6	-0.08	0.83	0.08	0.71	1.5	8.46	2459.27	0.98	0.49	0.56
2201	KILDIKGIDYWLAHKALCTEK	21	-0.1	0.6	0.6	-0.08	0.83	0.08	0.71	1.5	8.46	2459.27	0.98	0.49	0.48
2202	ILDIKGIDYWLAHKALCTEKL	21	-0.03	0.6	0.6	0.29	0.65	-0.15	0.62	0.5	7.06	2444.26	0.93	0.50	0.32
2203	LDIKGIDYWLAHKALCTEKLE	21	-0.09	0.6	0.6	-0.09	0.71	0.08	0.67	-0.5	5.5	2460.21	0.98	0.47	0.03
2204	DIKGIDYWLAHKALCTEKLEQ	21	-0.15	0.6	0.6	-0.44	0.77	0.17	0.76	-0.5	5.5	2475.18	0.94	0.46	0.03
2205	IKGIDYWLAHKALCTEKLEQW	21	-0.1	0.59	0.59	-0.31	0.77	-0.13	0.76	0.5	7.06	2546.31	0.93	0.46	0.17
2206	KGIDYWLAHKALCTEKLEQWL	21	-0.11	0.58	0.58	-0.35	0.77	-0.13	0.76	0.5	7.06	2546.31	0.98	0.47	0.41
2207	GIDYWLAHKALCTEKLEQWLC	21	-0.05	0.58	0.58	-0.04	0.6	-0.32	0.67	-0.5	5.46	2521.27	0.83	0.43	0.45
2208	IDYWLAHKALCTEKLEQWLCE	21	-0.09	0.58	0.58	-0.19	0.66	-0.18	0.71	-1.5	4.84	2593.33	0.93	0.41	0.14
2209	DYWLAHKALCTEKLEQWLCEK	21	-0.18	0.58	0.58	-0.59	0.83	0.05	0.81	-0.5	5.54	2608.34	0.86	0.41	0.24
2210	YWLAHKALCTEKLEQWLCEKL	21	-0.12	0.57	0.57	-0.24	0.83	-0.18	0.76	0.5	7.04	2606.42	0.99	0.44	0.88
2211	MRFFVPLFLVGILFPAILAKQF	22	0.22	0.62	0.62	1.66	0.34	-1.02	0.36	2	11.01	2568.59	0.93	0.67	0.98
2212	RFFVPLFLVGILFPAILAKQFT	22	0.2	0.61	0.61	1.55	0.34	-0.98	0.41	2	11.01	2538.5	0.98	0.63	0.89
2213	FFVPLFLVGILFPAILAKQFTK	22	0.23	0.61	0.61	1.57	0.39	-0.98	0.32	2	10.02	2510.49	0.80	0.63	0.99
2214	FVPLFLVGILFPAILAKQFTKC	22	0.21	0.61	0.61	1.56	0.39	-0.91	0.32	2	9.36	2466.45	0.94	0.60	0.98
2215	VPLFLVGILFPAILAKQFTKCE	22	0.15	0.61	0.61	1.27	0.45	-0.66	0.36	1	8.54	2448.39	0.93	0.57	0.88
2216	PLFLVGILFPAILAKQFTKCEL	22	0.15	0.6	0.6	1.25	0.45	-0.67	0.36	1	8.54	2462.42	0.93	0.52	0.82
2217	LFLVGILFPAILAKQFTKCELS	22	0.14	0.61	0.61	1.29	0.45	-0.66	0.41	1	8.54	2452.38	0.93	0.49	0.64
2218	FLVGILFPAILAKQFTKCELSQ	22	0.09	0.61	0.61	0.96	0.51	-0.57	0.5	1	8.54	2467.35	0.94	0.47	0.72
2219	LVGILFPAILAKQFTKCELSQL	22	0.08	0.6	0.6	1	0.51	-0.54	0.5	1	8.54	2433.34	0.93	0.44	0.21
2220	VGILFPAILAKQFTKCELSQLL	22	0.08	0.6	0.6	1	0.51	-0.54	0.5	1	8.54	2433.34	0.93	0.47	0.80
2221	GILFPAILAKQFTKCELSQLLK	22	0.01	0.6	0.6	0.64	0.67	-0.33	0.59	2	9.25	2462.38	0.83	0.47	0.94
2222	ILFPAILAKQFTKCELSQLLKD	22	-0.03	0.61	0.61	0.5	0.67	-0.2	0.64	1	8.51	2520.41	0.93	0.46	0.88
2223	LFPAILAKQFTKCELSQLLKDI	22	-0.03	0.61	0.61	0.5	0.67	-0.2	0.64	1	8.51	2520.41	0.93	0.43	0.57
2224	FPAILAKQFTKCELSQLLKDID	22	-0.09	0.62	0.62	0.16	0.67	0.02	0.68	0	6.43	2522.33	0.94	0.43	0.60
2225	PAILAKQFTKCELSQLLKDIDG	22	-0.11	0.62	0.62	0.02	0.67	0.14	0.68	0	6.43	2432.21	0.93	0.43	0.14
2226	AILAKQFTKCELSQLLKDIDGY	22	-0.1	0.63	0.63	0.03	0.67	0.03	0.73	0	6.43	2498.27	0.80	0.35	0.06
2227	ILAKQFTKCELSQLLKDIDGYG	22	-0.11	0.64	0.64	-0.07	0.67	0.05	0.73	0	6.43	2484.25	0.99	0.37	0.04
2228	LAKQFTKCELSQLLKDIDGYGG	22	-0.13	0.64	0.64	-0.29	0.67	0.14	0.73	0	6.43	2428.14	0.98	0.35	0.28
2229	AKQFTKCELSQLLKDIDGYGGI	22	-0.12	0.65	0.65	-0.26	0.67	0.14	0.73	0	6.43	2428.14	0.61	0.32	0.04
2230	KQFTKCELSQLLKDIDGYGGIA	22	-0.12	0.65	0.65	-0.26	0.67	0.14	0.73	0	6.43	2428.14	0.99	0.32	0.09
2231	QFTKCELSQLLKDIDGYGGIAL	22	-0.05	0.64	0.64	0.09	0.51	-0.08	0.64	-1	4.56	2413.13	0.94	0.32	0.03
2232	FTKCELSQLLKDIDGYGGIALP	22	-0.02	0.63	0.63	0.18	0.45	-0.09	0.55	-1	4.56	2382.11	0.86	0.35	0.15
2233	TKCELSQLLKDIDGYGGIALPE	22	-0.08	0.62	0.62	-0.11	0.51	0.16	0.59	-2	4.32	2364.05	0.99	0.37	0.06
2234	KCELSQLLKDIDGYGGIALPEL	22	-0.05	0.62	0.62	0.1	0.51	0.1	0.55	-2	4.32	2376.11	0.99	0.37	0.03
2235	CELSQLLKDIDGYGGIALPELI	22	0.04	0.63	0.63	0.48	0.34	-0.12	0.45	-3	3.92	2361.1	0.86	0.36	0.03
2236	ELSQLLKDIDGYGGIALPELIC	22	0.04	0.63	0.63	0.48	0.34	-0.12	0.45	-3	3.92	2361.1	0.61	0.37	0.03
2237	LSQLLKDIDGYGGIALPELICT	22	0.06	0.62	0.62	0.6	0.28	-0.28	0.45	-2	4.03	2333.09	0.99	0.36	0.04
2238	SQLLKDIDGYGGIALPELICTM	22	0.05	0.63	0.63	0.52	0.28	-0.25	0.45	-2	4.03	2351.12	0.99	0.37	0.12

2239	QLLKDIDGYGGIALPELICTMF	22	0.09	0.64	0.64	0.68	0.28	-0.38	0.41	-2	4.03	2411.22	0.94	0.36	0.04
2240	LLKDIDGYGGIALPELICTMFH	22	0.1	0.61	0.61	0.7	0.29	-0.41	0.36	-1.5	4.54	2420.23	0.97	0.43	0.04
2241	LKDIDGYGGIALPELICTMFHT	22	0.07	0.61	0.61	0.49	0.29	-0.35	0.41	-1.5	4.54	2408.17	0.97	0.46	0.03
2242	KDIDGYGGIALPELICTMFHTS	22	0.03	0.61	0.61	0.28	0.29	-0.25	0.45	-1.5	4.54	2382.08	0.97	0.38	0.03
2243	DIDGYGGIALPELICTMFHTSG	22	0.09	0.61	0.61	0.44	0.12	-0.39	0.36	-2.5	4.03	2310.96	0.94	0.34	0.06
2244	IDGYGGIALPELICTMFHTSGY	22	0.12	0.6	0.6	0.54	0.12	-0.63	0.36	-1.5	4.36	2359.05	0.97	0.34	0.07
2245	DGYGGIALPELICTMFHTSGYD	22	0.05	0.61	0.61	0.18	0.12	-0.41	0.41	-2.5	4.03	2360.97	0.94	0.33	0.09
2246	GYGGIALPELICTMFHTSGYDT	22	0.08	0.6	0.6	0.3	0.12	-0.57	0.41	-1.5	4.36	2346.99	0.83	0.29	0.11
2247	YGGIALPELICTMFHTSGYDTQ	22	0.04	0.6	0.6	0.16	0.18	-0.56	0.5	-1.5	4.36	2418.07	0.93	0.29	0.07
2248	GGIALPELICTMFHTSGYDTQA	22	0.05	0.59	0.59	0.3	0.18	-0.48	0.45	-1.5	4.36	2325.97	0.83	0.28	0.22
2249	GIALPELICTMFHTSGYDTQAI	22	0.08	0.59	0.59	0.53	0.18	-0.56	0.45	-1.5	4.36	2382.08	0.95	0.27	0.06
2250	IALPELICTMFHTSGYDTQAIV	22	0.09	0.59	0.59	0.74	0.18	-0.63	0.45	-1.5	4.36	2424.16	0.93	0.27	0.03
2251	ALPELICTMFHTSGYDTQAIVE	22	0.03	0.59	0.59	0.37	0.24	-0.41	0.5	-2.5	4.14	2440.11	0.80	0.31	0.03
2252	LPELICTMFHTSGYDTQAIVEN	22	-0.01	0.6	0.6	0.13	0.24	-0.38	0.59	-2.5	4.14	2483.14	0.93	0.34	0.04
2253	PELICTMFHTSGYDTQAIVENN	22	-0.06	0.61	0.61	-0.2	0.24	-0.29	0.68	-2.5	4.14	2484.08	0.63	0.30	0.04
2254	ELICTMFHTSGYDTQAIVENNE	22	-0.09	0.62	0.62	-0.29	0.3	-0.15	0.73	-3.5	4	2516.08	0.94	0.30	0.03
2255	LICTMFHTSGYDTQAIVENNES	22	-0.07	0.62	0.62	-0.16	0.24	-0.27	0.73	-2.5	4.14	2474.04	0.93	0.32	0.03
2256	ICTMFHTSGYDTQAIVENNEST	22	-0.1	0.62	0.62	-0.37	0.24	-0.21	0.77	-2.5	4.14	2461.98	0.93	0.33	0.11
2257	CTMFHTSGYDTQAIVENNESTE	22	-0.16	0.62	0.62	-0.73	0.3	0.01	0.82	-3.5	4	2477.93	0.94	0.32	0.04
2258	TMFHTSGYDTQAIVENNESTEY	22	-0.16	0.62	0.62	-0.9	0.3	-0.05	0.86	-3.5	4	2537.97	0.93	0.34	0.04
2259	MFHTSGYDTQAIVENNESTEYG	22	-0.15	0.63	0.63	-0.89	0.3	-0.03	0.82	-3.5	4	2493.92	0.98	0.35	0.04
2260	FHTSGYDTQAIVENNESTEYGL	22	-0.14	0.62	0.62	-0.8	0.3	-0.05	0.82	-3.5	4	2475.89	0.94	0.34	0.03
2261	HTSGYDTQAIVENNESTEYGLF	22	-0.14	0.62	0.62	-0.8	0.3	-0.05	0.82	-3.5	4	2475.89	0.93	0.31	0.04
2262	TSGYDTQAIVENNESTEYGLFQ	22	-0.15	0.65	0.65	-0.82	0.29	-0.02	0.86	-4	3.51	2466.88	0.93	0.27	0.03
2263	SGYDTQAIVENNESTEYGLFQI	22	-0.11	0.65	0.65	-0.58	0.29	-0.09	0.82	-4	3.51	2478.94	0.93	0.33	0.05
2264	GYDTQAIVENNESTEYGLFQIS	22	-0.11	0.65	0.65	-0.58	0.29	-0.09	0.82	-4	3.51	2478.94	0.83	0.39	0.09
2265	YDTQAIVENNESTEYGLFQISN	22	-0.14	0.66	0.66	-0.72	0.29	-0.08	0.91	-4	3.51	2535.99	0.98	0.38	0.04
2266	DTQAIVENNESTEYGLFQISNK	22	-0.2	0.66	0.66	-0.84	0.45	0.16	0.95	-3	4.01	2500.99	0.94	0.36	0.04
2267	TQAIVENNESTEYGLFQISNKL	22	-0.14	0.65	0.65	-0.51	0.45	-0.05	0.91	-2	4.26	2499.07	0.98	0.39	0.11
2268	QAIVENNESTEYGLFQISNKLW	22	-0.11	0.65	0.65	-0.52	0.45	-0.19	0.91	-2	4.26	2584.18	0.98	0.38	0.06
2269	AIVENNESTEYGLFQISNKLWC	22	-0.08	0.64	0.64	-0.25	0.4	-0.25	0.82	-2	4.26	2559.18	0.80	0.43	0.06
2270	IVENNESTEYGLFQISNKLWCK	22	-0.14	0.65	0.65	-0.5	0.56	-0.09	0.91	-1	4.79	2616.28	0.87	0.41	0.12
2271	VENNESTEYGLFQISNKLWCKS	22	-0.19	0.64	0.64	-0.75	0.56	0.01	0.95	-1	4.79	2590.19	0.98	0.40	0.04
2272	ENNESTEYGLFQISNKLWCKSS	22	-0.22	0.63	0.63	-0.97	0.56	0.09	1	-1	4.79	2578.13	0.61	0.42	0.05
2273	NNESTEYGLFQISNKLWCKSSQ	22	-0.23	0.63	0.63	-0.97	0.56	-0.04	1.05	0	6.45	2577.15	0.87	0.42	0.03
2274	NESTEYGLFQISNKLWCKSSQV	22	-0.17	0.63	0.63	-0.62	0.56	-0.11	0.95	0	6.45	2562.18	0.99	0.44	0.04
2275	ESTEYGLFQISNKLWCKSSQVP	22	-0.15	0.61	0.61	-0.54	0.56	-0.12	0.86	0	6.45	2545.19	0.61	0.48	0.04
2276	STEYGLFQISNKLWCKSSQVPQ	22	-0.15	0.61	0.61	-0.54	0.56	-0.25	0.91	1	8.51	2544.21	0.97	0.47	0.04
2277	TEYGLFQISNKLWCKSSQVPQS	22	-0.15	0.61	0.61	-0.54	0.56	-0.25	0.91	1	8.51	2544.21	0.99	0.47	0.03
2278	EYGLFQISNKLWCKSSQVPQSR	22	-0.22	0.62	0.62	-0.71	0.67	-0.1	1.05	2	9.25	2599.29	0.61	0.46	0.10
2279	YGLFQISNKLWCKSSQVPQSRN	22	-0.22	0.62	0.62	-0.71	0.62	-0.22	1.09	3	9.8	2584.28	0.99	0.45	0.07
2280	GLFQISNKLWCKSSQVPQSRN	22	-0.19	0.62	0.62	-0.45	0.62	-0.2	1.05	3	10.07	2534.27	0.93	0.40	0.55
2281	LFQISNKLWCKSSQVPQSRNIC	22	-0.2	0.62	0.62	-0.31	0.62	-0.25	1.05	3	9.53	2580.35	0.94	0.41	0.58
2282	FQISNKLWCKSSQVPQSRNICD	22	-0.25	0.63	0.63	-0.65	0.62	-0.03	1.09	2	8.98	2582.27	0.61	0.45	0.13
2283	QISNKLWCKSSQVPQSRNICDI	22	-0.25	0.63	0.63	-0.57	0.62	0	1.09	2	8.98	2548.26	0.99	0.47	0.10
2284	ISNKLWCKSSQVPQSRNICDIS	22	-0.23	0.63	0.63	-0.45	0.56	0.01	1.05	2	8.98	2507.2	0.99	0.49	0.10
2285	SNKLWCKSSQVPQSRNICDISC	22	-0.26	0.62	0.62	-0.54	0.56	0.05	1.05	2	8.77	2497.17	0.99	0.44	0.69
2286	NKLWCKSSQVPQSRNICDISCD	22	-0.28	0.63	0.63	-0.66	0.56	0.17	1.05	1	8.24	2525.18	0.99	0.44	0.34
2287	KLWCKSSQVPQSRNICDISCDK	22	-0.3	0.63	0.63	-0.68	0.73	0.3	1.05	2	8.75	2539.25	0.99	0.40	0.88
2288	LWCKSSQVPQSRNICDISCDKF	22	-0.22	0.63	0.63	-0.37	0.56	0.05	0.95	1	8.24	2558.25	0.97	0.40	0.69
2289	WCKSSQVPQSRNICDISCDKFL	22	-0.22	0.63	0.63	-0.37	0.56	0.05	0.95	1	8.24	2558.25	0.97	0.43	0.81
2290	CKSSQVPQSRNICDISCDKFLD	22	-0.27	0.64	0.64	-0.49	0.56	0.34	0.95	0	6.3	2487.12	0.86	0.32	0.17
2291	KSSQVPQSRNICDISCDKFLDD	22	-0.31	0.65	0.65	-0.76	0.56	0.52	1	-1	4.58	2499.07	0.99	0.30	0.12
2292	SSQVPQSRNICDISCDKFLDDD	22	-0.29	0.65	0.65	-0.75	0.39	0.52	0.95	-3	3.98	2485.98	0.99	0.30	0.06
2293	SQVPQSRNICDISCDKFLDDDI	22	-0.24	0.66	0.66	-0.5	0.39	0.42	0.91	-3	3.98	2512.07	0.99	0.32	0.08
2294	QVPQSRNICDISCDKFLDDDIT	22	-0.24	0.66	0.66	-0.5	0.39	0.39	0.91	-3	3.98	2526.1	0.98	0.32	0.06
2295	VPQSRNICDISCDKFLDDDITD	22	-0.24	0.66	0.66	-0.5	0.34	0.52	0.86	-4	3.86	2513.05	0.98	0.28	0.06
2296	PQSRNICDISCDKFLDDDITDD	22	-0.3	0.67	0.67	-0.85	0.34	0.72	0.91	-5	3.77	2529	0.87	0.28	0.10
2297	QSRNICDISCDKFLDDDITDDI	22	-0.26	0.68	0.68	-0.57	0.34	0.64	0.91	-5	3.77	2545.05	0.99	0.33	0.12
2298	SRNICDISCDKFLDDDITDDIM	22	-0.22	0.69	0.69	-0.33	0.28	0.57	0.82	-5	3.77	2548.11	0.99	0.30	0.09
2299	RNICDISCDKFLDDDITDDIMC	22	-0.21	0.69	0.69	-0.18	0.28	0.51	0.77	-5	3.77	2564.17	0.99	0.31	0.22
2300	NICDISCDKFLDDDITDDIMCA	22	-0.11	0.68	0.68	0.11	0.17	0.35	0.59	-6	3.5	2479.06	0.98	0.31	0.06
2301	ICDISCDKFLDDDITDDIMCAK	22	-0.14	0.68	0.68	0.09	0.33	0.48	0.59	-5	3.77	2493.13	0.99	0.30	0.14
2302	CDISCDKFLDDDITDDIMCAKK	22	-0.22	0.68	0.68	-0.29	0.5	0.7	0.68	-4	4	2508.14	0.61	0.33	0.23

2303	DISCDKFLDDDITDDIMCAKKI	22	-0.19	0.68	0.68	-0.2	0.5	0.66	0.68	-4	4	2518.17	0.61	0.34	0.07
2304	ISCDKFLDDDITDDIMCAKKIL	22	-0.13	0.67	0.67	0.13	0.5	0.45	0.64	-3	4.12	2516.25	0.98	0.38	0.40
2305	SCDKFLDDDITDDIMCAKKILD	22	-0.2	0.67	0.67	-0.23	0.5	0.66	0.68	-4	4	2518.17	0.98	0.38	0.18
2306	CDKFLDDDITDDIMCAKKILDI	22	-0.15	0.68	0.68	0.01	0.5	0.57	0.64	-4	4	2544.26	0.94	0.38	0.04
2307	DKFLDDDITDDIMCAKKILDIK	22	-0.2	0.68	0.68	-0.28	0.67	0.75	0.73	-3	4.22	2569.3	0.61	0.35	0.05
2308	KFLDDDITDDIMCAKKILDIKG	22	-0.16	0.68	0.68	-0.14	0.67	0.61	0.68	-2	4.4	2511.27	0.98	0.40	0.10
2309	FLDDDITDDIMCAKKILDIKGI	22	-0.08	0.68	0.68	0.24	0.5	0.4	0.59	-3	4.12	2496.26	0.61	0.39	0.04
2310	LDDDITDDIMCAKKILDIKGID	22	-0.14	0.68	0.68	-0.05	0.5	0.65	0.64	-4	4	2464.17	0.99	0.33	0.04
2311	DDDITDDIMCAKKILDIKGIDY	22	-0.16	0.69	0.69	-0.28	0.5	0.62	0.68	-4	4	2514.18	0.80	0.34	0.04
2312	DDITDDIMCAKKILDIKGIDYW	22	-0.11	0.68	0.68	-0.16	0.5	0.33	0.68	-3	4.12	2585.31	0.80	0.44	0.04
2313	DITDDIMCAKKILDIKGIDYWL	22	-0.06	0.67	0.67	0.17	0.5	0.11	0.64	-2	4.29	2583.39	0.94	0.46	0.04
2314	ITDDIMCAKKILDIKGIDYWLA	22	-0.01	0.66	0.66	0.41	0.5	-0.05	0.59	-1	4.58	2539.38	0.93	0.51	0.11
2315	TDDIMCAKKILDIKGIDYWLAH	22	-0.07	0.63	0.63	0.06	0.57	0.01	0.64	-0.5	5.37	2563.36	0.93	0.50	0.04
2316	DDIMCAKKILDIKGIDYWLAHK	22	-0.11	0.63	0.63	-0.08	0.73	0.17	0.68	0.5	7.05	2590.43	0.94	0.52	0.31
2317	DIMCAKKILDIKGIDYWLAHKA	22	-0.06	0.62	0.62	0.16	0.73	0.01	0.64	1.5	8.46	2546.42	0.94	0.56	0.96
2318	IMCAKKILDIKGIDYWLAHKAL	22	-0.01	0.61	0.61	0.49	0.73	-0.21	0.59	2.5	9.1	2544.5	0.97	0.57	0.98
2319	MCAKKILDIKGIDYWLAHKALC	22	-0.04	0.61	0.61	0.4	0.73	-0.17	0.59	2.5	8.87	2534.47	0.93	0.51	0.98
2320	CAKKILDIKGIDYWLAHKALCT	22	-0.06	0.6	0.6	0.28	0.73	-0.13	0.64	2.5	8.87	2504.38	0.69	0.53	0.97
2321	AKKILDIKGIDYWLAHKALCTE	22	-0.09	0.6	0.6	0.01	0.79	0.05	0.68	1.5	8.46	2530.36	0.94	0.55	0.86
2322	KKILDIKGIDYWLAHKALCTEK	22	-0.15	0.61	0.61	-0.25	0.96	0.21	0.77	2.5	9.05	2587.46	0.98	0.50	0.72
2323	KILDIKGIDYWLAHKALCTEKL	22	-0.07	0.6	0.6	0.1	0.79	-0.01	0.68	1.5	8.46	2572.45	0.98	0.48	0.46
2324	ILDIKGIDYWLAHKALCTEKLE	22	-0.05	0.6	0.6	0.12	0.68	-0.01	0.64	-0.5	5.5	2573.39	0.98	0.51	0.03
2325	LDIKGIDYWLAHKALCTEKLEQ	22	-0.12	0.6	0.6	-0.25	0.74	0.08	0.73	-0.5	5.5	2588.36	0.93	0.42	0.03
2326	DIKGIDYWLAHKALCTEKLEQW	22	-0.12	0.6	0.6	-0.46	0.74	0.01	0.77	-0.5	5.5	2661.41	0.94	0.45	0.04
2327	IKGIDYWLAHKALCTEKLEQWL	22	-0.07	0.59	0.59	-0.13	0.74	-0.21	0.73	0.5	7.06	2659.49	0.93	0.44	0.31
2328	KGIDYWLAHKALCTEKLEQWLC	22	-0.1	0.58	0.58	-0.22	0.74	-0.17	0.73	0.5	7.03	2649.46	0.93	0.46	0.57
2329	GIDYWLAHKALCTEKLEQWLCE	22	-0.08	0.58	0.58	-0.2	0.63	-0.17	0.68	-1.5	4.84	2650.4	0.83	0.40	0.10
2330	IDYWLAHKALCTEKLEQWLCEK	22	-0.13	0.58	0.58	-0.36	0.8	-0.04	0.77	-0.5	5.54	2721.52	0.93	0.40	0.13
2331	DYWLAHKALCTEKLEQWLCEKI	22	-0.14	0.58	0.58	-0.39	0.8	-0.04	0.77	-0.5	5.54	2721.52	0.86	0.44	0.57
2332	MRFFVPLFLVGILFPAILAKQFT	23	0.21	0.62	0.62	1.56	0.32	-0.99	0.39	2	11.01	2669.71	0.93	0.60	0.77
2333	RFFVPLFLVGILFPAILAKQFTK	23	0.15	0.61	0.61	1.31	0.48	-0.8	0.48	3	11.17	2666.69	0.98	0.62	0.96
2334	FFVPLFLVGILFPAILAKQFTKC	23	0.23	0.61	0.61	1.61	0.37	-0.98	0.3	2	9.36	2613.64	0.94	0.59	0.98
2335	FVPLFLVGILFPAILAKQFTKCE	23	0.17	0.61	0.61	1.34	0.43	-0.74	0.35	1	8.54	2595.58	0.94	0.57	0.98
2336	VPLFLVGILFPAILAKQFTKCEL	23	0.17	0.6	0.6	1.38	0.43	-0.71	0.35	1	8.54	2561.57	0.93	0.49	0.65
2337	PLFLVGILFPAILAKQFTKCELS	23	0.13	0.6	0.6	1.17	0.43	-0.63	0.39	1	8.54	2549.51	0.98	0.47	0.88
2338	LFLVGILFPAILAKQFTKCELSQ	23	0.11	0.61	0.61	1.08	0.48	-0.62	0.48	1	8.54	2580.53	0.93	0.47	0.44
2339	FLVGILFPAILAKQFTKCELSQL	23	0.11	0.61	0.61	1.08	0.48	-0.62	0.48	1	8.54	2580.53	0.94	0.45	0.48
2340	LVGILFPAILAKQFTKCELSQLL	23	0.1	0.6	0.6	1.13	0.48	-0.59	0.48	1	8.54	2546.52	0.93	0.43	0.67
2341	VGILFPAILAKQFTKCELSQLLK	23	0.03	0.61	0.61	0.79	0.64	-0.38	0.57	2	9.25	2561.53	0.93	0.47	0.92
2342	GILFPAILAKQFTKCELSQLLKD	23	-0.02	0.61	0.61	0.46	0.64	-0.19	0.61	1	8.51	2577.48	0.83	0.50	0.97
2343	ILFPAILAKQFTKCELSQLLKDI	23	0	0.61	0.61	0.67	0.64	-0.27	0.61	1	8.51	2633.59	0.93	0.49	0.73
2344	LFPAILAKQFTKCELSQLLKDID	23	-0.06	0.61	0.61	0.32	0.64	-0.06	0.65	0	6.43	2635.51	0.93	0.45	0.57
2345	FPAILAKQFTKCELSQLLKDIDG	23	-0.08	0.62	0.62	0.14	0.64	0.02	0.65	0	6.43	2579.4	0.94	0.44	0.19
2346	PAILAKQFTKCELSQLLKDIDGY	23	-0.1	0.62	0.62	-0.04	0.64	0.03	0.7	0	6.43	2595.4	0.93	0.39	0.07
2347	AILAKQFTKCELSQLLKDIDGYG	23	-0.09	0.63	0.63	0.01	0.64	0.03	0.7	0	6.43	2555.34	0.80	0.38	0.09
2348	LAKQFTKCELSQLLKDIDGYGG	23	-0.1	0.64	0.64	-0.08	0.64	0.05	0.7	0	6.43	2541.32	0.98	0.36	0.04
2349	LAKQFTKCELSQLLKDIDGYGG	23	-0.1	0.64	0.64	-0.08	0.64	0.05	0.7	0	6.43	2541.32	0.98	0.35	0.13
2350	AKQFTKCELSQLLKDIDGYGGIA	23	-0.11	0.64	0.64	-0.17	0.64	0.11	0.7	0	6.43	2499.23	0.61	0.33	0.03
2351	KQFTKCELSQLLKDIDGYGGIAL	23	-0.1	0.64	0.64	-0.08	0.64	0.05	0.7	0	6.43	2541.32	0.99	0.35	0.08
2352	QFTKCELSQLLKDIDGYGGIALP	23	-0.05	0.63	0.63	0.02	0.48	-0.08	0.61	-1	4.56	2510.26	0.87	0.34	0.03
2353	FTKCELSQLLKDIDGYGGIALPE	23	-0.05	0.63	0.63	0.02	0.48	0.04	0.57	-2	4.32	2511.24	0.86	0.33	0.13
2354	TKCELSQLLKDIDGYGGIALPEL	23	-0.05	0.62	0.62	0.06	0.48	0.07	0.57	-2	4.32	2477.23	0.99	0.36	0.03
2355	KCELSQLLKDIDGYGGIALPELI	23	-0.01	0.63	0.63	0.29	0.48	0.01	0.52	-2	4.32	2489.29	0.99	0.35	0.05
2356	CELSQLLKDIDGYGGIALPELIC	23	0.04	0.63	0.63	0.57	0.32	-0.16	0.43	-3	3.92	2464.25	0.86	0.35	0.03
2357	ELSQLLKDIDGYGGIALPELICT	23	0.03	0.62	0.62	0.43	0.32	-0.13	0.48	-3	3.92	2462.22	0.61	0.35	0.04
2358	LSQLLKDIDGYGGIALPELICTM	23	0.07	0.63	0.63	0.66	0.27	-0.32	0.43	-2	4.03	2464.3	0.97	0.35	0.07
2359	SQLLKDIDGYGGIALPELICTMF	23	0.07	0.63	0.63	0.62	0.27	-0.35	0.43	-2	4.03	2498.31	0.99	0.34	0.04
2360	QLLKDIDGYGGIALPELICTMFH	23	0.06	0.61	0.61	0.51	0.33	-0.39	0.43	-1.5	4.54	2548.38	0.94	0.39	0.04
2361	LLKDIDGYGGIALPELICTMFHT	23	0.09	0.6	0.6	0.63	0.28	-0.41	0.39	-1.5	4.54	2521.35	0.99	0.42	0.03
2362	LKDIDGYGGIALPELICTMFHTS	23	0.05	0.6	0.6	0.43	0.28	-0.32	0.43	-1.5	4.54	2495.26	0.97	0.37	0.03
2363	KDIDGYGGIALPELICTMFHTSG	23	0.04	0.61	0.61	0.25	0.28	-0.24	0.43	-1.5	4.54	2439.15	0.98	0.32	0.03
2364	DIDGYGGIALPELICTMFHTSGY	23	0.08	0.61	0.61	0.37	0.12	-0.47	0.39	-2.5	4.03	2474.15	0.94	0.33	0.07
2365	DGYGGIALPELICTMFHTSGYD	23	0.08	0.61	0.61	0.37	0.12	-0.47	0.39	-2.5	4.03	2474.15	0.93	0.32	0.08
2366	DGYGGIALPELICTMFHTSGYD	23	0.04	0.6	0.6	0.14	0.12	-0.41	0.43	-2.5	4.03	2462.09	0.94	0.29	0.10

2367	YGGIALPELICTMFHTSGYDT	23	0.05	0.6	0.6	0.14	0.17	-0.53	0.48	-1.5	4.36	2475.14	0.83	0.29	0.18
2368	GGIALPELICTMFHTSGYDTQ	23	0.05	0.59	0.59	0.23	0.17	-0.56	0.48	-1.5	4.36	2489.16	0.93	0.28	0.05
2369	GIALPELICTMFHTSGYDTQA	23	0.08	0.59	0.59	0.49	0.17	-0.53	0.43	-1.5	4.36	2439.15	0.83	0.28	0.07
2370	GIALPELICTMFHTSGYDTQAI	23	0.1	0.59	0.59	0.69	0.17	-0.6	0.43	-1.5	4.36	2481.23	0.95	0.30	0.09
2371	IALPELICTMFHTSGYDTQAIVE	23	0.06	0.59	0.59	0.55	0.23	-0.47	0.48	-2.5	4.14	2553.29	0.93	0.31	0.04
2372	LPELICTMFHTSGYDTQAIVE	23	0	0.6	0.6	0.2	0.23	-0.38	0.57	-2.5	4.14	2554.23	0.80	0.34	0.04
2373	PELICTMFHTSGYDTQAIVEN	23	-0.03	0.61	0.61	-0.03	0.23	-0.35	0.65	-2.5	4.14	2597.26	0.93	0.34	0.03
2374	ELICTMFHTSGYDTQAIVENN	23	-0.08	0.61	0.61	-0.34	0.28	-0.14	0.7	-3.5	4	2613.21	0.63	0.28	0.04
2375	LICTMFHTSGYDTQAIVENNE	23	-0.09	0.62	0.62	-0.31	0.28	-0.13	0.74	-3.5	4	2603.17	0.80	0.29	0.04
2376	ICTMFHTSGYDTQAIVENNES	23	-0.07	0.61	0.61	-0.19	0.23	-0.28	0.74	-2.5	4.14	2575.16	0.98	0.34	0.03
2377	CTMFHTSGYDTQAIVENNEST	23	-0.12	0.62	0.62	-0.5	0.28	-0.07	0.78	-3.5	4	2591.11	0.93	0.34	0.10
2378	TMFHTSGYDTQAIVENNESTE	23	-0.15	0.62	0.62	-0.76	0.28	-0.09	0.83	-3.5	4	2641.12	0.94	0.33	0.05
2379	MFHTSGYDTQAIVENNESTEY	23	-0.15	0.62	0.62	-0.88	0.28	-0.05	0.83	-3.5	4	2595.04	0.98	0.37	0.04
2380	FHTSGYDTQAIVENNESTEYG	23	-0.12	0.62	0.62	-0.69	0.28	-0.11	0.78	-3.5	4	2607.1	0.93	0.34	0.03
2381	HTSGYDTQAIVENNESTEYGL	23	-0.1	0.62	0.62	-0.65	0.28	-0.16	0.78	-3.5	4	2623.08	0.94	0.35	0.03
2382	TSGYDTQAIVENNESTEYGLF	23	-0.16	0.62	0.62	-0.92	0.34	-0.04	0.87	-3.5	4	2604.04	0.93	0.29	0.03
2383	SGYDTQAIVENNESTEYGLFQ	23	-0.11	0.65	0.65	-0.59	0.27	-0.1	0.83	-4	3.51	2580.06	0.93	0.28	0.04
2384	GYDTQAIVENNESTEYGLFQI	23	-0.11	0.65	0.65	-0.59	0.27	-0.07	0.83	-4	3.51	2566.03	0.93	0.35	0.04
2385	YDTQAIVENNESTEYGLFQIS	23	-0.13	0.66	0.66	-0.71	0.27	-0.07	0.87	-4	3.51	2593.06	0.83	0.37	0.05
2386	DTQAIVENNESTEYGLFQISN	23	-0.19	0.66	0.66	-0.86	0.43	0.06	0.96	-3	4.01	2664.18	0.93	0.36	0.05
2387	TQAIVENNESTEYGLFQISNK	23	-0.16	0.65	0.65	-0.64	0.43	0.08	0.91	-3	4.01	2614.17	0.94	0.40	0.08
2388	QAIVENNESTEYGLFQISNKL	23	-0.12	0.64	0.64	-0.53	0.43	-0.2	0.91	-2	4.26	2685.3	0.98	0.37	0.06
2389	AIVENNESTEYGLFQISNKLW	23	-0.11	0.64	0.64	-0.39	0.43	-0.23	0.87	-2	4.26	2687.33	0.98	0.37	0.09
2390	IVENNESTEYGLFQISNKLWC	23	-0.12	0.64	0.64	-0.4	0.54	-0.1	0.87	-1	4.79	2687.37	0.80	0.43	0.12
2391	VENNESTEYGLFQISNKLWCK	23	-0.15	0.65	0.65	-0.52	0.54	-0.07	0.91	-1	4.79	2703.37	0.87	0.42	0.05
2392	ENNESTEYGLFQISNKLWCKS	23	-0.19	0.64	0.64	-0.75	0.54	0.02	0.96	-1	4.79	2677.28	0.98	0.43	0.04
2393	NNESTEYGLFQISNKLWCKSS	23	-0.24	0.64	0.64	-1.08	0.59	0.1	1.04	-1	4.79	2706.28	0.80	0.42	0.05
2394	NESTEYGLFQISNKLWCKSSQ	23	-0.19	0.64	0.64	-0.75	0.54	-0.1	1	0	6.45	2676.3	0.87	0.42	0.03
2395	ESTEYGLFQISNKLWCKSSQV	23	-0.17	0.62	0.62	-0.67	0.54	-0.11	0.91	0	6.45	2659.31	0.99	0.44	0.04
2396	STEYGLFQISNKLWCKSSQVP	23	-0.17	0.62	0.62	-0.67	0.59	-0.11	0.91	0	6.45	2673.34	0.61	0.45	0.05
2397	TEYGLFQISNKLWCKSSQVPQ	23	-0.15	0.61	0.61	-0.55	0.54	-0.23	0.91	1	8.51	2631.3	0.99	0.46	0.02
2398	EYGLFQISNKLWCKSSQVPQS	23	-0.22	0.62	0.62	-0.71	0.64	-0.11	1.04	2	9.25	2700.41	0.99	0.45	0.04
2399	YGLFQISNKLWCKSSQVPQSR	23	-0.24	0.63	0.63	-0.83	0.64	-0.08	1.09	2	9.25	2713.41	0.61	0.44	0.09
2400	GLFQISNKLWCKSSQVPQSR	23	-0.18	0.63	0.63	-0.48	0.59	-0.29	1.04	3	9.8	2697.46	0.99	0.40	0.09
2401	LFQISNKLWCKSSQVPQSRNI	23	-0.18	0.62	0.62	-0.32	0.59	-0.23	1	3	9.53	2637.42	0.98	0.40	0.69
2402	FQISNKLWCKSSQVPQSRNIC	23	-0.22	0.63	0.63	-0.45	0.59	-0.1	1.04	2	8.98	2695.45	0.94	0.41	0.15
2403	QISNKLWCKSSQVPQSRNICD	23	-0.21	0.64	0.64	-0.42	0.59	-0.1	1.04	2	8.98	2695.45	0.61	0.42	0.09
2404	ISNKLWCKSSQVPQSRNICDI	23	-0.25	0.63	0.63	-0.58	0.59	0.02	1.09	2	8.98	2635.35	0.99	0.47	0.08
2405	SNKLWCKSSQVPQSRNICDIS	23	-0.22	0.63	0.63	-0.32	0.53	-0.03	1	2	8.77	2610.35	0.97	0.44	0.33
2406	NKLWCKSSQVPQSRNICDISC	23	-0.28	0.63	0.63	-0.67	0.53	0.17	1.04	1	8.24	2612.27	0.99	0.41	0.21
2407	KLWCKSSQVPQSRNICDISCD	23	-0.32	0.63	0.63	-0.8	0.69	0.29	1.09	2	8.75	2653.37	0.99	0.40	0.60
2408	LWCKSSQVPQSRNICDISCDK	23	-0.26	0.63	0.63	-0.53	0.69	0.17	1	2	8.75	2686.44	0.99	0.40	0.83
2409	WCKSSQVPQSRNICDISCDKF	23	-0.19	0.63	0.63	-0.19	0.53	-0.03	0.91	1	8.24	2671.43	0.97	0.43	0.78
2410	CKSSQVPQSRNICDISCDKFL	23	-0.24	0.64	0.64	-0.51	0.53	0.17	0.96	0	6.3	2673.35	0.97	0.32	0.52
2411	KSSQVPQSRNICDISCDKFLD	23	-0.29	0.65	0.65	-0.62	0.53	0.45	0.96	-1	4.58	2602.22	0.86	0.29	0.13
2412	SSQVPQSRNICDISCDKFLDD	23	-0.32	0.65	0.65	-0.88	0.53	0.63	1	-2	4.29	2614.17	0.99	0.29	0.08
2413	SQVPQSRNICDISCDKFLDDD	23	-0.25	0.65	0.65	-0.52	0.37	0.42	0.91	-3	3.98	2599.16	0.99	0.30	0.08
2414	QVPQSRNICDISCDKFLDDDI	23	-0.24	0.65	0.65	-0.51	0.37	0.39	0.91	-3	3.98	2613.19	0.99	0.31	0.07
2415	VPQSRNICDISCDKFLDDDIT	23	-0.26	0.66	0.66	-0.63	0.37	0.5	0.91	-4	3.86	2641.2	0.98	0.28	0.06
2416	PQSRNICDISCDKFLDDDITD	23	-0.26	0.67	0.67	-0.63	0.32	0.63	0.87	-5	3.77	2628.15	0.98	0.27	0.06
2417	PQSRNICDISCDKFLDDDITDD	23	-0.25	0.67	0.67	-0.62	0.32	0.61	0.87	-5	3.77	2642.18	0.87	0.27	0.13
2418	SRNICDISCDKFLDDDITDDI	23	-0.24	0.69	0.69	-0.47	0.32	0.56	0.87	-5	3.77	2676.26	0.99	0.31	0.13
2419	SRNICDISCDKFLDDDITDDIM	23	-0.21	0.68	0.68	-0.2	0.27	0.5	0.78	-5	3.77	2651.26	0.99	0.32	0.08
2420	RNICDISCDKFLDDDITDDIMC	23	-0.19	0.68	0.68	-0.09	0.27	0.47	0.74	-5	3.77	2635.26	0.98	0.32	0.09
2421	NICDISCDKFLDDDITDDIMCA	23	-0.16	0.68	0.68	-0.07	0.32	0.47	0.65	-5	3.77	2607.25	0.99	0.30	0.09
2422	ICDISCDKFLDDDITDDIMCAK	23	-0.18	0.68	0.68	-0.08	0.48	0.59	0.65	-4	4	2621.32	0.98	0.34	0.34
2423	CDISCDKFLDDDITDDIMCAKK	23	-0.18	0.68	0.68	-0.08	0.48	0.59	0.65	-4	4	2621.32	0.80	0.35	0.08
2424	DISCDKFLDDDITDDIMCAKKIL	23	-0.16	0.67	0.67	-0.03	0.48	0.56	0.65	-4	4	2631.35	0.61	0.39	0.12
2425	ISCDKFLDDDITDDIMCAKKILD	23	-0.16	0.67	0.67	-0.03	0.48	0.56	0.65	-4	4	2631.35	0.98	0.38	0.14
2426	SCDKFLDDDITDDIMCAKKILD	23	-0.16	0.67	0.67	-0.03	0.48	0.56	0.65	-4	4	2631.35	0.98	0.39	0.06
2427	CDKFLDDDITDDIMCAKKILDIK	23	-0.19	0.68	0.68	-0.16	0.64	0.67	0.7	-3	4.22	2672.45	0.80	0.36	0.10
2428	DKFLDDDITDDIMCAKKILDIKG	23	-0.19	0.68	0.68	-0.29	0.64	0.72	0.7	-3	4.22	2626.37	0.80	0.39	0.12
2429	KFLDDDITDDIMCAKKILDIKGI	23	-0.12	0.68	0.68	0.06	0.64	0.51	0.65	-2	4.4	2624.45	0.98	0.39	0.06
2430	FLDDDITDDIMCAKKILDIKGID	23	-0.11	0.68	0.68	0.08	0.48	0.51	0.61	-4	4	2611.36	0.61	0.39	0.04

2431	LDDDITDDIMCAKKILDIKGIDY	23	-0.13	0.68	0.68	-0.1	0.48	0.52	0.65	-4	4	2627.36	0.98	0.36	0.04
2432	DDDITDDIMCAKKILDIKGIDYW	23	-0.14	0.68	0.68	-0.3	0.48	0.45	0.7	-4	4	2700.41	0.80	0.47	0.03
2433	DDITDDIMCAKKILDIKGIDYWL	23	-0.09	0.67	0.67	0.01	0.48	0.24	0.65	-3	4.12	2698.49	0.94	0.48	0.03
2434	DITDDIMCAKKILDIKGIDYWLA	23	-0.04	0.66	0.66	0.24	0.48	0.09	0.61	-2	4.29	2654.48	0.80	0.53	0.06
2435	ITDDIMCAKKILDIKGIDYWLAH	23	-0.03	0.63	0.63	0.26	0.54	-0.07	0.61	-0.5	5.37	2676.54	0.93	0.53	0.06
2436	TDDIMCAKKILDIKGIDYWLAHK	23	-0.11	0.63	0.63	-0.11	0.7	0.14	0.7	0.5	7.05	2691.55	0.93	0.51	0.10
2437	DDIMCAKKILDIKGIDYWLAHKA	23	-0.09	0.63	0.63	0	0.7	0.14	0.65	0.5	7.05	2661.52	0.94	0.56	0.76
2438	DIMCAKKILDIKGIDYWLAHKAL	23	-0.04	0.62	0.62	0.32	0.7	-0.07	0.61	1.5	8.46	2659.6	0.94	0.56	0.95
2439	IMCAKKILDIKGIDYWLAHKALC	23	0	0.61	0.61	0.58	0.7	-0.24	0.57	2.5	8.87	2647.65	0.93	0.52	0.98
2440	MCAKKILDIKGIDYWLAHKALCT	23	-0.04	0.61	0.61	0.35	0.7	-0.18	0.61	2.5	8.87	2635.59	0.93	0.54	0.98
2441	CAKKILDIKGIDYWLAHKALCTE	23	-0.08	0.6	0.6	0.12	0.76	0	0.65	1.5	8.32	2633.51	0.69	0.53	0.97
2442	AKKILDIKGIDYWLAHKALCTEK	23	-0.13	0.6	0.6	-0.16	0.92	0.18	0.74	2.5	9.05	2658.55	0.94	0.55	0.90
2443	KKILDIKGIDYWLAHKALCTEKL	23	-0.12	0.6	0.6	-0.07	0.92	0.12	0.74	2.5	9.05	2700.64	0.98	0.51	0.70
2444	KILDIKGIDYWLAHKALCTEKLE	23	-0.1	0.6	0.6	-0.06	0.81	0.12	0.7	0.5	7.06	2701.58	0.98	0.52	0.04
2445	ILDIKGIDYWLAHKALCTEKLEQ	23	-0.08	0.6	0.6	-0.04	0.71	0	0.7	-0.5	5.5	2701.54	0.93	0.47	0.04
2446	LDIKGIDYWLAHKALCTEKLEQW	23	-0.1	0.6	0.6	-0.27	0.71	-0.07	0.74	-0.5	5.5	2774.59	0.98	0.42	0.06
2447	DIKGIDYWLAHKALCTEKLEQWL	23	-0.1	0.6	0.6	-0.27	0.71	-0.07	0.74	-0.5	5.5	2774.59	0.94	0.45	0.09
2448	KGIDYWLAHKALCTEKLEQWLC	23	-0.06	0.59	0.59	-0.01	0.71	-0.24	0.7	0.5	7.03	2762.64	0.93	0.45	0.51
2449	GIDYWLAHKALCTEKLEQWLC	23	-0.12	0.59	0.59	-0.36	0.76	-0.03	0.74	-0.5	5.54	2778.59	0.93	0.46	0.09
2450	GIDYWLAHKALCTEKLEQWLCE	23	-0.12	0.59	0.59	-0.36	0.76	-0.03	0.74	-0.5	5.54	2778.59	0.83	0.40	0.09
2451	DYWLAHKALCTEKLEQWLCEK	23	-0.11	0.58	0.58	-0.18	0.76	-0.11	0.74	-0.5	5.54	2834.7	0.93	0.44	0.43
2452	MRFFVPLFLVGILFPAILAKQFTK	24	0.15	0.62	0.62	1.33	0.46	-0.83	0.46	3	11.17	2797.9	0.98	0.60	0.94
2453	RFFVPLFLVGILFPAILAKQFTKC	24	0.14	0.61	0.61	1.36	0.46	-0.81	0.46	3	10.07	2769.84	0.98	0.59	0.98
2454	FFVPLFLVGILFPAILAKQFTKCE	24	0.19	0.61	0.61	1.4	0.41	-0.81	0.33	1	8.54	2742.77	0.94	0.58	0.98
2455	FVPLFLVGILFPAILAKQFTKCEL	24	0.19	0.61	0.61	1.44	0.41	-0.78	0.33	1	8.54	2708.76	0.94	0.52	0.95
2456	VPLFLVGILFPAILAKQFTKCELS	24	0.15	0.6	0.6	1.29	0.41	-0.67	0.38	1	8.54	2648.66	0.93	0.48	0.57
2457	PLFLVGILFPAILAKQFTKCELSQ	24	0.1	0.6	0.6	0.97	0.46	-0.6	0.46	1	8.54	2677.66	0.98	0.46	0.66
2458	LFLVGILFPAILAKQFTKCELSQL	24	0.13	0.61	0.61	1.2	0.46	-0.67	0.46	1	8.54	2693.71	0.93	0.47	0.35
2459	FLVGILFPAILAKQFTKCELSQLL	24	0.13	0.61	0.61	1.2	0.46	-0.67	0.46	1	8.54	2693.71	0.94	0.43	0.88
2460	LVGILFPAILAKQFTKCELSQLLK	24	0.05	0.6	0.6	0.92	0.62	-0.44	0.54	2	9.25	2674.71	0.93	0.41	0.72
2461	VGILFPAILAKQFTKCELSQLLKD	24	0	0.61	0.61	0.61	0.62	-0.24	0.58	1	8.51	2676.63	0.93	0.44	0.93
2462	GILFPAILAKQFTKCELSQLLKD	24	0.01	0.61	0.61	0.63	0.62	-0.25	0.58	1	8.51	2690.66	0.83	0.45	0.91
2463	ILFPAILAKQFTKCELSQLLKDID	24	-0.03	0.62	0.62	0.5	0.62	-0.13	0.62	0	6.43	2748.69	0.93	0.45	0.74
2464	LFPAILAKQFTKCELSQLLKDIDG	24	-0.05	0.62	0.62	0.29	0.62	-0.05	0.62	0	6.43	2692.58	0.93	0.42	0.21
2465	FPAILAKQFTKCELSQLLKDIDGY	24	-0.07	0.62	0.62	0.08	0.62	-0.07	0.67	0	6.43	2742.59	0.94	0.35	0.09
2466	AILAKQFTKCELSQLLKDIDGYG	24	-0.09	0.62	0.62	-0.05	0.62	0.03	0.67	0	6.43	2652.47	0.93	0.36	0.11
2467	ILAKQFTKCELSQLLKDIDGYGG	24	-0.08	0.64	0.64	0	0.62	0.03	0.67	0	6.43	2612.41	0.80	0.36	0.05
2468	LAKQFTKCELSQLLKDIDGYGG	24	-0.06	0.64	0.64	0.11	0.62	-0.02	0.67	0	6.43	2654.5	0.98	0.35	0.04
2469	AKQFTKCELSQLLKDIDGYGGI	24	-0.08	0.64	0.64	0	0.62	0.03	0.67	0	6.43	2612.41	0.98	0.35	0.30
2470	KQFTKCELSQLLKDIDGYGGIA	24	-0.08	0.64	0.64	0	0.62	0.03	0.67	0	6.43	2612.41	0.61	0.37	0.04
2471	QFTKCELSQLLKDIDGYGGIAL	24	-0.09	0.63	0.63	-0.15	0.62	0.05	0.67	0	6.43	2638.45	0.99	0.38	0.10
2472	FTKCELSQLLKDIDGYGGIALP	24	-0.07	0.63	0.63	-0.13	0.52	0.05	0.62	-2	4.32	2639.39	0.94	0.36	0.04
2473	TKCELSQLLKDIDGYGGIALPE	24	-0.02	0.62	0.62	0.17	0.46	-0.03	0.54	-2	4.32	2624.42	0.86	0.36	0.04
2474	TKCELSQLLKDIDGYGGIALPEL	24	-0.02	0.62	0.62	0.25	0.46	0	0.54	-2	4.32	2590.41	0.99	0.38	0.04
2475	KCELSQLLKDIDGYGGIALPELI	24	-0.01	0.63	0.63	0.38	0.46	-0.03	0.5	-2	4.32	2592.44	0.99	0.37	0.13
2476	CELSQLLKDIDGYGGIALPELICT	24	0.03	0.62	0.62	0.51	0.31	-0.17	0.46	-3	3.92	2565.37	0.86	0.35	0.04
2477	ELSQLLKDIDGYGGIALPELICTM	24	0.04	0.63	0.63	0.49	0.31	-0.18	0.46	-3	3.92	2593.43	0.61	0.36	0.05
2478	LSQLLKDIDGYGGIALPELICTMF	24	0.09	0.63	0.63	0.75	0.26	-0.41	0.42	-2	4.03	2611.49	0.97	0.35	0.03
2479	SQLLKDIDGYGGIALPELICTMFH	24	0.05	0.61	0.61	0.46	0.32	-0.36	0.46	-1.5	4.54	2635.47	0.97	0.40	0.04
2480	QLLKDIDGYGGIALPELICTMFH	24	0.05	0.61	0.61	0.46	0.32	-0.39	0.46	-1.5	4.54	2649.5	0.94	0.40	0.04
2481	LLKDIDGYGGIALPELICTMFHTS	24	0.07	0.6	0.6	0.58	0.27	-0.38	0.42	-1.5	4.54	2608.44	0.99	0.37	0.03
2482	KDIDGYGGIALPELICTMFHTSG	24	0.06	0.61	0.61	0.4	0.27	-0.31	0.42	-1.5	4.54	2552.33	0.93	0.34	0.03
2483	DIDGYGGIALPELICTMFHTSG	24	0.04	0.61	0.61	0.19	0.27	-0.33	0.46	-1.5	4.54	2602.34	0.98	0.33	0.04
2484	IDGYGGIALPELICTMFHTSGY	24	0.05	0.62	0.62	0.2	0.11	-0.33	0.42	-3.5	3.84	2589.25	0.94	0.33	0.04
2485	DGYGGIALPELICTMFHTSGYD	24	0.07	0.61	0.61	0.32	0.11	-0.47	0.42	-2.5	4.03	2575.27	0.93	0.29	0.09
2486	GYGGIALPELICTMFHTSGYDT	24	0.01	0.61	0.61	-0.01	0.17	-0.39	0.5	-2.5	4.03	2590.24	0.94	0.29	0.04
2487	YGGIALPELICTMFHTSGYDTQ	24	0.05	0.6	0.6	0.21	0.17	-0.53	0.46	-1.5	4.36	2546.23	0.83	0.28	0.22
2488	GGIALPELICTMFHTSGYDTQA	24	0.08	0.6	0.6	0.41	0.17	-0.61	0.46	-1.5	4.36	2602.34	0.93	0.28	0.07
2489	GIALPELICTMFHTSGYDTQAI	24	0.1	0.6	0.6	0.64	0.17	-0.57	0.42	-1.5	4.36	2538.3	0.83	0.32	0.11
2490	IALPELICTMFHTSGYDTQAIV	24	0.07	0.6	0.6	0.51	0.22	-0.45	0.46	-2.5	4.14	2610.36	0.95	0.32	0.15
2491	ALPELICTMFHTSGYDTQAIVE	24	0.03	0.6	0.6	0.38	0.22	-0.44	0.54	-2.5	4.14	2667.41	0.93	0.33	0.04
2492	LPELICTMFHTSGYDTQAIVEN	24	-0.02	0.6	0.6	0.05	0.22	-0.36	0.62	-2.5	4.14	2668.35	0.80	0.34	0.03
2493	PELICTMFHTSGYDTQAIVENN	24	-0.06	0.61	0.61	-0.17	0.27	-0.21	0.67	-3.5	4	2726.39	0.93	0.33	0.04
2494	ELICTMFHTSGYDTQAIVENNE	24	-0.09	0.61	0.61	-0.36	0.27	-0.12	0.71	-3.5	4	2700.3	0.63	0.27	0.05

2495	LICTMFHTSGYDTQAIVENNES	24	-0.1	0.62	0.62	-0.32	0.27	-0.14	0.75	-3.5	4	2704.29	0.80	0.30	0.03
2496	ICTMFHTSGYDTQAIVENNEST	24	-0.1	0.62	0.62	-0.33	0.27	-0.14	0.75	-3.5	4	2704.29	0.93	0.34	0.03
2497	CTMFHTSGYDTQAIVENNESTE	24	-0.12	0.62	0.62	-0.54	0.27	-0.16	0.79	-3.5	4	2754.3	0.98	0.35	0.06
2498	TMFHTSGYDTQAIVENNESTEY	24	-0.14	0.62	0.62	-0.74	0.27	-0.09	0.79	-3.5	4	2698.19	0.94	0.35	0.04
2499	MFHTSGYDTQAIVENNESTEYG	24	-0.12	0.62	0.62	-0.69	0.27	-0.12	0.79	-3.5	4	2708.22	0.93	0.36	0.03
2500	FHTSGYDTQAIVENNESTEYGL	24	-0.09	0.63	0.63	-0.54	0.27	-0.21	0.75	-3.5	4	2754.29	0.93	0.36	0.04
2501	HTSGYDTQAIVENNESTEYGLF	24	-0.13	0.62	0.62	-0.77	0.32	-0.15	0.83	-3.5	4	2751.23	0.94	0.34	0.04
2502	TSGYDTQAIVENNESTEYGLFQ	24	-0.12	0.62	0.62	-0.7	0.32	-0.12	0.83	-3.5	4	2717.22	0.93	0.30	0.04
2503	SGYDTQAIVENNESTEYGLFQI	24	-0.12	0.64	0.64	-0.6	0.26	-0.08	0.83	-4	3.51	2667.15	0.93	0.30	0.05
2504	GYDTQAIVENNESTEYGLFQIS	24	-0.14	0.65	0.65	-0.71	0.26	-0.06	0.88	-4	3.51	2680.15	0.93	0.35	0.04
2505	YDTQAIVENNESTEYGLFQISN	24	-0.17	0.66	0.66	-0.84	0.42	0.05	0.92	-3	4.01	2721.25	0.83	0.37	0.08
2506	DTQAIVENNESTEYGLFQISNK	24	-0.16	0.65	0.65	-0.67	0.42	-0.02	0.92	-3	4.01	2777.36	0.93	0.41	0.08
2507	TQAIVENNESTEYGLFQISNKL	24	-0.14	0.65	0.65	-0.65	0.42	-0.07	0.92	-3	4.01	2800.4	0.94	0.40	0.06
2508	QAIVENNESTEYGLFQISNKLW	24	-0.11	0.64	0.64	-0.4	0.42	-0.23	0.88	-2	4.26	2788.45	0.93	0.38	0.12
2509	AIVENNESTEYGLFQISNKLWC	24	-0.15	0.65	0.65	-0.53	0.57	-0.09	0.92	-1	4.79	2815.52	0.98	0.40	0.12
2510	IVENNESTEYGLFQISNKLWCK	24	-0.13	0.64	0.64	-0.42	0.52	-0.09	0.88	-1	4.79	2774.46	0.80	0.44	0.05
2511	VENNESTEYGLFQISNKLWCKS	24	-0.15	0.64	0.64	-0.53	0.52	-0.05	0.92	-1	4.79	2790.46	0.87	0.44	0.04
2512	ENNESTEYGLFQISNKLWCKSS	24	-0.21	0.64	0.64	-0.86	0.57	0.03	1	-1	4.79	2805.43	0.98	0.43	0.04
2513	NNESTEYGLFQISNKLWCKSSQ	24	-0.21	0.64	0.64	-0.86	0.57	0.03	1	-1	4.79	2805.43	0.80	0.42	0.06
2514	NESTEYGLFQISNKLWCKSSQV	24	-0.19	0.63	0.63	-0.78	0.52	-0.1	0.96	0	6.45	2773.43	0.94	0.42	0.03
2515	ESTEYGLFQISNKLWCKSSQVP	24	-0.19	0.62	0.62	-0.78	0.57	-0.1	0.96	0	6.45	2787.46	0.99	0.41	0.04
2516	STEYGLFQISNKLWCKSSQVPQ	24	-0.17	0.61	0.61	-0.67	0.57	-0.09	0.92	0	6.45	2760.43	0.61	0.45	0.03
2517	TEYGLFQISNKLWCKSSQVPQS	24	-0.22	0.61	0.61	-0.71	0.62	-0.09	1.04	2	9.25	2787.5	0.97	0.45	0.04
2518	EYGLFQISNKLWCKSSQVPQSR	24	-0.24	0.62	0.62	-0.83	0.62	-0.1	1.08	2	9.25	2814.53	0.97	0.44	0.03
2519	YGLFQISNKLWCKSSQVPQSRN	24	-0.2	0.63	0.63	-0.61	0.62	-0.15	1.04	2	9.25	2826.59	0.61	0.40	0.04
2520	GLFQISNKLWCKSSQVPQSRN	24	-0.17	0.63	0.63	-0.36	0.56	-0.32	1	3	9.42	2800.61	0.99	0.40	0.59
2521	LFQISNKLWCKSSQVPQSRNIC	24	-0.2	0.63	0.63	-0.45	0.56	-0.1	1	2	8.98	2752.52	0.93	0.42	0.65
2522	FQISNKLWCKSSQVPQSRNICD	24	-0.18	0.63	0.63	-0.25	0.56	-0.17	1	2	8.98	2808.63	0.94	0.41	0.12
2523	QISNKLWCKSSQVPQSRNICDI	24	-0.21	0.63	0.63	-0.44	0.56	-0.09	1.04	2	8.98	2782.54	0.61	0.43	0.09
2524	ISNKLWCKSSQVPQSRNICDIS	24	-0.24	0.63	0.63	-0.45	0.56	-0.02	1.04	2	8.77	2738.5	0.99	0.42	0.44
2525	SNKLWCKSSQVPQSRNICDISC	24	-0.24	0.63	0.63	-0.45	0.51	0.09	1	1	8.24	2725.45	0.97	0.41	0.15
2526	NKLWCKSSQVPQSRNICDISCD	24	-0.31	0.63	0.63	-0.8	0.66	0.29	1.08	2	8.75	2740.46	0.97	0.38	0.37
2527	KLWCKSSQVPQSRNICDISCDK	24	-0.28	0.64	0.64	-0.65	0.66	0.18	1.04	2	8.75	2800.56	0.99	0.41	0.68
2528	LWCKSSQVPQSRNICDISCDKF	24	-0.23	0.63	0.63	-0.35	0.66	0.09	0.96	2	8.75	2799.62	0.99	0.42	0.90
2529	WCKSSQVPQSRNICDISCDKFL	24	-0.21	0.63	0.63	-0.33	0.51	0.09	0.92	0	6.3	2786.53	0.97	0.31	0.44
2530	CKSSQVPQSRNICDISCDKFLD	24	-0.26	0.64	0.64	-0.63	0.51	0.29	0.96	-1	4.58	2788.45	0.97	0.28	0.18
2531	KSSQVPQSRNICDISCDKFLDD	24	-0.31	0.65	0.65	-0.74	0.51	0.56	0.96	-2	4.29	2717.32	0.86	0.28	0.08
2532	SSQVPQSRNICDISCDKFLDDD	24	-0.28	0.65	0.65	-0.66	0.51	0.52	0.96	-2	4.29	2727.35	0.99	0.29	0.08
2533	SQVPQSRNICDISCDKFLDDDI	24	-0.24	0.65	0.65	-0.53	0.36	0.38	0.92	-3	3.98	2700.28	0.99	0.28	0.07
2534	QVPQSRNICDISCDKFLDDDIT	24	-0.26	0.66	0.66	-0.64	0.36	0.5	0.92	-4	3.86	2728.29	0.99	0.27	0.07
2535	VPQSRNICDISCDKFLDDDITD	24	-0.28	0.67	0.67	-0.75	0.36	0.61	0.92	-5	3.77	2756.3	0.99	0.27	0.05
2536	VPQSRNICDISCDKFLDDDITDD	24	-0.22	0.67	0.67	-0.42	0.31	0.53	0.83	-5	3.77	2741.33	0.98	0.26	0.09
2537	QSRNICDISCDKFLDDDITDDI	24	-0.23	0.67	0.67	-0.51	0.31	0.53	0.83	-5	3.77	2773.39	0.87	0.27	0.14
2538	SRNICDISCDKFLDDDITDDIM	24	-0.23	0.68	0.68	-0.34	0.31	0.49	0.83	-5	3.77	2779.41	0.99	0.32	0.13
2539	RNICDISCDKFLDDDITDDIMC	24	-0.19	0.68	0.68	-0.12	0.26	0.46	0.75	-5	3.77	2722.35	0.98	0.32	0.09
2540	NICDISCDKFLDDDITDDIMCA	24	-0.22	0.68	0.68	-0.25	0.41	0.57	0.79	-4	4	2763.45	0.99	0.29	0.12
2541	ICDISCDKFLDDDITDDIMCAK	24	-0.2	0.68	0.68	-0.23	0.46	0.57	0.71	-4	4	2735.44	0.98	0.32	0.25
2542	CDISCDKFLDDDITDDIMCAKK	24	-0.14	0.68	0.68	0.11	0.46	0.49	0.62	-4	4	2734.5	0.98	0.33	0.20
2543	CDISCDKFLDDDITDDIMCAKKI	24	-0.15	0.67	0.67	0.08	0.46	0.49	0.62	-4	4	2734.5	0.94	0.38	0.20
2544	DISCDKFLDDDITDDIMCAKKIL	24	-0.18	0.68	0.68	-0.17	0.46	0.66	0.67	-5	3.9	2746.45	0.80	0.37	0.04
2545	ISCDKFLDDDITDDIMCAKKILD	24	-0.12	0.68	0.68	0.16	0.46	0.46	0.62	-4	4	2744.53	0.93	0.37	0.04
2546	SCDKFLDDDITDDIMCAKKILDI	24	-0.2	0.67	0.67	-0.19	0.61	0.66	0.71	-3	4.22	2759.54	0.98	0.35	0.33
2547	CDKFLDDDITDDIMCAKKILDIK	24	-0.18	0.68	0.68	-0.17	0.61	0.65	0.67	-3	4.22	2729.52	0.80	0.37	0.17
2548	DKFLDDDITDDIMCAKKILDIKG	24	-0.15	0.68	0.68	-0.09	0.61	0.61	0.67	-3	4.22	2739.55	0.80	0.36	0.04
2549	KFLDDDITDDIMCAKKILDIKGID	24	-0.15	0.68	0.68	-0.09	0.61	0.61	0.67	-3	4.22	2739.55	0.98	0.36	0.04
2550	FLDDDITDDIMCAKKILDIKGIDY	24	-0.1	0.69	0.69	0.02	0.46	0.39	0.62	-4	4	2774.55	0.80	0.39	0.04
2551	LDDDITDDIMCAKKILDIKGIDYW	24	-0.11	0.68	0.68	-0.13	0.46	0.35	0.67	-4	4	2813.59	0.98	0.42	0.04
2552	DDDITDDIMCAKKILDIKGIDYWL	24	-0.11	0.68	0.68	-0.13	0.46	0.35	0.67	-4	4	2813.59	0.80	0.44	0.03
2553	DDITDDIMCAKKILDIKGIDYWLA	24	-0.07	0.67	0.67	0.09	0.46	0.21	0.62	-3	4.12	2769.58	0.80	0.48	0.04
2554	DITDDIMCAKKILDIKGIDYWLAH	24	-0.06	0.64	0.64	0.1	0.52	0.06	0.62	-1.5	4.67	2791.64	0.94	0.48	0.04
2555	TDDIMCAKKILDIKGIDYWLAHK	24	-0.08	0.63	0.63	0.08	0.67	0.06	0.67	0.5	7.05	2804.73	0.97	0.48	0.10
2556	TDDIMCAKKILDIKGIDYWLAHKA	24	-0.1	0.62	0.62	-0.03	0.67	0.12	0.67	0.5	7.05	2762.64	0.93	0.54	0.30
2557	DDIMCAKKILDIKGIDYWLAHKAL	24	-0.07	0.62	0.62	0.16	0.67	0.06	0.62	0.5	7.05	2774.7	0.94	0.56	0.53
2558	DIMCAKKILDIKGIDYWLAHKALC	24	-0.03	0.62	0.62	0.41	0.67	-0.11	0.58	1.5	8.32	2762.75	0.94	0.50	0.96

2559	MCAKKILDIKGIDYWLAHKALCT	24	-0.01	0.61	0.61	0.52	0.67	-0.25	0.58	2.5	8.87	2748.77	0.93	0.52	0.97
2560	MCAKKILDIKGIDYWLAHKALCTI	24	-0.07	0.61	0.61	0.19	0.72	-0.05	0.62	1.5	8.32	2764.72	0.93	0.51	0.93
2561	CAKKILDIKGIDYWLAHKALCTEI	24	-0.12	0.6	0.6	-0.05	0.88	0.13	0.71	2.5	8.84	2761.7	0.69	0.52	0.94
2562	AKKILDIKGIDYWLAHKALCTEKI	24	-0.1	0.6	0.6	0	0.88	0.1	0.71	2.5	9.05	2771.73	0.94	0.52	0.92
2563	KKILDIKGIDYWLAHKALCTEKLE	24	-0.14	0.61	0.61	-0.22	0.93	0.24	0.75	1.5	8.44	2829.77	0.98	0.51	0.12
2564	KILDIKGIDYWLAHKALCTEKLEC	24	-0.12	0.61	0.61	-0.2	0.83	0.12	0.75	0.5	7.06	2829.73	0.98	0.44	0.05
2565	LDIKGIDYWLAHKALCTEKLEQV	24	-0.06	0.6	0.6	-0.07	0.68	-0.14	0.71	-0.5	5.5	2887.77	0.93	0.45	0.06
2566	DIKGIDYWLAHKALCTEKLEQW	24	-0.07	0.59	0.59	-0.1	0.68	-0.14	0.71	-0.5	5.5	2887.77	0.93	0.40	0.20
2567	IKGIDYWLAHKALCTEKLEQWL	24	-0.09	0.6	0.6	-0.16	0.68	-0.11	0.71	-0.5	5.5	2877.74	0.94	0.44	0.20
2568	KGIDYWLAHKALCTEKLEQWLC	24	-0.09	0.59	0.59	-0.16	0.73	-0.11	0.71	-0.5	5.54	2891.77	0.93	0.43	0.08
2569	GIDYWLAHKALCTEKLEQWLCE	24	-0.16	0.59	0.59	-0.51	0.88	0.09	0.79	0.5	7.04	2906.78	0.93	0.46	0.13
2570	IDYWLAHKALCTEKLEQWLCEK	24	-0.09	0.59	0.59	-0.19	0.73	-0.11	0.71	-0.5	5.54	2891.77	0.83	0.44	0.37
2571	RFFVPLFLVGILFPAILAKQFTK	25	0.15	0.62	0.62	1.38	0.44	-0.83	0.44	3	10.07	2901.05	0.93	0.58	0.94
2572	FFVPLFLVGILFPAILAKQFTKC	25	0.11	0.62	0.62	1.16	0.49	-0.66	0.48	2	9.36	2898.97	0.98	0.59	0.78
2573	FVPLFLVGILFPAILAKQFTKCE	25	0.2	0.61	0.61	1.5	0.39	-0.85	0.32	1	8.54	2855.95	0.94	0.54	0.88
2574	VPLFLVGILFPAILAKQFTKCEL	25	0.17	0.6	0.6	1.35	0.39	-0.74	0.36	1	8.54	2795.85	0.94	0.51	0.94
2575	PLFLVGILFPAILAKQFTKCELS	25	0.12	0.6	0.6	1.1	0.44	-0.63	0.44	1	8.54	2776.81	0.93	0.47	0.38
2576	LFLVGILFPAILAKQFTKCELSQ	25	0.12	0.6	0.6	1.08	0.44	-0.64	0.44	1	8.54	2790.84	0.93	0.45	0.45
2577	FLVGILFPAILAKQFTKCELSQL	25	0.14	0.6	0.6	1.3	0.44	-0.72	0.44	1	8.54	2806.89	0.93	0.45	0.79
2578	LVGILFPAILAKQFTKCELSQLL	25	0.08	0.61	0.61	0.99	0.59	-0.52	0.52	2	9.25	2821.9	0.94	0.45	0.96
2579	VGILFPAILAKQFTKCELSQLLK	25	0.02	0.61	0.61	0.74	0.59	-0.3	0.56	1	8.51	2789.81	0.93	0.44	0.61
2580	VGILFPAILAKQFTKCELSQLLKD	25	0.03	0.62	0.62	0.77	0.59	-0.3	0.56	1	8.51	2789.81	0.93	0.46	0.86
2581	GILFPAILAKQFTKCELSQLLKDI	25	-0.02	0.62	0.62	0.46	0.59	-0.12	0.6	0	6.43	2805.76	0.83	0.45	0.85
2582	LFPAILAKQFTKCELSQLLKDID	25	-0.02	0.62	0.62	0.46	0.59	-0.12	0.6	0	6.43	2805.76	0.93	0.46	0.23
2583	FPAILAKQFTKCELSQLLKDIDG	25	-0.05	0.62	0.62	0.23	0.59	-0.14	0.64	0	6.43	2855.77	0.93	0.35	0.09
2584	PAILAKQFTKCELSQLLKDIDGY	25	-0.06	0.63	0.63	0.06	0.59	-0.07	0.64	0	6.43	2799.66	0.94	0.35	0.15
2585	AILAKQFTKCELSQLLKDIDGYG	25	-0.08	0.63	0.63	-0.07	0.59	0.03	0.64	0	6.43	2709.54	0.98	0.36	0.06
2586	ILAKQFTKCELSQLLKDIDGYG	25	-0.05	0.64	0.64	0.18	0.59	-0.04	0.64	0	6.43	2725.59	0.80	0.35	0.05
2587	AKQFTKCELSQLLKDIDGYGG	25	-0.05	0.64	0.64	0.18	0.59	-0.04	0.64	0	6.43	2725.59	0.98	0.34	0.04
2588	AKQFTKCELSQLLKDIDGYGGIA	25	-0.06	0.63	0.63	0.15	0.59	-0.04	0.64	0	6.43	2725.59	0.93	0.37	0.35
2589	KQFTKCELSQLLKDIDGYGGIAI	25	-0.08	0.63	0.63	-0.07	0.59	0.03	0.64	0	6.43	2709.54	0.61	0.36	0.03
2590	QFTKCELSQLLKDIDGYGGIALI	25	-0.12	0.63	0.63	-0.28	0.64	0.17	0.68	-1	4.79	2767.58	0.99	0.36	0.08
2591	FTKCELSQLLKDIDGYGGIALPI	25	-0.05	0.63	0.63	0.03	0.5	-0.02	0.6	-2	4.32	2752.57	0.94	0.37	0.04
2592	TKCELSQLLKDIDGYGGIALPEI	25	0.01	0.63	0.63	0.35	0.45	-0.1	0.52	-2	4.32	2737.6	0.86	0.35	0.06
2593	KCELSQLLKDIDGYGGIALPELI	25	-0.02	0.62	0.62	0.34	0.45	-0.04	0.52	-2	4.32	2693.56	0.99	0.37	0.11
2594	CELSQLLKDIDGYGGIALPELIC	25	-0.02	0.62	0.62	0.34	0.45	-0.04	0.52	-2	4.32	2693.56	0.99	0.34	0.03
2595	ELSQLLKDIDGYGGIALPELICT	25	0.04	0.63	0.63	0.57	0.3	-0.22	0.44	-3	3.92	2696.58	0.86	0.34	0.04
2596	LSQLLKDIDGYGGIALPELICTM	25	0.06	0.63	0.63	0.58	0.3	-0.28	0.44	-3	3.92	2740.62	0.61	0.34	0.04
2597	SQLLKDIDGYGGIALPELICTMF	25	0.07	0.6	0.6	0.59	0.31	-0.42	0.44	-1.5	4.54	2748.65	0.97	0.39	0.03
2598	QLLKDIDGYGGIALPELICTMFH	25	0.04	0.6	0.6	0.41	0.31	-0.36	0.48	-1.5	4.54	2736.59	0.99	0.39	0.03
2599	LLKDIDGYGGIALPELICTMFHT	25	0.04	0.6	0.6	0.41	0.31	-0.36	0.48	-1.5	4.54	2736.59	0.94	0.35	0.04
2600	LKDIDGYGGIALPELICTMFHTS	25	0.08	0.6	0.6	0.54	0.26	-0.37	0.4	-1.5	4.54	2665.51	0.93	0.33	0.03
2601	KDIDGYGGIALPELICTMFHTSG	25	0.05	0.61	0.61	0.33	0.26	-0.39	0.44	-1.5	4.54	2715.52	0.93	0.33	0.04
2602	DIDGYGGIALPELICTMFHTSGY	25	0	0.62	0.62	0.04	0.26	-0.2	0.48	-2.5	4.22	2717.44	0.93	0.32	0.05
2603	DGYGGIALPELICTMFHTSGYD	25	0.04	0.61	0.61	0.17	0.11	-0.33	0.44	-3.5	3.84	2690.37	0.94	0.29	0.05
2604	GYGGIALPELICTMFHTSGYDT	25	0.04	0.61	0.61	0.17	0.16	-0.44	0.48	-2.5	4.03	2703.42	0.93	0.28	0.04
2605	GYGGIALPELICTMFHTSGYDTC	25	0.02	0.6	0.6	0.06	0.16	-0.39	0.48	-2.5	4.03	2661.33	0.94	0.27	0.04
2606	YGGIALPELICTMFHTSGYDTQ	25	0.08	0.6	0.6	0.38	0.16	-0.58	0.44	-1.5	4.36	2659.41	0.83	0.27	0.07
2607	GGIALPELICTMFHTSGYDTQA	25	0.1	0.6	0.6	0.56	0.16	-0.64	0.44	-1.5	4.36	2701.49	0.93	0.31	0.05
2608	GIALPELICTMFHTSGYDTQAIV	25	0.07	0.6	0.6	0.48	0.21	-0.43	0.44	-2.5	4.14	2667.43	0.83	0.33	0.17
2609	IALPELICTMFHTSGYDTQAIVE	25	0.04	0.6	0.6	0.35	0.21	-0.42	0.52	-2.5	4.14	2724.48	0.95	0.33	0.05
2610	ALPELICTMFHTSGYDTQAIVEN	25	0.01	0.61	0.61	0.23	0.21	-0.42	0.6	-2.5	4.14	2781.53	0.93	0.33	0.03
2611	LPELICTMFHTSGYDTQAIVENN	25	-0.05	0.61	0.61	-0.09	0.26	-0.22	0.64	-3.5	4	2797.48	0.94	0.33	0.04
2612	PELICTMFHTSGYDTQAIVENNI	25	-0.07	0.61	0.61	-0.2	0.26	-0.19	0.68	-3.5	4	2813.48	0.93	0.32	0.05
2613	ELICTMFHTSGYDTQAIVENNES	25	-0.1	0.61	0.61	-0.38	0.26	-0.14	0.72	-3.5	4	2801.42	0.87	0.28	0.04
2614	LICTMFHTSGYDTQAIVENNEST	25	-0.12	0.62	0.62	-0.45	0.31	-0.02	0.76	-4.5	3.91	2833.42	0.80	0.29	0.04
2615	CTMFHTSGYDTQAIVENNESTI	25	-0.09	0.62	0.62	-0.36	0.26	-0.23	0.76	-3.5	4	2867.48	0.98	0.33	0.03
2616	TMFHTSGYDTQAIVENNESTEY	25	-0.11	0.63	0.63	-0.53	0.26	-0.16	0.76	-3.5	4	2811.37	0.93	0.34	0.13
2617	MFHTSGYDTQAIVENNESTEY	25	-0.11	0.62	0.62	-0.56	0.26	-0.16	0.76	-3.5	4	2811.37	0.94	0.33	0.03
2618	MFHTSGYDTQAIVENNESTEYG	25	-0.09	0.62	0.62	-0.55	0.26	-0.22	0.76	-3.5	4	2855.41	0.93	0.36	0.03
2619	HTSGYDTQAIVENNESTEYGL	25	-0.11	0.63	0.63	-0.66	0.31	-0.19	0.8	-3.5	4	2882.44	0.93	0.34	0.03
2620	HTSGYDTQAIVENNESTEYGLF	25	-0.09	0.63	0.63	-0.56	0.31	-0.21	0.8	-3.5	4	2864.41	0.94	0.35	0.04
2621	TSGYDTQAIVENNESTEYGLFC	25	-0.13	0.62	0.62	-0.7	0.31	-0.1	0.84	-3.5	4	2804.31	0.93	0.33	0.08
2622	SGYDTQAIVENNESTEYGLFQIS	25	-0.14	0.65	0.65	-0.71	0.25	-0.07	0.88	-4	3.51	2781.27	0.93	0.33	0.03

2623	GYDTQAIVENNESTEYGLFQISI	25	-0.17	0.66	0.66	-0.84	0.4	0.06	0.92	-3	4.01	2808.34	0.93	0.34	0.03
2624	YDTQAIVENNESTEYGLFQISNI	25	-0.14	0.66	0.66	-0.66	0.4	-0.02	0.88	-3	4.01	2834.43	0.83	0.39	0.09
2625	DTQAIVENNESTEYGLFQISNKL	25	-0.13	0.65	0.65	-0.68	0.4	-0.16	0.92	-3	4.01	2963.59	0.93	0.38	0.04
2626	TQAIVENNESTEYGLFQISNKLW	25	-0.13	0.64	0.64	-0.52	0.4	-0.1	0.88	-3	4.01	2903.55	0.94	0.38	0.09
2627	QAIVENNESTEYGLFQISNKLWC	25	-0.15	0.64	0.64	-0.54	0.55	-0.1	0.92	-1	4.79	2916.64	0.93	0.38	0.23
2628	AIVENNESTEYGLFQISNKLWCK	25	-0.15	0.64	0.64	-0.54	0.55	-0.08	0.92	-1	4.79	2902.61	0.98	0.40	0.04
2629	VENNESTEYGLFQISNKLWCKS	25	-0.14	0.64	0.64	-0.44	0.5	-0.07	0.88	-1	4.79	2861.55	0.80	0.45	0.04
2630	ENNESTEYGLFQISNKLWCKSS	25	-0.17	0.64	0.64	-0.65	0.55	-0.04	0.96	-1	4.79	2918.61	0.87	0.43	0.04
2631	NNESTEYGLFQISNKLWCKSSQ	25	-0.18	0.64	0.64	-0.66	0.55	-0.03	0.96	-1	4.79	2904.58	0.93	0.41	0.04
2632	NESTEYGLFQISNKLWCKSSQ	25	-0.2	0.63	0.63	-0.89	0.55	0.03	0.96	-1	4.79	2902.56	0.61	0.41	0.05
2633	ESTEYGLFQISNKLWCKSSQV	25	-0.21	0.63	0.63	-0.89	0.55	-0.08	1	0	6.45	2901.58	0.94	0.37	0.03
2634	STEYGLFQISNKLWCKSSQVP	25	-0.19	0.62	0.62	-0.78	0.55	-0.08	0.96	0	6.45	2874.55	0.99	0.38	0.03
2635	TEYGLFQISNKLWCKSSQVPQ	25	-0.24	0.62	0.62	-0.82	0.64	0.03	1.04	1	8.51	2916.63	0.61	0.41	0.03
2636	EYGLFQISNKLWCKSSQVPQS	25	-0.24	0.62	0.62	-0.82	0.59	-0.08	1.08	2	9.25	2901.62	0.97	0.41	0.03
2637	YGLFQISNKLWCKSSQVPQSR	25	-0.2	0.63	0.63	-0.61	0.59	-0.16	1.04	2	9.25	2927.71	0.97	0.37	0.04
2638	GLFQISNKLWCKSSQVPQSRN	25	-0.19	0.63	0.63	-0.48	0.59	-0.19	1	2	8.94	2929.74	0.61	0.38	0.35
2639	LFQISNKLWCKSSQVPQSRNI	25	-0.19	0.63	0.63	-0.48	0.54	-0.19	1	2	8.94	2915.71	0.99	0.40	0.17
2640	LFQISNKLWCKSSQVPQSRNIC	25	-0.17	0.63	0.63	-0.25	0.54	-0.17	0.96	2	8.98	2865.7	0.98	0.41	0.35
2641	FQISNKLWCKSSQVPQSRNICD	25	-0.18	0.63	0.63	-0.27	0.54	-0.16	1	2	8.98	2895.72	0.94	0.40	0.11
2642	QISNKLWCKSSQVPQSRNICDIS	25	-0.2	0.63	0.63	-0.32	0.54	-0.12	1	2	8.77	2885.69	0.61	0.39	0.50
2643	SNKLWCKSSQVPQSRNICDISC	25	-0.25	0.63	0.63	-0.57	0.54	0.1	1.04	1	8.24	2853.6	0.99	0.41	0.11
2644	NKLWCKSSQVPQSRNICDISCD	25	-0.27	0.63	0.63	-0.59	0.64	0.21	1.04	2	8.75	2853.64	0.97	0.39	0.24
2645	KLWCKSSQVPQSRNICDISCD	25	-0.28	0.63	0.63	-0.66	0.64	0.18	1.04	2	8.75	2887.65	0.99	0.38	0.37
2646	KLWCKSSQVPQSRNICDISCDK	25	-0.24	0.63	0.63	-0.47	0.64	0.1	1	2	8.75	2913.74	0.99	0.39	0.83
2647	WCKSSQVPQSRNICDISCDKF	25	-0.25	0.63	0.63	-0.47	0.64	0.21	0.96	1	8.23	2914.72	0.97	0.27	0.71
2648	WCKSSQVPQSRNICDISCDKFLI	25	-0.23	0.64	0.64	-0.46	0.49	0.21	0.92	-1	4.58	2901.63	0.97	0.25	0.18
2649	CKSSQVPQSRNICDISCDKFLDI	25	-0.28	0.65	0.65	-0.75	0.49	0.4	0.96	-2	4.29	2903.55	0.97	0.25	0.10
2650	KSSQVPQSRNICDISCDKFLDD	25	-0.27	0.65	0.65	-0.53	0.49	0.46	0.92	-2	4.29	2830.5	0.86	0.27	0.08
2651	SSQVPQSRNICDISCDKFLDDD	25	-0.28	0.65	0.65	-0.66	0.49	0.49	0.96	-2	4.29	2828.47	0.99	0.26	0.08
2652	SQVPQSRNICDISCDKFLDDIT	25	-0.26	0.65	0.65	-0.64	0.34	0.49	0.92	-4	3.86	2815.38	0.99	0.24	0.08
2653	QVPQSRNICDISCDKFLDDDITD	25	-0.28	0.66	0.66	-0.75	0.34	0.6	0.92	-5	3.77	2843.39	0.99	0.25	0.06
2654	VPQSRNICDISCDKFLDDDITDI	25	-0.24	0.67	0.67	-0.54	0.34	0.51	0.88	-5	3.77	2869.48	0.99	0.25	0.07
2655	PQSRNICDISCDKFLDDDITDDI	25	-0.2	0.67	0.67	-0.32	0.29	0.45	0.8	-5	3.77	2872.54	0.98	0.26	0.10
2656	QSRNICDISCDKFLDDDITDDIM	25	-0.22	0.67	0.67	-0.39	0.29	0.47	0.8	-5	3.77	2876.54	0.87	0.27	0.14
2657	SRNICDISCDKFLDDDITDDIMC	25	-0.21	0.68	0.68	-0.26	0.29	0.45	0.8	-5	3.77	2850.5	0.98	0.31	0.13
2658	RNICDISCDKFLDDDITDDIMCA	25	-0.23	0.68	0.68	-0.27	0.39	0.56	0.8	-4	4	2850.54	0.99	0.29	0.07
2659	NICDISCDKFLDDDITDDIMCAK	25	-0.26	0.68	0.68	-0.4	0.54	0.67	0.84	-3	4.22	2891.64	0.98	0.31	0.36
2660	ICDISCDKFLDDDITDDIMCAKK	25	-0.16	0.68	0.68	-0.04	0.44	0.48	0.68	-4	4	2848.62	0.98	0.31	0.17
2661	CDISCDKFLDDDITDDIMCAKKI	25	-0.11	0.67	0.67	0.26	0.44	0.4	0.6	-4	4	2847.68	0.98	0.36	0.40
2662	DISCDKFLDDDITDDIMCAKKIL	25	-0.17	0.68	0.68	-0.06	0.44	0.59	0.64	-5	3.9	2849.6	0.80	0.36	0.04
2663	DISCDKFLDDDITDDIMCAKKILD	25	-0.14	0.68	0.68	0.02	0.44	0.56	0.64	-5	3.9	2859.63	0.94	0.36	0.04
2664	SCDKFLDDDITDDIMCAKKILDI	25	-0.16	0.68	0.68	0	0.59	0.56	0.68	-3	4.22	2872.72	0.98	0.33	0.30
2665	CDKFLDDDITDDIMCAKKILDIK	25	-0.18	0.68	0.68	-0.2	0.59	0.63	0.68	-3	4.22	2816.61	0.98	0.33	0.32
2666	CDKFLDDDITDDIMCAKKILDIKG	25	-0.14	0.68	0.68	0.02	0.59	0.55	0.64	-3	4.22	2842.7	0.80	0.33	0.05
2667	DKFLDDDITDDIMCAKKILDIKGI	25	-0.17	0.69	0.69	-0.22	0.59	0.71	0.68	-4	4.1	2854.65	0.80	0.32	0.04
2668	KFLDDDITDDIMCAKKILDIKGID	25	-0.14	0.69	0.69	-0.14	0.59	0.5	0.68	-3	4.22	2902.74	0.98	0.36	0.06
2669	LDDDITDDIMCAKKILDIKGIDY	25	-0.08	0.68	0.68	-0.02	0.44	0.24	0.64	-4	4	2960.78	0.80	0.46	0.04
2670	DDDITDDIMCAKKILDIKGIDYW	25	-0.09	0.67	0.67	0.02	0.44	0.27	0.64	-4	4	2926.77	0.98	0.42	0.03
2671	DDITDDIMCAKKILDIKGIDYWL	25	-0.1	0.67	0.67	-0.06	0.44	0.32	0.64	-4	4	2884.68	0.80	0.47	0.03
2672	DITDDIMCAKKILDIKGIDYWLA	25	-0.09	0.64	0.64	-0.04	0.5	0.18	0.64	-2.5	4.39	2906.74	0.94	0.47	0.03
2673	ITDDIMCAKKILDIKGIDYWLAH	25	-0.1	0.64	0.64	-0.06	0.65	0.18	0.68	-0.5	5.42	2919.83	0.94	0.46	0.05
2674	TDDIMCAKKILDIKGIDYWLAHK	25	-0.06	0.63	0.63	0.15	0.65	0.04	0.64	0.5	7.05	2875.82	0.97	0.54	0.27
2675	DDIMCAKKILDIKGIDYWLAHKA	25	-0.07	0.62	0.62	0.12	0.65	0.04	0.64	0.5	7.05	2875.82	0.93	0.54	0.24
2676	DIMCAKKILDIKGIDYWLAHKAL	25	-0.06	0.62	0.62	0.25	0.65	0.02	0.6	0.5	7.03	2877.85	0.94	0.50	0.74
2677	IMCAKKILDIKGIDYWLAHKALC	25	-0.04	0.62	0.62	0.36	0.65	-0.12	0.6	1.5	8.32	2863.87	0.94	0.51	0.76
2678	MCAKKILDIKGIDYWLAHKALCT	25	-0.04	0.61	0.61	0.36	0.7	-0.12	0.6	1.5	8.32	2877.9	0.93	0.51	0.92
2679	CAKKILDIKGIDYWLAHKALCTE	25	-0.11	0.61	0.61	0.03	0.84	0.07	0.68	2.5	8.84	2892.91	0.93	0.52	0.91
2680	AKKILDIKGIDYWLAHKALCTEK	25	-0.1	0.6	0.6	0.1	0.84	0.05	0.68	2.5	8.84	2874.88	0.69	0.51	0.96
2681	KKILDIKGIDYWLAHKALCTEKL	25	-0.12	0.6	0.6	-0.14	0.89	0.21	0.72	1.5	8.44	2900.86	0.94	0.55	0.51
2682	KILDIKGIDYWLAHKALCTEKLE	25	-0.16	0.61	0.61	-0.35	0.94	0.24	0.8	1.5	8.44	2957.92	0.98	0.44	0.08
2683	LDIKGIDYWLAHKALCTEKLEQ	25	-0.1	0.6	0.6	-0.23	0.8	-0.02	0.76	0.5	7.06	3015.96	0.98	0.44	0.08
2684	DIKGIDYWLAHKALCTEKLEQW	25	-0.04	0.6	0.6	0.08	0.65	-0.21	0.68	-0.5	5.5	3000.95	0.93	0.45	0.14
2685	DIKGIDYWLAHKALCTEKLEQWI	25	-0.07	0.59	0.59	0	0.65	-0.18	0.68	-0.5	5.5	2990.92	0.93	0.41	0.24
2686	KGIDYWLAHKALCTEKLEQWL	25	-0.11	0.6	0.6	-0.29	0.7	0.02	0.72	-1.5	4.9	3006.87	0.94	0.43	0.03

2687	GIDYWLAHKALCTEKLEQWLCI	25	-0.13	0.6	0.6	-0.31	0.85	0.02	0.76	0.5	7.04	3019.96	0.93	0.44	0.11
2688	GIDYWLAHKALCTEKLEQWLCE	25	-0.13	0.59	0.59	-0.34	0.85	0.02	0.76	0.5	7.04	3019.96	0.93	0.50	0.43

Table S2. Processed and raw SWATH-intensity proteomic data sets of ALA-A2 treated (ACP) vs. untreated (Ctrl) A549 cells.

					Process SWATH-intensity dataset (log2 transformed, VSN normalized, median imputed)																		Raw SWATH-intensity dataset																	
ID	Uniprot ID	Entry names	species	Gene symbol	ACP1_1	ACP1_2	ACP1_3	ACP2_1	ACP2_2	ACP2_3	ACP3_1	ACP3_2	ACP3_3	Ctrl1_1	Ctrl1_2	Ctrl1_3	Ctrl2_1	Ctrl2_2	Ctrl2_3	Ctrl3_1	Ctrl3_2	Ctrl3_3	ACP1_1	ACP1_2	ACP1_3	ACP2_1	ACP2_2	ACP2_3	ACP3_1	ACP3_2	ACP3_3	Ctrl1_1	Ctrl1_2	Ctrl1_3	Ctrl2_1	Ctrl2_2	Ctrl2_3	Ctrl3_1	Ctrl3_2	Ctrl3_3
1	Q15149	PLEC	HUMAN	*PLEC*	15.75	14.46	14.85	15.38	15.12	14.87	14.61	14.88	15.56	15.54	14.29	13.99	15.07	14.30	13.29	14.54	14.92	14.17	4.73E+04	7.28E+04	1.31E+05	1.19E+04	7.47E+03	8.02E+03	6.98E+03	7.90E+03	8.73E+03	1.11E+05	1.40E+04	3.03E+04	1.00E+05	3.30E+04	9.36E+04	1.52E+04	4.13E+04	6.04E+04
2	P00352	AL1A1	HUMAN	*ALDH1A1*	16.06	17.09	17.21	15.18	14.79	14.63	15.01	14.97	14.68	17.14	16.68	16.18	15.60	17.25	17.77	15.49	16.40	17.17	5.86E+04	4.51E+05	6.75E+05	1.03E+04	5.95E+03	6.77E+03	9.21E+03	8.38E+03	4.75E+03	3.36E+05	7.30E+04	1.42E+05	1.45E+05	2.54E+05	2.19E+06	2.94E+04	1.14E+05	4.87E+05
3	P21333	FLNA	HUMAN	*FLNA*	14.56	14.85	14.91	14.30	12.99	14.01	13.99	14.50	13.57	15.24	14.85	14.61	14.13	14.46	15.15	14.33	15.01	14.89	2.07E+04	9.54E+04	1.37E+05	5.65E+03	1.73E+03	4.45E+03	4.56E+03	6.07E+03	2.20E+03	8.96E+04	2.06E+04	4.72E+04	5.20E+04	3.68E+04	3.50E+05	1.32E+04	4.39E+04	9.98E+04
4	P07355	ANXA2	HUMAN	*ANXA2*	16.19	18.76	17.75	15.11	15.35	14.77	16.05	15.75	15.29	16.69	16.26	17.38	16.95	16.41	16.97	16.06	17.16	17.19	6.40E+04	1.44E+06	9.87E+05	9.83E+03	8.81E+03	7.49E+03	1.88E+04	1.44E+04	7.21E+03	2.46E+05	5.46E+04	3.28E+05	3.72E+05	1.42E+05	1.25E+06	4.34E+04	1.94E+05	4.91E+05
5	O43707	ACTN4	HUMAN	*ACTN4*	13.29	12.92	14.35	13.42	13.97	12.86	13.63	12.67	12.39	15.24	13.57	13.62	12.80	13.43	11.23	13.47	13.91	11.61	8.65E+03	2.47E+04	9.22E+04	3.08E+03	3.38E+03	2.01E+03	3.56E+03	1.73E+03	9.90E+02	8.97E+04	8.53E+03	2.32E+04	2.04E+04	1.80E+04	1.89E+04	7.30E+03	2.05E+04	9.75E+03
6	P14618	KPYM	HUMAN	*PKM*	15.55	15.98	17.30	15.41	14.69	14.84	15.38	15.15	14.34	18.16	16.08	15.82	15.77	16.05	17.68	15.03	16.46	17.79	4.11E+04	2.09E+05	7.21E+05	1.21E+04	5.57E+03	7.83E+03	1.18E+04	9.48E+03	3.76E+03	6.82E+05	4.81E+04	1.10E+05	1.64E+05	1.10E+05	2.05E+06	2.14E+04	1.19E+05	7.44E+05
7	P08238	HS90B	HUMAN	*HSP90AB1*	15.29	13.59	15.05	13.82	12.34	13.68	14.26	14.31	11.24	14.46	14.61	14.92	16.20	15.22	16.16	15.55	15.08	15.76	3.43E+04	3.96E+04	1.51E+05	4.04E+03	1.11E+03	3.53E+03	5.49E+03	5.30E+03	4.58E+02	5.21E+04	1.74E+04	5.86E+04	2.21E+05	6.22E+04	7.11E+05	3.06E+04	4.59E+04	1.82E+05
8	P05787	K2C8	HUMAN	*KRT8*	14.38	16.80	18.13	14.61	12.99	14.14	14.65	15.03	13.02	17.36	15.57	17.45	16.21	15.96	16.92	14.73	15.11	17.10	1.84E+04	3.69E+05	1.28E+06	6.97E+03	1.73E+03	4.84E+03	7.14E+03	8.71E+03	1.51E+03	3.92E+05	3.39E+04	3.44E+05	2.22E+05	1.03E+05	1.20E+06	1.74E+04	4.69E+04	4.63E+05
9	P05783	K1C18	HUMAN	*KRT18*	15.06	16.37	17.52	13.64	13.31	14.38	13.55	14.00	12.16	18.22	17.54	17.67	15.12	16.20	15.26	14.89	15.03	17.53	2.94E+04	2.74E+05	8.41E+05	3.59E+03	2.16E+03	5.72E+03	3.36E+03	4.29E+03	8.47E+02	7.13E+05	1.32E+05	4.00E+05	1.04E+05	1.23E+05	3.79E+05	1.94E+04	4.43E+04	6.24E+05
10	O60218	AK1BA	HUMAN	*AKR1B10*	14.39	16.98	15.28	15.38	14.36	13.78	14.80	13.94	13.52	18.35	16.68	17.94	14.43	14.91	16.79	15.02	15.90	17.33	1.85E+04	4.20E+05	1.77E+05	1.19E+04	4.44E+03	3.79E+03	7.93E+03	4.12E+03	2.13E+03	7.79E+05	7.27E+04	4.82E+05	6.41E+04	5.00E+04	1.10E+06	2.12E+04	8.13E+04	5.40E+05
11	P11413	G6PD	HUMAN	*G6PD*	15.99	15.38	16.60	13.96	13.71	14.12	14.29	14.83	12.52	14.29	14.71	14.43	16.17	13.96	16.44	14.70	15.27	13.96	5.58E+04	1.38E+05	4.44E+05	4.46E+03	2.84E+03	4.77E+03	5.58E+03	7.62E+03	1.08E+03	4.64E+04	1.87E+04	4.14E+04	2.16E+05	2.59E+04	8.67E+05	1.70E+04	5.24E+04	5.21E+04
12	P63261	ACTG	HUMAN	*ACTG1*	13.23	14.78	14.89	12.28	14.75	13.29	11.78	11.09	14.75	19.41	17.74	14.72	15.18	15.85	18.44	13.87	14.14	17.52	8.32E+03	9.09E+04	1.35E+05	1.42E+03	NA	2.70E+03	1.01E+03	6.09E+02	NA	1.63E+06	1.52E+05	5.10E+04	1.08E+05	9.60E+04	3.48E+06	9.64E+03	2.40E+04	6.20E+05
13	P07437	TBB5	HUMAN	*TUBB*	12.55	13.96	11.56	11.80	10.63	12.03	11.28	11.66	11.22	11.99	13.27	14.73	12.85	13.07	13.04	13.56	16.06	13.41	5.25E+03	5.15E+04	1.24E+04	1.02E+03	3.58E+02	1.15E+03	7.25E+02	8.83E+02	4.51E+02	9.08E+03	6.95E+03	5.14E+04	2.11E+04	1.40E+04	7.76E+04	7.78E+03	9.06E+04	3.54E+04
14	Q00610	CLH1	HUMAN	*CLTC*	15.04	15.07	15.01	14.83	13.85	13.97	14.66	14.39	13.26	14.93	15.13	14.39	15.41	14.62	16.05	14.61	14.77	15.91	2.89E+04	1.11E+05	1.47E+05	8.10E+03	3.12E+03	4.32E+03	7.23E+03	5.63E+03	1.79E+03	7.24E+04	2.50E+04	4.03E+04	1.27E+05	4.09E+04	6.58E+05	1.60E+04	3.72E+04	2.02E+05
15	P06733	ENOA	HUMAN	*ENO1*	13.95	14.93	13.65	13.60	13.96	13.20	14.37	13.68	12.37	16.02	13.78	12.69	15.64	14.63	13.14	14.24	14.57	16.02	1.36E+04	1.01E+05	5.65E+04	3.49E+03	3.37E+03	2.54E+03	5.92E+03	3.46E+03	9.75E+02	1.55E+05	9.88E+03	1.17E+04	1.49E+05	4.13E+04	8.35E+04	1.24E+04	3.23E+04	2.19E+05
16	P68363	TBA1B	HUMAN	*TUBA1B*	14.41	16.53	15.43	12.32	11.78	11.10	11.88	12.29	11.99	14.01	15.86	13.90	15.38	16.70	14.31	14.03	15.67	15.06	1.87E+04	3.06E+05	1.96E+05	1.45E+03	7.65E+02	6.25E+02	1.08E+03	1.34E+03	7.53E+02	3.81E+04	4.14E+04	2.84E+04	1.25E+05	1.74E+05	1.94E+05	1.08E+04	6.91E+04	1.12E+05
17	P11142	HSP7C	HUMAN	*HSPA8*	15.68	15.53	16.26	14.27	13.53	13.84	13.98	14.07	12.70	18.78	15.70	15.77	16.46	14.34	17.06	14.98	16.36	17.09	4.49E+04	1.53E+05	3.51E+05	5.53E+03	2.50E+03	3.94E+03	4.50E+03	4.51E+03	1.22E+03	1.05E+06	3.70E+04	1.06E+05	2.64E+05	3.39E+04	1.34E+06	2.06E+04	1.12E+05	4.58E+05
18	P08670	VIME	HUMAN	*VIM*	14.31	17.50	17.14	14.00	12.58	13.43	12.80	13.23	13.08	16.04	15.94	16.68	16.26	15.46	17.21	14.57	16.46	16.59	1.74E+04	6.02E+05	6.43E+05	4.59E+03	1.31E+03	2.98E+03	2.01E+03	2.54E+03	1.57E+03	1.56E+05	4.38E+04	2.00E+05	2.30E+05	7.33E+04	1.47E+06	1.56E+04	1.19E+05	3.24E+05
19	P35579	MYH9	HUMAN	*MYH9*	15.34	14.17	15.12	14.69	16.13	15.27	14.60	15.27	13.65	15.71	15.20	14.43	15.24	15.85	14.21	14.85	14.72	14.93	3.56E+04	5.94E+04	1.58E+05	7.38E+03	1.51E+04	1.06E+04	6.92E+03	1.03E+04	2.34E+03	1.24E+05	2.62E+04	4.16E+04	1.13E+05	9.63E+04	1.81E+05	1.89E+04	3.58E+04	1.02E+05
20	P10809	CH60	HUMAN	*HSPD1*	14.57	14.92	14.19	14.06	14.10	14.72	14.16	14.15	13.70	14.38	16.73	14.88	14.57	14.74	15.45	13.82	15.45	15.00	2.09E+04	1.00E+05	8.24E+04	4.77E+03	3.70E+03	7.21E+03	5.10E+03	4.77E+03	2.41E+03	4.94E+04	7.57E+04	5.72E+04	7.08E+04	4.45E+04	4.33E+05	9.28E+03	5.92E+04	1.08E+05
21	O60701	UGDH	HUMAN	*UGDH*	14.53	14.98	14.64	14.90	13.85	14.93	14.29	14.60	13.02	14.09	14.83	14.43	14.46	15.30	14.48	15.63	14.67	16.12	2.04E+04	1.04E+05	1.13E+05	8.50E+03	3.11E+03	8.33E+03	5.61E+03	6.49E+03	1.52E+03	4.02E+04	2.03E+04	4.15E+04	6.58E+04	6.59E+04	2.19E+05	3.24E+04	3.45E+04	2.33E+05
22	O75369	FLNB	HUMAN	*FLNB*	13.83	13.45	13.03	12.83	12.58	12.38	11.84	12.70	12.13	13.42	14.35	13.16	13.42	13.54	14.70	14.02	13.58	13.65	1.25E+04	3.60E+04	3.62E+04	2.05E+03	1.31E+03	1.46E+03	1.05E+03	1.77E+03	8.30E+02	2.52E+04	1.46E+04	1.66E+04	3.17E+04	1.93E+04	2.56E+05	1.07E+04	1.63E+04	4.19E+04
23	P00338	LDHA	HUMAN	*LDHA*	13.63	14.02	13.24	13.23	12.43	12.83	12.94	13.58	12.79	12.22	13.39	12.15	12.07	13.74	12.86	13.87	12.73	13.42	1.10E+04	5.36E+04	4.21E+04	2.71E+03	1.18E+03	1.98E+03	2.22E+03	3.23E+03	1.29E+03	1.07E+04	7.53E+03	7.71E+03	1.21E+04	2.23E+04	6.81E+04	9.59E+03	9.09E+03	3.55E+04
24	P29401	TKT	HUMAN	*TKT*	14.67	14.88	14.20	13.86	14.97	13.47	13.78	13.82	13.82	16.84	15.62	15.26	15.91	14.48	15.87	14.51	16.38	15.41	2.23E+04	9.73E+04	8.28E+04	4.15E+03	6.75E+03	3.07E+03	3.93E+03	3.80E+03	2.62E+03	2.74E+05	3.50E+04	7.45E+04	1.80E+05	3.72E+04	5.82E+05	1.49E+04	1.13E+05	1.43E+05
25	P04083	ANXA1	HUMAN	*ANXA1*	16.25	16.96	16.46	15.74	14.97	14.63	14.86	14.59	13.41	16.29	16.16	16.14	14.63	16.32	16.61	15.08	14.66	16.05	6.68E+04	4.12E+05	4.02E+05	1.52E+04	6.78E+03	6.81E+03	8.30E+03	6.46E+03	1.98E+03	1.87E+05	5.10E+04	1.38E+05	7.41E+04	1.33E+05	9.76E+05	2.21E+04	3.44E+04	2.23E+05
26	P13639	EF2	HUMAN	*EEF2*	14.27	15.70	14.88	13.51	12.94	12.73	12.72	13.24	12.77	11.99	12.65	13.48	12.19	12.72	14.44	13.43	14.60	14.14	1.70E+04	1.73E+05	1.34E+05	3.28E+03	1.67E+03	1.85E+03	1.91E+03	2.56E+03	1.28E+03	9.12E+03	4.57E+03	2.10E+04	1.32E+04	1.10E+04	2.13E+05	7.09E+03	3.31E+04	5.88E+04
27	P08729	K2C7	HUMAN	*KRT7*	13.71	14.77	14.46	13.78	11.72	13.07	12.59	12.51	12.87	13.74	13.39	14.97	13.71	12.94	14.41	13.79	13.52	14.03	1.16E+04	9.02E+04	9.99E+04	3.94E+03	7.36E+02	2.33E+03	1.75E+03	1.55E+03	1.37E+03	3.15E+04	7.53E+03	6.07E+04	3.89E+04	1.28E+04	2.09E+05	9.10E+03	1.57E+04	5.46E+04
28	P52209	6PGD	HUMAN	*PGD*	12.08	11.36	12.17	12.54	11.07	10.99	12.42	12.57	12.18	12.23	12.41	11.97	13.26	12.13	13.36	11.80	12.80	12.36	3.81E+03	8.18E+03	1.95E+04	1.69E+03	4.77E+02	5.83E+02	1.56E+03	1.62E+03	8.59E+02	1.08E+04	3.87E+03	6.67E+03	2.82E+04	7.29E+03	9.85E+04	2.36E+03	9.54E+03	1.68E+04
29	P49327	FAS	HUMAN	*FASN*	13.77	14.13	14.41	13.41	13.94	14.19	13.25	13.65	14.11	13.94	14.41	12.14	14.83	13.69	13.68	14.16	13.50	13.66	1.20E+04	5.78E+04	9.63E+04	3.06E+03	3.31E+03	5.01E+03	2.74E+03	3.39E+03	3.19E+03	3.62E+04	1.52E+04	7.63E+03	8.49E+04	2.16E+04	1.23E+05	1.17E+04	1.54E+04	4.22E+04
30	P07900	HS90A	HUMAN	*HSP90AA1*	13.54	13.44	14.13	13.63	11.14	13.01	12.65	13.69	11.11	15.07	14.34	15.27	16.39	13.46	16.71	13.63	13.87	13.24	1.03E+04	3.57E+04	7.89E+04	3.56E+03	5.00E+02	2.23E+03	1.82E+03	3.47E+03	4.21E+02	7.99E+04	1.45E+04	7.49E+04	2.52E+05	1.83E+04	1.04E+06	8.16E+03	2.00E+04	3.13E+04
31	P42704	LPPRC	HUMAN	*LRPPRC*	10.66	10.56	9.19	9.98	9.98	10.91	11.70	9.86	10.48	8.45	8.30	9.99	10.69	9.34	10.27	8.58	9.06	8.44	1.51E+03	4.53E+03	1.50E+03	3.16E+02	NA	5.54E+02	9.57E+02	2.83E+02	2.80E+02	4.23E+02	3.17E+02	8.91E+02	4.29E+03	1.06E+03	7.43E+03	3.38E+02	8.29E+02	6.35E+02
32	P78527	PRKDC	HUMAN	*PRKDC*	13.42	12.32	13.11	13.69	13.54	12.78	14.18	14.35	14.14	12.76	14.29	13.27	13.90	14.63	11.53	14.33	13.69	12.88	9.47E+03	1.62E+04	3.83E+04	3.71E+03	2.52E+03	1.92E+03	5.17E+03	5.45E+03	3.27E+03	1.57E+04	1.40E+04	1.80E+04	4.43E+04	4.14E+04	2.42E+04	1.32E+04	1.76E+04	2.43E+04
33	Q5VTE0	EF1A3	HUMAN	*EF1A3*	12.60	14.03	14.69	11.79	10.08	13.02	12.14	12.56	11.82	17.75	15.82	16.64	14.93	12.29	15.72	12.71	14.09	13.99	5.41E+03	5.38E+04	1.17E+05	1.02E+03	2.53E+02	2.25E+03	1.29E+03	1.62E+03	6.73E+02	5.13E+05	4.03E+04	1.96E+05	9.09E+04	8.14E+03	5.23E+05	4.36E+03	2.32E+04	5.32E+04
34	P60174	TPIS	HUMAN	*TPI1*	14.22	14.85	14.42	13.75	14.15	14.18	14.67	13.44	13.40	14.07	14.64	13.13	15.02	13.48	14.36	13.31	13.33	15.64	1.64E+04	9.57E+04	9.71E+04	3.86E+03	3.84E+03	4.99E+03	7.26E+03	2.92E+03	1.97E+03	3.97E+04	1.78E+04	1.62E+04	9.70E+04	1.86E+04	2.00E+05	6.57E+03	1.38E+04	1.68E+05
35	P04406	G3P	HUMAN	*GAPDH*	14.29	14.29	17.06	14.70	13.55	13.11	15.26	13.84	13.55	18.49	15.34	17.26	16.61	15.17	16.57	14.71	15.15	15.65	1.73E+04	6.45E+04	6.11E+05	7.42E+03	2.54E+03	2.39E+03	1.09E+04	3.86E+03	2.17E+03	8.57E+05	2.89E+04	3.00E+05	2.93E+05	6.00E+04	9.45E+05	1.72E+04	4.84E+04	1.68E+05
36	P38646	GRP75	HUMAN	*HSPA9*	13.08	15.53	16.00	13.31	12.14	12.76	12.71	13.00	11.79	15.67	13.03	13.91	13.88	14.12	15.36	13.17	13.80	14.33	7.50E+03	1.53E+05	2.91E+05	2.85E+03	9.76E+02	1.89E+03	1.89E+03	2.17E+03	6.60E+02	1.21E+05	5.88E+03	2.87E+04	4.38E+04	2.90E+04	4.05E+05	5.95E+03	1.91E+04	6.74E+04
37	P55072	TERA	HUMAN	*VCP*	14.63	15.47	14.86	13.12	12.22	11.60	12.40	13.21	11.69	15.52	13.98	14.04	14.01	14.67	14.82	13.33	13.33	15.83	2.17E+04	1.46E+05	1.32E+05	2.50E+03	1.02E+03	8.69E+02	1.54E+03	2.50E+03	6.18E+02	1.09E+05	1.13E+04	3.13E+04	4.80E+04	4.24E+04	2.77E+05	6.66E+03	1.38E+04	1.91E+05
38	P61978	HNRPK	HUMAN	*HNRNPK*	11.65	12.82	11.29	13.27	12.04	12.09	12.69	11.21	12.27	14.60	11.82	13.64	13.46	13.96	11.43	12.50	11.39	11.69	2.87E+03	2.31E+04	1.01E+04	2.78E+03	9.12E+02	1.20E+03	1.87E+03	6.57E+02	9.08E+02	5.76E+04	2.60E+03	2.35E+04	3.26E+04	2.60E+04	2.23E+04	3.79E+03	3.67E+03	1.03E+04
39	P19338	NUCL	HUMAN	*NCL*	13.36	14.40	12.89	14.06	12.88	12.18	14.52	12.60	11.14	12.92	13.71	14.08	12.47	13.62	13.42	14.53	13.59	12.81	9.11E+03	6.99E+04	3.29E+04	4.79E+03	1.60E+03	1.27E+03	6.54E+03	1.66E+03	4.30E+02	1.77E+04	9.42E+03	3.22E+04	1.61E+04	2.06E+04	1.02E+05	1.52E+04	1.65E+04	2.31E+04
40	P06576	ATPB	HUMAN	*ATP5F1B*	13.16	15.04	15.26	12.03	12.17	11.56	10.94	11.08	11.84	14.03	12.08	13.66	13.61	12.67	12.84	13.23	12.69	12.53	7.92E+03	1.09E+05	1.74E+05	1.19E+03	9.92E+02	8.45E+02	5.83E+02	6.04E+02	6.83E+02	3.85E+04	3.10E+03	2.38E+04	3.61E+04	1.06E+04	6.73E+04	6.20E+03	8.86E+03	1.90E+04
41	Q86VP6	CAND1	HUMAN	*CAND1*	13.53	13.78	12.44	12.80	12.95	12.87	13.71	11.98	13.49	11.10	13.35	12.37	13.47	13.47	10.58	13.03	12.61	13.50	1.02E+04	4.52E+04	2.37E+04	2.02E+03	1.68E+03	2.04E+03	3.74E+03	1.09E+03	2.09E+03	4.72E+03	7.33E+03	9.16E+03	3.27E+04	1.85E+04	1.02E+04	5.41E+03	8.43E+03	3.75E+04
42	Q14204	DYHC1	HUMAN	*DYNC1H1*	13.00	14.19	12.92	12.83	13.76	13.61	12.61	13.22	13.23	11.73	13.02	12.27	13.44	14.67	12.90	13.00	12.62	12.08	7.13E+03	6.02E+04	3.36E+04	2.06E+03	2.93E+03	3.38E+03	1.77E+03	2.52E+03	1.75E+03	7.55E+03	5.85E+03	8.41E+03	3.22E+04	4.23E+04	6.98E+04	5.30E+03	8.46E+03	1.37E+04
43	P63104	1433Z	HUMAN	*YWHAZ*	13.51	13.85	13.77	13.78	12.09	12.85	12.87	13.18	12.12	12.70	12.61	12.95	13.95	14.08	12.18	13.26	13.53	13.78	1.01E+04	4.76E+04	6.13E+04	3.94E+03	9.38E+02	2.00E+03	2.12E+03	2.46E+03	8.24E+02	1.51E+04	4.45E+03	1.41E+04	4.58E+04	2.81E+04	4.08E+04	6.33E+03	1.58E+04	4.57E+04
44	P04264	K2C1	HUMAN	*KRT1*	12.04	10.57	10.92	11.65	12.37	12.44	11.20	11.93	8.67	11.98	13.49	12.27	13.32	11.56	10.45	12.19	11.46	11.32	3.71E+03	4.56E+03	7.57E+03	9.26E+02	1.14E+03	1.52E+03	6.88E+02	1.06E+03	9.68E+01	9.05E+03	8.09E+03	8.42E+03	2.94E+04	4.92E+03	8.98E+03	3.06E+03	3.86E+03	7.89E+03
45	P07237	PDIA1	HUMAN	*P4HB*	12.55	11.62	12.16	12.28	11.35	12.49	12.53	12.77	12.65	13.55	12.01	11.67	12.17	12.44	11.45	12.79	12.42	12.37	5.23E+03	9.83E+03	1.94E+04	1.42E+03	5.74E+02	1.57E+03	1.68E+03	1.85E+03	1.17E+03	2.75E+04	2.96E+03	5.21E+03	1.29E+04	9.04E+03	2.27E+04	4.60E+03	7.37E+03	1.69E+04
46	P02545	LMNA	HUMAN	*LMNA*	13.93	13.41	14.27	12.03	11.95	11.45	14.65	12.34	13.28	14.11	13.28	14.25	12.06	11.37	13.63	14.64	12.13	12.66	1.34E+04	3.50E+04	8.72E+04	1.19E+03	8.57E+02	7.84E+02	7.14E+03	1.39E+03	NA	4.09E+04	6.99E+03	3.64E+04	1.20E+04	4.33E+03	1.20E+05	1.63E+04	6.07E+03	2.08E+04
47	P14625	ENPL	HUMAN	*HSP90B1*	14.40	14.20	12.71	12.68	12.40	12.63	12.93	13.19	11.47	16.39	15.04	12.80	13.74	13.77	13.01	14.51	13.01	15.01	1.86E+04	6.07E+04	2.89E+04	1.86E+03	1.16E+03	1.73E+03	2.19E+03	2.46E+03	5.35E+02	1.99E+05	2.35E+04	1.27E+04	3.96E+04	2.27E+04	7.61E+04	1.49E+04	1.11E+04	1.08E+05
48	P07195	LDHB	HUMAN	*LDHB*	13.98	11.27	11.58	12.79	13.01	12.54	12.84	12.95	12.68	12.31	13.36	12.93	13.71	14.66	11.28	12.58	12.75	11.86	1.39E+04	7.67E+03	1.26E+04	2.01E+03	1.76E+03	1.62E+03	2.07E+03	2.10E+03	1.20E+03	1.14E+04	7.41E+03	1.40E+04	3.89E+04	4.20E+04	1.96E+04	3.98E+03	9.27E+03	1.17E+04
49	P36578	RL4	HUMAN	*RPL4*	13.05	14.65	14.61	14.01	12.67	14.45	13.86	13.67	12.62	14.47	15.17	14.61	14.88	14.19	15.27	14.26	14.77	13.00	7.34E+03	8.29E+04	1.11E+05	4.63E+03	1.40E+03	6.02E+03	4.17E+03	3.43E+03	1.16E+03	5.25E+04	2.57E+04	4.72E+04	8.81E+04	3.05E+04	3.80E+05	1.26E+04	3.72E+04	2.64E+04
50	P53396	ACLY	HUMAN	*ACLY*	12.93	10.76	11.19	10.97	12.50	11.93	11.82	12.24	12.18	11.17	11.41	10.95	10.57	11.37	12.32	11.82	11.83	11.39	6.79E+03	5.26E+03	9.34E+03	5.94E+02	1.24E+03	1.08E+03	1.04E+03	1.30E+03	8.55E+02	4.96E+03	1.99E+03	2.75E+03	3.91E+03	4.31E+03	4.54E+04	2.39E+03	4.96E+03	8.31E+03
51	P62805	H4	HUMAN	*H4C1*	12.80	15.84	15.96	12.83	12.59	11.96	12.50	12.03	10.86	18.40	14.71	15.12	14.44	13.30	14.14	13.27	14.29	16.86	6.21E+03	1.89E+05	2.84E+05	2.05E+03	1.32E+03	1.10E+03	1.64E+03	1.13E+03	3.56E+02	8.04E+05	1.87E+04	6.76E+04	6.47E+04	1.64E+04	1.72E+05	6.37E+03	2.67E+04	3.92E+05
52	Q92616	GCN1	HUMAN	*GCN1*	11.95	12.94	11.28	12.54	13.16	12.27	12.48	12.17	13.33	11.05	12.73	12.34	13.30	12.47	11.04	12.44	12.08	11.39	3.49E+03	2.51E+04	1.01E+04	1.69E+03	1.94E+03	1.36E+03	1.62E+03	1.24E+03	1.87E+03	4.56E+03	4.81E+03	8.91E+03	2.91E+04	9.26E+03	1.59E+04	3.63E+03	5.84E+03	8.32E+03
53	P05141	ADT2	HUMAN	*SLC25A5*	12.09	13.29	14.99	12.94	11.60	15.53	12.71	13.23	14.41	10.59	14.04	11.59	14.16	13.54	14.31	13.66	12.84	14.47	3.85E+03	3.21E+04	1.44E+05	2.21E+03	6.79E+02	1.27E+04	1.89E+03	2.54E+03	3.95E+03	3.20E+03	1.18E+04	4.85E+03	5.33E+04	1.95E+04	1.94E+05	8.31E+03	9.83E+03	7.44E+04
54	P0DMV9	HS71B	HUMAN	*HSPA1B*	13.81	13.34	13.23	13.15	13.59	13.80	13.29	13.07	12.65	13.30	14.17	12.73	12.47	13.74	12.46	13.92	14.00	12.67	1.24E+04	3.34E+04	4.20E+04	2.55E+03	2.61E+03	3.84E+03	2.82E+03	2.27E+03	1.18E+03	2.31E+04	1.29E+04	1.20E+04	1.61E+04	2.23E+04	5.05E+04	9.94E+03	2.19E+04	2.09E+04
55	O00571	DDX3X	HUMAN	*DDX3X*	13.77	14.05	12.78	13.04	14.97	13.14	14.20	12.47	13.29	13.61	14.29	13.72	13.86	13.43	11.95	13.95	14.26	12.51	1.20E+04	5.48E+04	3.04E+04	2.38E+03	6.76E+03	2.44E+03	5.24E+03	1.52E+03	1.82E+03	2.87E+04	1.40E+04	2.49E+04	4.32E+04	1.80E+04	3.41E+04	1.01E+04	2.61E+04	1.87E+04
56	P40227	TCPZ	HUMAN	*CCT6A*	13.56	11.61	10.84	12.12	9.30	14.07	12.63	12.53	12.32	11.25	12.79	10.89	12.03	12.78	13.59	11.83	13.54	12.84	1.04E+04	9.80E+03	7.09E+03	1.27E+03	1.59E+02	4.62E+03	1.80E+03	1.58E+03	9.44E+02	5.30E+03	5.02E+03	2.59E+03	1.17E+04	1.15E+04	1.16E+05	2.40E+03	1.59E+04	2.37E+04
57	P60842	IF4A1	HUMAN	*EIF4A1*	12.71	13.84	14.61	13.13	12.13	12.73	13.26	13.28	12.03	12.05	14.19	14.80	12.37	13.94	15.20	14.24	14.78	14.28	5.83E+03	4.72E+04	1.11E+05	2.53E+03	9.69E+02	1.84E+03	2.75E+03	2.62E+03	7.76E+02	9.52E+03	1.30E+04	5.37E+04	1.50E+04	2.56E+04	3.64E+05	1.24E+04	3.74E+04	6.50E+04
58	P15311	EZRI	HUMAN	*EZR*	12.24	13.58	13.74	13.13	12.65	12.47	13.01	12.80	11.76	13.75	12.61	13.60	11.31	13.29	14.47	12.39	14.24	13.31	4.25E+03	3.95E+04	6.00E+04	2.52E+03	1.37E+03	1.55E+03	2.32E+03	1.89E+03	6.46E+02	3.16E+04	4.45E+03	2.28E+04	6.92E+03	1.63E+04	2.18E+05	3.52E+03	2.58E+04	3.31E+04
59	P78371	TCPB	HUMAN	*CCT2*	12.95	13.94	13.64	11.68	14.08	12.52	12.48	13.03	12.78	10.62	12.23	12.77	10.86	13.22	13.26	12.07	11.70	13.12	6.85E+03	5.08E+04	5.61E+04	9.49E+02	3.64E+03	1.60E+03	1.62E+03	2.22E+03	1.29E+03	3.26E+03	3.42E+03	1.24E+04	4.91E+03	1.56E+04	9.15E+04	2.83E+03	4.53E+03	2.88E+04
60	Q06830	PRDX1	HUMAN	*PRDX1*	12.79	14.77	13.66	12.23	11.19	12.73	12.61	12.38	14.52	17.24	14.69	15.12	15.02	12.05	15.68	12.49	14.48	15.38	6.16E+03	9.00E+04	5.68E+04	1.37E+03	5.17E+02	1.84E+03	1.76E+03	1.43E+03	4.24E+03	3.60E+05	1.84E+04	6.75E+04	9.69E+04	6.89E+03	5.08E+05	3.77E+03	3.04E+04	1.40E+05
61	P46940	IQGA1	HUMAN	*IQGAP1*	11.12	9.72	10.04	11.02	11.97	11.82	10.62	11.43	12.07	9.55	11.43	10.39	11.90	10.20	9.87	12.67	9.49	10.32	2.02E+03	2.37E+03	3.61E+03	6.12E+02	8.69E+02	1.00E+03	4.73E+02	7.59E+02	7.97E+02	1.35E+03	2.02E+03	1.52E+03	1.07E+04	1.91E+03	4.48E+03	4.25E+03	1.07E+03	3.71E+03
62	P11021	BIP	HUMAN	*HSPA5*	12.25	13.08	12.14	13.37	12.93	12.09	13.02	12.31	11.70	14.33	13.48	13.77	11.07	12.76	11.84	12.81	12.82	12.34	4.28E+03	2.78E+04	1.91E+04	2.98E+03	1.66E+03	1.20E+03	2.34E+03	1.36E+03	6.23E+02	4.77E+04	8.00E+03	2.58E+04	5.76E+03	1.13E+04	3.12E+04	4.66E+03	9.70E+03	1.66E+04
63	P04075	ALDOA	HUMAN	*ALDOA*	13.42	13.19	14.72	14.03	10.91	12.74	14.65	12.52	10.37	15.16	13.18	15.18	12.73	13.67	14.92	13.69	13.49	15.39	9.44E+03	3.00E+04	1.20E+05	4.67E+03	4.30E+02	1.86E+03	7.19E+03	1.57E+03	2.61E+02	8.51E+04	6.53E+03	7.06E+04	1.94E+04	2.12E+04	2.98E+05	8.53E+03	1.53E+04	1.41E+05
64	P30838	AL3A1	HUMAN	*ALDH3A1*	13.28	15.61	13.88	13.66	14.53	12.63	13.74	14.94	11.47	13.91	13.90	13.40	14.56	13.42	14.62	13.72	16.51	15.21	8.62E+03	1.62E+05	6.65E+04	3.64E+03	4.99E+03	1.73E+03	3.82E+03	8.20E+03	5.32E+02	3.56E+04	1.07E+04	1.98E+04	7.06E+04	1.78E+04	2.41E+05	8.67E+03	1.24E+05	1.24E+05
65	P52895	AK1C2	HUMAN	*AKR1C2*	12.86	11.52	13.25	11.66	12.70	11.34	12.70	10.60	12.70	13.44	14.39	12.77	12.92	12.70	11.92	12.98	12.49	11.54	6.45E+03	9.14E+03	4.26E+04	9.34E+02	NA	7.29E+02	NA	4.46E+02	NA	2.55E+04	1.50E+04	1.24E+04	2.22E+04	1.08E+04	3.31E+04	5.22E+03	7.73E+03	9.31E+03
66	Q08211	DHX9	HUMAN	*DHX9*	10.12	8.06	8.69	9.49	10.26	10.40	10.47	11.59	11.69	9.13	9.46	9.49	10.28	10.60	9.11	8.77	8.78	8.52	1.07E+03	5.02E+02	7.46E+02	NA	2.85E+02	4.00E+02	4.30E+02	8.42E+02	6.15E+02	9.10E+02	5.86E+02	3.24E+02	3.11E+03	2.54E+03	7.27E+02	3.72E+02	7.02E+02	7.01E+02
67	P30153	2AAA	HUMAN	*PPP2R1A*	11.49	9.83	10.00	12.78	12.14	11.09	10.85	12.64	12.03	10.07	11.38	11.19	10.56	12.66	10.68	11.47	12.12	10.54	2.58E+03	2.58E+03	3.46E+03	1.98E+03	9.75E+02	6.20E+02	5.50E+02	1.70E+03	7.76E+02	2.12E+03	1.95E+03	3.44E+03	3.87E+03	1.05E+04	1.14E+04	1.89E+03	6.01E+03	4.40E+03
68	Q9Y490	TLN1	HUMAN	*TLN1*	10.55	12.43	12.49	10.82	10.38	9.53	9.73	9.92	10.38	8.95	10.24	10.07	10.53	11.32	10.58	10.23	8.19	10.62	1.41E+03	1.75E+04	2.46E+04	5.38E+02	NA	2.37E+02	2.72E+02	2.94E+02	NA	7.63E+02	9.36E+02	1.01E+03	3.78E+03	4.16E+03	1.03E+04	8.60E+02	5.09E+02	4.69E+03
69	P40926	MDHM	HUMAN	*MDH2*	12.64	14.62	15.04	12.49	12.82	12.88	11.91	12.45	10.40	12.87	12.68	11.48	11.98	14.36	11.21	12.67	13.06	13.49	5.57E+03	8.12E+04	1.49E+05	1.63E+03	1.54E+03	2.05E+03	1.11E+03	1.50E+03	2.66E+02	1.70E+04	4.66E+03	4.43E+03	1.13E+04	3.43E+04	1.85E+04	4.24E+03	1.14E+04	3.73E+04
70	P08195	4F2	HUMAN	*SLC3A2*	12.97	15.04	11.72	12.41	12.52	14.37	11.24	11.56	12.66	12.47	11.95	11.44	13.80	14.53	13.92	13.20	14.21	13.64	6.96E+03	1.09E+05	1.40E+04	1.55E+03	1.26E+03	5.70E+03	7.07E+02	8.27E+02	1.19E+03	1.28E+04	2.84E+03	4.30E+03	4.13E+04	3.85E+04	1.47E+05	6.08E+03	2.52E+04	4.16E+04
71	P26641	EF1G	HUMAN	*EEF1G*	11.30	11.97	10.95	12.20	10.93	13.51	13.67	12.14	12.40	15.45	11.94	13.66	13.73	13.43	14.77	11.95	12.79	15.19	2.28E+03	1.26E+04	7.73E+03	1.34E+03	4.36E+02	3.15E+03	3.64E+03	1.21E+03	9.96E+02	1.04E+05	2.82E+03	2.39E+04	3.92E+04	1.80E+04	2.69E+05	2.61E+03	9.49E+03	1.23E+05
72	P11940	PABP1	HUMAN	*PABPC1*	13.12	11.99	12.33	14.44	14.33	11.85	13.36	12.78	12.11	10.66	14.33	13.77	12.98	13.16	11.50	10.81	14.04	11.47	7.73E+03	1.28E+04	2.20E+04	6.22E+03	4.35E+03	1.03E+03	2.94E+03	1.87E+03	8.17E+02	3.38E+03	1.43E+04	2.57E+04	2.32E+04	1.49E+04	2.36E+04	1.24E+03	2.25E+04	8.81E+03
73	P55060	XPO2	HUMAN	*CSE1L*	13.28	11.21	12.31	13.17	14.21	12.72	13.75	13.07	13.81	10.36	13.46	11.29	13.72	13.38	10.07	14.25	12.72	11.37	8.58E+03	7.29E+03	2.17E+04	2.60E+03	4.01E+03	1.84E+03	3.85E+03	2.28E+03	2.61E+03	2.66E+03	7.91E+03	3.74E+03	3.92E+04	1.73E+04	5.85E+03	1.25E+04	9.07E+03	8.18E+03
74	P26599	PTBP1	HUMAN	*PTBP1*	11.18	13.73	11.65	11.72	12.45	11.71	14.82	11.89	13.39	10.77	10.98	11.18	12.78	11.68	12.90	11.35	12.62	10.15	2.10E+03	4.36E+04	1.32E+04	9.71E+02	1.20E+03	9.31E+02	8.07E+03	1.03E+03	1.95E+03	3.68E+03	1.50E+03	3.39E+03	2.01E+04	5.33E+03	7.03E+04	1.75E+03	8.46E+03	3.22E+03
75	P00558	PGK1	HUMAN	*PGK1*	13.76	15.57	13.45	12.37	12.87	12.68	12.59	14.00	12.62	13.09	13.30	12.55	14.68	11.15	11.86	11.99	13.45	14.75	1.20E+04	1.58E+05	4.89E+04	1.51E+03	1.59E+03	1.79E+03	1.74E+03	4.29E+03	1.16E+03	2.00E+04	7.09E+03	1.05E+04	7.65E+04	3.71E+03	3.17E+04	2.67E+03	1.50E+04	9.02E+04
76	O60506	HNRPQ	HUMAN	*SYNCRIP*	10.47	12.11	12.23	12.32	11.84	10.44	10.24	11.40	9.78	9.16	9.75	10.42	8.66	10.45	9.93	9.80	10.13	12.80	1.33E+03	1.39E+04	2.05E+04	1.45E+03	7.97E+02	4.11E+02	3.72E+02	7.43E+02	1.81E+02	9.40E+02	6.96E+02	1.58E+03	6.32E+02	2.29E+03	4.87E+03	6.61E+02	1.61E+03	2.29E+04
77	Q14974	IMB1	HUMAN	*KPNB1*	13.63	15.78	15.02	11.85	12.05	11.76	10.32	12.17	11.67	13.75	13.47	13.83	14.22	14.19	13.76	12.60	13.63	14.63	1.09E+04	1.82E+05	1.47E+05	1.06E+03	9.16E+02	9.65E+02	3.92E+02	1.24E+03	6.09E+02	3.16E+04	7.97E+03	2.69E+04	5.56E+04	3.03E+04	1.31E+05	4.04E+03	1.69E+04	8.28E+04
78	Q9NR30	DDX21	HUMAN	*DDX21*	10.10	10.26	12.49	10.35	10.23	11.76	9.52	12.35	10.90	9.89	12.21	12.89	10.90	10.17	13.13	10.29	11.38	11.23	1.06E+03	3.60E+03	2.46E+04	4.00E+02	2.78E+02	9.65E+02	2.41E+02	1.40E+03	NA	1.83E+03	3.39E+03	1.35E+04	5.07E+03	1.89E+03	8.33E+04	8.89E+02	3.66E+03	7.43E+03
79	P22392	NDKB	HUMAN	*NME2*	13.64	11.96	11.67	11.44	11.72	11.46	11.41	10.71	10.39	11.48	11.73	11.33	12.14	13.75	13.08	12.86	11.11	12.62	1.10E+04	1.25E+04	1.35E+04	8.08E+02	7.35E+02	7.89E+02	7.90E+02	4.76E+02	2.64E+02	6.27E+03	2.45E+03	3.89E+03	1.27E+04	2.24E+04	7.99E+04	4.82E+03	3.04E+03	2.02E+04
80	P62987	RL40	HUMAN	*UBA52*	12.21	12.68	15.16	13.05	9.90	12.58	12.24	12.32	10.03	14.04	14.29	16.25	14.55	13.21	13.23	12.31	13.75	12.80	4.16E+03	2.09E+04	1.63E+05	2.38E+03	2.27E+02	1.67E+03	1.37E+03	1.37E+03	2.11E+02	3.89E+04	1.40E+04	1.49E+05	7.00E+04	1.54E+04	8.93E+04	3.32E+03	1.84E+04	2.29E+04
81	P07737	PROF1	HUMAN	*PFN1*	11.72	10.93	14.64	13.39	12.30	13.20	12.98	12.76	13.26	14.27	12.19	13.79	13.45	13.48	13.38	12.59	12.28	14.97	3.00E+03	5.95E+03	1.13E+05	3.02E+03	1.08E+03	2.55E+03	2.27E+03	1.84E+03	1.79E+03	4.57E+04	3.35E+03	2.62E+04	3.23E+04	1.87E+04	9.99E+04	4.02E+03	6.69E+03	1.05E+05
82	P05388	RLA0	HUMAN	*RPLP0*	11.79	12.94	11.67	12.42	11.70	12.30	12.20	13.17	12.62	12.65	12.83	12.81	13.27	12.08	11.18	12.96	12.52	11.52	3.15E+03	2.51E+04	1.35E+04	1.56E+03	7.22E+02	1.39E+03	1.34E+03	2.44E+03	1.15E+03	1.46E+04	5.13E+03	1.28E+04	2.84E+04	7.05E+03	1.80E+04	5.16E+03	7.92E+03	9.16E+03
83	Q6FI13	H2A2A	HUMAN	*H2AC19*	12.22	14.06	14.09	12.93	11.68	11.50	11.18	11.54	11.11	13.51	11.71	15.12	13.68	14.34	13.74	11.76	13.13	13.96	4.18E+03	5.51E+04	7.71E+04	2.20E+03	7.14E+02	8.11E+02	6.81E+02	8.16E+02	4.20E+02	2.68E+04	2.42E+03	6.74E+04	3.81E+04	3.37E+04	1.29E+05	2.29E+03	1.20E+04	5.21E+04
84	Q99623	PHB2	HUMAN	*PHB2*	11.28	10.96	11.33	11.88	11.59	10.40	10.35	10.82	11.65	11.59	12.71	12.05	11.43	11.74	11.61	12.18	12.65	10.57	2.25E+03	6.08E+03	1.04E+04	1.08E+03	NA	4.00E+02	4.00E+02	5.11E+02	6.01E+02	6.80E+03	4.75E+03	7.09E+03	7.55E+03	5.58E+03	2.58E+04	3.05E+03	8.66E+03	4.51E+03
85	P09211	GSTP1	HUMAN	*GSTP1*	9.41	9.70	10.70	11.65	10.53	11.18	11.62	10.76	10.76	14.01	10.07	9.90	13.56	12.47	11.53	10.34	11.71	9.06	7.03E+02	2.33E+03	6.33E+03	9.26E+02	3.36E+02	6.56E+02	9.09E+02	4.93E+02	NA	3.81E+04	8.46E+02	7.75E+02	3.50E+04	9.27E+03	2.42E+04	9.18E+02	4.57E+03	1.25E+03
86	P23528	COF1	HUMAN	*CFL1*	12.92	12.42	12.78	11.60	10.42	12.35	13.33	12.45	10.49	15.57	13.31	11.23	11.62	11.80	14.24	13.24	12.61	14.61	6.76E+03	1.74E+04	3.03E+04	9.00E+02	3.14E+02	1.43E+03	2.90E+03	1.50E+03	2.82E+02	1.13E+05	7.14E+03	3.57E+03	8.72E+03	5.82E+03	1.84E+05	6.25E+03	8.39E+03	8.16E+04
87	P06744	G6PI	HUMAN	*GPI*	12.98	11.82	15.09	12.11	12.03	11.48	11.67	12.01	12.64	12.50	13.14	14.16	12.08	13.40	14.33	13.03	12.59	12.98	7.03E+03	1.14E+04	1.55E+05	1.26E+03	9.04E+02	8.03E+02	9.41E+02	1.11E+03	1.17E+03	1.31E+04	6.38E+03	3.43E+04	1.22E+04	1.76E+04	1.96E+05	5.43E+03	8.28E+03	2.61E+04
88	Q92598	HS105	HUMAN	*HSPH1*	13.64	12.91	12.34	12.17	12.95	13.09	14.07	11.80	11.35	12.62	13.60	13.36	11.96	11.51	11.63	11.51	12.72	12.82	1.11E+04	2.45E+04	2.21E+04	1.31E+03	1.68E+03	2.37E+03	4.79E+03	9.68E+02	4.91E+02	1.43E+04	8.72E+03	1.91E+04	1.11E+04	4.77E+03	2.63E+04	1.95E+03	9.08E+03	2.33E+04
89	Q13813	SPTN1	HUMAN	*SPTAN1*	10.32	12.75	11.13	11.88	10.86	10.49	10.42	10.29	12.18	9.38	10.38	10.98	11.92	11.12	9.62	10.89	10.72	10.72	1.21E+03	2.20E+04	8.88E+03	1.09E+03	4.17E+02	4.23E+02	4.17E+02	3.66E+02	8.60E+02	1.16E+03	1.02E+03	2.82E+03	1.09E+04	3.63E+03	3.03E+03	1.30E+03	2.36E+03	5.03E+03
90	P22314	UBA1	HUMAN	*UBA1*	9.93	9.22	9.47	10.75	12.84	12.30	12.11	12.18	12.13	10.30	11.31	11.05	10.11	11.58	9.68	9.58	9.04	8.59	9.56E+02	1.57E+03	2.06E+03	5.15E+02	1.56E+03	1.39E+03	1.26E+03	1.24E+03	8.29E+02	2.54E+03	1.86E+03	3.01E+03	2.70E+03	4.97E+03	3.37E+03	5.81E+02	8.19E+02	7.57E+02
91	P15559	NQO1	HUMAN	*NQO1*	11.63	10.60	12.78	11.01	11.95	11.44	12.26	10.75	11.69	14.77	10.71	11.78	11.71	11.55	13.34	10.93	13.39	12.01	2.84E+03	4.67E+03	3.04E+04	6.10E+02	8.55E+02	7.79E+02	1.39E+03	4.88E+02	6.15E+02	6.48E+04	1.26E+03	5.69E+03	9.26E+03	4.88E+03	9.68E+04	1.33E+03	1.43E+04	1.31E+04
92	P22626	ROA2	HUMAN	*HNRNPA2B1*	12.19	12.04	12.77	11.78	10.68	12.03	12.92	12.33	10.82	16.42	12.50	12.61	14.24	11.85	11.94	12.53	13.38	13.94	4.11E+03	1.33E+04	3.01E+04	1.01E+03	3.70E+02	1.15E+03	2.18E+03	1.38E+03	3.47E+02	2.04E+05	4.12E+03	1.10E+04	5.63E+04	6.00E+03	3.37E+04	3.85E+03	1.42E+04	5.14E+04
93	P50991	TCPD	HUMAN	*CCT4*	12.04	12.95	13.45	13.52	11.53	12.07	14.78	12.46	11.76	10.70	12.44	11.59	13.10	11.72	10.56	11.87	11.31	11.59	3.72E+03	2.53E+04	4.91E+04	3.30E+03	6.46E+02	1.18E+03	7.85E+03	1.51E+03	6.45E+02	3.47E+03	3.95E+03	4.87E+03	2.52E+04	5.50E+03	1.01E+04	2.48E+03	3.49E+03	9.66E+03
94	P23526	SAHH	HUMAN	*AHCY*	13.26	14.37	11.94	13.11	12.42	13.60	11.19	12.92	13.05	14.43	12.87	13.73	11.07	13.32	13.20	14.10	14.37	13.53	8.48E+03	6.85E+04	1.64E+04	2.49E+03	1.18E+03	3.35E+03	6.87E+02	2.06E+03	1.55E+03	5.11E+04	5.28E+03	2.51E+04	5.77E+03	1.67E+04	8.72E+04	1.13E+04	2.81E+04	3.85E+04
95	Q00839	HNRPU	HUMAN	*HNRNPU*	11.88	11.95	13.48	10.84	11.53	11.36	11.82	11.02	13.72	14.23	12.88	12.45	11.90	10.88	13.79	11.23	12.04	11.64	3.35E+03	1.24E+04	4.99E+04	5.44E+02	6.45E+02	7.41E+02	1.04E+03	5.81E+02	2.44E+03	4.42E+04	5.33E+03	9.70E+03	1.07E+04	3.08E+03	1.34E+05	1.62E+03	5.71E+03	1.00E+04
96	P49411	EFTU	HUMAN	*TUFM*	10.30	12.66	13.54	10.58	10.04	10.40	10.93	10.54	12.39	9.33	13.18	10.58	11.05	10.13	11.64	9.16	10.68	9.46	1.20E+03	2.07E+04	5.21E+04	NA	2.47E+02	4.00E+02	5.78E+02	4.30E+02	9.86E+02	1.11E+03	6.53E+03	1.88E+03	5.67E+03	1.83E+03	2.65E+04	4.59E+02	2.29E+03	1.80E+03
97	P07910	HNRPC	HUMAN	*HNRNPC*	10.45	10.50	10.94	11.50	11.09	9.45	10.42	10.02	11.01	10.46	10.50	10.45	11.09	11.79	12.25	12.06	12.77	11.33	1.32E+03	4.33E+03	7.67E+03	8.38E+02	4.82E+02	2.27E+02	4.18E+02	3.13E+02	3.95E+02	2.88E+03	1.10E+03	1.62E+03	5.84E+03	5.78E+03	4.31E+04	2.81E+03	9.40E+03	7.98E+03
98	P17844	DDX5	HUMAN	*DDX5*	10.50	13.43	14.22	11.07	12.31	10.78	10.97	11.11	10.81	12.40	14.38	12.62	14.16	12.12	10.34	10.86	12.10	13.57	1.36E+03	3.54E+04	8.44E+04	6.35E+02	1.09E+03	5.07E+02	5.94E+02	6.16E+02	3.46E+02	1.22E+04	1.49E+04	1.10E+04	5.30E+04	7.26E+03	8.00E+03	1.28E+03	5.94E+03	3.96E+04
99	P04843	RPN1	HUMAN	*RPN1*	11.00	9.72	11.19	10.18	10.23	9.44	11.03	11.00	11.03	9.38	11.44	10.80	11.33	10.14	10.10	11.53	9.93	9.77	1.87E+03	2.38E+03	9.37E+03	3.58E+02	2.78E+02	2.26E+02	6.15E+02	5.73E+02	4.00E+02	1.16E+03	2.03E+03	2.37E+03	6.99E+03	1.84E+03	6.10E+03	1.98E+03	1.41E+03	2.37E+03
100	Q15084	PDIA6	HUMAN	*PDIA6*	13.70	9.73	9.39	11.91	12.67	11.32	12.23	11.73	11.50	10.55	12.99	10.63	11.19	11.06	11.68	11.09	10.71	12.78	1.15E+04	2.40E+03	1.90E+03	1.10E+03	1.39E+03	7.22E+02	1.36E+03	9.25E+02	5.43E+02	3.09E+03	5.75E+03	1.98E+03	6.30E+03	3.48E+03	2.74E+04	1.48E+03	2.33E+03	2.27E+04
101	P13797	PLST	HUMAN	*PLS3*	11.18	10.74	9.20	11.50	11.90	12.17	11.48	13.09	11.48	10.97	11.51	11.30	11.30	12.08	9.46	12.17	11.30	11.59	2.11E+03	5.20E+03	1.51E+03	8.42E+02	8.26E+02	1.27E+03	8.28E+02	2.31E+03	NA	4.28E+03	2.12E+03	3.80E+03	6.84E+03	7.07E+03	2.23E+03	3.02E+03	3.45E+03	9.65E+03
102	P05556	ITB1	HUMAN	*ITGB1*	9.13	10.83	9.58	9.80	9.76	10.40	10.47	11.18	9.76	9.96	9.71	12.10	9.22	9.35	11.25	8.16	9.21	8.67	6.01E+02	5.53E+03	2.32E+03	2.84E+02	NA	4.00E+02	4.30E+02	6.45E+02	NA	1.93E+03	6.81E+02	7.37E+03	1.20E+03	1.06E+03	1.91E+04	2.77E+02	9.03E+02	8.37E+02
103	P50990	TCPQ	HUMAN	*CCT8*	11.11	11.77	10.63	12.37	11.66	11.93	11.80	11.19	11.66	14.00	11.32	11.30	11.53	11.35	13.40	12.16	11.29	11.97	2.01E+03	1.10E+04	5.97E+03	1.51E+03	7.07E+02	1.08E+03	1.03E+03	6.49E+02	6.06E+02	3.77E+04	1.88E+03	3.78E+03	8.13E+03	4.26E+03	1.01E+05	3.00E+03	3.44E+03	1.27E+04
104	P18206	VINC	HUMAN	*VCL*	13.17	13.04	13.11	11.38	11.62	12.42	12.52	12.31	11.97	10.22	11.13	10.70	11.20	12.56	12.08	11.84	11.37	13.15	7.96E+03	2.69E+04	3.84E+04	7.75E+02	6.87E+02	1.50E+03	1.67E+03	1.36E+03	7.43E+02	2.39E+03	1.65E+03	2.14E+03	6.36E+03	9.82E+03	3.78E+04	2.43E+03	3.63E+03	2.93E+04
105	Q14152	EIF3A	HUMAN	*EIF3A*	10.72	9.70	9.48	10.94	12.06	11.04	12.59	11.58	11.63	9.65	11.75	10.62	10.24	10.45	10.84	11.48	11.60	10.93	1.57E+03	2.34E+03	2.09E+03	5.81E+02	9.19E+02	6.02E+02	1.75E+03	8.39E+02	5.94E+02	1.48E+03	2.49E+03	1.96E+03	3.01E+03	2.28E+03	1.32E+04	1.91E+03	4.24E+03	5.93E+03
106	P52292	IMA1	HUMAN	*KPNA2*	13.08	13.56	14.03	12.29	12.20	12.57	12.49	12.86	12.80	13.25	11.68	13.85	13.95	12.79	13.74	11.78	11.93	13.11	7.53E+03	3.88E+04	7.40E+04	1.42E+03	1.01E+03	1.66E+03	1.63E+03	1.98E+03	1.30E+03	2.23E+04	2.38E+03	2.74E+04	4.59E+04	1.16E+04	1.29E+05	2.33E+03	5.28E+03	2.85E+04
107	P57053	H2BFS	HUMAN	*H2BS1*	9.88	8.08	8.21	8.54	9.81	9.09	8.54	9.26	9.35	8.44	5.94	9.38	8.03	10.36	9.01	8.80	6.15	8.31	9.28E+02	5.10E+02	2.15E+02	1.40E+02	2.15E+02	1.85E+02	1.40E+02	2.01E+02	1.40E+02	4.16E+02	1.40E+02	2.15E+02	2.15E+02	2.14E+03	3.64E+02	3.78E+02	2.15E+02	5.37E+02
108	P13804	ETFA	HUMAN	*ETFA*	11.07	10.47	13.17	11.01	11.92	9.76	10.58	12.16	11.27	12.05	10.42	12.04	13.98	11.01	9.38	10.58	10.32	9.97	1.95E+03	4.25E+03	4.02E+04	NA	8.38E+02	2.72E+02	4.63E+02	1.23E+03	4.68E+02	9.47E+03	1.05E+03	7.04E+03	4.68E+04	3.36E+03	1.85E+03	1.07E+03	1.81E+03	2.79E+03
109	P23246	SFPQ	HUMAN	*SFPQ*	10.77	14.59	13.04	11.37	11.83	12.14	11.92	11.41	12.02	10.75	11.52	10.39	11.78	10.64	10.96	11.83	10.95	12.26	1.61E+03	7.94E+04	3.65E+04	7.71E+02	7.91E+02	1.24E+03	1.11E+03	7.49E+02	7.70E+02	3.61E+03	2.14E+03	1.52E+03	9.78E+03	2.60E+03	1.48E+04	2.41E+03	2.73E+03	1.57E+04
110	O00410	IPO5	HUMAN	*IPO5*	12.41	10.58	12.74	12.34	12.29	11.56	12.21	12.19	12.02	10.99	11.76	11.44	10.62	10.31	9.99	11.45	10.84	10.54	4.77E+03	4.59E+03	2.95E+04	1.48E+03	1.07E+03	8.44E+02	1.35E+03	1.26E+03	7.71E+02	4.36E+03	2.50E+03	4.28E+03	4.08E+03	2.06E+03	5.28E+03	1.88E+03	2.55E+03	4.41E+03
111	Q15365	PCBP1	HUMAN	*PCBP1*	10.78	12.39	15.12	10.70	9.64	10.28	9.16	10.24	11.40	13.01	9.51	12.30	12.01	10.70	13.14	10.40	9.53	10.36	1.62E+03	1.70E+04	1.58E+05	NA	1.94E+02	3.70E+02	1.96E+02	3.56E+02	5.08E+02	1.88E+04	6.04E+02	8.62E+03	1.16E+04	2.71E+03	8.33E+04	9.54E+02	1.10E+03	3.81E+03
112	P62826	RAN	HUMAN	*RAN*	11.54	10.09	10.80	12.97	12.10	11.11	10.69	11.92	11.54	12.38	12.04	11.11	11.17	11.12	11.69	13.83	12.46	11.07	2.67E+03	3.17E+03	6.85E+03	2.26E+03	9.47E+02	6.28E+02	4.94E+02	1.05E+03	NA	1.21E+04	3.02E+03	3.18E+03	6.19E+03	3.63E+03	2.77E+04	9.35E+03	7.60E+03	6.55E+03
113	P08865	RSSA	HUMAN	*RPSA*	13.27	12.75	14.01	11.69	11.05	11.88	11.27	10.45	4.12	16.26	14.80	12.66	13.49	14.40	14.12	11.70	13.98	12.17	8.55E+03	2.20E+04	7.25E+04	9.54E+02	4.73E+02	1.05E+03	7.22E+02	4.05E+02	2.65E+01	1.83E+05	1.99E+04	1.14E+04	3.32E+04	3.53E+04	1.70E+05	2.21E+03	2.16E+04	1.47E+04
114	Q13765	NACA	HUMAN	*NACA*	12.71	12.51	12.13	12.09	10.69	12.13	9.06	12.05	12.18	12.48	12.15	11.52	12.71	12.23	11.27	12.59	12.25	11.26	5.86E+03	1.86E+04	1.89E+04	1.25E+03	3.73E+02	1.24E+03	1.85E+02	1.15E+03	8.55E+02	1.29E+04	3.24E+03	4.58E+03	1.91E+04	7.83E+03	1.95E+04	4.00E+03	6.57E+03	7.58E+03
115	Q01518	CAP1	HUMAN	*CAP1*	11.44	11.60	12.49	9.72	11.35	11.46	11.05	11.58	11.41	9.51	11.18	10.82	11.41	10.82	9.76	11.03	10.74	12.65	2.49E+03	9.69E+03	2.46E+04	2.71E+02	5.75E+02	7.92E+02	6.24E+02	8.37E+02	5.13E+02	1.30E+03	1.70E+03	2.40E+03	7.43E+03	2.95E+03	3.80E+03	1.43E+03	2.38E+03	2.07E+04
116	P23396	RS3	HUMAN	*RPS3*	10.49	9.71	9.60	11.19	10.49	10.23	11.55	12.19	10.49	10.15	11.21	10.49	10.18	10.19	11.20	11.05	10.15	10.91	1.35E+03	2.35E+03	2.37E+03	6.83E+02	NA	3.61E+02	8.66E+02	1.25E+03	NA	2.26E+03	1.74E+03	1.70E+03	2.84E+03	1.91E+03	1.83E+04	1.45E+03	1.63E+03	5.83E+03
117	P62937	PPIA	HUMAN	*PPIA*	11.26	14.20	11.62	12.71	11.74	9.72	12.95	11.76	12.62	15.21	12.61	13.40	12.37	12.13	13.57	12.17	13.08	13.56	2.21E+03	6.07E+04	1.30E+04	1.90E+03	7.46E+02	2.65E+02	2.23E+03	9.41E+02	1.15E+03	8.80E+04	4.44E+03	1.98E+04	1.50E+04	7.31E+03	1.14E+05	3.02E+03	1.16E+04	3.93E+04

118	P27708	PYR1	HUMAN	*CAD*	6.54	10.72	11.55	10.04	10.84	10.51	10.39	11.11	10.67	9.87	10.32	9.84	12.22	10.02	10.20	10.39	10.44	10.34	2.03E+02	5.12E+03	1.23E+04	3.29E+02	4.11E+02	4.30E+02	NA	6.15E+02	3.16E+02	1.79E+03	9.83E+02	7.02E+02	1.35E+04	1.69E+03	6.83E+03	9.48E+02	1.96E+03	3.76E+03
119	P29692	EF1D	HUMAN	*EEF1D*	11.82	11.40	10.79	11.25	11.25	10.81	11.31	11.42	11.25	10.31	12.05	11.14	11.88	11.25	10.02	12.21	10.87	10.77	3.21E+03	8.43E+03	6.80E+03	NA	NA	5.18E+02	7.40E+02	7.52E+02	NA	2.56E+03	3.03E+03	3.27E+03	1.05E+04	3.97E+03	5.50E+03	3.10E+03	2.61E+03	5.26E+03
120	Q16881	TRXR1	HUMAN	*TXNRD1*	10.77	11.89	9.92	10.73	11.68	10.48	9.22	11.35	10.92	10.75	11.08	10.63	13.79	10.39	10.04	10.02	11.08	9.29	1.61E+03	1.20E+04	3.22E+03	5.09E+02	7.15E+02	4.21E+02	2.02E+02	7.21E+02	3.70E+02	3.62E+03	1.60E+03	1.98E+03	4.09E+04	2.19E+03	5.64E+03	7.56E+02	2.98E+03	1.56E+03
121	Q9Y265	RUVB1	HUMAN	*RUVBL1*	11.97	10.13	9.60	11.33	10.75	10.13	9.43	10.54	10.37	8.91	8.94	10.18	10.53	9.78	9.68	9.72	11.01	9.63	3.56E+03	3.27E+03	2.37E+03	7.54E+02	3.87E+02	NA	2.29E+02	4.30E+02	2.61E+02	7.31E+02	4.39E+02	1.17E+03	3.80E+03	1.44E+03	3.38E+03	6.33E+02	2.85E+03	2.10E+03
122	P50395	GDIB	HUMAN	*GDI2*	11.06	9.75	9.30	10.84	10.93	11.51	9.57	10.29	10.29	10.50	9.60	12.13	9.93	9.82	9.92	9.76	11.91	12.34	1.95E+03	2.43E+03	1.71E+03	5.44E+02	4.36E+02	8.19E+02	2.48E+02	3.67E+02	NA	2.98E+03	6.35E+02	7.55E+03	2.32E+03	1.48E+03	4.81E+03	6.45E+02	5.23E+03	1.66E+04
123	P42330	AK1C3	HUMAN	*AKR1C3*	13.04	11.41	13.13	12.07	12.17	10.95	12.74	11.97	12.17	12.17	12.67	10.56	12.16	12.10	10.69	12.57	12.44	12.23	7.29E+03	8.46E+03	3.90E+04	1.23E+03	NA	5.66E+02	1.93E+03	1.09E+03	NA	1.04E+04	4.60E+03	1.85E+03	1.29E+04	7.16E+03	1.14E+04	3.97E+03	7.48E+03	1.53E+04
124	Q13200	PSMD2	HUMAN	*PSMD2*	11.28	10.72	10.59	10.72	11.14	12.33	10.97	11.04	13.66	10.20	11.86	10.76	10.44	13.31	10.58	11.95	11.51	11.56	2.25E+03	5.09E+03	5.78E+03	5.04E+02	5.01E+02	1.42E+03	5.94E+02	5.90E+02	2.35E+03	2.34E+03	2.68E+03	2.26E+03	3.52E+03	1.65E+04	1.03E+04	2.61E+03	3.99E+03	9.47E+03
125	P37837	TALDO	HUMAN	*TALDO1*	11.58	12.82	11.47	10.68	11.01	11.13	11.56	11.19	11.14	11.02	12.40	11.38	13.85	12.90	13.59	12.37	12.09	12.92	2.74E+03	2.30E+04	1.16E+04	4.91E+02	4.58E+02	6.36E+02	8.76E+02	6.49E+02	4.30E+02	4.45E+03	3.84E+03	4.07E+03	4.29E+04	1.25E+04	1.15E+05	3.45E+03	5.90E+03	2.49E+04
126	P49368	TCPG	HUMAN	*CCT3*	11.38	14.54	13.64	9.26	10.95	11.12	10.40	11.61	9.47	15.53	12.66	13.21	12.54	12.86	12.08	11.90	12.15	14.12	2.40E+03	7.72E+04	5.60E+04	2.08E+02	4.41E+02	6.35E+02	4.13E+02	8.53E+02	1.51E+02	1.10E+05	4.59E+03	1.71E+04	1.70E+04	1.21E+04	3.78E+04	2.52E+03	6.14E+03	5.80E+04
127	P62258	1433E	HUMAN	*YWHAE*	12.01	11.61	11.87	11.70	10.47	11.30	9.97	11.65	11.24	11.02	12.33	10.80	11.69	11.59	12.38	12.00	11.68	11.83	3.64E+03	9.81E+03	1.56E+04	9.58E+02	3.24E+02	7.10E+02	3.16E+02	8.75E+02	4.58E+02	4.46E+03	3.68E+03	2.36E+03	9.14E+03	5.01E+03	4.75E+04	2.70E+03	4.46E+03	1.15E+04
128	P16402	H13	HUMAN	*H1-3*	11.61	13.63	13.60	10.00	13.17	10.27	11.43	10.83	13.17	14.68	13.48	13.13	14.03	10.91	15.25	13.20	12.79	15.49	2.78E+03	4.07E+04	5.43E+04	3.22E+02	NA	3.69E+02	8.03E+02	5.16E+02	NA	6.08E+04	8.03E+03	1.62E+04	4.86E+04	3.13E+03	3.76E+05	6.10E+03	9.52E+03	1.51E+05
129	P62701	RS4X	HUMAN	*RPS4X*	9.81	9.72	12.65	10.47	10.14	10.51	10.14	10.14	10.14	13.84	12.14	10.56	13.79	9.47	9.37	9.01	8.47	9.45	8.89E+02	2.37E+03	2.77E+04	4.31E+02	NA	4.27E+02	NA	NA	NA	3.37E+04	3.22E+03	1.84E+03	4.11E+04	1.15E+03	1.79E+03	4.22E+02	5.91E+02	1.79E+03
130	P11586	C1TC	HUMAN	*MTHFD1*	12.42	12.86	12.82	11.44	10.40	10.68	11.03	12.04	12.04	11.55	12.96	11.31	12.79	11.62	12.59	12.52	11.71	13.85	4.79E+03	2.38E+04	3.13E+04	8.06E+02	3.10E+02	4.76E+02	6.18E+02	1.14E+03	NA	6.59E+03	5.61E+03	3.82E+03	2.02E+04	5.12E+03	5.55E+04	3.83E+03	4.56E+03	4.80E+04
131	P34932	HSP74	HUMAN	*HSPA4*	11.44	11.75	11.74	11.71	11.56	12.05	11.20	12.35	11.99	11.06	12.46	11.31	11.26	13.48	11.07	12.43	11.25	10.92	2.49E+03	1.08E+04	1.42E+04	9.65E+02	6.60E+02	1.17E+03	6.87E+02	1.40E+03	7.55E+02	4.57E+03	4.00E+03	3.84E+03	6.63E+03	1.85E+04	1.63E+04	3.61E+03	3.35E+03	5.88E+03
132	P62249	RS16	HUMAN	*RPS16*	8.23	9.66	9.58	10.47	10.64	10.42	10.35	9.80	9.80	9.30	9.75	10.45	9.76	9.84	9.50	10.23	10.24	9.73	3.80E+02	2.27E+03	2.32E+03	4.30E+02	3.62E+02	4.06E+02	4.00E+02	NA	NA	1.08E+03	6.97E+02	1.62E+03	2.00E+03	1.49E+03	2.40E+03	8.60E+02	1.72E+03	2.29E+03
133	O00303	EIF3F	HUMAN	*EIF3F*	12.19	10.26	12.19	12.73	12.96	12.55	12.01	11.89	13.31	11.68	12.90	11.33	10.47	11.11	9.63	12.61	11.12	10.50	4.12E+03	3.62E+03	1.98E+04	1.92E+03	1.70E+03	1.64E+03	1.18E+03	1.03E+03	1.85E+03	7.25E+03	5.41E+03	3.88E+03	3.62E+03	3.61E+03	3.09E+03	4.06E+03	3.07E+03	4.25E+03
134	P35232	PHB	HUMAN	*PHB*	10.93	10.59	12.03	9.79	10.59	9.88	10.59	10.45	10.59	14.37	11.24	12.98	12.48	10.27	9.95	10.14	10.46	13.88	1.79E+03	4.63E+03	1.76E+04	2.84E+02	NA	2.91E+02	NA	4.06E+02	NA	4.88E+04	1.78E+03	1.45E+04	1.62E+04	2.01E+03	5.03E+03	8.13E+02	1.99E+03	4.92E+04
135	O15067	PUR4	HUMAN	*PFAS*	10.10	8.61	9.77	12.58	11.60	10.39	11.22	10.44	11.13	9.74	10.39	10.33	8.67	9.23	9.64	10.03	10.20	9.95	1.06E+03	9.00E+02	2.81E+03	1.74E+03	6.79E+02	3.97E+02	7.01E+02	4.03E+02	4.27E+02	1.61E+03	1.03E+03	1.42E+03	6.41E+02	9.79E+02	3.14E+03	7.60E+02	1.68E+03	2.75E+03
136	Q14103	HNRPD	HUMAN	*HNRNPD*	11.47	11.49	13.75	10.55	11.83	10.60	13.11	11.18	11.47	10.08	11.71	10.53	12.86	10.44	10.34	12.63	11.97	9.70	2.54E+03	8.94E+03	6.05E+04	4.52E+02	7.92E+02	4.54E+02	2.49E+03	6.45E+02	NA	2.14E+03	2.42E+03	1.77E+03	2.13E+04	2.27E+03	8.00E+03	4.13E+03	5.44E+03	2.24E+03
137	P31939	PUR9	HUMAN	*ATIC*	12.02	12.13	12.14	9.82	11.04	11.75	11.55	11.45	11.03	13.52	11.15	11.54	10.55	11.51	13.77	12.26	11.27	11.84	3.66E+03	1.42E+04	1.90E+04	2.87E+02	4.69E+02	9.56E+02	8.69E+02	7.68E+02	4.00E+02	2.70E+04	1.67E+03	4.67E+03	3.85E+03	4.74E+03	1.32E+05	3.21E+03	3.40E+03	1.15E+04
138	P62269	RS18	HUMAN	*RPS18*	12.19	10.70	12.59	10.92	11.53	12.38	11.33	10.16	13.02	11.34	12.03	12.32	11.54	12.33	10.83	12.64	12.10	12.18	4.11E+03	5.05E+03	2.65E+04	5.73E+02	6.45E+02	1.46E+03	7.51E+02	3.38E+02	1.51E+03	5.64E+03	3.00E+03	8.81E+03	8.22E+03	8.40E+03	1.32E+04	4.16E+03	5.92E+03	1.48E+04
139	P41252	SYIC	HUMAN	*IARS1*	13.58	12.97	13.82	11.84	11.61	11.98	12.71	13.06	12.42	13.30	13.49	12.55	13.99	13.85	11.83	13.93	14.69	14.24	1.06E+04	2.56E+04	6.37E+04	1.05E+03	6.82E+02	1.11E+03	1.90E+03	2.27E+03	1.01E+03	2.31E+04	8.09E+03	1.05E+04	4.71E+04	2.41E+04	3.10E+04	1.00E+04	3.51E+04	6.32E+04
140	P13010	XRCC5	HUMAN	*XRCC5*	13.06	10.99	12.70	8.75	11.07	11.73	12.19	11.37	11.63	11.43	12.73	11.82	11.24	10.82	14.17	9.68	12.33	11.63	7.42E+03	6.23E+03	2.87E+04	1.57E+02	4.78E+02	9.43E+02	1.33E+03	7.28E+02	NA	6.05E+03	4.82E+03	5.91E+03	6.55E+03	2.94E+03	1.75E+05	6.17E+02	6.92E+03	9.92E+03
141	P08727	K1C19	HUMAN	*KRT19*	11.96	12.43	11.39	11.50	12.13	11.97	11.10	11.84	11.90	10.41	11.99	14.55	13.50	13.56	14.29	12.05	11.97	14.01	3.52E+03	1.75E+04	1.09E+04	8.41E+02	9.66E+02	1.11E+03	6.46E+02	9.95E+02	7.11E+02	2.78E+03	2.91E+03	4.53E+04	3.36E+04	1.97E+04	1.91E+05	2.79E+03	5.42E+03	5.37E+04
142	P62241	RS8	HUMAN	*RPS8*	10.98	9.43	12.10	12.55	13.26	11.79	11.64	12.35	9.08	11.24	10.66	10.36	11.05	12.74	9.46	11.13	10.20	13.61	1.85E+03	1.87E+03	1.85E+04	1.70E+03	2.08E+03	9.84E+02	9.21E+02	1.40E+03	1.20E+02	5.23E+03	1.22E+03	1.47E+03	5.67E+03	1.11E+04	2.21E+03	1.52E+03	1.68E+03	4.07E+04
143	P62424	RL7A	HUMAN	*RPL7A*	10.66	9.09	10.67	9.90	11.67	10.95	10.11	11.62	10.41	10.03	11.01	11.15	11.12	11.66	13.43	10.93	9.09	12.33	1.50E+03	1.40E+03	6.18E+03	3.01E+02	7.07E+02	5.68E+02	3.44E+02	8.60E+02	2.68E+02	2.05E+03	1.53E+03	3.32E+03	5.98E+03	5.28E+03	1.03E+05	1.34E+03	8.45E+02	1.64E+04
144	Q01082	SPTB2	HUMAN	*SPTBN1*	8.73	9.37	10.65	10.47	10.63	10.37	10.86	10.37	11.03	9.84	9.80	10.38	9.11	9.64	11.16	10.05	10.40	9.15	4.86E+02	1.78E+03	6.08E+03	4.30E+02	3.58E+02	3.92E+02	5.54E+02	NA	4.00E+02	1.74E+03	7.15E+02	1.50E+03	1.07E+03	1.30E+03	1.77E+04	7.69E+02	1.91E+03	1.37E+03
145	P61026	RAB10	HUMAN	*RAB10*	13.37	15.46	13.72	11.02	9.98	10.92	9.68	10.92	11.03	15.61	12.42	12.04	13.95	11.48	11.79	12.84	12.78	16.02	9.17E+03	1.46E+05	5.92E+04	6.15E+02	2.38E+02	5.55E+02	2.65E+02	5.46E+02	4.00E+02	1.16E+05	3.91E+03	7.02E+03	4.60E+04	4.67E+03	3.00E+04	4.77E+03	9.41E+03	2.19E+05
146	P55209	NP1L1	HUMAN	*NAP1L1*	11.57	13.97	11.56	11.54	11.40	11.89	11.00	12.29	11.34	10.02	9.42	10.03	9.10	10.15	13.88	9.74	12.29	9.26	2.72E+03	5.17E+04	1.24E+04	8.61E+02	5.92E+02	1.05E+03	6.03E+02	1.34E+03	4.88E+02	2.02E+03	5.73E+02	9.52E+02	1.06E+03	1.85E+03	1.43E+05	6.39E+02	6.74E+03	1.50E+03
147	P63244	RACK1	HUMAN	*RACK1*	12.24	12.38	12.69	10.65	11.51	12.92	11.26	12.48	12.24	11.89	13.41	12.08	13.25	14.44	11.46	11.12	11.40	13.64	4.25E+03	1.69E+04	2.85E+04	4.83E+02	6.39E+02	2.11E+03	7.17E+02	1.53E+03	NA	8.43E+03	7.65E+03	7.30E+03	2.81E+04	3.61E+04	2.28E+04	1.51E+03	3.69E+03	4.15E+04
148	P68871	HBB	HUMAN	*HBB*	11.76	11.47	10.96	10.00	11.57	8.98	10.31	11.58	11.74	11.70	11.46	11.86	10.86	10.76	11.79	12.25	12.40	11.65	3.07E+03	8.83E+03	7.83E+03	3.22E+02	6.64E+02	1.74E+02	3.88E+02	8.39E+02	6.39E+02	7.37E+03	2.05E+03	6.10E+03	4.92E+03	2.82E+03	3.00E+04	3.20E+03	7.30E+03	1.00E+04
149	P83731	RL24	HUMAN	*RPL24*	10.89	10.21	10.67	10.35	11.67	11.69	11.81	12.02	11.36	9.96	11.61	13.33	9.92	11.70	10.82	10.90	10.76	10.38	1.74E+03	3.48E+03	6.19E+03	3.99E+02	7.07E+02	9.18E+02	1.03E+03	1.12E+03	4.96E+02	1.93E+03	2.27E+03	1.88E+04	2.29E+03	5.41E+03	1.30E+04	1.31E+03	2.41E+03	3.89E+03
150	P17987	TCPA	HUMAN	*TCP1*	11.81	11.29	10.95	12.36	12.85	12.45	12.79	11.73	11.36	12.40	11.54	11.71	11.47	11.47	11.49	12.12	12.10	12.64	3.19E+03	7.77E+03	7.74E+03	1.50E+03	1.57E+03	1.53E+03	2.00E+03	9.23E+02	4.95E+02	1.22E+04	2.17E+03	5.40E+03	7.77E+03	4.62E+03	2.35E+04	2.92E+03	5.92E+03	2.05E+04
151	Q02878	RL6	HUMAN	*RPL6*	9.10	12.89	11.64	11.02	10.31	12.89	10.45	10.88	9.85	8.29	9.90	9.69	11.19	7.94	11.18	9.65	6.43	10.38	5.91E+02	2.42E+04	1.31E+04	6.15E+02	2.92E+02	2.06E+03	4.24E+02	5.30E+02	1.89E+02	3.36E+02	7.60E+02	5.21E+02	6.33E+03	4.04E+02	1.80E+04	6.06E+02	2.36E+02	3.87E+03
152	P30050	RL12	HUMAN	*RPL12*	11.38	12.97	12.56	11.51	10.64	11.26	11.17	12.06	11.54	13.90	13.67	11.54	11.41	10.99	13.23	12.68	11.17	12.83	2.40E+03	2.56E+04	2.60E+04	8.49E+02	3.61E+02	6.93E+02	6.77E+02	1.16E+03	NA	3.52E+04	9.12E+03	4.66E+03	7.44E+03	3.31E+03	8.91E+04	4.27E+03	3.18E+03	2.35E+04
153	P78344	IF4G2	HUMAN	*EIF4G2*	9.42	10.89	8.59	10.47	10.05	10.64	10.82	10.43	11.14	8.58	9.72	10.11	8.49	8.31	9.31	10.00	10.24	8.92	7.10E+02	5.78E+03	6.16E+02	4.30E+02	2.49E+02	4.66E+02	5.39E+02	4.00E+02	4.30E+02	5.03E+02	6.83E+02	1.06E+03	5.04E+02	5.20E+02	1.52E+03	7.46E+02	1.73E+03	1.08E+03
154	P24534	EF1B	HUMAN	*EEF1B2*	11.44	13.25	11.64	13.25	12.62	11.98	10.94	11.09	12.21	12.25	12.26	11.23	12.21	11.68	12.72	12.16	12.47	12.53	2.49E+03	3.11E+04	1.31E+04	2.75E+03	1.35E+03	1.12E+03	5.81E+02	6.08E+02	NA	1.10E+04	3.51E+03	3.57E+03	1.33E+04	5.34E+03	6.13E+04	3.00E+03	7.63E+03	1.89E+04
155	P46782	RS5	HUMAN	*RPS5*	12.53	12.30	15.05	11.30	10.31	12.14	12.29	12.03	11.96	11.01	11.34	12.06	12.69	10.74	14.30	11.24	12.13	14.14	5.19E+03	1.60E+04	1.50E+05	7.37E+02	2.92E+02	1.25E+03	1.42E+03	1.13E+03	7.41E+02	4.41E+03	1.90E+03	7.13E+03	1.88E+04	2.78E+03	1.92E+05	1.63E+03	6.07E+03	5.89E+04
156	Q92945	FUBP2	HUMAN	*KHSRP*	11.65	12.51	13.09	10.80	11.42	10.51	10.08	11.42	11.42	10.84	11.37	11.42	11.92	9.94	11.75	11.21	12.08	13.18	2.86E+03	1.85E+04	3.78E+04	5.30E+02	NA	4.30E+02	3.36E+02	NA	NA	3.88E+03	1.94E+03	4.21E+03	1.08E+04	1.60E+03	2.91E+04	1.60E+03	5.85E+03	3.02E+04
157	P61353	RL27	HUMAN	*RPL27*	11.56	9.60	10.15	9.60	9.81	9.31	9.81	9.08	9.81	10.77	9.47	10.80	8.78	10.41	9.81	11.07	10.43	9.23	2.70E+03	2.16E+03	3.96E+03	2.53E+02	NA	2.09E+02	NA	1.82E+02	NA	3.67E+03	5.90E+02	2.36E+03	7.36E+02	2.22E+03	4.09E+03	1.46E+03	1.95E+03	1.47E+03
158	O00299	CLIC1	HUMAN	*CLIC1*	10.19	9.53	9.69	11.87	13.22	11.84	11.08	10.73	12.91	9.24	8.76	10.41	11.38	9.21	9.45	11.54	9.28	8.70	1.12E+03	2.03E+03	2.59E+03	1.07E+03	2.03E+03	1.02E+03	6.36E+02	4.84E+02	1.41E+03	1.02E+03	3.99E+02	1.55E+03	7.25E+03	9.68E+02	2.18E+03	1.99E+03	9.45E+02	8.65E+02
159	O00764	PDXK	HUMAN	*PDXK*	9.74	10.10	9.00	12.09	10.40	11.11	9.48	11.06	12.14	9.48	9.85	10.13	11.18	9.09	9.44	10.94	9.50	10.55	8.55E+02	3.20E+03	1.18E+03	1.25E+03	3.10E+02	6.30E+02	2.35E+02	5.95E+02	8.32E+02	1.27E+03	7.39E+02	1.09E+03	6.26E+03	8.88E+02	2.14E+03	1.35E+03	1.08E+03	4.43E+03
160	Q9NZL4	HPBP1	HUMAN	*HSPBP1*	11.52	11.21	10.50	10.63	11.69	11.22	11.40	10.75	11.80	12.28	13.80	12.16	12.60	11.83	11.33	12.07	11.44	11.53	2.63E+03	7.30E+03	5.36E+03	4.77E+02	7.21E+02	6.77E+02	7.84E+02	4.90E+02	6.63E+02	1.12E+04	9.96E+03	7.74E+03	1.77E+04	5.94E+03	2.04E+04	2.84E+03	3.81E+03	9.21E+03
161	O75390	CISY	HUMAN	*CS*	9.89	13.70	12.92	12.05	11.30	10.51	10.25	10.92	11.30	11.46	11.18	12.35	11.52	13.26	9.58	9.67	11.42	10.60	9.31E+02	4.27E+04	3.35E+04	1.21E+03	NA	4.30E+02	3.74E+02	5.47E+02	NA	6.17E+03	1.71E+03	9.00E+03	8.09E+03	1.59E+04	2.84E+03	6.11E+02	3.74E+03	4.60E+03
162	P35908	K22E	HUMAN	*KRT2*	9.72	9.47	9.27	10.15	10.91	9.78	9.64	10.43	10.65	9.61	10.52	10.69	10.52	9.93	10.56	10.03	10.35	10.75	8.45E+02	1.93E+03	1.66E+03	3.52E+02	4.30E+02	2.75E+02	2.58E+02	4.00E+02	3.11E+02	1.43E+03	1.12E+03	2.11E+03	3.77E+03	1.59E+03	1.01E+04	7.60E+02	1.85E+03	5.16E+03
163	Q15019	Sep-02	HUMAN	*SEPTIN2*	12.73	10.80	10.14	12.68	13.96	10.92	11.50	12.12	12.68	11.05	13.03	11.93	11.55	12.50	9.21	12.13	11.60	9.51	5.91E+03	5.43E+03	3.93E+03	1.86E+03	3.36E+03	5.55E+02	8.43E+02	1.20E+03	1.20E+03	4.55E+03	5.89E+03	6.47E+03	8.25E+03	9.42E+03	1.14E+03	2.95E+03	4.24E+03	1.90E+03
164	P12268	IMDH2	HUMAN	*IMPDH2*	10.92	9.84	9.65	11.43	10.39	10.39	10.39	10.27	10.39	9.80	9.34	12.23	10.54	11.99	9.79	10.65	10.50	9.70	1.78E+03	2.61E+03	2.48E+03	8.01E+02	NA	NA	NA	3.64E+02	NA	1.69E+03	5.49E+02	8.18E+03	3.82E+03	6.63E+03	4.00E+03	1.12E+03	2.03E+03	2.24E+03
165	P12004	PCNA	HUMAN	*PCNA*	11.51	12.62	9.69	9.89	11.45	11.60	11.10	11.50	10.55	10.44	11.51	11.70	9.63	10.12	10.78	12.84	11.46	9.06	2.61E+03	2.01E+04	2.58E+03	3.01E+02	6.15E+02	8.68E+02	6.45E+02	7.93E+02	2.92E+02	2.84E+03	2.11E+03	5.34E+03	1.77E+03	1.81E+03	1.25E+04	4.77E+03	3.85E+03	1.25E+03
166	P27824	CALX	HUMAN	*CANX*	10.68	9.39	10.51	11.12	11.90	10.48	10.45	11.08	10.48	9.93	10.24	10.63	12.86	10.00	10.27	11.08	9.92	9.69	1.53E+03	1.82E+03	5.43E+03	6.54E+02	8.30E+02	NA	4.25E+02	6.03E+02	NA	1.87E+03	9.35E+02	1.99E+03	2.12E+04	1.67E+03	7.40E+03	1.47E+03	1.41E+03	2.21E+03
167	P27695	APEX1	HUMAN	*APEX1*	13.96	10.63	10.55	12.31	13.86	11.64	11.74	11.43	11.63	11.67	11.94	10.74	12.61	12.99	11.79	13.33	11.61	12.75	1.37E+04	4.77E+03	5.61E+03	1.45E+03	3.15E+03	8.93E+02	9.85E+02	7.60E+02	5.92E+02	7.23E+03	2.82E+03	2.24E+03	1.78E+04	1.32E+04	3.00E+04	6.64E+03	4.26E+03	2.22E+04
168	P14923	PLAK	HUMAN	*JUP*	10.71	9.40	9.09	10.26	10.80	10.40	10.42	9.69	11.34	8.80	9.35	9.69	9.10	8.62	9.80	8.99	9.38	9.88	1.56E+03	1.83E+03	1.33E+03	3.78E+02	4.00E+02	4.00E+02	4.17E+02	NA	4.89E+02	6.45E+02	5.52E+02	5.24E+02	1.06E+03	6.45E+02	4.06E+03	4.18E+02	1.00E+03	2.60E+03
169	P46777	RL5	HUMAN	*RPL5*	10.99	10.84	9.68	10.76	10.88	13.90	10.16	11.48	10.88	9.34	13.77	11.89	10.07	11.00	10.20	10.99	12.18	10.87	1.86E+03	5.59E+03	2.56E+03	5.17E+02	4.22E+02	4.11E+03	3.54E+02	7.84E+02	NA	1.12E+03	9.82E+03	6.22E+03	2.60E+03	3.34E+03	6.87E+03	1.38E+03	6.28E+03	5.67E+03
170	P23284	PPIB	HUMAN	*PPIB*	10.82	12.54	13.38	10.47	10.94	9.31	11.22	8.81	10.52	10.14	9.98	11.17	14.05	9.41	9.51	9.56	9.69	12.29	1.67E+03	1.89E+04	4.66E+04	4.30E+02	4.40E+02	2.10E+02	7.00E+02	1.58E+02	2.87E+02	2.24E+03	7.98E+02	3.36E+03	4.92E+04	1.11E+03	2.47E+03	5.74E+02	1.21E+03	1.60E+04
171	Q96AG4	LRC59	HUMAN	*LRRC59*	9.13	8.69	8.73	9.72	10.26	14.72	11.41	10.43	11.30	12.25	9.62	9.77	9.21	8.18	9.23	9.48	9.80	8.82	6.02E+02	9.74E+02	7.85E+02	2.70E+02	2.84E+02	7.23E+03	7.90E+02	4.01E+02	4.76E+02	1.10E+04	6.44E+02	6.13E+02	1.18E+03	4.74E+02	1.21E+03	5.50E+02	1.30E+03	9.83E+02
172	Q01813	PFKAP	HUMAN	*PFKP*	9.34	8.70	9.82	10.94	10.79	11.88	11.56	10.75	11.19	10.24	11.86	10.67	10.13	10.56	9.52	10.63	8.27	9.26	6.77E+02	9.83E+02	2.93E+03	5.82E+02	3.97E+02	1.04E+03	8.71E+02	4.89E+02	4.42E+02	2.43E+03	2.67E+03	2.08E+03	2.73E+03	2.46E+03	2.50E+03	1.10E+03	5.30E+02	1.50E+03
173	P13645	K1C10	HUMAN	*KRT10*	12.82	13.25	10.28	9.30	11.49	10.51	11.21	15.00	11.49	10.27	14.06	11.65	12.80	11.08	11.85	12.40	10.89	11.33	6.28E+03	3.13E+04	4.47E+03	2.11E+02	NA	4.30E+02	6.93E+02	8.53E+03	NA	2.50E+03	1.19E+04	5.13E+03	2.04E+04	3.52E+03	3.13E+04	3.53E+03	2.64E+03	7.95E+03
174	Q16658	FSCN1	HUMAN	*FSCN1*	11.20	10.58	11.24	9.72	10.18	10.40	10.95	11.15	10.93	10.53	10.85	11.01	12.35	10.93	11.67	10.30	10.79	11.67	2.13E+03	4.60E+03	9.71E+03	2.71E+02	2.71E+02	4.00E+02	5.84E+02	6.31E+02	NA	3.05E+03	1.38E+03	2.92E+03	1.48E+04	3.18E+03	2.73E+04	8.98E+02	2.47E+03	1.03E+04
175	Q99832	TCPH	HUMAN	*CCT7*	9.20	10.16	8.92	10.09	10.91	10.48	10.09	10.24	11.26	8.90	9.41	9.84	9.11	12.64	9.15	10.64	10.02	12.86	6.25E+02	3.35E+03	1.06E+03	3.38E+02	4.30E+02	4.20E+02	NA	3.57E+02	4.65E+02	7.19E+02	5.71E+02	7.00E+02	1.07E+03	1.04E+04	8.87E+02	1.11E+03	1.50E+03	2.39E+04
176	Q8WUM4	PDC6I	HUMAN	*PDCD6IP*	10.36	10.19	8.94	9.99	10.74	10.19	10.47	11.38	11.53	8.64	8.32	9.41	9.70	11.43	11.41	7.71	11.30	8.70	1.25E+03	3.41E+03	1.08E+03	3.20E+02	3.86E+02	NA	4.30E+02	7.33E+02	5.55E+02	5.38E+02	3.20E+02	2.49E+02	1.90E+03	4.49E+03	2.20E+04	2.28E+02	3.46E+03	8.60E+02
177	P39656	OST48	HUMAN	*DDOST*	9.24	10.53	9.73	10.02	10.02	10.06	10.01	10.02	10.42	9.72	9.41	9.91	9.11	10.32	11.30	10.46	10.02	11.09	6.40E+02	4.43E+03	2.69E+03	NA	NA	3.25E+02	3.23E+02	NA	2.69E+02	1.58E+03	5.69E+02	7.93E+02	1.07E+03	2.09E+03	2.00E+04	9.88E+02	1.50E+03	6.67E+03
178	Q99873	ANM1	HUMAN	*PRMT1*	11.67	11.29	10.92	11.65	10.63	11.46	11.02	11.73	10.62	11.36	10.26	10.03	12.06	11.21	11.37	11.40	10.87	11.50	2.90E+03	7.73E+03	7.53E+03	9.31E+02	3.59E+02	7.91E+02	6.14E+02	9.27E+02	3.06E+02	5.72E+03	9.46E+02	9.43E+02	1.20E+04	3.86E+03	2.11E+04	1.81E+03	2.60E+03	9.04E+03
179	P68366	TBA4A	HUMAN	*TUBA4A*	12.27	13.33	13.51	10.84	11.05	11.63	11.89	10.72	12.62	13.94	12.50	12.41	11.42	9.88	10.26	12.03	10.91	11.41	4.34E+03	3.30E+04	5.11E+04	5.44E+02	4.72E+02	8.85E+02	1.09E+03	4.81E+02	1.15E+03	3.63E+04	4.10E+03	9.41E+03	7.52E+03	1.53E+03	7.34E+03	2.75E+03	2.68E+03	8.43E+03
180	P26639	SYTC	HUMAN	*TARS1*	10.84	9.04	12.03	11.59	9.76	9.64	11.87	11.18	11.31	9.30	9.76	10.77	9.35	9.58	9.38	9.76	10.04	8.60	1.69E+03	1.35E+03	1.76E+04	8.92E+02	NA	2.53E+02	1.07E+03	6.45E+02	4.79E+02	1.07E+03	6.99E+02	2.30E+03	1.36E+03	1.25E+03	1.84E+03	6.45E+02	1.52E+03	7.69E+02
181	Q7KZF4	SND1	HUMAN	*SND1*	11.77	11.72	10.27	10.10	11.29	11.09	9.50	10.47	11.29	12.80	11.02	12.29	11.32	12.55	10.83	12.20	11.49	11.26	3.09E+03	1.06E+04	4.42E+03	3.41E+02	NA	6.19E+02	2.38E+02	4.11E+02	NA	1.62E+04	1.54E+03	8.55E+03	6.97E+03	9.77E+03	1.31E+04	3.08E+03	3.94E+03	7.56E+03
182	Q01105	SET	HUMAN	*SET*	9.44	8.60	9.37	10.20	10.91	11.33	11.15	11.11	11.99	9.20	9.80	10.01	8.67	10.53	9.60	10.29	9.60	8.67	7.17E+02	8.94E+02	1.85E+03	3.64E+02	4.30E+02	7.28E+02	6.68E+02	6.15E+02	7.53E+02	9.81E+02	7.17E+02	9.15E+02	6.41E+02	2.40E+03	2.93E+03	8.90E+02	1.15E+03	8.33E+02
183	P37802	TAGL2	HUMAN	*TAGLN2*	10.53	9.34	9.62	11.12	10.91	10.51	10.97	9.53	11.88	9.72	10.07	10.40	9.72	10.76	9.18	11.25	10.74	9.62	1.39E+03	1.73E+03	2.41E+03	6.56E+02	4.30E+02	4.30E+02	5.94E+02	2.34E+02	7.00E+02	1.57E+03	8.44E+02	1.53E+03	1.93E+03	2.83E+03	1.01E+03	1.64E+03	2.39E+03	2.08E+03
184	P52907	CAZA1	HUMAN	*CAPZA1*	9.85	9.50	10.60	11.40	11.36	10.23	11.37	11.67	11.97	9.90	10.38	10.63	9.60	10.02	9.37	10.48	10.31	9.78	9.14E+02	1.98E+03	5.85E+03	7.89E+02	5.77E+02	3.61E+02	7.70E+02	8.89E+02	7.42E+02	1.84E+03	1.02E+03	1.99E+03	1.73E+03	1.69E+03	1.81E+03	1.00E+03	1.80E+03	2.40E+03
185	P04844	RPN2	HUMAN	*RPN2*	10.48	11.89	10.16	10.82	11.20	8.60	9.98	10.26	10.26	9.84	10.37	10.42	9.20	11.34	10.04	10.93	9.87	9.45	1.34E+03	1.19E+04	4.02E+03	5.39E+02	5.20E+02	1.43E+02	3.17E+02	NA	NA	1.74E+03	1.01E+03	1.57E+03	1.18E+03	4.23E+03	5.63E+03	1.34E+03	1.36E+03	1.79E+03
186	Q15366	PCBP2	HUMAN	*PCBP2*	9.22	9.36	8.68	10.35	9.69	9.96	9.69	8.95	11.35	9.80	10.14	10.32	9.33	8.80	9.59	10.09	9.02	9.82	6.31E+02	1.76E+03	7.21E+02	4.00E+02	NA	3.06E+02	NA	1.71E+02	4.91E+02	1.68E+03	8.81E+02	1.39E+03	1.34E+03	7.27E+02	2.86E+03	7.89E+02	8.08E+02	2.47E+03
187	P14868	SYDC	HUMAN	*DARS1*	10.42	10.75	11.29	10.32	10.51	10.42	10.47	10.37	10.52	12.28	12.02	10.72	11.71	9.94	10.23	9.54	12.32	11.35	1.30E+03	5.21E+03	1.01E+04	3.92E+02	3.32E+02	4.05E+02	4.30E+02	3.87E+02	2.87E+02	1.12E+04	2.99E+03	2.18E+03	9.30E+03	1.60E+03	7.07E+03	5.70E+02	6.91E+03	8.08E+03
188	P42167	LAP2B	HUMAN	*TMPO*	11.61	12.11	9.73	10.44	11.20	11.59	10.47	9.54	11.54	10.73	10.35	11.77	11.19	9.28	12.15	10.45	11.50	11.65	2.79E+03	1.39E+04	2.70E+03	4.22E+02	5.21E+02	8.59E+02	4.30E+02	2.36E+02	5.59E+02	3.57E+03	1.01E+03	5.68E+03	6.30E+03	1.01E+03	3.97E+04	9.83E+02	3.96E+03	1.01E+04
189	P30048	PRDX3	HUMAN	*PRDX3*	10.54	10.92	10.16	10.43	10.57	10.73	12.15	10.98	12.90	10.31	10.72	10.90	10.39	12.24	9.53	10.33	11.15	12.70	1.40E+03	5.92E+03	4.02E+03	4.19E+02	3.45E+02	4.93E+02	1.30E+03	5.68E+02	1.40E+03	2.57E+03	1.27E+03	2.62E+03	3.38E+03	7.90E+03	2.58E+03	9.14E+02	3.13E+03	2.14E+04
190	P15880	RS2	HUMAN	*RPS2*	9.59	13.41	12.54	8.11	10.21	12.04	9.60	12.31	10.92	9.03	10.33	10.97	11.26	12.71	11.72	10.37	11.46	9.80	7.80E+02	3.48E+04	2.56E+04	1.14E+02	2.75E+02	1.16E+03	2.53E+02	1.36E+03	3.70E+02	8.30E+02	9.92E+02	2.80E+03	6.63E+02	1.09E+04	2.83E+04	9.34E+02	3.85E+03	2.43E+03
191	P22087	FBRL	HUMAN	*FBL*	10.72	12.16	11.94	11.28	12.14	12.33	10.96	10.56	10.51	11.30	11.19	11.71	10.67	11.56	13.27	11.87	13.15	11.17	1.56E+03	1.45E+04	1.65E+04	7.25E+02	9.71E+02	1.41E+03	5.91E+02	4.34E+02	2.86E+02	5.47E+03	1.72E+03	5.41E+03	4.22E+03	4.90E+03	9.22E+04	2.47E+03	1.22E+04	7.09E+03
192	P61981	1433G	HUMAN	*YWHAG*	10.95	11.67	9.01	10.77	10.91	10.00	10.35	10.93	11.14	9.51	9.85	9.86	11.45	10.75	9.78	9.68	10.28	10.05	1.81E+03	1.02E+04	1.20E+03	5.20E+02	4.30E+02	3.13E+02	4.00E+02	5.49E+02	4.30E+02	1.31E+03	7.38E+02	7.24E+02	7.68E+03	2.80E+03	3.94E+03	6.15E+02	1.76E+03	2.99E+03
193	P04792	HSPB1	HUMAN	*HSPB1*	10.81	12.69	12.47	11.42	10.48	11.80	11.46	11.75	11.18	11.61	10.72	12.10	12.84	11.01	11.41	10.18	10.83	10.53	1.65E+03	2.11E+04	2.43E+04	7.97E+02	3.26E+02	9.91E+02	8.21E+02	9.38E+02	4.41E+02	6.89E+03	1.26E+03	7.40E+03	2.10E+04	3.37E+03	2.20E+04	8.31E+02	2.53E+03	4.36E+03
194	P15121	ALDR	HUMAN	*AKR1B1*	12.77	10.34	11.52	11.71	9.43	11.43	9.53	12.11	11.71	11.52	13.00	10.50	12.16	12.24	9.18	13.59	11.90	12.16	6.10E+03	3.84E+03	1.20E+04	NA	1.71E+02	7.73E+02	2.43E+02	1.19E+03	NA	6.47E+03	5.78E+03	1.73E+03	1.29E+04	7.89E+03	1.02E+03	7.96E+03	5.19E+03	1.46E+04
195	P30101	PDIA3	HUMAN	*PDIA3*	12.79	9.65	9.65	11.31	10.91	11.15	9.87	11.04	11.14	9.98	10.52	11.82	9.27	10.32	9.74	11.98	10.36	9.88	6.15E+03	2.25E+03	2.48E+03	7.43E+02	4.30E+02	6.45E+02	2.97E+02	5.90E+02	4.30E+02	1.96E+03	1.12E+03	5.90E+03	1.26E+03	2.09E+03	3.68E+03	2.66E+03	1.86E+03	2.59E+03
196	P62277	RS13	HUMAN	*RPS13*	9.59	8.39	9.13	10.46	10.46	10.49	10.46	10.54	10.94	12.18	10.01	10.78	9.24	12.46	9.27	10.46	9.97	11.34	7.83E+02	7.19E+02	1.39E+03	NA	NA	4.23E+02	NA	4.30E+02	3.77E+02	1.04E+04	8.14E+02	2.33E+03	1.23E+03	9.20E+03	1.36E+03	9.92E+02	1.44E+03	8.06E+03
197	P08758	ANXA5	HUMAN	*ANXA5*	9.21	13.90	11.45	10.41	11.76	10.13	12.14	13.08	12.51	8.83	10.15	11.87	12.44	12.44	11.80	10.32	11.14	11.84	6.28E+02	4.93E+04	1.14E+04	4.13E+02	7.53E+02	3.38E+02	1.29E+03	2.29E+03	1.07E+03	6.73E+02	8.85E+02	6.15E+03	1.57E+04	9.08E+03	3.03E+04	9.06E+02	3.11E+03	1.15E+04
198	O75874	IDHC	HUMAN	*IDH1*	8.82	9.98	11.21	10.44	12.24	9.43	11.46	11.18	11.18	8.23	14.58	10.53	11.59	11.67	9.34	13.64	11.99	9.42	5.08E+02	2.91E+03	9.49E+03	4.22E+02	1.04E+03	2.24E+02	8.18E+02	6.43E+02	NA	3.08E+02	1.71E+04	1.78E+03	8.52E+03	5.32E+03	1.65E+03	8.19E+03	5.51E+03	1.74E+03
199	Q15393	SF3B3	HUMAN	*SF3B3*	10.96	9.38	9.66	10.40	10.82	10.40	9.46	10.18	10.01	11.92	9.75	10.61	9.14	10.50	9.78	11.34	10.94	10.67	1.82E+03	1.80E+03	2.51E+03	NA	4.05E+02	4.00E+02	2.32E+02	3.44E+02	2.08E+02	8.64E+03	6.96E+02	1.94E+03	1.10E+03	2.36E+03	3.94E+03	1.74E+03	2.73E+03	4.86E+03
200	P68371	TBB4B	HUMAN	*TUBB4B*	10.91	10.55	11.55	11.07	11.22	11.14	11.26	9.84	11.22	15.11	11.18	10.47	11.76	11.82	11.88	11.35	11.67	10.38	1.76E+03	4.51E+03	1.24E+04	6.32E+02	NA	6.42E+02	7.17E+02	2.80E+02	NA	8.21E+04	1.71E+03	1.66E+03	9.66E+03	5.87E+03	3.21E+04	1.75E+03	4.43E+03	3.87E+03
201	P63010	AP2B1	HUMAN	*AP2B1*	8.55	8.56	9.28	9.25	10.08	9.25	9.51	10.26	10.85	8.80	9.89	10.74	9.14	8.55	9.22	9.48	7.99	8.95	4.43E+02	8.56E+02	1.68E+03	NA	2.53E+02	NA	2.39E+02	3.61E+02	3.55E+02	6.45E+02	7.56E+02	2.23E+03	1.10E+03	6.14E+02	1.16E+03	5.49E+02	4.58E+02	1.12E+03
202	Q96AE4	FUBP1	HUMAN	*FUBP1*	10.57	9.07	10.22	10.50	10.50	11.20	10.50	10.54	10.56	12.72	9.32	10.80	10.36	9.61	10.50	11.35	8.90	8.66	1.42E+03	1.37E+03	4.22E+03	NA	NA	6.68E+02	NA	4.30E+02	2.95E+02	1.53E+04	5.40E+02	2.36E+03	3.29E+03	1.27E+03	9.53E+03	1.76E+03	7.53E+02	8.26E+02
203	O43390	HNRPR	HUMAN	*HNRNPR*	8.96	9.49	9.57	10.22	10.43	10.70	10.17	10.17	11.03	9.58	9.66	12.52	9.39	10.81	11.14	9.30	9.56	12.00	5.48E+02	1.97E+03	2.30E+03	3.69E+02	3.16E+02	4.83E+02	NA	3.40E+02	4.00E+02	1.39E+03	6.59E+02	1.02E+04	1.41E+03	2.93E+03	1.73E+04	4.96E+02	1.12E+03	1.30E+04
204	P20073	ANXA7	HUMAN	*ANXA7*	12.06	10.34	10.28	11.41	13.37	11.77	11.43	11.98	11.43	8.80	10.08	10.55	11.71	9.97	9.60	9.76	8.78	9.29	3.77E+03	3.84E+03	4.45E+03	7.91E+02	2.25E+03	9.70E+02	8.00E+02	1.09E+03	5.20E+02	6.45E+02	8.50E+02	1.83E+03	9.27E+03	1.64E+03	2.91E+03	6.45E+02	7.03E+02	1.55E+03
205	P25398	RS12	HUMAN	*RPS12*	10.32	10.10	9.81	11.61	10.71	11.56	10.81	10.73	10.41	9.90	11.42	11.42	10.30	10.35	12.26	12.00	10.15	10.65	1.21E+03	3.19E+03	2.91E+03	9.04E+02	3.78E+02	8.47E+02	5.37E+02	4.84E+02	2.68E+02	1.84E+03	2.00E+03	4.23E+03	3.16E+03	2.12E+03	4.32E+04	2.70E+03	1.63E+03	4.78E+03
206	P27348	1433T	HUMAN	*YWHAQ*	9.92	12.61	12.27	10.47	10.91	10.38	10.47	10.76	9.93	9.15	8.48	10.10	8.73	12.41	9.34	11.70	9.00	8.85	9.50E+02	1.99E+04	2.10E+04	4.30E+02	4.30E+02	3.95E+02	4.30E+02	4.92E+02	1.98E+02	9.28E+02	3.46E+02	1.05E+03	6.99E+02	8.86E+03	1.65E+03	2.20E+03	8.00E+02	1.01E+03
207	Q16719	KYNU	HUMAN	*KYNU*	9.21	8.73	9.29	6.07	9.21	9.47	9.21	10.43	9.21	8.67	9.22	9.94	8.37	8.57	9.28	8.35	9.41	9.20	6.29E+02	1.01E+03	1.70E+03	5.58E+01	NA	2.29E+02	NA	4.00E+02	NA	5.61E+02	5.11E+02	8.24E+02	4.18E+02	6.22E+02	1.41E+03	3.02E+02	1.02E+03	1.43E+03
208	P20700	LMNB1	HUMAN	*LMNB1*	11.19	8.96	9.60	11.49	10.31	11.12	11.93	10.81	10.31	9.25	10.07	10.67	9.77	9.58	10.01	11.50	10.27	10.31	2.11E+03	1.25E+03	2.36E+03	8.36E+02	2.93E+02	6.34E+02	1.12E+03	5.08E+02	NA	1.03E+03	8.45E+02	2.08E+03	2.01E+03	1.25E+03	5.41E+03	1.94E+03	1.76E+03	3.68E+03
209	P61088	UBE2N	HUMAN	*UBE2N*	9.81	10.03	9.15	9.84	9.84	9.84	10.23	10.92	9.84	9.86	10.30	10.22	8.64	9.38	9.56	11.33	9.45	9.73	8.92E+02	3.03E+03	1.43E+03	NA	NA	NA	3.69E+02	5.45E+02	NA	1.77E+03	9.73E+02	1.22E+03	6.22E+02	1.09E+03	2.72E+03	1.73E+03	1.05E+03	2.28E+03
210	P05387	RLA2	HUMAN	*RPLP2*	10.33	10.54	10.48	11.53	11.53	10.92	10.28	10.51	11.03	13.37	10.36	10.31	10.13	10.92	11.87	11.25	11.55	11.46	1.22E+03	4.45E+03	5.27E+03	8.60E+02	6.45E+02	NA	3.83E+02	4.21E+02	4.00E+02	2.43E+04	1.01E+03	1.38E+03	2.75E+03	3.15E+03	3.20E+04	1.65E+03	4.10E+03	8.79E+03
211	Q9UKM9	RALY	HUMAN	*RALY*	10.19	9.11	8.94	10.58	11.95	13.27	10.47	10.88	12.11	9.35	9.35	10.13	10.01	9.50	9.73	9.32	10.09	8.35	1.12E+03	1.42E+03	1.08E+03	4.62E+02	8.56E+02	2.66E+03	4.30E+02	5.33E+02	8.19E+02	1.12E+03	5.50E+02	1.10E+03	2.48E+03	1.18E+03	3.62E+03	5.00E+02	1.56E+03	5.70E+02
212	P52272	HNRPM	HUMAN	*HNRNPM*	9.64	9.24	11.34	10.03	10.91	9.87	8.40	9.90	10.92	9.96	10.72	13.78	10.67	10.41	11.07	10.54	10.42	9.24	8.04E+02	1.60E+03	1.05E+04	3.28E+02	4.30E+02	2.89E+02	1.31E+02	2.89E+02	3.70E+02	1.93E+03	1.27E+03	2.61E+04	4.23E+03	2.21E+03	1.63E+04	1.04E+03	1.93E+03	1.48E+03
213	P39019	RS19	HUMAN	*RPS19*	12.16	11.19	12.30	11.11	11.85	10.28	10.34	6.12	11.85	13.94	11.36	11.88	11.80	12.75	12.24	12.06	9.65	12.46	4.04E+03	7.22E+03	2.15E+04	6.51E+02	8.01E+02	3.72E+02	3.97E+02	6.00E+01	NA	3.63E+04	1.92E+03	6.18E+03	9.91E+03	1.12E+04	4.27E+04	2.82E+03	1.18E+03	1.80E+04
214	Q9BSJ8	ESYT1	HUMAN	*ESYT1*	10.11	10.56	12.86	10.21	10.36	10.70	10.11	9.70	11.69	9.17	9.38	9.65	8.45	7.77	12.33	7.90	11.03	8.45	1.07E+03	4.54E+03	3.21E+04	3.66E+02	3.03E+02	4.83E+02	NA	2.58E+02	6.15E+02	9.47E+02	5.59E+02	4.87E+02	4.73E+02	3.58E+02	4.56E+04	2.47E+02	2.89E+03	6.45E+02
215	Q15185	TEBP	HUMAN	*PTGES3*	12.29	9.60	9.27	10.68	10.57	10.36	10.89	10.11	10.57	12.12	11.09	10.56	9.72	10.16	9.68	10.67	11.10	11.12	4.39E+03	2.15E+03	1.65E+03	4.92E+02	3.46E+02	3.91E+02	5.63E+02	3.29E+02	NA	9.96E+03	1.61E+03	1.84E+03	1.93E+03	1.87E+03	3.35E+03	1.13E+03	3.03E+03	6.83E+03
216	P60866	RS20	HUMAN	*RPS20*	9.68	11.41	9.47	10.42	8.05	10.86	10.14	11.62	10.41	13.66	10.41	10.88	10.29	10.63	9.86	8.74	9.33	11.94	8.21E+02	8.45E+03	2.07E+03	4.16E+02	8.21E+01	5.35E+02	3.49E+02	8.60E+02	NA	2.98E+04	1.04E+03	2.56E+03	3.12E+03	2.58E+03	4.44E+03	3.67E+02	9.74E+02	1.24E+04
217	P06748	NPM	HUMAN	*NPM1*	10.49	12.69	11.72	11.34	11.43	9.51	10.07	10.58	11.43	12.61	11.58	11.41	12.78	10.67	10.42	11.46	11.77	13.75	1.35E+03	2.11E+04	1.40E+04	7.57E+02	NA	2.34E+02	3.36E+02	4.39E+02	NA	1.42E+04	2.22E+03	4.17E+03	2.01E+04	2.66E+03	8.75E+03	1.89E+03	4.74E+03	4.50E+04
218	O75643	U520	HUMAN	*SNRNP200*	10.23	8.93	9.65	11.25	11.63	9.60	10.88	10.54	11.03	10.38	9.66	10.69	9.30	9.28	9.79	10.23	11.18	9.38	1.14E+03	1.22E+03	2.48E+03	7.14E+02	6.91E+02	2.47E+02	5.59E+02	4.30E+02	4.00E+02	2.71E+03	6.58E+02	2.12E+03	1.30E+03	1.02E+03	3.96E+03	8.60E+02	3.20E+03	1.68E+03
219	Q14498	RBM39	HUMAN	*RBM39*	9.03	10.27	10.19	10.10	9.33	8.87	11.73	9.88	10.37	9.11	9.50	10.66	10.13	9.16	9.90	11.17	10.59	9.80	5.70E+02	3.65E+03	4.12E+03	3.42E+02	1.61E+02	1.65E+02	9.77E+02	2.87E+02	2.60E+02	8.99E+02	5.99E+02	2.06E+03	2.74E+03	9.34E+02	4.66E+03	1.56E+03	2.16E+03	2.43E+03
220	P68133	ACTS	HUMAN	*ACTA1*	13.22	11.95	12.60	9.63	12.06	9.65	12.64	9.81	12.06	17.17	10.74	11.15	12.02	15.53	12.80	10.65	12.14	14.81	8.24E+03	1.24E+04	2.67E+04	2.57E+02	9.21E+02	2.54E+02	1.81E+03	2.76E+02	NA	3.43E+05	1.29E+03	3.31E+03	1.16E+04	7.71E+04	6.50E+04	1.12E+03	6.09E+03	9.38E+04
221	P61247	RS3A	HUMAN	*RPS3A*	10.99	10.13	9.55	9.76	10.78	10.94	9.47	10.44	11.14	10.92	11.53	10.06	9.52	11.30	10.18	10.64	11.72	10.09	1.86E+03	3.26E+03	2.25E+03	2.78E+02	3.95E+02	5.65E+02	2.34E+02	4.03E+02	4.30E+02	4.11E+03	2.15E+03	9.86E+02	1.60E+03	4.10E+03	6.68E+03	1.11E+03	4.60E+03	3.08E+03
222	Q96E39	RMXL1	HUMAN	*RBMXL1*	10.79	10.12	12.27	12.88	10.91	10.47	10.45	10.88	10.88	12.55	10.64	10.36	10.25	11.52	9.47	11.96	12.78	12.22	1.64E+03	3.24E+03	2.10E+04	2.12E+03	4.30E+02	4.17E+02	4.25E+02	5.31E+02	NA	1.35E+04	1.21E+03	1.46E+03	3.03E+03	4.78E+03	2.24E+03	2.63E+03	9.41E+03	1.52E+04
223	P00403	COX2	HUMAN	*MT-CO2*	9.86	9.91	8.79	11.28	9.88	11.49	10.66	10.45	9.88	10.33	9.62	9.69	9.76	10.27	9.48	10.33	9.58	9.16	9.16E+02	2.75E+03	8.68E+02	7.25E+02	NA	8.06E+02	4.84E+02	4.04E+02	NA	2.62E+03	6.45E+02	5.29E+02	2.00E+03	2.01E+03	2.29E+03	9.12E+02	1.14E+03	1.37E+03
224	P84103	SRSF3	HUMAN	*SRSF3*	10.82	13.10	11.63	11.35	13.19	11.77	12.08	12.01	11.64	14.64	10.77	14.26	13.35	14.44	12.00	13.85	14.00	13.23	1.67E+03	2.82E+04	1.30E+04	7.63E+02	1.99E+03	9.72E+02	1.24E+03	1.11E+03	5.95E+02	5.92E+04	1.31E+03	3.66E+04	3.01E+04	3.61E+04	3.55E+04	9.46E+03	2.18E+04	3.11E+04
225	P40925	MDHC	HUMAN	*MDH1*	12.98	9.24	11.19	9.84	9.62	10.40	10.47	9.54	10.38	10.11	10.86	12.08	8.90	12.68	11.33	9.67	10.13	9.47	7.02E+03	1.59E+03	9.34E+03	2.92E+02	1.91E+02	4.00E+02	4.30E+02	2.35E+02	2.62E+02	2.18E+03	1.39E+03	7.25E+03	8.51E+02	1.07E+04	2.06E+04	6.14E+02	1.60E+03	1.83E+03
226	P12814	ACTN1	HUMAN	*ACTN1*	9.53	8.53	9.39	10.47	10.24	10.40	11.10	10.30	11.03	8.79	10.70	10.21	11.31	11.56	9.67	9.36	10.76	9.89	7.55E+02	8.34E+02	1.88E+03	4.30E+02	2.81E+02	4.00E+02	6.45E+02	3.70E+02	4.00E+02	6.41E+02	1.25E+03	1.22E+03	6.92E+03	4.92E+03	3.27E+03	5.13E+02	2.42E+03	2.61E+03
227	Q12931	TRAP1	HUMAN	*TRAP1*	10.23	9.54	12.88	12.14	13.39	12.84	11.85	11.43	10.77	12.23	10.46	10.45	11.41	9.75	12.10	10.21	10.88	11.66	1.15E+03	2.05E+03	3.26E+04	1.29E+03	2.28E+03	2.00E+03	1.06E+03	7.58E+02	3.37E+02	1.08E+04	1.07E+03	1.63E+03	7.42E+03	1.41E+03	3.82E+04	8.50E+02	2.61E+03	1.02E+04
228	O00159	MYO1C	HUMAN	*MYO1C*	12.23	9.76	8.74	9.86	9.86	9.95	10.47	9.86	11.14	8.28	9.86	9.55	10.49	8.30	10.65	9.76	11.33	8.15	4.22E+03	2.46E+03	8.05E+02	NA	NA	3.05E+02	4.30E+02	NA	4.30E+02	3.33E+02	7.43E+02	3.78E+02	3.66E+03	5.18E+02	1.11E+04	6.45E+02	3.53E+03	4.27E+02
229	P09525	ANXA4	HUMAN	*ANXA4*	11.14	12.19	8.74	10.65	10.65	12.04	11.48	10.27	12.43	8.44	8.92	9.68	9.24	9.69	10.40	9.96	10.84	9.87	2.05E+03	1.48E+04	8.11E+02	4.84E+02	3.64E+02	1.16E+03	8.29E+02	3.64E+02	1.02E+03	4.19E+02	4.35E+02	5.14E+02	1.22E+03	1.35E+03	8.58E+03	7.27E+02	2.55E+03	2.58E+03
230	Q9Y262	EIF3L	HUMAN	*EIF3L*	10.18	11.19	10.77	11.46	11.19	12.98	12.62	13.73	12.39	10.87	9.33	11.79	11.00	10.41	10.69	9.37	12.56	11.48	1.11E+03	7.20E+03	6.73E+03	8.21E+02	NA	2.19E+03	1.78E+03	3.56E+03	9.89E+02	3.98E+03	5.44E+02	5.76E+03	5.45E+03	2.21E+03	1.15E+04	5.17E+02	8.10E+03	8.91E+03
231	P21359	NF1	HUMAN	*NF1*	11.37	11.44	11.93	11.75	12.41	12.19	9.53	11.39	12.41	12.64	13.07	16.25	16.59	10.27	15.64	16.29	12.95	16.97	2.38E+03	8.67E+03	1.63E+04	9.90E+02	NA	1.28E+03	2.41E+02	7.40E+02	NA	1.45E+04	6.07E+03	1.49E+05	2.88E+05	2.01E+03	4.95E+05	5.10E+04	1.06E+04	4.22E+05
232	Q9UHX1	PUF60	HUMAN	*PUF60*	11.03	10.87	11.40	11.15	10.04	11.65	8.92	11.53	11.62	11.30	12.15	10.72	12.14	10.69	9.29	10.22	11.19	10.55	1.91E+03	5.72E+03	1.09E+04	6.67E+02	2.47E+02	8.95E+02	1.72E+02	8.13E+02	5.90E+02	5.48E+03	3.24E+03	2.18E+03	1.27E+04	2.70E+03	1.45E+03	8.52E+02	3.21E+03	4.44E+03
233	Q07020	RL18	HUMAN	*RPL18*	10.12	10.18	11.80	10.88	9.93	9.70	10.47	10.43	10.91	11.53	10.11	11.34	9.30	11.13	11.60	9.80	10.52	10.36	1.07E+03	3.41E+03	1.49E+04	5.61E+02	2.31E+02	2.62E+02	4.30E+02	4.00E+02	3.68E+02	6.50E+03	8.66E+02	3.94E+03	1.30E+03	3.65E+03	2.57E+04	6.61E+02	2.07E+03	3.83E+03
234	P37108	SRP14	HUMAN	*SRP14*	8.87	8.64	9.28	9.92	10.91	8.54	11.87	10.37	10.45	8.47	9.51	9.80	10.68	9.23	9.46	9.53	9.18	10.84	5.24E+02	9.23E+02	1.67E+03	3.06E+02	4.30E+02	1.40E+02	1.08E+03	3.86E+02	2.74E+02	4.33E+02	6.04E+02	6.52E+02	4.27E+03	9.81E+02	2.20E+03	5.65E+02	8.87E+02	5.51E+03
235	P21796	VDAC1	HUMAN	*VDAC1*	9.12	12.12	9.61	10.23	10.19	9.77	10.23	9.73	10.23	9.40	10.39	10.44	11.81	10.93	10.32	8.93	10.23	10.91	5.98E+02	1.40E+04	2.40E+03	NA	2.72E+02	2.72E+02	NA	2.63E+02	NA	1.18E+03	1.03E+03	1.62E+03	9.97E+03	3.17E+03	7.79E+03	4.04E+02	1.71E+03	5.81E+03
236	Q15233	NONO	HUMAN	*NONO*	10.14	13.23	12.32	11.44	10.63	10.63	9.08	10.19	10.89	11.26	10.28	10.48	10.63	11.24	9.93	12.87	9.91	11.03	1.08E+03	3.08E+04	2.18E+03	8.09E+02	NA	NA	4.77E+02	1.82E+03	[illegible]	2.33E+03	4.04E+03	1.79E+03	1.33E+03	3.65E+03	2.59E+03	7.14E+03	1.01E+04	1.86E+03
237	Q16401	PSMD5	HUMAN	*PSMD5*	9.25	9.21	9.31	10.47	11.21	9.30	10.47	8.29	11.03	10.36	[illegible]	10.32	10.34	8.79	9.38	9.56	9.01	9.09	6.41E+02	1.56E+03	1.73E+03	4.30E+02	5.24E+02	2.08E+02	4.30E+02	1.23E+03	4.00E+02	2.67E+03	4.60E+02	1.40E+03	3.25E+03	7.21E+02	1.83E+03	5.73E+02	8.05E+02	1.29E+03

238	Q14764	MVP	HUMAN	*MVP*	8.54	9.99	8.71	9.53	9.53	10.51	10.66	9.74	10.86	9.81	7.95	9.47	8.74	8.05	9.09	9.59	8.22	9.63	4.41E+02	2.94E+03	7.63E+02	NA	NA	4.30E+02	4.86E+02	2.65E+02	3.58E+02	1.70E+03	2.70E+02	3.01E+02	7.05E+02	4.36E+02	6.72E+02	5.85E+02	5.16E+02	2.10E+03
239	Q8IVL1	NAV2	HUMAN	*NAV2*	11.15	10.33	11.44	11.76	11.44	11.44	10.63	11.43	11.44	12.62	13.51	11.94	10.77	12.23	9.78	12.45	11.64	9.53	2.06E+03	3.82E+03	1.13E+04	1.00E+03	NA	NA	4.75E+02	7.58E+02	NA	1.42E+04	8.19E+03	6.48E+03	4.56E+03	7.80E+03	3.92E+03	3.65E+03	4.36E+03	1.93E+03
240	Q9NQC3	RTN4	HUMAN	*RTN4*	10.94	12.43	14.05	11.07	11.05	10.91	11.20	9.41	11.05	14.63	9.77	10.51	11.84	11.33	11.04	10.57	10.71	11.91	1.80E+03	1.76E+04	7.48E+04	6.33E+02	NA	5.51E+02	6.91E+02	2.18E+02	NA	5.89E+04	7.05E+02	1.74E+03	1.02E+04	4.18E+03	1.59E+04	1.06E+03	2.34E+03	1.22E+04
241	P62917	RL8	HUMAN	*RPL8*	12.21	13.36	13.72	10.95	11.68	9.90	11.68	11.51	11.68	11.71	11.68	11.34	10.90	11.97	11.61	12.23	11.44	15.45	4.16E+03	3.37E+04	5.92E+04	5.87E+02	NA	2.96E+02	NA	8.02E+02	NA	7.41E+03	2.38E+03	3.93E+03	5.08E+03	6.55E+03	2.59E+04	3.15E+03	3.80E+03	1.47E+05
242	P35268	RL22	HUMAN	*RPL22*	11.09	9.81	12.88	12.52	10.58	11.25	12.49	11.98	11.25	12.45	9.46	10.62	10.08	11.67	9.72	11.68	13.48	10.60	1.98E+03	2.54E+03	3.25E+04	1.67E+03	3.48E+02	6.91E+02	1.64E+03	1.09E+03	NA	1.26E+04	5.87E+02	1.96E+03	2.63E+03	5.32E+03	3.59E+03	2.18E+03	1.52E+04	4.59E+03
243	Q9NPP4	NLRC4	HUMAN	*NLRC4*	11.88	11.25	13.58	13.29	12.99	9.59	11.12	11.83	12.99	15.41	13.20	13.46	10.81	13.09	15.48	11.31	12.88	14.19	3.34E+03	7.52E+03	5.35E+04	2.82E+03	NA	2.46E+02	6.54E+02	9.85E+02	NA	1.01E+05	6.64E+03	2.06E+04	4.73E+03	1.42E+04	4.42E+05	1.71E+03	1.01E+04	6.11E+04
244	P49721	PSB2	HUMAN	*PSMB2*	12.15	11.86	9.09	10.54	9.95	11.48	11.00	10.54	10.70	11.28	10.42	10.05	10.21	9.55	11.63	9.67	12.03	9.36	4.00E+03	1.17E+04	1.32E+03	NA	2.34E+02	8.00E+02	6.03E+02	4.30E+02	3.22E+02	5.42E+03	1.05E+03	9.80E+02	2.94E+03	1.22E+03	2.63E+04	6.14E+02	5.66E+03	1.66E+03
245	O95373	IPO7	HUMAN	*IPO7*	10.42	8.96	9.01	10.47	10.63	10.95	10.47	10.43	10.92	10.38	9.62	9.99	9.46	10.23	12.29	9.61	10.90	11.62	1.29E+03	1.25E+03	1.19E+03	4.30E+02	3.59E+02	5.68E+02	4.31E+02	4.00E+02	3.70E+02	2.71E+03	6.46E+02	8.95E+02	1.52E+03	1.95E+03	4.42E+04	5.93E+02	2.66E+03	9.89E+03
246	P34931	HS71L	HUMAN	*HSPA1L*	9.47	10.51	11.11	10.47	10.80	11.78	9.54	10.13	10.24	11.70	8.57	10.14	8.53	10.24	12.06	9.57	8.67	11.98	7.30E+02	4.37E+03	8.79E+03	4.30E+02	4.00E+02	9.75E+02	2.44E+02	3.33E+02	NA	7.38E+03	3.62E+02	1.11E+03	5.34E+02	1.97E+03	3.70E+04	5.77E+02	6.59E+02	1.28E+04
247	Q71UI9	H2AV	HUMAN	*H2AZ2*	9.02	8.71	9.26	9.60	10.80	10.40	10.17	11.11	12.22	8.86	9.47	9.75	10.37	9.10	9.42	9.54	9.36	8.87	5.68E+02	9.87E+02	1.63E+03	2.52E+02	4.00E+02	4.00E+02	3.57E+02	6.18E+02	8.79E+02	6.94E+02	5.91E+02	5.90E+02	3.32E+03	8.94E+02	2.02E+03	5.68E+02	9.94E+02	1.03E+03
248	Q99729	ROAA	HUMAN	*HNRNPAB*	9.88	9.07	9.29	11.49	11.03	11.08	11.49	12.28	11.46	8.79	11.23	9.66	9.06	9.85	9.53	9.42	10.60	8.53	9.27E+02	1.38E+03	1.70E+03	8.33E+02	4.66E+02	6.18E+02	8.33E+02	1.34E+03	5.28E+02	6.44E+02	1.77E+03	4.96E+02	1.02E+03	1.51E+03	2.56E+03	5.31E+02	2.17E+03	7.07E+02
249	Q9UKN8	TF3C4	HUMAN	*GTF3C4*	11.29	9.52	9.77	12.17	11.04	10.10	10.38	12.18	10.22	9.54	10.87	10.47	9.84	10.48	9.42	11.29	10.72	10.40	2.26E+03	2.02E+03	2.81E+03	1.31E+03	4.69E+02	3.33E+02	4.06E+02	1.25E+03	2.38E+02	1.34E+03	1.40E+03	1.67E+03	2.14E+03	2.34E+03	2.04E+03	1.68E+03	2.35E+03	3.93E+03
250	P18084	ITB5	HUMAN	*ITGB5*	11.12	8.78	10.55	10.47	10.91	11.15	11.10	11.18	11.87	10.75	10.55	9.60	11.03	8.76	11.32	10.97	10.62	10.86	2.02E+03	1.06E+03	5.59E+03	4.30E+02	4.30E+02	6.45E+02	6.45E+02	6.45E+02	6.96E+02	3.62E+03	1.14E+03	4.33E+02	5.60E+03	7.08E+02	2.04E+04	1.37E+03	2.20E+03	5.61E+03
251	Q8IWJ2	GCC2	HUMAN	*GCC2*	12.38	10.80	12.21	10.10	11.91	10.56	11.64	11.51	12.15	12.15	11.32	12.06	12.02	10.69	11.78	12.15	12.22	10.81	4.67E+03	5.43E+03	2.01E+04	3.41E+02	8.31E+02	4.41E+02	9.22E+02	8.02E+02	8.42E+02	1.02E+04	1.87E+03	7.13E+03	1.17E+04	2.69E+03	2.97E+04	2.99E+03	6.42E+03	5.42E+03
252	Q9Y512	SAM50	HUMAN	*SAMM50*	10.74	11.21	10.52	10.95	11.02	10.16	9.56	11.18	11.02	11.21	12.13	10.85	12.30	9.67	12.88	11.53	10.94	12.07	1.58E+03	7.32E+03	5.45E+03	5.87E+02	4.63E+02	3.45E+02	2.47E+02	6.45E+02	NA	5.12E+03	3.21E+03	2.49E+03	1.43E+04	1.33E+03	6.92E+04	1.98E+03	2.72E+03	1.36E+04
253	Q9H0S4	DDX47	HUMAN	*DDX47*	9.52	9.94	9.18	10.35	10.71	10.23	9.41	10.00	11.03	9.93	9.93	11.24	12.00	9.58	9.36	10.12	8.26	9.24	7.52E+02	2.82E+03	1.49E+03	4.00E+02	3.77E+02	3.61E+02	2.26E+02	3.08E+02	4.00E+02	1.88E+03	7.76E+02	3.58E+03	1.15E+04	1.25E+03	1.76E+03	8.04E+02	5.27E+02	1.48E+03
254	Q92599	Sep-08	HUMAN	*SEPTIN8*	8.04	8.69	8.93	9.69	9.69	9.69	9.69	9.69	10.54	9.94	10.08	9.92	8.34	10.07	9.63	9.82	9.69	9.20	3.48E+02	9.74E+02	1.07E+03	NA	NA	NA	NA	NA	2.92E+02	1.90E+03	8.51E+02	8.01E+02	3.98E+02	1.76E+03	3.07E+03	6.70E+02	1.21E+03	1.43E+03
255	Q10713	MPPA	HUMAN	*PMPCA*	8.06	8.71	8.61	9.36	10.80	10.21	10.47	9.59	9.86	8.68	9.36	9.72	8.63	9.22	9.26	11.73	9.50	8.45	3.51E+02	9.95E+02	6.45E+02	NA	4.00E+02	3.56E+02	4.30E+02	2.42E+02	1.91E+02	5.65E+02	5.55E+02	5.65E+02	6.15E+02	9.77E+02	1.30E+03	2.26E+03	1.08E+03	6.45E+02
256	P21980	TGM2	HUMAN	*TGM2*	12.26	9.20	9.17	10.12	10.02	9.46	10.35	10.02	11.03	9.07	9.73	9.91	9.22	12.50	12.69	9.48	10.96	12.66	4.29E+03	1.55E+03	1.46E+03	3.47E+02	2.44E+02	2.28E+02	4.00E+02	NA	4.00E+02	8.58E+02	6.86E+02	7.83E+02	1.20E+03	9.41E+03	6.01E+04	5.50E+02	2.76E+03	2.08E+04
257	Q9Y2Z9	COQ6	HUMAN	*COQ6*	11.68	12.26	13.10	10.49	10.89	10.68	10.46	11.73	11.73	11.78	10.87	17.82	11.95	12.26	11.57	11.57	13.93	12.32	2.93E+03	1.55E+04	3.81E+04	4.37E+02	4.23E+02	4.77E+02	4.27E+02	NA	NA	7.81E+03	1.40E+03	4.46E+05	1.11E+04	8.01E+03	2.51E+04	2.03E+03	2.08E+04	1.64E+04
258	Q9P1U1	ARP3B	HUMAN	*ACTR3B*	9.04	12.03	8.93	11.54	10.30	12.18	10.53	10.81	11.41	9.83	9.73	10.49	12.64	12.55	10.01	9.29	9.51	9.34	5.73E+02	1.32E+04	1.07E+03	8.63E+02	2.91E+02	1.27E+03	4.48E+02	5.09E+02	5.14E+02	1.73E+03	6.87E+02	1.70E+03	1.82E+04	9.76E+03	5.39E+03	4.93E+02	1.08E+03	1.63E+03
259	Q99460	PSMD1	HUMAN	*PSMD1*	10.62	8.78	8.98	10.21	10.08	10.43	9.82	9.73	10.48	11.26	11.02	9.84	9.99	12.67	9.26	8.89	12.67	8.59	1.47E+03	1.06E+03	1.15E+03	3.66E+02	2.54E+02	4.07E+02	2.87E+02	2.63E+02	2.79E+02	5.33E+03	1.53E+03	6.95E+02	2.43E+03	1.06E+04	1.33E+03	3.98E+02	8.77E+03	7.59E+02
260	Q96Q89	KI20B	HUMAN	*KIF20B*	9.75	9.53	10.18	11.31	10.08	9.87	10.20	11.01	10.18	10.44	10.60	10.67	9.92	8.61	9.88	10.26	11.20	10.00	8.59E+02	2.03E+03	4.07E+03	7.43E+02	2.53E+02	2.90E+02	3.62E+02	5.77E+02	NA	2.85E+03	1.17E+03	2.07E+03	2.30E+03	6.38E+02	4.55E+03	8.77E+02	3.23E+03	2.85E+03
261	Q8N715	CC185	HUMAN	*CCDC185*	12.08	11.17	11.13	12.07	13.00	11.70	11.49	12.41	11.95	12.12	11.95	11.65	9.33	12.05	11.09	11.92	12.92	12.60	3.82E+03	7.08E+03	8.93E+03	1.23E+03	1.75E+03	9.24E+02	8.37E+02	1.46E+03	NA	9.98E+03	2.84E+03	5.14E+03	1.34E+03	6.92E+03	1.67E+04	2.56E+03	1.04E+04	1.99E+04
262	Q5SQ64	LY66F	HUMAN	*LY6G6F*	12.22	11.40	11.36	11.21	11.36	11.10	10.90	10.77	11.36	11.87	11.76	12.35	11.36	10.73	10.76	11.51	11.37	12.24	4.20E+03	8.37E+03	1.07E+04	6.94E+02	NA	6.25E+02	5.68E+02	4.96E+02	NA	8.35E+03	2.51E+03	8.97E+03	7.17E+03	2.76E+03	1.23E+04	1.96E+03	3.62E+03	1.54E+04
263	Q4G148	GXLT1	HUMAN	*GXYLT1*	10.98	14.48	11.82	10.78	11.63	10.23	11.63	11.03	10.53	12.88	11.08	11.26	13.93	13.10	11.83	11.44	14.14	13.43	1.84E+03	7.37E+04	1.50E+04	5.25E+02	NA	3.59E+02	NA	5.87E+02	2.88E+02	1.71E+04	1.60E+03	3.67E+03	4.52E+04	1.43E+04	3.09E+04	1.86E+03	2.41E+04	3.59E+04
264	Q16630	CPSF6	HUMAN	*CPSF6*	10.55	8.34	10.24	10.47	10.73	10.28	10.12	11.51	10.56	8.13	10.24	9.47	9.51	7.03	10.55	8.09	9.47	10.33	1.40E+03	6.85E+02	4.32E+03	4.30E+02	3.82E+02	3.70E+02	3.46E+02	8.00E+02	2.95E+02	2.61E+02	NA	3.00E+02	1.59E+03	2.16E+02	9.98E+03	2.68E+02	1.06E+03	3.73E+03
265	Q15102	PA1B3	HUMAN	*PAFAH1B3*	10.74	12.45	10.98	10.59	9.95	10.48	9.36	10.30	10.59	11.87	10.85	10.80	11.94	9.58	10.32	10.66	10.51	9.41	1.58E+03	1.78E+04	7.90E+03	NA	2.34E+02	4.20E+02	2.20E+02	3.70E+02	NA	8.32E+03	1.38E+03	2.37E+03	1.10E+04	1.25E+03	7.79E+03	1.12E+03	2.05E+03	1.74E+03
266	Q14978	NOLC1	HUMAN	*NOLC1*	11.81	11.46	12.37	10.99	10.97	11.31	9.92	10.97	10.58	8.41	8.28	9.57	10.95	12.89	11.58	9.84	11.37	10.69	3.19E+03	8.78E+03	2.26E+04	6.01E+02	NA	7.17E+02	3.06E+02	NA	2.98E+02	4.00E+02	3.13E+02	4.00E+02	5.26E+03	1.24E+04	2.54E+04	6.77E+02	3.64E+03	4.91E+03
267	Q13148	TADBP	HUMAN	*TARDBP*	8.46	8.48	9.40	11.02	13.29	11.72	10.42	11.85	9.86	9.07	8.44	10.04	9.50	8.13	9.42	9.02	8.65	10.04	4.24E+02	7.95E+02	1.91E+03	6.11E+02	2.12E+03	9.38E+02	4.16E+02	1.00E+03	1.90E+02	8.60E+02	3.38E+02	9.69E+02	1.57E+03	4.58E+02	2.01E+03	4.25E+02	6.53E+02	2.96E+03
268	Q02790	FKBP4	HUMAN	*FKBP4*	11.60	10.82	11.02	11.02	11.58	8.86	9.83	8.78	10.39	11.63	11.58	11.10	11.65	10.97	11.49	11.15	10.82	9.13	2.77E+03	5.49E+03	8.21E+03	NA	6.69E+02	1.64E+02	2.90E+02	1.56E+02	2.64E+02	6.97E+03	2.22E+03	3.17E+03	8.89E+03	3.28E+03	2.34E+04	1.54E+03	2.52E+03	1.33E+03
269	P67809	YBOX1	HUMAN	*YBX1*	8.69	9.83	11.03	10.47	9.74	10.26	10.23	9.81	9.74	12.94	8.05	9.66	8.96	8.62	9.54	8.98	10.70	9.11	4.76E+02	2.59E+03	8.28E+03	4.30E+02	NA	3.66E+02	3.69E+02	2.76E+02	NA	1.80E+04	2.82E+02	4.97E+02	9.13E+02	6.45E+02	2.63E+03	4.15E+02	2.33E+03	1.31E+03
270	P63173	RL38	HUMAN	*RPL38*	10.02	8.20	9.81	7.58	9.47	10.46	9.47	9.47	9.93	9.68	8.51	10.15	9.24	9.35	9.22	9.71	9.47	9.24	1.01E+03	5.89E+02	2.92E+03	9.03E+01	NA	4.16E+02	NA	NA	1.99E+02	1.52E+03	3.50E+02	1.13E+03	1.23E+03	1.07E+03	1.17E+03	6.29E+02	1.06E+03	1.48E+03
271	P46783	RS10	HUMAN	*RPS10*	9.42	12.79	9.33	9.85	10.77	9.85	9.85	9.15	10.53	9.82	9.85	12.65	13.58	9.22	14.17	9.59	12.45	9.56	7.07E+02	2.25E+04	1.78E+03	NA	3.94E+02	NA	NA	1.90E+02	2.89E+02	1.71E+03	7.38E+02	1.13E+04	3.54E+04	9.72E+02	1.75E+05	5.86E+02	7.53E+03	1.99E+03
272	P42285	MTREX	HUMAN	*MTREX*	9.40	8.73	11.81	9.83	10.91	9.94	11.62	11.56	9.94	9.49	9.36	10.07	10.05	11.76	9.33	10.89	9.64	9.35	7.01E+02	1.01E+03	1.49E+04	2.90E+02	4.30E+02	NA	9.11E+02	8.28E+02	NA	1.28E+03	5.52E+02	1.00E+03	2.55E+03	5.64E+03	1.64E+03	1.30E+03	1.18E+03	1.64E+03
273	P31153	METK2	HUMAN	*MAT2A*	11.21	11.98	14.07	9.77	11.31	12.57	10.51	11.61	11.34	10.13	11.65	10.92	9.08	10.68	11.34	11.52	11.58	11.47	2.15E+03	1.27E+04	7.57E+04	2.80E+02	5.58E+02	1.66E+03	4.40E+02	8.55E+02	NA	2.22E+03	2.32E+03	2.67E+03	1.03E+03	2.67E+03	2.06E+04	1.96E+03	4.18E+03	8.82E+03
274	P05386	RLA1	HUMAN	*RPLP1*	10.14	13.22	10.88	9.96	10.30	10.25	11.37	10.30	10.30	8.68	10.82	9.99	11.54	12.11	11.14	10.30	9.72	9.36	1.08E+03	3.06E+04	7.33E+03	3.13E+02	NA	3.65E+02	7.73E+02	NA	NA	5.62E+02	1.36E+03	8.96E+02	8.21E+03	7.22E+03	1.73E+04	8.97E+02	1.24E+03	1.66E+03
275	O00629	IMA3	HUMAN	*KPNA4*	9.44	11.22	9.29	11.41	11.47	11.08	10.15	11.02	11.00	10.51	10.16	10.59	10.60	11.41	10.97	10.50	9.80	9.68	7.15E+02	7.38E+03	1.69E+03	7.90E+02	6.21E+02	6.15E+02	3.53E+02	5.82E+02	3.91E+02	3.01E+03	8.93E+02	1.90E+03	4.00E+03	4.42E+03	1.49E+04	1.02E+03	1.30E+03	2.20E+03
276	P52701	MSH6	HUMAN	*MSH6*	11.14	10.45	10.75	11.75	12.66	9.89	10.98	9.44	11.24	11.28	10.13	11.43	8.90	9.87	10.38	13.39	10.44	10.32	2.05E+03	4.16E+03	6.57E+03	9.94E+02	1.38E+03	2.93E+02	5.96E+02	2.22E+02	4.59E+02	5.39E+03	8.76E+02	4.25E+03	8.50E+02	1.53E+03	8.36E+03	6.91E+03	1.96E+03	3.72E+03
277	P27797	CALR	HUMAN	*CALR*	10.23	9.91	10.18	12.07	12.33	11.92	10.34	10.89	11.65	12.18	11.54	12.14	9.91	10.72	11.65	14.20	12.18	12.24	1.15E+03	2.75E+03	4.07E+03	1.23E+03	1.10E+03	1.07E+03	3.96E+02	5.34E+02	NA	1.04E+04	2.16E+03	7.65E+03	2.27E+03	2.75E+03	2.68E+04	1.21E+04	6.26E+03	1.55E+04
278	P62314	SMD1	HUMAN	*SNRPD1*	9.33	8.65	10.90	10.35	10.10	9.32	11.03	9.32	11.09	8.76	9.01	9.15	11.90	9.29	9.23	9.46	9.10	8.81	6.73E+02	9.38E+02	7.43E+03	4.00E+02	2.57E+02	NA	6.15E+02	2.08E+02	4.15E+02	6.22E+02	4.56E+02	3.00E+01	1.07E+04	1.02E+03	1.18E+03	5.42E+02	8.50E+02	9.71E+02
279	Q99733	NP1L4	HUMAN	*NAP1L4*	11.50	9.30	9.40	9.55	10.28	12.16	10.86	8.51	11.14	9.85	11.69	10.37	9.29	10.88	9.62	9.08	10.23	9.37	2.60E+03	1.68E+03	1.92E+03	2.45E+02	2.88E+02	1.26E+03	5.52E+02	1.37E+02	4.30E+02	1.76E+03	2.38E+03	1.48E+03	1.28E+03	3.07E+03	3.00E+03	4.39E+02	1.71E+03	1.68E+03
280	Q12906	ILF3	HUMAN	*ILF3*	10.89	9.82	11.83	10.17	10.29	10.70	10.11	10.59	10.29	9.55	11.34	10.27	9.00	10.31	9.79	12.48	10.19	12.47	1.75E+03	2.58E+03	1.51E+04	3.56E+02	NA	4.83E+02	3.44E+02	4.43E+02	NA	1.35E+03	1.90E+03	1.31E+03	9.54E+02	2.07E+03	4.00E+03	3.73E+03	1.67E+03	1.82E+04
281	P49902	5NTC	HUMAN	*NT5C2*	10.60	9.68	9.32	13.57	12.54	11.29	10.99	11.44	11.14	9.58	11.67	10.01	11.65	9.88	9.35	12.07	11.40	10.49	1.45E+03	2.29E+03	1.76E+03	3.40E+03	1.27E+03	7.07E+02	6.00E+02	7.65E+02	4.30E+02	1.40E+03	2.36E+03	9.22E+02	8.91E+03	1.53E+03	1.73E+03	2.83E+03	3.70E+03	4.23E+03
282	P49588	SYAC	HUMAN	*AARS1*	11.16	9.26	9.50	10.87	10.32	10.60	11.95	10.44	12.03	9.62	11.57	10.08	11.90	11.39	9.95	10.61	10.64	9.34	2.07E+03	1.63E+03	2.13E+03	5.54E+02	2.95E+02	4.54E+02	1.13E+03	4.02E+02	7.72E+02	1.44E+03	2.21E+03	1.02E+03	1.07E+04	4.36E+03	5.01E+03	1.09E+03	2.24E+03	1.63E+03
283	Q32P51	RA1L2	HUMAN	*HNRNPA1L2*	8.56	9.37	9.25	10.89	10.04	9.98	10.00	10.47	11.03	8.78	11.05	9.77	9.61	10.20	9.68	10.34	8.69	9.37	4.46E+02	1.77E+03	1.61E+03	5.62E+02	2.47E+02	3.10E+02	3.22E+02	4.09E+02	4.00E+02	6.34E+02	1.57E+03	6.15E+02	1.74E+03	1.92E+03	3.34E+03	9.20E+02	6.67E+02	1.67E+03
284	Q00005	2ABB	HUMAN	*PPP2R2B*	10.96	9.33	9.53	10.87	11.18	10.55	10.85	10.14	11.11	9.63	10.24	10.95	9.62	10.52	10.09	9.46	9.54	8.43	1.82E+03	1.72E+03	2.20E+03	5.56E+02	5.14E+02	4.39E+02	5.50E+02	3.35E+02	4.20E+02	1.45E+03	9.34E+02	2.74E+03	1.75E+03	2.40E+03	6.00E+03	5.44E+02	1.10E+03	6.28E+02
285	P51991	ROA3	HUMAN	*HNRNPA3*	11.47	10.24	10.69	9.38	10.73	12.01	12.14	10.77	10.73	9.88	12.22	10.46	9.51	10.78	9.39	12.20	12.07	10.18	2.54E+03	3.56E+03	6.27E+03	2.22E+02	NA	1.14E+03	1.29E+03	4.96E+02	NA	1.81E+03	3.40E+03	1.65E+03	1.58E+03	2.87E+03	1.87E+03	3.08E+03	5.81E+03	3.32E+03
286	P51665	PSMD7	HUMAN	*PSMD7*	11.84	9.47	10.31	11.35	11.62	10.35	8.40	11.08	9.26	9.96	10.53	9.95	8.28	8.45	9.49	11.83	10.99	8.15	3.26E+03	1.93E+03	4.55E+03	7.63E+02	6.86E+02	3.89E+02	1.31E+02	6.04E+02	1.34E+02	1.94E+03	1.12E+03	8.45E+02	3.63E+02	5.74E+02	2.34E+03	2.41E+03	2.81E+03	4.30E+02

Table S3. Reactome pathway enrichment analysis of the significantly differentially expressed proteins of ALA-A2 treated A549 cells.

Pathway identifier	Pathway name	#Entities found	#Entities total	Entities ratio	Entities pValue	Entities FDR	#Reactions found	#Reactions total	Reactions ratio	Species identifier	Species name	Submitted entities found
**R-HSA-9613829**	**Chaperone Mediated Autophagy**	**4**	**23**	**0.002**	**1.46E-06**	**1.11E-03**	**15**	**19**	**0.0014**	**9606**	**Homo sapiens**	**HSPA8;HSP90AA1;HSP90AB1;UBA52**
R-HSA-2262752	Cellular responses to stress	13	952	0.067	1.75E-05	5.41E-03	55	381	0.0281	9606	Homo sapiens	HSPA8;HSP90AA1;HSP90AB1;TUBB;PRDX1;H2AC19;RPSA;H4C1;UBA52;LRPPRC;RPL6;HSP90B1
R-HSA-8953897	Cellular responses to stimuli	13	970	0.068	2.14E-05	5.41E-03	55	412	0.0303	9606	Homo sapiens	HSPA8;HSP90AA1;HSP90AB1;TUBB;PRDX1;H2AC19;RPSA;H4C1;UBA52;LRPPRC;RPL6;HSP90B1
R-HSA-1266738	Developmental Biology	14	1263	0.089	7.81E-05	1.11E-02	66	556	0.0410	9606	Homo sapiens	HSPA8;HSP90AA1;HSP90AB1;JUP;TUBB;ANXA4;H2AC19;RPSA;RPL6;ACTG1;KRT19;KRT18;H4C1;UBA52
R-HSA-449147	Signaling by Interleukins	10	643	0.045	6.73E-05	1.11E-02	17	493	0.0363	9606	Homo sapiens	HSPA8;HSP90AA1;HNRNPA2B1;TALDO1;UBA52;HSP90B1
R-HSA-9013418	RHOBTB2 GTPase cycle	3	24	0.002	8.79E-05	1.11E-02	2	2	0.0001	9606	Homo sapiens	HSP90AA1;HSP90AB1;ACTG1
R-HSA-422475	Axon guidance	9	585	0.041	1.78E-04	1.22E-02	18	298	0.0219	9606	Homo sapiens	HSPA8;HSP90AA1;HSP90AB1;TUBB;ANXA4;RPSA;UBA52;RPL6;ACTG1
R-HSA-9675108	Nervous system development	9	621	0.044	2.77E-04	1.22E-02	18	324	0.0239	9606	Homo sapiens	HSPA8;HSP90AA1;HSP90AB1;TUBB;ANXA4;RPSA;UBA52;RPL6;ACTG1
**R-HSA-9612973**	**Autophagy**	**5**	**166**	**0.012**	**2.92E-04**	**1.22E-02**	**37**	**108**	**0.0080**	**9606**	**Homo sapiens**	**HSPA8;HSP90AA1;HSP90AB1;TUBB;UBA52**
R-HSA-9663891	Selective autophagy	4	89	0.006	2.77E-04	1.22E-02	20	48	0.0035	9606	Homo sapiens	HSPA8;HSP90AA1;TUBB;UBA52
R-HSA-3371497	HSP90 chaperone cycle for steroid hormone receptors (SHR) in th	4	72	0.005	1.24E-04	1.22E-02	16	16	0.0012	9606	Homo sapiens	HSPA8;HSP90AA1;HSP90AB1;TUBB
R-HSA-8852276	The role of GTSE1 in G2/M progression after G2 checkpoint	4	83	0.006	2.13E-04	1.22E-02	5	10	0.0007	9606	Homo sapiens	HSP90AA1;HSP90AB1;TUBB;UBA52
R-HSA-2565942	Regulation of PLK1 Activity at G2/M Transition	4	92	0.006	3.14E-04	1.22E-02	5	12	0.0009	9606	Homo sapiens	HSP90AA1;TUBB;UBA52
R-HSA-8950505	Gene and protein expression by JAK-STAT signaling after Interleu	4	73	0.005	1.31E-04	1.22E-02	2	36	0.0027	9606	Homo sapiens	HNRNPA2B1;TALDO1
R-HSA-9020591	Interleukin-12 signaling	4	84	0.006	2.23E-04	1.22E-02	2	56	0.0041	9606	Homo sapiens	HNRNPA2B1;TALDO1
R-HSA-389958	Cooperation of Prefoldin and TriC/CCT in actin and tubulin folding	3	37	0.003	3.12E-04	1.22E-02	6	6	0.0004	9606	Homo sapiens	CCT3;TUBB;CCT4
R-HSA-389957	Prefoldin mediated transfer of substrate to CCT/TriC	3	29	0.002	1.53E-04	1.22E-02	2	2	0.0001	9606	Homo sapiens	CCT3;TUBB;CCT4
R-HSA-389960	Formation of tubulin folding intermediates by CCT/TriC	3	30	0.002	1.69E-04	1.22E-02	2	2	0.0001	9606	Homo sapiens	CCT3;TUBB;CCT4
R-HSA-9706574	RHOBTB GTPase Cycle	3	36	0.003	2.88E-04	1.22E-02	2	4	0.0003	9606	Homo sapiens	HSP90AA1;HSP90AB1;ACTG1
R-HSA-168273	Influenza Viral RNA Transcription and Replication	5	176	0.012	3.81E-04	1.29E-02	3	14	0.0010	9606	Homo sapiens	HSP90AA1;RPSA;UBA52;RPL6;IPO5
R-HSA-390466	Chaperonin-mediated protein folding	4	96	0.007	3.68E-04	1.29E-02	16	19	0.0014	9606	Homo sapiens	CCT3;TUBB;GAPDH;CCT4
R-HSA-447115	Interleukin-12 family signaling	4	96	0.007	3.68E-04	1.29E-02	2	114	0.0084	9606	Homo sapiens	HNRNPA2B1;TALDO1
R-HSA-390471	Association of TriC/CCT with target proteins during biosynthesis	3	40	0.003	3.91E-04	1.29E-02	2	2	0.0001	9606	Homo sapiens	CCT3;GAPDH;CCT4
R-HSA-391251	Protein folding	4	102	0.007	4.62E-04	1.39E-02	22	28	0.0021	9606	Homo sapiens	CCT3;TUBB;GAPDH;CCT4
R-HSA-156842	Eukaryotic Translation Elongation	4	102	0.007	4.62E-04	1.39E-02	6	9	0.0007	9606	Homo sapiens	EEF1G;RPSA;UBA52;RPL6
R-HSA-70171	Glycolysis	4	110	0.008	6.13E-04	1.56E-02	2	24	0.0018	9606	Homo sapiens	PKM;GAPDH
R-HSA-9646399	Aggrephagy	3	47	0.003	6.24E-04	1.56E-02	13	15	0.0011	9606	Homo sapiens	HSP90AA1;TUBB;UBA52
R-HSA-3371568	Attenuation phase	3	47	0.003	6.24E-04	1.56E-02	4	5	0.0004	9606	Homo sapiens	HSPA8;HSP90AA1;HSP90AB1
R-HSA-163754	Insulin effects increased synthesis of Xylulose-5-Phosphate	2	10	0.001	5.88E-04	1.56E-02	3	3	0.0002	9606	Homo sapiens	TALDO1;TKT
R-HSA-5336415	Uptake and function of diphtheria toxin	2	10	0.001	5.88E-04	1.56E-02	1	5	0.0004	9606	Homo sapiens	HSP90AA1;HSP90AB1
R-HSA-168255	Influenza Infection	5	200	0.014	6.78E-04	1.63E-02	3	59	0.0043	9606	Homo sapiens	HSP90AA1;RPSA;UBA52;RPL6;IPO5
R-HSA-69275	G2/M Transition	5	212	0.015	8.79E-04	1.92E-02	22	78	0.0057	9606	Homo sapiens	HSP90AA1;HSP90AB1;TUBB;UBA52
R-HSA-453274	Mitotic G2-G2/M phases	5	214	0.015	9.17E-04	1.92E-02	22	80	0.0059	9606	Homo sapiens	HSP90AA1;HSP90AB1;TUBB;UBA52
R-HSA-5617833	Cilium Assembly	5	210	0.015	8.43E-04	1.92E-02	17	50	0.0037	9606	Homo sapiens	CCT3;HSP90AA1;TUBB;CCT4
R-HSA-6785807	Interleukin-4 and Interleukin-13 signaling	5	211	0.015	8.61E-04	1.92E-02	3	47	0.0035	9606	Homo sapiens	HSPA8;HSP90AA1;HSP90B1
R-HSA-71387	Metabolism of carbohydrates	7	456	0.032	0.001016305	2.03E-02	11	243	0.0179	9606	Homo sapiens	PKM;TALDO1;TKT;UBA52;GAPDH
R-HSA-390450	Folding of actin by CCT/TriC	2	13	0.001	9.87E-04	2.03E-02	2	2	0.0001	9606	Homo sapiens	CCT3;CCT4
R-HSA-192905	vRNP Assembly	2	14	0.001	0.001142541	2.17E-02	1	2	0.0001	9606	Homo sapiens	HSP90AA1;IPO5
R-HSA-3371571	HSF1-dependent transactivation	3	59	0.004	0.001197748	2.28E-02	5	8	0.0006	9606	Homo sapiens	HSPA8;HSP90AA1;HSP90AB1
R-HSA-381183	ATF6 (ATF6-alpha) activates chaperone genes	2	15	0.001	0.001308662	2.36E-02	1	5	0.0004	9606	Homo sapiens	HSP90B1
R-HSA-70326	Glucose metabolism	4	140	0.010	0.001488626	2.68E-02	3	50	0.0037	9606	Homo sapiens	PKM;GAPDH
R-HSA-381033	ATF6 (ATF6-alpha) activates chaperones	2	17	0.001	0.00167341	3.01E-02	1	10	0.0007	9606	Homo sapiens	HSP90B1
R-HSA-5663205	Infectious disease	12	1357	0.095	0.002114039	3.17E-02	55	752	0.0554	9606	Homo sapiens	HSP90AA1;HSP90AB1;TUBB;H2AC19;RPSA;H4C1;UBA52;RPL6;ACTG1;IPO5
**R-HSA-1632852**	**Macroautophagy**	**4**	**150**	**0.011**	**0.001912184**	**3.17E-02**	**20**	**87**	**0.0064**	**9606**	**Homo sapiens**	**HSPA8;HSP90AA1;TUBB;UBA52**
R-HSA-9018519	Estrogen-dependent gene expression	4	154	0.011	0.002102838	3.17E-02	15	66	0.0049	9606	Homo sapiens	HSP90AA1;HSP90AB1;H2AC19;H4C1
R-HSA-380284	Loss of proteins required for interphase microtubule organization	3	71	0.005	0.002026779	3.17E-02	3	3	0.0002	9606	Homo sapiens	HSP90AA1;TUBB
R-HSA-380259	Loss of Nlp from mitotic centrosomes	3	71	0.005	0.002026779	3.17E-02	2	2	0.0001	9606	Homo sapiens	HSP90AA1;TUBB
R-HSA-399954	Sema3A PAK dependent Axon repulsion	2	19	0.001	0.002081	3.17E-02	2	6	0.0004	9606	Homo sapiens	HSP90AA1;HSP90AB1
R-HSA-8854518	AURKA Activation by TPX2	3	74	0.005	0.0022779	3.42E-02	2	2	0.0001	9606	Homo sapiens	HSP90AA1;TUBB
R-HSA-380287	Centrosome maturation	3	83	0.006	0.003144167	4.40E-02	6	6	0.0004	9606	Homo sapiens	HSP90AA1;TUBB
R-HSA-380270	Recruitment of mitotic centrosome proteins and complexes	3	81	0.006	0.002936647	4.40E-02	3	3	0.0002	9606	Homo sapiens	HSP90AA1;TUBB
R-HSA-68886	M Phase	6	416	0.029	0.003246889	4.55E-02	22	91	0.0067	9606	Homo sapiens	HSP90AA1;TUBB;H2AC19;H4C1;UBA52
R-HSA-977225	Amyloid fiber formation	3	89	0.006	0.003820042	4.88E-02	6	33	0.0024	9606	Homo sapiens	H2AC19;H4C1;UBA52
R-HSA-2559582	Senescence-Associated Secretory Phenotype (SASP)	3	91	0.006	0.004063488	4.88E-02	3	22	0.0016	9606	Homo sapiens	H2AC19;H4C1;UBA52
R-HSA-9645723	Diseases of programmed cell death	3	90	0.006	0.003940615	4.88E-02	2	28	0.0021	9606	Homo sapiens	PRDX1;H2AC19;H4C1
R-HSA-9679191	Potential therapeutics for SARS	3	91	0.006	0.004063488	4.88E-02	2	34	0.0025	9606	Homo sapiens	HSP90AA1;HSP90AB1;TUBB
R-HSA-5637815	Signaling by Ligand-Responsive EGFR Variants in Cancer	2	26	0.002	0.003836455	4.88E-02	23	23	0.0017	9606	Homo sapiens	HSP90AA1;UBA52
R-HSA-1236382	Constitutive Signaling by Ligand-Responsive EGFR Cancer Variant	2	26	0.002	0.003836455	4.88E-02	23	23	0.0017	9606	Homo sapiens	HSP90AA1;UBA52
R-HSA-5620922	BBSome-mediated cargo-targeting to cilium	2	25	0.002	0.003554934	4.88E-02	1	5	0.0004	9606	Homo sapiens	CCT3;CCT4
R-HSA-1280215	Cytokine Signaling in Immune system	10	1092	0.077	0.004120042	4.94E-02	25	708	0.0521	9606	Homo sapiens	HSPA8;HSP90AA1;HNRNPA2B1;TALDO1;UBA52;HSP90B1
R-HSA-69278	Cell Cycle, Mitotic	7	596	0.042	0.004567039	5.48E-02	67	350	0.0258	9606	Homo sapiens	HSP90AA1;HSP90AB1;TUBB;H2AC19;H4C1;UBA52
R-HSA-5620912	Anchoring of the basal body to the plasma membrane	3	99	0.007	0.005130885	5.64E-02	8	9	0.0007	9606	Homo sapiens	HSP90AA1;TUBB
R-HSA-156902	Peptide chain elongation	3	97	0.007	0.004849792	5.64E-02	4	5	0.0004	9606	Homo sapiens	RPSA;UBA52;RPL6
R-HSA-380320	Recruitment of NuMA to mitotic centrosomes	3	97	0.007	0.004849792	5.64E-02	2	2	0.0001	9606	Homo sapiens	HSP90AA1;TUBB
R-HSA-1643685	Disease	16	2431	0.170	0.007105283	5.76E-02	164	1632	0.1202	9606	Homo sapiens	HSP90AA1;HSP90AB1;TUBB;H2AC19;TALDO1;RPSA;RPL6;ACTG1;IPO5;EEF1G;PRDX1;NF1;H4C1;UBA52
R-HSA-6798695	Neutrophil degranulation	6	480	0.034	0.006471569	5.76E-02	4	10	0.0007	9606	Homo sapiens	HSPA8;HSP90AA1;PKM;HSP90AB1;JUP;TUBB
R-HSA-72766	Translation	5	339	0.024	0.006597343	5.76E-02	27	99	0.0073	9606	Homo sapiens	EEF1G;RPSA;UBA52;TARS1;RPL6
R-HSA-1852241	Organelle biogenesis and maintenance	5	338	0.024	0.006517566	5.76E-02	17	86	0.0063	9606	Homo sapiens	CCT3;HSP90AA1;TUBB;CCT4
R-HSA-9679506	SARS-CoV Infections	4	217	0.015	0.007059251	5.76E-02	4	256	0.0189	9606	Homo sapiens	HSP90AA1;HSP90AB1;TUBB;UBA52
R-HSA-168928	DDX58/IFIH1-mediated induction of interferon-alpha/beta	3	100	0.007	0.005275046	5.76E-02	7	53	0.0039	9606	Homo sapiens	HSP90AA1;HSP90AB1;UBA52
R-HSA-8939236	RUNX1 regulates transcription of genes involved in differentiatio	3	106	0.007	0.006191331	5.76E-02	5	15	0.0011	9606	Homo sapiens	H2AC19;H4C1;UBA52
R-HSA-68867	Assembly of the pre-replicative complex	3	102	0.007	0.005570655	5.76E-02	4	18	0.0013	9606	Homo sapiens	H2AC19;H4C1;UBA52
R-HSA-72764	Eukaryotic Translation Termination	3	106	0.007	0.006191331	5.76E-02	3	5	0.0004	9606	Homo sapiens	RPSA;UBA52;RPL6
R-HSA-72689	Formation of a pool of free 40S subunits	3	106	0.007	0.006191331	5.76E-02	2	2	0.0001	9606	Homo sapiens	RPSA;UBA52;RPL6
R-HSA-2408557	Selenocysteine synthesis	3	112	0.008	0.007197229	5.76E-02	2	7	0.0005	9606	Homo sapiens	RPSA;UBA52;RPL6

R-HSA-975956	Nonsense Mediated Decay (NMD) independent of the Exon Juncti	3	101	0.007	0.005421632	5.76E-02	1	1	0.0001	9606	Homo sapiens	RPSA;UBA52;RPL6
R-HSA-1643713	Signaling by EGFR in Cancer	2	32	0.002	0.005734288	5.76E-02	40	42	0.0031	9606	Homo sapiens	HSP90AA1;UBA52
R-HSA-5334118	DNA methylation	2	36	0.003	0.007193161	5.76E-02	7	7	0.0005	9606	Homo sapiens	H2AC19;H4C1
R-HSA-8863795	Downregulation of ERBB2 signaling	2	36	0.003	0.007193161	5.76E-02	7	14	0.0010	9606	Homo sapiens	HSP90AA1;UBA52
R-HSA-5689901	Metalloprotease DUBs	2	32	0.002	0.005734288	5.76E-02	5	6	0.0004	9606	Homo sapiens	H2AC19;UBA52
**R-HSA-9615710**	**Late endosomal microautophagy**	**2**	**35**	**0.002**	**0.006814229**	**5.76E-02**	**3**	**3**	**0.0002**	**9606**	**Homo sapiens**	**HSPA8;UBA52**
R-HSA-418990	Adherens junctions interactions	2	35	0.002	0.006814229	5.76E-02	3	16	0.0012	9606	Homo sapiens	JUP;ACTG1
R-HSA-171306	Packaging Of Telomere Ends	2	34	0.002	0.006444727	5.76E-02	2	2	0.0001	9606	Homo sapiens	H2AC19;H4C1
R-HSA-5626467	RHO GTPases activate IQGAPs	2	36	0.003	0.007193161	5.76E-02	2	5	0.0004	9606	Homo sapiens	TUBB;ACTG1
R-HSA-73728	RNA Polymerase I Promoter Opening	2	34	0.002	0.006444727	5.76E-02	1	2	0.0001	9606	Homo sapiens	H2AC19;H4C1
R-HSA-2559580	Oxidative Stress Induced Senescence	3	114	0.008	0.007552809	6.04E-02	6	40	0.0029	9606	Homo sapiens	H2AC19;H4C1;UBA52
R-HSA-192823	Viral mRNA Translation	3	114	0.008	0.007552809	6.04E-02	2	2	0.0001	9606	Homo sapiens	RPSA;UBA52;RPL6
R-HSA-1799339	SRP-dependent cotranslational protein targeting to membrane	3	119	0.008	0.008486799	6.08E-02	5	5	0.0004	9606	Homo sapiens	RPSA;UBA52;RPL6
R-HSA-9633012	Response of EIF2AK4 (GCN2) to amino acid deficiency	3	115	0.008	0.007734443	6.08E-02	4	16	0.0012	9606	Homo sapiens	RPSA;UBA52;RPL6
R-HSA-69002	DNA Replication Pre-Initiation	3	120	0.008	0.008681384	6.08E-02	4	27	0.0020	9606	Homo sapiens	H2AC19;H4C1;UBA52
R-HSA-72706	GTP hydrolysis and joining of the 60S ribosomal subunit	3	120	0.008	0.008681384	6.08E-02	3	3	0.0002	9606	Homo sapiens	RPSA;UBA52;RPL6
R-HSA-156827	L13a-mediated translational silencing of Ceruloplasmin expressic	3	120	0.008	0.008681384	6.08E-02	2	3	0.0002	9606	Homo sapiens	RPSA;UBA52;RPL6
R-HSA-211000	Gene Silencing by RNA	3	120	0.008	0.008681384	6.08E-02	2	40	0.0029	9606	Homo sapiens	HSP90AA1;H2AC19;H4C1
R-HSA-5675482	Regulation of necroptotic cell death	2	38	0.003	0.007979047	6.08E-02	5	18	0.0013	9606	Homo sapiens	HSP90AA1;UBA52
R-HSA-110330	Recognition and association of DNA glycosylase with site contain	2	39	0.003	0.008385866	6.08E-02	2	10	0.0007	9606	Homo sapiens	H2AC19;H4C1
R-HSA-68616	Assembly of the ORC complex at the origin of replication	2	40	0.003	0.008801846	6.16E-02	2	11	0.0008	9606	Homo sapiens	H2AC19;H4C1
R-HSA-6814122	Cooperation of PDCL (PhLP1) and TRiC/CCT in G-protein beta foldi	2	41	0.003	0.009226921	6.46E-02	8	11	0.0008	9606	Homo sapiens	CCT3;CCT4
R-HSA-4420097	VEGFA-VEGFR2 Pathway	3	127	0.009	0.01011709	6.46E-02	19	79	0.0058	9606	Homo sapiens	HSP90AA1;JUP;ACTG1
R-HSA-72613	Eukaryotic Translation Initiation	3	130	0.009	0.010772208	6.46E-02	12	21	0.0015	9606	Homo sapiens	RPSA;UBA52;RPL6
R-HSA-72737	Cap-dependent Translation Initiation	3	130	0.009	0.010772208	6.46E-02	10	18	0.0013	9606	Homo sapiens	RPSA;UBA52;RPL6
R-HSA-373760	L1CAM interactions	3	130	0.009	0.010772208	6.46E-02	8	54	0.0040	9606	Homo sapiens	HSPA8;TUBB;ACTG1
R-HSA-927802	Nonsense-Mediated Decay (NMD)	3	124	0.009	0.009485942	6.46E-02	5	6	0.0004	9606	Homo sapiens	RPSA;UBA52;RPL6
R-HSA-975957	Nonsense Mediated Decay (NMD) enhanced by the Exon Junction	3	124	0.009	0.009485942	6.46E-02	4	5	0.0004	9606	Homo sapiens	RPSA;UBA52;RPL6
R-HSA-5218920	VEGFR2 mediated vascular permeability	2	44	0.003	0.010556057	6.46E-02	7	15	0.0011	9606	Homo sapiens	HSP90AA1;JUP
R-HSA-212300	PRC2 methylates histones and DNA	2	44	0.003	0.010556057	6.46E-02	4	4	0.0003	9606	Homo sapiens	H2AC19;H4C1
R-HSA-3371511	HSF1 activation	2	43	0.003	0.010104092	6.46E-02	3	7	0.0005	9606	Homo sapiens	HSP90AA1;HSP90AB1
R-HSA-110328	Recognition and association of DNA glycosylase with site contain	2	42	0.003	0.009661025	6.46E-02	1	21	0.0015	9606	Homo sapiens	H2AC19;H4C1
R-HSA-6791055	TALDO1 deficiency: failed conversion of SH7P, GA3P to Fru(6)P, E	1	3	0.000	0.010456874	6.46E-02	1	1	0.0001	9606	Homo sapiens	TALDO1
R-HSA-6791462	TALDO1 deficiency: failed conversion of Fru(6)P, E4P to SH7P, GA	1	3	0.000	0.010456874	6.46E-02	1	1	0.0001	9606	Homo sapiens	TALDO1
R-HSA-427359	SIRT1 negatively regulates rRNA expression	2	45	0.003	0.011016855	6.61E-02	3	5	0.0004	9606	Homo sapiens	H2AC19;H4C1
R-HSA-110331	Cleavage of the damaged purine	2	45	0.003	0.011016855	6.61E-02	2	9	0.0007	9606	Homo sapiens	H2AC19;H4C1
R-HSA-194315	Signaling by Rho GTPases	7	709	0.050	0.011407215	6.84E-02	32	203	0.0150	9606	Homo sapiens	HSP90AA1;HSP90AB1;JUP;TUBB;H2AC19;H4C1;ACTG1
R-HSA-71336	Pentose phosphate pathway	2	46	0.003	0.011486421	6.89E-02	6	20	0.0015	9606	Homo sapiens	TALDO1;TKT
R-HSA-5213460	RIPK1-mediated regulated necrosis	2	46	0.003	0.011486421	6.89E-02	5	34	0.0025	9606	Homo sapiens	HSP90AA1;UBA52
R-HSA-73927	Depurination	2	46	0.003	0.011486421	6.89E-02	4	19	0.0014	9606	Homo sapiens	H2AC19;H4C1
R-HSA-1640170	Cell Cycle	7	734	0.051	0.013605581	6.96E-02	91	449	0.0331	9606	Homo sapiens	HSP90AA1;HSP90AB1;TUBB;H2AC19;H4C1;UBA52
R-HSA-9716542	Signaling by Rho GTPases, Miro GTPases and RHOBTB3	7	725	0.051	0.012781516	6.96E-02	32	212	0.0156	9606	Homo sapiens	HSP90AA1;HSP90AB1;JUP;TUBB;H2AC19;H4C1;ACTG1
R-HSA-8939211	ESR-mediated signaling	4	257	0.018	0.012545548	6.96E-02	20	111	0.0082	9606	Homo sapiens	HSP90AA1;HSP90AB1;H2AC19;H4C1
R-HSA-194138	Signaling by VEGF	3	138	0.010	0.012637618	6.96E-02	19	86	0.0063	9606	Homo sapiens	HSP90AA1;JUP;ACTG1
R-HSA-3371556	Cellular response to heat stress	3	135	0.009	0.011917791	6.96E-02	15	29	0.0021	9606	Homo sapiens	HSPA8;HSP90AA1;HSP90AB1
R-HSA-6809371	Formation of the cornified envelope	3	138	0.010	0.012637618	6.96E-02	9	27	0.0020	9606	Homo sapiens	KRT19;KRT18;JUP
R-HSA-5625886	Activated PKN1 stimulates transcription of AR (androgen recepto	2	49	0.003	0.012947085	6.96E-02	8	11	0.0008	9606	Homo sapiens	H2AC19;H4C1
R-HSA-9670095	Inhibition of DNA recombination at telomere	2	48	0.003	0.0124516	6.96E-02	3	4	0.0003	9606	Homo sapiens	H2AC19;H4C1
R-HSA-427389	ERCC6 (CSB) and EHMT2 (G9a) positively regulate rRNA expressio	2	48	0.003	0.0124516	6.96E-02	2	4	0.0003	9606	Homo sapiens	H2AC19;H4C1
R-HSA-9665230	Drug resistance in ERBB2 KD mutants	1	4	0.000	0.013918627	6.96E-02	8	8	0.0006	9606	Homo sapiens	HSP90AA1
R-HSA-9652282	Drug-mediated inhibition of ERBB2 signaling	1	4	0.000	0.013918627	6.96E-02	3	3	0.0002	9606	Homo sapiens	HSP90AA1
R-HSA-9665737	Drug resistance in ERBB2 TMD/JMD mutants	1	4	0.000	0.013918627	6.96E-02	1	1	0.0001	9606	Homo sapiens	HSP90AA1
R-HSA-9665246	Resistance of ERBB2 KD mutants to neratinib	1	4	0.000	0.013918627	6.96E-02	1	1	0.0001	9606	Homo sapiens	HSP90AA1
R-HSA-9665247	Resistance of ERBB2 KD mutants to osimertinib	1	4	0.000	0.013918627	6.96E-02	1	1	0.0001	9606	Homo sapiens	HSP90AA1
R-HSA-9665251	Resistance of ERBB2 KD mutants to lapatinib	1	4	0.000	0.013918627	6.96E-02	1	1	0.0001	9606	Homo sapiens	HSP90AA1
R-HSA-9665245	Resistance of ERBB2 KD mutants to tesevatinib	1	4	0.000	0.013918627	6.96E-02	1	1	0.0001	9606	Homo sapiens	HSP90AA1
R-HSA-9665233	Resistance of ERBB2 KD mutants to trastuzumab	1	4	0.000	0.013918627	6.96E-02	1	1	0.0001	9606	Homo sapiens	HSP90AA1
R-HSA-9665249	Resistance of ERBB2 KD mutants to afatinib	1	4	0.000	0.013918627	6.96E-02	1	1	0.0001	9606	Homo sapiens	HSP90AA1
R-HSA-9665244	Resistance of ERBB2 KD mutants to sapitinib	1	4	0.000	0.013918627	6.96E-02	1	1	0.0001	9606	Homo sapiens	HSP90AA1
R-HSA-9665250	Resistance of ERBB2 KD mutants to AEE788	1	4	0.000	0.013918627	6.96E-02	1	1	0.0001	9606	Homo sapiens	HSP90AA1
R-HSA-73928	Depyrimidination	2	51	0.004	0.013963528	6.98E-02	2	41	0.0030	9606	Homo sapiens	H2AC19;H4C1
R-HSA-110329	Cleavage of the damaged pyrimidine	2	51	0.004	0.013963528	6.98E-02	1	20	0.0015	9606	Homo sapiens	H2AC19;H4C1
R-HSA-190828	Gap junction trafficking	2	52	0.004	0.014484361	7.24E-02	7	20	0.0015	9606	Homo sapiens	TUBB;ACTG1
R-HSA-9710421	Defective pyroptosis	2	52	0.004	0.014484361	7.24E-02	1	3	0.0002	9606	Homo sapiens	H2AC19;H4C1
R-HSA-3214858	RMTs methylate histone arginines	2	53	0.004	0.015013518	7.51E-02	8	22	0.0016	9606	Homo sapiens	H2AC19;H4C1
R-HSA-168256	Immune System	16	2684	0.188	0.017983012	7.58E-02	108	1623	0.1195	9606	Homo sapiens	HSPA8;HSP90AA1;PKM;HSP90AB1;JUP;TUBB;HNRNPA2B1;TALDO1;UBA52;ACTG1;HSP90B1
R-HSA-2029482	Regulation of actin dynamics for phagocytic cup formation	3	158	0.011	0.018068446	7.58E-02	8	24	0.0018	9606	Homo sapiens	HSP90AA1;HSP90AB1;ACTG1
R-HSA-2299718	Condensation of Prophase Chromosomes	2	55	0.004	0.016096556	7.58E-02	8	10	0.0007	9606	Homo sapiens	H2AC19;H4C1
R-HSA-157858	Gap junction trafficking and regulation	2	56	0.004	0.016650313	7.58E-02	7	24	0.0018	9606	Homo sapiens	TUBB;ACTG1
R-HSA-437239	Recycling pathway of L1	2	55	0.004	0.016096556	7.58E-02	5	14	0.0010	9606	Homo sapiens	TUBB;ACTG1
R-HSA-912446	Meiotic recombination	2	58	0.004	0.017781999	7.58E-02	4	9	0.0007	9606	Homo sapiens	H2AC19;H4C1
R-HSA-774815	Nucleosome assembly	2	54	0.004	0.015550937	7.58E-02	3	4	0.0003	9606	Homo sapiens	H2AC19;H4C1
R-HSA-606279	Deposition of new CENPA-containing nucleosomes at the centron	2	54	0.004	0.015550937	7.58E-02	3	4	0.0003	9606	Homo sapiens	H2AC19;H4C1
R-HSA-450408	AUF1 (hnRNP D0) binds and destabilizes mRNA	2	56	0.004	0.016650313	7.58E-02	2	4	0.0003	9606	Homo sapiens	HSPA8;UBA52
R-HSA-5620920	Cargo trafficking to the periciliary membrane	2	57	0.004	0.017212148	7.58E-02	1	27	0.0020	9606	Homo sapiens	CCT3;CCT4
R-HSA-5339562	Uptake and actions of bacterial toxins	2	60	0.004	0.018945508	7.58E-02	1	66	0.0049	9606	Homo sapiens	HSP90AA1;HSP90AB1
R-HSA-8856828	Clathrin-mediated endocytosis	3	161	0.011	0.018978862	7.59E-02	16	35	0.0026	9606	Homo sapiens	HSPA8;UBA52;ACTG1

R-HSA-1221632	Meiotic synapsis	2	62	0.004	0.02014036	8.06E-02	4	6	0.0004	9606	Homo sapiens	H2AC19;H4C1
R-HSA-5250924	B-WICH complex positively regulates rRNA expression	2	62	0.004	0.02014036	8.06E-02	1	3	0.0002	9606	Homo sapiens	H2AC19;H4C1
R-HSA-73929	Base-Excision Repair, AP Site Formation	2	63	0.004	0.020749391	8.30E-02	6	64	0.0047	9606	Homo sapiens	H2AC19;H4C1
R-HSA-3214815	HDACs deacetylate histones	2	63	0.004	0.020749391	8.30E-02	4	5	0.0004	9606	Homo sapiens	H2AC19;H4C1
R-HSA-73772	RNA Polymerase I Promoter Escape	2	64	0.004	0.021366079	8.55E-02	1	2	0.0001	9606	Homo sapiens	H2AC19;H4C1
R-HSA-70263	Gluconeogenesis	2	66	0.005	0.022622194	9.05E-02	1	26	0.0019	9606	Homo sapiens	GAPDH
R-HSA-69306	DNA Replication	3	174	0.012	0.023215314	9.29E-02	8	53	0.0039	9606	Homo sapiens	H2AC19;H4C1;UBA52
R-HSA-5693565	Recruitment and ATM-mediated phosphorylation of repair and si	2	67	0.005	0.023261504	9.30E-02	30	39	0.0029	9606	Homo sapiens	H4C1;UBA52
R-HSA-421270	Cell-cell junction organization	2	67	0.005	0.023261504	9.30E-02	3	21	0.0015	9606	Homo sapiens	JUP;ACTG1
R-HSA-201722	Formation of the beta-catenin:TCF transactivating complex	2	67	0.005	0.023261504	9.30E-02	2	13	0.0010	9606	Homo sapiens	H2AC19;H4C1
R-HSA-1227986	Signaling by ERBB2	2	68	0.005	0.023908238	9.56E-02	12	46	0.0034	9606	Homo sapiens	HSP90AA1;UBA52
R-HSA-9711097	Cellular response to starvation	3	176	0.012	0.023909171	9.56E-02	4	28	0.0021	9606	Homo sapiens	RPSA;UBA52;RPL6
R-HSA-5663202	Diseases of signal transduction by growth factor receptors and se	5	498	0.035	0.029868403	9.67E-02	101	484	0.0356	9606	Homo sapiens	EEF1G;HSP90AA1;NF1;UBA52;ACTG1
R-HSA-195258	RHO GTPase Effectors	4	326	0.023	0.027274544	9.67E-02	23	113	0.0083	9606	Homo sapiens	TUBB;H2AC19;H4C1;ACTG1
R-HSA-72163	mRNA Splicing - Major Pathway	3	185	0.013	0.027170485	9.67E-02	8	9	0.0007	9606	Homo sapiens	HSPA8;HNRNPA2B1;SRSF3
R-HSA-2029480	Fcgamma receptor (FCGR) dependent phagocytosis	3	193	0.014	0.030259999	9.67E-02	8	42	0.0031	9606	Homo sapiens	HSP90AA1;HSP90AB1;ACTG1
R-HSA-72172	mRNA Splicing	3	196	0.014	0.031464666	9.67E-02	8	14	0.0010	9606	Homo sapiens	HSPA8;HNRNPA2B1;SRSF3
R-HSA-9010553	Regulation of expression of SLITs and ROBOs	3	183	0.013	0.026426108	9.67E-02	5	20	0.0015	9606	Homo sapiens	RPSA;UBA52;RPL6
R-HSA-2408522	Selenoamino acid metabolism	3	180	0.013	0.02533058	9.67E-02	2	33	0.0024	9606	Homo sapiens	RPSA;UBA52;RPL6
R-HSA-6791226	Major pathway of rRNA processing in the nucleolus and cytosol	3	189	0.013	0.028692861	9.67E-02	2	7	0.0005	9606	Homo sapiens	RPSA;UBA52;RPL6
R-HSA-5693606	DNA Double Strand Break Response	2	75	0.005	0.02863847	9.67E-02	30	48	0.0035	9606	Homo sapiens	H4C1;UBA52
R-HSA-8936459	RUNX1 regulates genes involved in megakaryocyte differentiatio	2	78	0.005	0.03077142	9.67E-02	20	33	0.0024	9606	Homo sapiens	H2AC19;H4C1
R-HSA-168643	Nucleotide-binding domain, leucine rich repeat containing recept	2	71	0.005	0.025892415	9.67E-02	14	46	0.0034	9606	Homo sapiens	HSP90AB1;UBA52
R-HSA-5625740	RHO GTPases activate PKNs	2	80	0.006	0.032227517	9.67E-02	8	20	0.0015	9606	Homo sapiens	H2AC19;H4C1
R-HSA-5658442	Regulation of RAS by GAPs	2	71	0.005	0.025892415	9.67E-02	5	5	0.0004	9606	Homo sapiens	NF1;UBA52
R-HSA-5218859	Regulated Necrosis	2	77	0.005	0.030053556	9.67E-02	5	57	0.0042	9606	Homo sapiens	HSP90AA1;UBA52
R-HSA-427413	NoRC negatively regulates rRNA expression	2	80	0.006	0.032227517	9.67E-02	3	7	0.0005	9606	Homo sapiens	H2AC19;H4C1
R-HSA-5250913	Positive epigenetic regulation of rRNA expression	2	80	0.006	0.032227517	9.67E-02	3	7	0.0005	9606	Homo sapiens	H2AC19;H4C1
R-HSA-373755	Semaphorin interactions	2	71	0.005	0.025892415	9.67E-02	2	41	0.0030	9606	Homo sapiens	HSP90AA1;HSP90AB1
R-HSA-2559586	DNA Damage/Telomere Stress Induced Senescence	2	71	0.005	0.025892415	9.67E-02	1	18	0.0013	9606	Homo sapiens	H2AC19;H4C1
R-HSA-9616222	Transcriptional regulation of granulopoiesis	2	71	0.005	0.025892415	9.67E-02	1	27	0.0020	9606	Homo sapiens	H2AC19;H4C1
R-HSA-8937144	Aryl hydrocarbon receptor signalling	1	8	0.001	0.027647347	9.67E-02	3	5	0.0004	9606	Homo sapiens	HSP90AB1
R-HSA-6791465	Pentose phosphate pathway disease	1	8	0.001	0.027647347	9.67E-02	2	4	0.0003	9606	Homo sapiens	TALDO1
R-HSA-9706377	FLT3 signaling by CBL mutants	1	7	0.000	0.024232831	9.67E-02	1	1	0.0001	9606	Homo sapiens	UBA52
R-HSA-9694493	Maturation of protein E	1	8	0.001	0.027647347	9.67E-02	1	5	0.0004	9606	Homo sapiens	UBA52
R-HSA-9683683	Maturation of protein E	1	8	0.001	0.027647347	9.67E-02	1	5	0.0004	9606	Homo sapiens	UBA52
R-HSA-6802953	RAS signaling downstream of NF1 loss-of-function variants	1	9	0.001	0.031050153	9.67E-02	1	1	0.0001	9606	Homo sapiens	NF1
R-HSA-1445148	Translocation of SLC2A4 (GLUT4) to the plasma membrane	2	81	0.006	0.032965642	9.89E-02	3	15	0.0011	9606	Homo sapiens	TUBB;ACTG1
R-HSA-5663084	Diseases of carbohydrate metabolism	2	81	0.006	0.032965642	9.89E-02	3	44	0.0032	9606	Homo sapiens	TALDO1;UBA52
R-HSA-5578749	Transcriptional regulation by small RNAs	2	81	0.006	0.032965642	9.89E-02	1	5	0.0004	9606	Homo sapiens	H2AC19;H4C1
R-HSA-2559583	Cellular Senescence	3	200	0.014	0.033109871	9.93E-02	10	90	0.0066	9606	Homo sapiens	H2AC19;H4C1;UBA52
R-HSA-8948747	Regulation of PTEN localization	1	10	0.001	0.034441287	1.03E-01	2	4	0.0003	9606	Homo sapiens	UBA52
R-HSA-447041	CHL1 interactions	1	10	0.001	0.034441287	1.03E-01	1	5	0.0004	9606	Homo sapiens	HSPA8
R-HSA-8868773	rRNA processing in the nucleus and cytosol	3	207	0.015	0.03609576	1.08E-01	2	15	0.0011	9606	Homo sapiens	RPSA;UBA52;RPL6
R-HSA-392499	Metabolism of proteins	13	2205	0.155	0.037222073	1.09E-01	157	798	0.0588	9606	Homo sapiens	CCT3;HSPA8;TUBB;H2AC19;RPSA;RPL6;HSP90B1;EEF1G;H4C1;UBA52;TARS1;GAPDH;CCT4
R-HSA-168249	Innate Immune System	9	1334	0.093	0.040443628	1.09E-01	80	710	0.0523	9606	Homo sapiens	HSPA8;HSP90AA1;PKM;HSP90AB1;JUP;TUBB;UBA52;ACTG1;HSP90B1
R-HSA-9006931	Signaling by Nuclear Receptors	4	387	0.027	0.046452616	1.09E-01	20	192	0.0141	9606	Homo sapiens	HSP90AA1;HSP90AB1;H2AC19;H4C1
R-HSA-6805567	Keratinization	3	226	0.016	0.04487783	1.09E-01	16	34	0.0025	9606	Homo sapiens	KRT19;KRT18;JUP
R-HSA-201681	TCF dependent signaling in response to WNT	3	216	0.015	0.040133082	1.09E-01	15	71	0.0052	9606	Homo sapiens	H2AC19;H4C1;UBA52
R-HSA-68877	Mitotic Prometaphase	3	211	0.015	0.037862696	1.09E-01	9	20	0.0015	9606	Homo sapiens	HSP90AA1;TUBB
R-HSA-9609690	HCMV Early Events	3	215	0.015	0.039673537	1.09E-01	7	12	0.0009	9606	Homo sapiens	TUBB;H2AC19;H4C1
R-HSA-9711123	Cellular response to chemical stress	3	212	0.015	0.038311299	1.09E-01	5	71	0.0052	9606	Homo sapiens	PRDX1;UBA52;LRPPRC
R-HSA-376176	Signaling by ROBO receptors	3	235	0.016	0.049377788	1.09E-01	5	60	0.0044	9606	Homo sapiens	RPSA;UBA52;RPL6
R-HSA-72312	rRNA processing	3	245	0.017	0.054628601	1.09E-01	2	21	0.0015	9606	Homo sapiens	RPSA;UBA52;RPL6
R-HSA-6802957	Oncogenic MAPK signaling	2	93	0.007	0.042326409	1.09E-01	25	46	0.0034	9606	Homo sapiens	NF1;ACTG1
R-HSA-1500620	Meiosis	2	92	0.006	0.041512004	1.09E-01	8	15	0.0011	9606	Homo sapiens	H2AC19;H4C1
R-HSA-5250941	Negative epigenetic regulation of rRNA expression	2	89	0.006	0.039105429	1.09E-01	6	12	0.0009	9606	Homo sapiens	H2AC19;H4C1
R-HSA-73884	Base Excision Repair	2	100	0.007	0.048193067	1.09E-01	6	107	0.0079	9606	Homo sapiens	H2AC19;H4C1
R-HSA-446728	Cell junction organization	2	94	0.007	0.043146836	1.09E-01	4	37	0.0027	9606	Homo sapiens	JUP;ACTG1
R-HSA-5689603	UCH proteinases	2	98	0.007	0.046487763	1.09E-01	4	11	0.0008	9606	Homo sapiens	H2AC19;UBA52
R-HSA-1912408	Pre-NOTCH Transcription and Translation	2	89	0.006	0.039105429	1.09E-01	3	28	0.0021	9606	Homo sapiens	H2AC19;H4C1
R-HSA-73854	RNA Polymerase I Promoter Clearance	2	87	0.006	0.037532089	1.09E-01	2	10	0.0007	9606	Homo sapiens	H2AC19;H4C1
R-HSA-73864	RNA Polymerase I Transcription	2	89	0.006	0.039105429	1.09E-01	2	14	0.0010	9606	Homo sapiens	H2AC19;H4C1
R-HSA-5693607	Processing of DNA double-strand break ends	2	90	0.006	0.039901464	1.09E-01	2	17	0.0013	9606	Homo sapiens	H4C1;UBA52
R-HSA-450531	Regulation of mRNA stability by proteins that bind AU-rich eleme	2	93	0.007	0.042326409	1.09E-01	2	26	0.0019	9606	Homo sapiens	HSPA8;UBA52
R-HSA-6804756	Regulation of TP53 Activity through Phosphorylation	2	95	0.007	0.043973234	1.09E-01	2	26	0.0019	9606	Homo sapiens	UBA52
R-HSA-9613354	Lipophagy	1	12	0.001	0.0411887	1.09E-01	5	5	0.0004	9606	Homo sapiens	HSPA8
R-HSA-9014325	TICAM1,TRAF6-dependent induction of TAK1 complex	1	13	0.001	0.044545056	1.09E-01	5	6	0.0004	9606	Homo sapiens	UBA52
R-HSA-1358803	Downregulation of ERBB2:ERBB3 signaling	1	16	0.001	0.054545191	1.09E-01	5	8	0.0006	9606	Homo sapiens	UBA52
R-HSA-190873	Gap junction degradation	1	12	0.001	0.0411887	1.09E-01	4	4	0.0003	9606	Homo sapiens	ACTG1
R-HSA-937042	IRAK2 mediated activation of TAK1 complex	1	12	0.001	0.0411887	1.09E-01	4	5	0.0004	9606	Homo sapiens	UBA52
R-HSA-9645460	Alpha-protein kinase 1 signaling pathway	1	14	0.001	0.047889898	1.09E-01	4	10	0.0007	9606	Homo sapiens	UBA52
R-HSA-9634285	Constitutive Signaling by Overexpressed ERBB2	1	16	0.001	0.054545191	1.09E-01	4	8	0.0006	9606	Homo sapiens	HSP90AA1
R-HSA-5689877	Josephin domain DUBs	1	13	0.001	0.044545056	1.09E-01	3	6	0.0004	9606	Homo sapiens	UBA52
R-HSA-196025	Formation of annular gap junctions	1	11	0.001	0.03782079	1.09E-01	2	2	0.0001	9606	Homo sapiens	ACTG1
R-HSA-1253288	Downregulation of ERBB4 signaling	1	11	0.001	0.03782079	1.09E-01	2	5	0.0004	9606	Homo sapiens	UBA52
R-HSA-9013973	TICAM1-dependent activation of IRF3/IRF7	1	14	0.001	0.047889898	1.09E-01	2	9	0.0007	9606	Homo sapiens	UBA52

R-HSA-3000484	Scavenging by Class F Receptors	1	16	0.001	0.054545191	1.09E-01	2	2	0.0001	9606	Homo sapiens	HSP90AA1
R-HSA-2691232	Constitutive Signaling by NOTCH1 HD Domain Mutants	1	16	0.001	0.054545191	1.09E-01	2	6	0.0004	9606	Homo sapiens	UBA52
R-HSA-2691230	Signaling by NOTCH1 HD Domain Mutants in Cancer	1	16	0.001	0.054545191	1.09E-01	2	6	0.0004	9606	Homo sapiens	UBA52
R-HSA-1679131	Trafficking and processing of endosomal TLR	1	16	0.001	0.054545191	1.09E-01	2	7	0.0005	9606	Homo sapiens	HSP90B1
R-HSA-3785653	Myoclonic epilepsy of Lafora	1	11	0.001	0.03782079	1.09E-01	1	2	0.0001	9606	Homo sapiens	UBA52
R-HSA-9637628	Modulation by Mtb of host immune system	1	11	0.001	0.03782079	1.09E-01	1	6	0.0004	9606	Homo sapiens	UBA52
R-HSA-8849469	PTK6 Regulates RTKs and Their Effectors AKT1 and DOK1	1	11	0.001	0.03782079	1.09E-01	1	8	0.0006	9606	Homo sapiens	UBA52
R-HSA-9664873	Pexophagy	1	13	0.001	0.044545056	1.09E-01	1	13	0.0010	9606	Homo sapiens	UBA52
R-HSA-8876493	InlA-mediated entry of Listeria monocytogenes into host cells	1	14	0.001	0.047889898	1.09E-01	1	5	0.0004	9606	Homo sapiens	UBA52
R-HSA-209560	NF-kB is activated and signals survival	1	15	0.001	0.051223263	1.09E-01	1	4	0.0003	9606	Homo sapiens	UBA52
R-HSA-937039	IRAK1 recruits IKK complex	1	16	0.001	0.054545191	1.09E-01	1	4	0.0003	9606	Homo sapiens	UBA52
R-HSA-975144	IRAK1 recruits IKK complex upon TLR7/8 or 9 stimulation	1	16	0.001	0.054545191	1.09E-01	1	4	0.0003	9606	Homo sapiens	UBA52
R-HSA-209543	p75NTR recruits signalling complexes	1	16	0.001	0.054545191	1.09E-01	1	9	0.0007	9606	Homo sapiens	UBA52
R-HSA-5653656	Vesicle-mediated transport	6	827	0.058	0.068026701	1.09E-01	42	252	0.0186	9606	Homo sapiens	HSPA8;HSP90AA1;TUBB;UBA52;ACTG1;HSP90B1
R-HSA-8953854	Metabolism of RNA	6	783	0.055	0.05497913	1.09E-01	23	187	0.0138	9606	Homo sapiens	HSPA8;HNRNPA2B1;SRSF3;RPSA;UBA52;RPL6
R-HSA-3700989	Transcriptional Regulation by TP53	4	487	0.034	0.09071775	1.09E-01	14	259	0.0191	9606	Homo sapiens	PRDX1;UBA52;LRPPRC
R-HSA-9012999	RHO GTPase cycle	4	460	0.032	0.077236969	1.09E-01	9	91	0.0067	9606	Homo sapiens	HSP90AA1;HSP90AB1;JUP;ACTG1
R-HSA-8878171	Transcriptional regulation by RUNX1	3	261	0.018	0.063566034	1.09E-01	25	132	0.0097	9606	Homo sapiens	H2AC19;H4C1;UBA52
R-HSA-157118	Signaling by NOTCH	3	259	0.018	0.062413425	1.09E-01	15	154	0.0113	9606	Homo sapiens	H2AC19;H4C1;UBA52
R-HSA-5673001	RAF/MAP kinase cascade	3	322	0.023	0.10324824	1.09E-01	15	75	0.0055	9606	Homo sapiens	NF1;UBA52;ACTG1
R-HSA-5684996	MAPK1/MAPK3 signaling	3	329	0.023	0.108321056	1.09E-01	15	82	0.0060	9606	Homo sapiens	NF1;UBA52;ACTG1
R-HSA-72203	Processing of Capped Intron-Containing Pre-mRNA	3	256	0.018	0.060703323	1.09E-01	14	32	0.0024	9606	Homo sapiens	HSPA8;HNRNPA2B1;SRSF3
R-HSA-9609646	HCMV Infection	3	270	0.019	0.068875456	1.09E-01	8	31	0.0023	9606	Homo sapiens	TUBB;H2AC19;H4C1
R-HSA-5619507	Activation of HOX genes during differentiation	2	116	0.008	0.062620964	1.09E-01	31	43	0.0032	9606	Homo sapiens	H2AC19;H4C1
R-HSA-5617472	Activation of anterior HOX genes in hindbrain development during	2	116	0.008	0.062620964	1.09E-01	31	43	0.0032	9606	Homo sapiens	H2AC19;H4C1
R-HSA-212165	Epigenetic regulation of gene expression	2	139	0.010	0.085530318	1.09E-01	20	34	0.0025	9606	Homo sapiens	H2AC19;H4C1
R-HSA-3214847	HATs acetylate histones	2	110	0.008	0.057052502	1.09E-01	10	15	0.0011	9606	Homo sapiens	H2AC19;H4C1
R-HSA-5663220	RHO GTPases Activate Formins	2	149	0.010	0.096175197	1.09E-01	9	27	0.0020	9606	Homo sapiens	TUBB;ACTG1
R-HSA-1474165	Reproduction	2	123	0.009	0.069341288	1.09E-01	8	24	0.0018	9606	Homo sapiens	H2AC19;H4C1
R-HSA-68875	Mitotic Prophase	2	135	0.009	0.081381357	1.09E-01	8	34	0.0025	9606	Homo sapiens	H2AC19;H4C1
R-HSA-73886	Chromosome Maintenance	2	138	0.010	0.084487025	1.09E-01	8	38	0.0028	9606	Homo sapiens	H2AC19;H4C1
R-HSA-157579	Telomere Maintenance	2	111	0.008	0.057967835	1.09E-01	5	34	0.0025	9606	Homo sapiens	H2AC19;H4C1
R-HSA-5610787	Hedgehog 'off' state	2	124	0.009	0.070320187	1.09E-01	5	32	0.0024	9606	Homo sapiens	TUBB;UBA52
R-HSA-5628897	TP53 Regulates Metabolic Genes	2	126	0.009	0.072291692	1.09E-01	4	34	0.0025	9606	Homo sapiens	PRDX1;LRPPRC
R-HSA-1500931	Cell-Cell communication	2	133	0.009	0.079331488	1.09E-01	4	60	0.0044	9606	Homo sapiens	JUP;ACTG1
R-HSA-9707564	Cytoprotection by HMOX1	2	158	0.011	0.106063517	1.09E-01	4	37	0.0027	9606	Homo sapiens	UBA52;LRPPRC
R-HSA-1912422	Pre-NOTCH Expression and Processing	2	114	0.008	0.060744602	1.09E-01	3	38	0.0028	9606	Homo sapiens	H2AC19;H4C1
R-HSA-5693567	HDR through Homologous Recombination (HRR) or Single Strand	2	124	0.009	0.070320187	1.09E-01	3	42	0.0031	9606	Homo sapiens	H4C1;UBA52
R-HSA-5693538	Homology Directed Repair	2	130	0.009	0.076288356	1.09E-01	3	50	0.0037	9606	Homo sapiens	H4C1;UBA52
R-HSA-163685	Integration of energy metabolism	2	145	0.010	0.091872081	1.09E-01	3	62	0.0046	9606	Homo sapiens	TALDO1;TKT
R-HSA-69481	G2/M Checkpoints	2	154	0.011	0.101634541	1.09E-01	3	24	0.0018	9606	Homo sapiens	H4C1;UBA52
R-HSA-381119	Unfolded Protein Response (UPR)	2	155	0.011	0.102736768	1.09E-01	1	94	0.0069	9606	Homo sapiens	HSP90B1
R-HSA-5637812	Signaling by EGFRvIII in Cancer	1	22	0.002	0.074238711	1.09E-01	18	18	0.0013	9606	Homo sapiens	HSP90AA1
R-HSA-5637810	Constitutive Signaling by EGFRvIII	1	22	0.002	0.074238711	1.09E-01	18	18	0.0013	9606	Homo sapiens	HSP90AA1
R-HSA-8866652	Synthesis of active ubiquitin: roles of E1 and E2 enzymes	1	33	0.002	0.109304777	1.09E-01	13	16	0.0012	9606	Homo sapiens	UBA52
R-HSA-2173795	Downregulation of SMAD2/3:SMAD4 transcriptional activity	1	33	0.002	0.109304777	1.09E-01	9	25	0.0018	9606	Homo sapiens	UBA52
R-HSA-2173788	Downregulation of TGF-beta receptor signaling	1	28	0.002	0.093530059	1.09E-01	8	28	0.0021	9606	Homo sapiens	UBA52
R-HSA-110320	Translesion Synthesis by POLH	1	24	0.002	0.080713446	1.09E-01	7	8	0.0006	9606	Homo sapiens	UBA52
R-HSA-844456	The NLRP3 inflammasome	1	24	0.002	0.080713446	1.09E-01	7	18	0.0013	9606	Homo sapiens	HSP90AB1
R-HSA-389977	Post-chaperonin tubulin folding pathway	1	25	0.002	0.08393415	1.09E-01	6	9	0.0007	9606	Homo sapiens	TUBB
R-HSA-937072	TRAF6-mediated induction of TAK1 complex within TLR4 complex	1	19	0.001	0.064442732	1.09E-01	5	6	0.0004	9606	Homo sapiens	UBA52
R-HSA-2122948	Activated NOTCH1 Transmits Signal to the Nucleus	1	32	0.002	0.106171508	1.09E-01	5	21	0.0015	9606	Homo sapiens	UBA52
R-HSA-975163	IRAK2 mediated activation of TAK1 complex upon TLR7/8 or 9 sti	1	18	0.001	0.061154887	1.09E-01	4	5	0.0004	9606	Homo sapiens	UBA52
R-HSA-110312	Translesion synthesis by REV1	1	19	0.001	0.064442732	1.09E-01	4	5	0.0004	9606	Homo sapiens	UBA52
R-HSA-3134975	Regulation of innate immune responses to cytosolic DNA	1	21	0.001	0.070984606	1.09E-01	4	9	0.0007	9606	Homo sapiens	UBA52
R-HSA-9665348	Signaling by ERBB2 ECD mutants	1	23	0.002	0.077481645	1.09E-01	4	15	0.0011	9606	Homo sapiens	HSP90AA1
R-HSA-8876384	Listeria monocytogenes entry into host cells	1	27	0.002	0.090342419	1.09E-01	4	13	0.0010	9606	Homo sapiens	UBA52
R-HSA-175474	Assembly Of The HIV Virion	1	27	0.002	0.090342419	1.09E-01	4	16	0.0012	9606	Homo sapiens	UBA52
R-HSA-9665686	Signaling by ERBB2 TMD/JMD mutants	1	30	0.002	0.099872532	1.09E-01	4	13	0.0010	9606	Homo sapiens	HSP90AA1
R-HSA-174490	Membrane binding and targetting of GAG proteins	1	17	0.001	0.057855719	1.09E-01	3	4	0.0003	9606	Homo sapiens	UBA52
R-HSA-174495	Synthesis And Processing Of GAG, GAGPOL Polyproteins	1	18	0.001	0.061154887	1.09E-01	3	5	0.0004	9606	Homo sapiens	UBA52
R-HSA-205043	NRIF signals cell death from the nucleus	1	18	0.001	0.061154887	1.09E-01	3	7	0.0005	9606	Homo sapiens	UBA52
R-HSA-8875360	InlB-mediated entry of Listeria monocytogenes into host cell	1	19	0.001	0.064442732	1.09E-01	3	8	0.0006	9606	Homo sapiens	UBA52
R-HSA-5656121	Translesion synthesis by POLI	1	20	0.001	0.067719293	1.09E-01	3	3	0.0002	9606	Homo sapiens	UBA52
R-HSA-5655862	Translesion synthesis by POLK	1	21	0.001	0.070984606	1.09E-01	3	3	0.0002	9606	Homo sapiens	UBA52
R-HSA-4641263	Regulation of FZD by ubiquitination	1	22	0.002	0.074238711	1.09E-01	3	6	0.0004	9606	Homo sapiens	UBA52
R-HSA-6807004	Negative regulation of MET activity	1	25	0.002	0.08393415	1.09E-01	3	12	0.0009	9606	Homo sapiens	UBA52
R-HSA-110314	Recognition of DNA damage by PCNA-containing replication com	1	30	0.002	0.099872532	1.09E-01	3	6	0.0004	9606	Homo sapiens	UBA52
R-HSA-3214842	HDMs demethylate histones	1	31	0.002	0.103027439	1.09E-01	3	17	0.0013	9606	Homo sapiens	H4C1
R-HSA-5684264	MAP3K8 (TPL2)-dependent MAPK1/3 activation	1	18	0.001	0.061154887	1.09E-01	2	8	0.0006	9606	Homo sapiens	UBA52
R-HSA-2173791	TGF-beta receptor signaling in EMT (epithelial to mesenchymal t	1	19	0.001	0.064442732	1.09E-01	2	6	0.0004	9606	Homo sapiens	UBA52
R-HSA-1295596	Spry regulation of FGF signaling	1	19	0.001	0.064442732	1.09E-01	2	11	0.0008	9606	Homo sapiens	UBA52
R-HSA-193639	p75NTR signals via NF-kB	1	19	0.001	0.064442732	1.09E-01	2	13	0.0010	9606	Homo sapiens	UBA52
R-HSA-936964	Activation of IRF3/IRF7 mediated by TBK1/IKK epsilon	1	20	0.001	0.067719293	1.09E-01	2	9	0.0007	9606	Homo sapiens	UBA52
R-HSA-2979096	NOTCH2 Activation and Transmission of Signal to the Nucleus	1	23	0.002	0.077481645	1.09E-01	2	11	0.0008	9606	Homo sapiens	UBA52
R-HSA-174048	APC/C:Cdc20 mediated degradation of Cyclin B	1	24	0.002	0.080713446	1.09E-01	2	3	0.0002	9606	Homo sapiens	UBA52
R-HSA-9609523	Insertion of tail-anchored proteins into the endoplasmic reticulun	1	25	0.002	0.08393415	1.09E-01	2	7	0.0005	9606	Homo sapiens	ANXA4

R-HSA-179409	APC-Cdc20 mediated degradation of Nek2A	1	26	0.002	0.087143795	1.09E-01	2	3	0.0002	9606	Homo sapiens	UBA52
R-HSA-9636383	Prevention of phagosomal-lysosomal fusion	1	26	0.002	0.087143795	1.09E-01	2	11	0.0008	9606	Homo sapiens	UBA52
R-HSA-3322077	Glycogen synthesis	1	26	0.002	0.087143795	1.09E-01	2	24	0.0018	9606	Homo sapiens	UBA52
R-HSA-5696397	Gap-filling DNA repair synthesis and ligation in GG-NER	1	27	0.002	0.090342419	1.09E-01	2	2	0.0001	9606	Homo sapiens	UBA52
R-HSA-9013507	NOTCH3 Activation and Transmission of Signal to the Nucleus	1	28	0.002	0.093530059	1.09E-01	2	15	0.0011	9606	Homo sapiens	UBA52
R-HSA-9637687	Suppression of phagosomal maturation	1	32	0.002	0.106171508	1.09E-01	2	17	0.0013	9606	Homo sapiens	UBA52
R-HSA-445095	Interaction between L1 and Ankyrins	1	33	0.002	0.109304777	1.09E-01	2	4	0.0003	9606	Homo sapiens	ACTG1
R-HSA-901032	ER Quality Control Compartment (ERQC)	1	33	0.002	0.109304777	1.09E-01	2	9	0.0007	9606	Homo sapiens	UBA52
R-HSA-975110	TRAF6 mediated IRF7 activation in TLR7/8 or 9 signaling	1	17	0.001	0.057855719	1.09E-01	1	5	0.0004	9606	Homo sapiens	UBA52
R-HSA-9708530	Regulation of BACH1 activity	1	18	0.001	0.061154887	1.09E-01	1	7	0.0005	9606	Homo sapiens	UBA52
R-HSA-193144	Estrogen biosynthesis	1	18	0.001	0.061154887	1.09E-01	1	7	0.0005	9606	Homo sapiens	AKR1B10
R-HSA-168927	TICAM1, RIP1-mediated IKK complex recruitment	1	19	0.001	0.064442732	1.09E-01	1	3	0.0002	9606	Homo sapiens	UBA52
R-HSA-8876725	Protein methylation	1	19	0.001	0.064442732	1.09E-01	1	9	0.0007	9606	Homo sapiens	HSPA8
R-HSA-446353	Cell-extracellular matrix interactions	1	19	0.001	0.064442732	1.09E-01	1	10	0.0007	9606	Homo sapiens	ACTG1
R-HSA-9706369	Negative regulation of FLT3	1	20	0.001	0.067719293	1.09E-01	1	12	0.0009	9606	Homo sapiens	UBA52
R-HSA-190840	Microtubule-dependent trafficking of connexons from Golgi to th	1	22	0.002	0.074238711	1.09E-01	1	2	0.0001	9606	Homo sapiens	TUBB
R-HSA-5205685	PINK1-PRKN Mediated Mitophagy	1	22	0.002	0.074238711	1.09E-01	1	8	0.0006	9606	Homo sapiens	UBA52
R-HSA-3229121	Glycogen storage diseases	1	22	0.002	0.074238711	1.09E-01	1	10	0.0007	9606	Homo sapiens	UBA52
R-HSA-190872	Transport of connexons to the plasma membrane	1	23	0.002	0.077481645	1.09E-01	1	3	0.0002	9606	Homo sapiens	TUBB
R-HSA-6804760	Regulation of TP53 Activity through Methylation	1	23	0.002	0.077481645	1.09E-01	1	12	0.0009	9606	Homo sapiens	UBA52
R-HSA-937041	IKK complex recruitment mediated by RIP1	1	24	0.002	0.080713446	1.09E-01	1	3	0.0002	9606	Homo sapiens	UBA52
R-HSA-912631	Regulation of signaling by CBL	1	24	0.002	0.080713446	1.09E-01	1	7	0.0005	9606	Homo sapiens	UBA52
R-HSA-450321	JNK (c-Jun kinases) phosphorylation and activation mediated by a	1	26	0.002	0.087143795	1.09E-01	1	3	0.0002	9606	Homo sapiens	UBA52
R-HSA-450302	activated TAK1 mediates p38 MAPK activation	1	27	0.002	0.090342419	1.09E-01	1	5	0.0004	9606	Homo sapiens	UBA52
R-HSA-9705462	Inactivation of CSF3 (G-CSF) signaling	1	27	0.002	0.090342419	1.09E-01	1	9	0.0007	9606	Homo sapiens	UBA52
R-HSA-5357956	TNFR1-induced NFkappaB signaling pathway	1	30	0.002	0.099872532	1.09E-01	1	5	0.0004	9606	Homo sapiens	UBA52
R-HSA-8862803	Deregulated CDK5 triggers multiple neurodegenerative pathways	1	30	0.002	0.099872532	1.09E-01	1	22	0.0016	9606	Homo sapiens	PRDX1
R-HSA-8863678	Neurodegenerative Diseases	1	30	0.002	0.099872532	1.09E-01	1	22	0.0016	9606	Homo sapiens	PRDX1
R-HSA-5205647	Mitophagy	1	31	0.002	0.103027439	1.09E-01	1	15	0.0011	9606	Homo sapiens	UBA52
R-HSA-5601884	PIWI-interacting RNA (piRNA) biogenesis	1	31	0.002	0.103027439	1.09E-01	1	15	0.0011	9606	Homo sapiens	HSP90AA1
R-HSA-9734009	Defective Intrinsic Pathway for Apoptosis	1	33	0.002	0.109304777	1.09E-01	1	24	0.0018	9606	Homo sapiens	PRDX1
R-HSA-195721	Signaling by WNT	3	332	0.023	0.110525007	1.11E-01	19	157	0.0116	9606	Homo sapiens	H2AC19;H4C1;UBA52
R-HSA-622312	Inflammasomes	1	34	0.002	0.112427281	1.12E-01	7	28	0.0021	9606	Homo sapiens	HSP90AB1
R-HSA-5654732	Negative regulation of FGFR3 signaling	1	34	0.002	0.112427281	1.12E-01	3	14	0.0010	9606	Homo sapiens	UBA52
R-HSA-9619483	Activation of AMPK downstream of NMDARs	1	34	0.002	0.112427281	1.12E-01	1	3	0.0002	9606	Homo sapiens	TUBB
R-HSA-162599	Late Phase of HIV Life Cycle	2	164	0.011	0.112804131	1.13E-01	18	78	0.0057	9606	Homo sapiens	UBA52
R-HSA-5656169	Termination of translesion DNA synthesis	1	35	0.002	0.115539057	1.16E-01	6	9	0.0007	9606	Homo sapiens	UBA52
R-HSA-917729	Endosomal Sorting Complex Required For Transport (ESCRT)	1	35	0.002	0.115539057	1.16E-01	4	4	0.0003	9606	Homo sapiens	UBA52
R-HSA-9664565	Signaling by ERBB2 KD Mutants	1	35	0.002	0.115539057	1.16E-01	4	17	0.0013	9606	Homo sapiens	HSP90AA1
R-HSA-5654733	Negative regulation of FGFR4 signaling	1	35	0.002	0.115539057	1.16E-01	3	14	0.0010	9606	Homo sapiens	UBA52
R-HSA-9674555	Signaling by CSF3 (G-CSF)	1	35	0.002	0.115539057	1.16E-01	1	21	0.0015	9606	Homo sapiens	UBA52
R-HSA-5693532	DNA Double-Strand Break Repair	2	168	0.012	0.117359395	1.17E-01	37	111	0.0082	9606	Homo sapiens	H4C1;UBA52
R-HSA-5358351	Signaling by Hedgehog	2	168	0.012	0.117359395	1.17E-01	12	82	0.0060	9606	Homo sapiens	TUBB;UBA52
R-HSA-2173782	Binding and Uptake of Ligands by Scavenger Receptors	2	168	0.012	0.117359395	1.17E-01	4	33	0.0024	9606	Homo sapiens	HSP90AA1;HSP90B1
R-HSA-1227990	Signaling by ERBB2 in Cancer	1	36	0.003	0.118640141	1.19E-01	25	62	0.0046	9606	Homo sapiens	HSP90AA1
R-HSA-2173789	TGF-beta receptor signaling activates SMADs	1	36	0.003	0.118640141	1.19E-01	8	39	0.0029	9606	Homo sapiens	UBA52
R-HSA-9609507	Protein localization	2	170	0.012	0.119654669	1.20E-01	14	53	0.0039	9606	Homo sapiens	ANXA4;UBA52
R-HSA-203615	eNOS activation	1	37	0.003	0.121730568	1.22E-01	8	20	0.0015	9606	Homo sapiens	HSP90AA1
R-HSA-182971	EGFR downregulation	1	37	0.003	0.121730568	1.22E-01	6	22	0.0016	9606	Homo sapiens	UBA52
R-HSA-936440	Negative regulators of DDX58/IFIH1 signaling	1	37	0.003	0.121730568	1.22E-01	4	13	0.0010	9606	Homo sapiens	UBA52
R-HSA-162588	Budding and maturation of HIV virion	1	37	0.003	0.121730568	1.22E-01	2	4	0.0003	9606	Homo sapiens	UBA52
R-HSA-888590	GABA synthesis, release, reuptake and degradation	1	37	0.003	0.121730568	1.22E-01	2	10	0.0007	9606	Homo sapiens	HSPA8
R-HSA-1474151	Tetrahydrobiopterin (BH4) synthesis, recycling, salvage and regula	1	37	0.003	0.121730568	1.22E-01	2	16	0.0012	9606	Homo sapiens	HSP90AA1
R-HSA-2173796	SMAD2/SMAD3:SMAD4 heterotrimer regulates transcription	1	38	0.003	0.124810375	1.25E-01	2	19	0.0014	9606	Homo sapiens	UBA52
R-HSA-1980145	Signaling by NOTCH2	1	38	0.003	0.124810375	1.25E-01	2	20	0.0015	9606	Homo sapiens	UBA52
R-HSA-9013407	RHOH GTPase cycle	1	38	0.003	0.124810375	1.25E-01	1	3	0.0002	9606	Homo sapiens	JUP
R-HSA-9610379	HCMV Late Events	2	175	0.012	0.125442179	1.25E-01	1	19	0.0014	9606	Homo sapiens	H2AC19;H4C1
R-HSA-5654726	Negative regulation of FGFR1 signaling	1	39	0.003	0.127879597	1.28E-01	3	15	0.0011	9606	Homo sapiens	UBA52
R-HSA-445989	TAK1 activates NFkB by phosphorylation and activation of IKKs co	1	39	0.003	0.127879597	1.28E-01	1	5	0.0004	9606	Homo sapiens	UBA52
R-HSA-9668328	Sealing of the nuclear envelope (NE) by ESCRT-III	1	39	0.003	0.127879597	1.28E-01	1	7	0.0005	9606	Homo sapiens	TUBB
R-HSA-5633007	Regulation of TP53 Activity	2	178	0.012	0.128947209	1.29E-01	10	98	0.0072	9606	Homo sapiens	UBA52
R-HSA-162587	HIV Life Cycle	2	179	0.013	0.13012078	1.30E-01	18	117	0.0086	9606	Homo sapiens	UBA52
R-HSA-168638	NOD1/2 Signaling Pathway	1	40	0.003	0.13093827	1.31E-01	7	18	0.0013	9606	Homo sapiens	UBA52
R-HSA-5696394	DNA Damage Recognition in GG-NER	1	40	0.003	0.13093827	1.31E-01	3	5	0.0004	9606	Homo sapiens	UBA52
R-HSA-202131	Metabolism of nitric oxide: NOS3 activation and regulation	1	41	0.003	0.13398643	1.34E-01	8	26	0.0019	9606	Homo sapiens	HSP90AA1
R-HSA-5663213	RHO GTPases Activate WASPs and WAVEs	1	41	0.003	0.13398643	1.34E-01	4	10	0.0007	9606	Homo sapiens	ACTG1
R-HSA-5654727	Negative regulation of FGFR2 signaling	1	41	0.003	0.13398643	1.34E-01	3	14	0.0010	9606	Homo sapiens	UBA52
R-HSA-190861	Gap junction assembly	1	41	0.003	0.13398643	1.34E-01	3	16	0.0012	9606	Homo sapiens	TUBB
R-HSA-5696400	Dual Incision in GG-NER	1	41	0.003	0.13398643	1.34E-01	2	3	0.0002	9606	Homo sapiens	UBA52
R-HSA-901042	Calnexin/calreticulin cycle	1	41	0.003	0.13398643	1.34E-01	2	13	0.0010	9606	Homo sapiens	UBA52
R-HSA-5357905	Regulation of TNFR1 signaling	1	41	0.003	0.13398643	1.34E-01	2	17	0.0013	9606	Homo sapiens	UBA52
R-HSA-2559585	Oncogene Induced Senescence	1	42	0.003	0.137024112	1.37E-01	1	19	0.0014	9606	Homo sapiens	UBA52
R-HSA-6804757	Regulation of TP53 Degradation	1	43	0.003	0.140051351	1.40E-01	7	30	0.0022	9606	Homo sapiens	UBA52
R-HSA-8982491	Glycogen metabolism	1	43	0.003	0.140051351	1.40E-01	2	39	0.0029	9606	Homo sapiens	UBA52
R-HSA-432720	Lysosome Vesicle Biogenesis	1	43	0.003	0.140051351	1.40E-01	1	8	0.0006	9606	Homo sapiens	HSPA8
R-HSA-9682385	FLT3 signaling in disease	1	43	0.003	0.140051351	1.40E-01	1	52	0.0038	9606	Homo sapiens	UBA52
R-HSA-168898	Toll-like Receptor Cascades	2	188	0.013	0.140794485	1.41E-01	33	185	0.0136	9606	Homo sapiens	UBA52;HSP90B1

R-HSA-5689896	Ovarian tumor domain proteases	1	44	0.003	0.143068182	1.43E-01	7	14	0.0010	9606	Homo sapiens	UBA52
R-HSA-6802948	Signaling by high-kinase activity BRAF mutants	1	44	0.003	0.143068182	1.43E-01	4	6	0.0004	9606	Homo sapiens	ACTG1
R-HSA-419037	NCAM1 interactions	1	44	0.003	0.143068182	1.43E-01	1	10	0.0007	9606	Homo sapiens	ANXA4
R-HSA-3769402	Deactivation of the beta-catenin transactivating complex	1	44	0.003	0.143068182	1.43E-01	1	14	0.0010	9606	Homo sapiens	UBA52
R-HSA-73894	DNA Repair	3	380	0.027	0.148026671	1.48E-01	102	337	0.0248	9606	Homo sapiens	H2AC19;H4C1;UBA52
R-HSA-5683057	MAPK family signaling cascades	3	380	0.027	0.148026671	1.48E-01	17	122	0.0090	9606	Homo sapiens	NF1;UBA52;ACTG1
R-HSA-6806003	Regulation of TP53 Expression and Degradation	1	46	0.003	0.149070762	1.49E-01	7	35	0.0026	9606	Homo sapiens	UBA52
R-HSA-9648002	RAS processing	1	46	0.003	0.149070762	1.49E-01	1	21	0.0015	9606	Homo sapiens	UBA52
R-HSA-2467813	Separation of Sister Chromatids	2	195	0.014	0.149225469	1.49E-01	4	8	0.0006	9606	Homo sapiens	TUBB;UBA52
R-HSA-5696395	Formation of Incision Complex in GG-NER	1	47	0.003	0.15205658	1.52E-01	7	10	0.0007	9606	Homo sapiens	UBA52
R-HSA-73779	RNA Polymerase II Transcription Pre-Initiation And Promoter Ope	1	47	0.003	0.15205658	1.52E-01	5	5	0.0004	9606	Homo sapiens	UBA52
R-HSA-110313	Translesion synthesis by Y family DNA polymerases bypasses lesi	1	48	0.003	0.15503213	1.55E-01	23	28	0.0021	9606	Homo sapiens	UBA52
R-HSA-9607240	FLT3 Signaling	1	48	0.003	0.15503213	1.55E-01	1	43	0.0032	9606	Homo sapiens	UBA52
R-HSA-5674135	MAP2K and MAPK activation	1	49	0.003	0.157997447	1.58E-01	8	12	0.0009	9606	Homo sapiens	ACTG1
R-HSA-9656223	Signaling by RAF1 mutants	1	49	0.003	0.157997447	1.58E-01	4	7	0.0005	9606	Homo sapiens	ACTG1
R-HSA-3214841	PKMTs methylate histone lysines	1	49	0.003	0.157997447	1.58E-01	3	22	0.0016	9606	Homo sapiens	H4C1
R-HSA-3000480	Scavenging by Class A Receptors	1	49	0.003	0.157997447	1.58E-01	2	10	0.0007	9606	Homo sapiens	HSP90B1
R-HSA-9609736	Assembly and cell surface presentation of NMDA receptors	1	49	0.003	0.157997447	1.58E-01	2	23	0.0017	9606	Homo sapiens	TUBB
R-HSA-5675221	Negative regulation of MAPK pathway	1	49	0.003	0.157997447	1.58E-01	1	17	0.0013	9606	Homo sapiens	UBA52
R-HSA-9683701	Translation of Structural Proteins	1	49	0.003	0.157997447	1.58E-01	1	37	0.0027	9606	Homo sapiens	UBA52
R-HSA-211733	Regulation of activated PAK-2p34 by proteasome mediated degra	1	50	0.004	0.160952565	1.61E-01	2	2	0.0001	9606	Homo sapiens	UBA52
R-HSA-379716	Cytosolic tRNA aminoacylation	1	50	0.004	0.160952565	1.61E-01	1	21	0.0015	9606	Homo sapiens	TARS1
R-HSA-512988	Interleukin-3, Interleukin-5 and GM-CSF signaling	1	50	0.004	0.160952565	1.61E-01	1	38	0.0028	9606	Homo sapiens	UBA52
R-HSA-5689880	Ub-specific processing proteases	2	206	0.014	0.16267666	1.63E-01	36	40	0.0029	9606	Homo sapiens	H2AC19;UBA52
R-HSA-75893	TNF signaling	1	51	0.004	0.163897518	1.64E-01	3	32	0.0024	9606	Homo sapiens	UBA52
R-HSA-5654743	Signaling by FGFR4	1	51	0.004	0.163897518	1.64E-01	3	41	0.0030	9606	Homo sapiens	UBA52
R-HSA-9637690	Response of Mtb to phagocytosis	1	51	0.004	0.163897518	1.64E-01	2	30	0.0022	9606	Homo sapiens	UBA52
R-HSA-69231	Cyclin D associated events in G1	1	51	0.004	0.163897518	1.64E-01	1	15	0.0011	9606	Homo sapiens	UBA52
R-HSA-69236	G1 Phase	1	51	0.004	0.163897518	1.64E-01	1	15	0.0011	9606	Homo sapiens	UBA52
R-HSA-167162	RNA Polymerase II HIV Promoter Escape	1	52	0.004	0.166832341	1.67E-01	6	9	0.0007	9606	Homo sapiens	UBA52
R-HSA-167161	HIV Transcription Initiation	1	52	0.004	0.166832341	1.67E-01	4	4	0.0003	9606	Homo sapiens	UBA52
R-HSA-75953	RNA Polymerase II Transcription Initiation	1	52	0.004	0.166832341	1.67E-01	3	3	0.0002	9606	Homo sapiens	UBA52
R-HSA-3928662	EPHB-mediated forward signaling	1	52	0.004	0.166832341	1.67E-01	1	26	0.0019	9606	Homo sapiens	ACTG1
R-HSA-6783310	Fanconi Anemia Pathway	1	53	0.004	0.169757067	1.70E-01	6	15	0.0011	9606	Homo sapiens	UBA52
R-HSA-73776	RNA Polymerase II Promoter Escape	1	53	0.004	0.169757067	1.70E-01	5	7	0.0005	9606	Homo sapiens	UBA52
R-HSA-5654741	Signaling by FGFR3	1	53	0.004	0.169757067	1.70E-01	3	43	0.0032	9606	Homo sapiens	UBA52
R-HSA-180534	Vpu mediated degradation of CD4	1	53	0.004	0.169757067	1.70E-01	2	5	0.0004	9606	Homo sapiens	UBA52
R-HSA-9006934	Signaling by Receptor Tyrosine Kinases	4	618	0.043	0.170191808	1.70E-01	48	746	0.0549	9606	Homo sapiens	HSP90AA1;JUP;UBA52;ACTG1
R-HSA-9660826	Purinergic signaling in leishmaniasis infection	1	54	0.004	0.172671731	1.73E-01	5	23	0.0017	9606	Homo sapiens	HSP90AB1
R-HSA-9664424	Cell recruitment (pro-inflammatory response)	1	54	0.004	0.172671731	1.73E-01	5	23	0.0017	9606	Homo sapiens	HSP90AB1
R-HSA-9649948	Signaling downstream of RAS mutants	1	54	0.004	0.172671731	1.73E-01	4	7	0.0005	9606	Homo sapiens	ACTG1
R-HSA-6802946	Signaling by moderate kinase activity BRAF mutants	1	54	0.004	0.172671731	1.73E-01	4	7	0.0005	9606	Homo sapiens	ACTG1
R-HSA-6802955	Paradoxical activation of RAF signaling by kinase inactive BRAF	1	54	0.004	0.172671731	1.73E-01	4	7	0.0005	9606	Homo sapiens	ACTG1
R-HSA-6802949	Signaling by RAS mutants	1	54	0.004	0.172671731	1.73E-01	4	9	0.0007	9606	Homo sapiens	ACTG1
R-HSA-69613	p53-Independent G1/S DNA damage checkpoint	1	54	0.004	0.172671731	1.73E-01	2	4	0.0003	9606	Homo sapiens	UBA52
R-HSA-69601	Ubiquitin Mediated Degradation of Phosphorylated Cdc25A	1	54	0.004	0.172671731	1.73E-01	2	4	0.0003	9606	Homo sapiens	UBA52
R-HSA-69610	p53-Independent DNA Damage Response	1	54	0.004	0.172671731	1.73E-01	2	4	0.0003	9606	Homo sapiens	UBA52
R-HSA-349425	Autodegradation of the E3 ubiquitin ligase COP1	1	54	0.004	0.172671731	1.73E-01	2	5	0.0004	9606	Homo sapiens	UBA52
R-HSA-75815	Ubiquitin-dependent degradation of Cyclin D	1	54	0.004	0.172671731	1.73E-01	2	5	0.0004	9606	Homo sapiens	UBA52
R-HSA-169911	Regulation of Apoptosis	1	54	0.004	0.172671731	1.73E-01	2	5	0.0004	9606	Homo sapiens	UBA52
R-HSA-72695	Formation of the ternary complex, and subsequently, the 43S con	1	54	0.004	0.172671731	1.73E-01	1	3	0.0002	9606	Homo sapiens	RPSA
R-HSA-76042	RNA Polymerase II Transcription Initiation And Promoter Clearanc	1	55	0.004	0.175576367	1.76E-01	9	11	0.0008	9606	Homo sapiens	UBA52
R-HSA-8955332	Carboxyterminal post-translational modifications of tubulin	1	55	0.004	0.175576367	1.76E-01	6	6	0.0004	9606	Homo sapiens	TUBB
R-HSA-8854050	FBXL7 down-regulates AURKA during mitotic entry and in early m	1	55	0.004	0.175576367	1.76E-01	4	6	0.0004	9606	Homo sapiens	UBA52
R-HSA-174113	SCF-beta-TrCP mediated degradation of Emi1	1	55	0.004	0.175576367	1.76E-01	2	3	0.0002	9606	Homo sapiens	UBA52
R-HSA-3928665	EPH-ephrin mediated repulsion of cells	1	55	0.004	0.175576367	1.76E-01	1	9	0.0007	9606	Homo sapiens	ACTG1
R-HSA-5620924	Intraflagellar transport	1	56	0.004	0.178471008	1.78E-01	6	12	0.0009	9606	Homo sapiens	TUBB
R-HSA-6781823	Formation of TC-NER Pre-Incision Complex	1	56	0.004	0.178471008	1.78E-01	3	7	0.0005	9606	Homo sapiens	UBA52
R-HSA-180585	Vif-mediated degradation of APOBEC3G	1	56	0.004	0.178471008	1.78E-01	2	4	0.0003	9606	Homo sapiens	UBA52
R-HSA-73893	DNA Damage Bypass	1	57	0.004	0.181355689	1.81E-01	26	34	0.0025	9606	Homo sapiens	UBA52
R-HSA-2173793	Transcriptional activity of SMAD2/SMAD3:SMAD4 heterotrimer	1	57	0.004	0.181355689	1.81E-01	11	44	0.0032	9606	Homo sapiens	UBA52
R-HSA-4641258	Degradation of DVL	1	57	0.004	0.181355689	1.81E-01	4	7	0.0005	9606	Homo sapiens	UBA52
R-HSA-4641257	Degradation of AXIN	1	57	0.004	0.181355689	1.81E-01	4	8	0.0006	9606	Homo sapiens	UBA52
R-HSA-5693571	Nonhomologous End-Joining (NHEJ)	1	57	0.004	0.181355689	1.81E-01	4	13	0.0010	9606	Homo sapiens	H4C1
R-HSA-532668	N-glycan trimming in the ER and Calnexin/Calreticulin cycle	1	57	0.004	0.181355689	1.81E-01	4	18	0.0013	9606	Homo sapiens	UBA52
R-HSA-8941858	Regulation of RUNX3 expression and activity	1	57	0.004	0.181355689	1.81E-01	3	9	0.0007	9606	Homo sapiens	UBA52
R-HSA-9604323	Negative regulation of NOTCH4 signaling	1	57	0.004	0.181355689	1.81E-01	2	6	0.0004	9606	Homo sapiens	UBA52
R-HSA-2122947	NOTCH1 Intracellular Domain Regulates Transcription	1	57	0.004	0.181355689	1.81E-01	1	18	0.0013	9606	Homo sapiens	UBA52
R-HSA-72187	mRNA 3'-end processing	1	58	0.004	0.184230442	1.84E-01	3	3	0.0002	9606	Homo sapiens	SRSF3
R-HSA-69541	Stabilization of p53	1	59	0.004	0.187095301	1.87E-01	4	15	0.0011	9606	Homo sapiens	UBA52
R-HSA-68827	CDT1 association with the CDC6:ORC:origin complex	1	59	0.004	0.187095301	1.87E-01	2	3	0.0002	9606	Homo sapiens	UBA52
R-HSA-9013409	RHOJ GTPase cycle	1	59	0.004	0.187095301	1.87E-01	1	3	0.0002	9606	Homo sapiens	JUP
R-HSA-8932339	ROS sensing by NFE2L2	1	60	0.004	0.1899503	1.90E-01	2	8	0.0006	9606	Homo sapiens	UBA52
R-HSA-8866654	E3 ubiquitin ligases ubiquitinate target proteins	1	61	0.004	0.192795472	1.93E-01	16	16	0.0012	9606	Homo sapiens	UBA52
R-HSA-177929	Signaling by EGFR	1	61	0.004	0.192795472	1.93E-01	6	49	0.0036	9606	Homo sapiens	UBA52
R-HSA-5362768	Hh mutants are degraded by ERAD	1	61	0.004	0.192795472	1.93E-01	2	6	0.0004	9606	Homo sapiens	UBA52
R-HSA-5676590	NIK-->noncanonical NF-kB signaling	1	61	0.004	0.192795472	1.93E-01	2	9	0.0007	9606	Homo sapiens	UBA52

R-HSA-432722	Golgi Associated Vesicle Biogenesis	1	61	0.004	0.192795472	1.93E-01	1	7	0.0005	9606	Homo sapiens	HSPA8
R-HSA-3000171	Non-integrin membrane-ECM interactions	1	61	0.004	0.192795472	1.93E-01	1	22	0.0016	9606	Homo sapiens	TKT
R-HSA-5654736	Signaling by FGFR1	1	62	0.004	0.19563085	1.96E-01	3	48	0.0035	9606	Homo sapiens	UBA52
R-HSA-72649	Translation initiation complex formation	1	62	0.004	0.19563085	1.96E-01	2	2	0.0001	9606	Homo sapiens	RPSA
R-HSA-187577	SCF(Skp2)-mediated degradation of p27/p21	1	62	0.004	0.19563085	1.96E-01	2	7	0.0005	9606	Homo sapiens	UBA52
R-HSA-4551638	SUMOylation of chromatin organization proteins	1	62	0.004	0.19563085	1.96E-01	2	15	0.0011	9606	Homo sapiens	H4C1
R-HSA-5610785	GLI3 is processed to GLI3R by the proteasome	1	62	0.004	0.19563085	1.96E-01	1	5	0.0004	9606	Homo sapiens	UBA52
R-HSA-5610783	Degradation of GLI2 by the proteasome	1	62	0.004	0.19563085	1.96E-01	1	5	0.0004	9606	Homo sapiens	UBA52
R-HSA-5610780	Degradation of GLI1 by the proteasome	1	62	0.004	0.19563085	1.96E-01	1	6	0.0004	9606	Homo sapiens	UBA52
R-HSA-6811436	COPI-independent Golgi-to-ER retrograde traffic	1	63	0.004	0.198456467	1.98E-01	2	7	0.0005	9606	Homo sapiens	TUBB
R-HSA-9012852	Signaling by NOTCH3	1	63	0.004	0.198456467	1.98E-01	2	37	0.0027	9606	Homo sapiens	UBA52
R-HSA-9013406	RHOQ GTPase cycle	1	63	0.004	0.198456467	1.98E-01	1	5	0.0004	9606	Homo sapiens	JUP
R-HSA-72702	Ribosomal scanning and start codon recognition	1	64	0.004	0.201272355	2.01E-01	2	2	0.0001	9606	Homo sapiens	RPSA
R-HSA-174084	Autodegradation of Cdh1 by Cdh1:APC/C	1	64	0.004	0.201272355	2.01E-01	2	2	0.0001	9606	Homo sapiens	UBA52
R-HSA-5387390	Hh mutants abrogate ligand secretion	1	64	0.004	0.201272355	2.01E-01	2	7	0.0005	9606	Homo sapiens	UBA52
R-HSA-9662361	Sensory processing of sound by outer hair cells of the cochlea	1	64	0.004	0.201272355	2.01E-01	1	8	0.0006	9606	Homo sapiens	ACTG1
R-HSA-5357801	Programmed Cell Death	2	238	0.017	0.202908481	2.03E-01	7	197	0.0145	9606	Homo sapiens	HSP90AA1;UBA52
R-HSA-199991	Membrane Trafficking	4	667	0.047	0.204850016	2.05E-01	38	219	0.0161	9606	Homo sapiens	HSPA8;TUBB;UBA52;ACTG1
R-HSA-5678895	Defective CFTR causes cystic fibrosis	1	66	0.005	0.206875079	2.07E-01	3	7	0.0005	9606	Homo sapiens	UBA52
R-HSA-6782210	Gap-filling DNA repair synthesis and ligation in TC-NER	1	66	0.005	0.206875079	2.07E-01	2	2	0.0001	9606	Homo sapiens	UBA52
R-HSA-72662	Activation of the mRNA upon binding of the cap-binding complex	1	66	0.005	0.206875079	2.07E-01	2	6	0.0004	9606	Homo sapiens	RPSA
R-HSA-5607761	Dectin-1 mediated noncanonical NF-kB signaling	1	66	0.005	0.206875079	2.07E-01	2	9	0.0007	9606	Homo sapiens	UBA52
R-HSA-9725370	Signaling by ALK fusions and activated point mutants	1	66	0.005	0.206875079	2.07E-01	2	29	0.0021	9606	Homo sapiens	EEF1G
R-HSA-9700206	Signaling by ALK in cancer	1	66	0.005	0.206875079	2.07E-01	2	37	0.0027	9606	Homo sapiens	EEF1G
R-HSA-4608870	Asymmetric localization of PCP proteins	1	66	0.005	0.206875079	2.07E-01	1	7	0.0005	9606	Homo sapiens	UBA52
R-HSA-9033241	Peroxisomal protein import	1	67	0.005	0.20966198	2.10E-01	12	26	0.0019	9606	Homo sapiens	UBA52
R-HSA-73856	RNA Polymerase II Transcription Termination	1	67	0.005	0.20966198	2.10E-01	1	4	0.0003	9606	Homo sapiens	SRSF3
R-HSA-6782135	Dual incision in TC-NER	1	68	0.005	0.212439284	2.12E-01	7	7	0.0005	9606	Homo sapiens	UBA52
R-HSA-2644603	Signaling by NOTCH1 in Cancer	1	68	0.005	0.212439284	2.12E-01	6	39	0.0029	9606	Homo sapiens	UBA52
R-HSA-174154	APC/C:Cdc20 mediated degradation of Securin	1	68	0.005	0.212439284	2.12E-01	2	3	0.0002	9606	Homo sapiens	UBA52
R-HSA-983189	Kinesins	1	68	0.005	0.212439284	2.12E-01	2	14	0.0010	9606	Homo sapiens	TUBB
R-HSA-2644606	Constitutive Signaling by NOTCH1 PEST Domain Mutants	1	68	0.005	0.212439284	2.12E-01	2	21	0.0015	9606	Homo sapiens	UBA52
R-HSA-2894858	Signaling by NOTCH1 HD+PEST Domain Mutants in Cancer	1	68	0.005	0.212439284	2.12E-01	2	21	0.0015	9606	Homo sapiens	UBA52
R-HSA-2894862	Constitutive Signaling by NOTCH1 HD+PEST Domain Mutants	1	68	0.005	0.212439284	2.12E-01	2	21	0.0015	9606	Homo sapiens	UBA52
R-HSA-2644602	Signaling by NOTCH1 PEST Domain Mutants in Cancer	1	68	0.005	0.212439284	2.12E-01	2	21	0.0015	9606	Homo sapiens	UBA52
R-HSA-8978934	Metabolism of cofactors	1	68	0.005	0.212439284	2.12E-01	2	28	0.0021	9606	Homo sapiens	HSP90AA1
R-HSA-379724	tRNA Aminoacylation	1	68	0.005	0.212439284	2.12E-01	1	42	0.0031	9606	Homo sapiens	TARS1
R-HSA-450294	MAP kinase activation	1	69	0.005	0.215207022	2.15E-01	4	32	0.0024	9606	Homo sapiens	UBA52
R-HSA-3299685	Detoxification of Reactive Oxygen Species	1	69	0.005	0.215207022	2.15E-01	1	34	0.0025	9606	Homo sapiens	PRDX1
R-HSA-68882	Mitotic Anaphase	2	249	0.017	0.217012871	2.17E-01	5	32	0.0024	9606	Homo sapiens	TUBB;UBA52
R-HSA-69563	p53-Dependent G1 DNA Damage Response	1	70	0.005	0.217965227	2.18E-01	4	22	0.0016	9606	Homo sapiens	UBA52
R-HSA-69580	p53-Dependent G1/S DNA damage checkpoint	1	70	0.005	0.217965227	2.18E-01	4	22	0.0016	9606	Homo sapiens	UBA52
R-HSA-375165	NCAM signaling for neurite out-growth	1	70	0.005	0.217965227	2.18E-01	1	23	0.0017	9606	Homo sapiens	ANXA4
R-HSA-2555396	Mitotic Metaphase and Anaphase	2	250	0.018	0.218300085	2.18E-01	5	33	0.0024	9606	Homo sapiens	TUBB;UBA52
R-HSA-9006927	Signaling by Non-Receptor Tyrosine Kinases	1	71	0.005	0.220713932	2.21E-01	1	53	0.0039	9606	Homo sapiens	UBA52
R-HSA-8848021	Signaling by PTK6	1	71	0.005	0.220713932	2.21E-01	1	53	0.0039	9606	Homo sapiens	UBA52
R-HSA-69615	G1/S DNA Damage Checkpoints	1	72	0.005	0.223453168	2.23E-01	6	26	0.0019	9606	Homo sapiens	UBA52
R-HSA-1234176	Oxygen-dependent proline hydroxylation of Hypoxia-inducible Fac	1	72	0.005	0.223453168	2.23E-01	4	12	0.0009	9606	Homo sapiens	UBA52
R-HSA-5358346	Hedgehog ligand biogenesis	1	72	0.005	0.223453168	2.23E-01	2	15	0.0011	9606	Homo sapiens	UBA52
R-HSA-1169091	Activation of NF-kappaB in B cells	1	72	0.005	0.223453168	2.23E-01	1	10	0.0007	9606	Homo sapiens	UBA52
R-HSA-5685942	HDR through Homologous Recombination (HRR)	1	72	0.005	0.223453168	2.23E-01	1	19	0.0014	9606	Homo sapiens	UBA52
R-HSA-196071	Metabolism of steroid hormones	1	72	0.005	0.223453168	2.23E-01	1	40	0.0029	9606	Homo sapiens	AKR1B10
R-HSA-4839726	Chromatin organization	2	256	0.018	0.226038252	2.26E-01	28	85	0.0063	9606	Homo sapiens	H2AC19;H4C1
R-HSA-3247509	Chromatin modifying enzymes	2	256	0.018	0.226038252	2.26E-01	28	85	0.0063	9606	Homo sapiens	H2AC19;H4C1
R-HSA-6802952	Signaling by BRAF and RAF1 fusions	1	73	0.005	0.226182967	2.26E-01	4	5	0.0004	9606	Homo sapiens	ACTG1
R-HSA-174184	Cdc20:Phospho-APC/C mediated degradation of Cyclin A	1	73	0.005	0.226182967	2.26E-01	2	3	0.0002	9606	Homo sapiens	UBA52
R-HSA-68949	Orc1 removal from chromatin	1	73	0.005	0.226182967	2.26E-01	2	4	0.0003	9606	Homo sapiens	UBA52
R-HSA-9694635	Translation of Structural Proteins	1	73	0.005	0.226182967	2.26E-01	1	43	0.0032	9606	Homo sapiens	UBA52
R-HSA-8948751	Regulation of PTEN stability and activity	1	74	0.005	0.228903362	2.29E-01	6	13	0.0010	9606	Homo sapiens	UBA52
R-HSA-174178	APC/C:Cdh1 mediated degradation of Cdc20 and other APC/C:Cdh	1	74	0.005	0.228903362	2.29E-01	4	6	0.0004	9606	Homo sapiens	UBA52
R-HSA-179419	APC:Cdc20 mediated degradation of cell cycle proteins prior to sa	1	74	0.005	0.228903362	2.29E-01	4	6	0.0004	9606	Homo sapiens	UBA52
R-HSA-9707587	Regulation of HMOX1 expression and activity	1	75	0.005	0.231614383	2.32E-01	3	19	0.0014	9606	Homo sapiens	UBA52
R-HSA-69017	CDK-mediated phosphorylation and removal of Cdc6	1	75	0.005	0.231614383	2.32E-01	2	4	0.0003	9606	Homo sapiens	UBA52
R-HSA-9013026	RHOB GTPase cycle	1	75	0.005	0.231614383	2.32E-01	1	6	0.0004	9606	Homo sapiens	JUP
R-HSA-176409	APC/C:Cdc20 mediated degradation of mitotic proteins	1	76	0.005	0.234316063	2.34E-01	8	13	0.0010	9606	Homo sapiens	UBA52
R-HSA-9662360	Sensory processing of sound by inner hair cells of the cochlea	1	76	0.005	0.234316063	2.34E-01	1	7	0.0005	9606	Homo sapiens	ACTG1
R-HSA-162906	HIV Infection	2	264	0.018	0.236389918	2.36E-01	22	160	0.0118	9606	Homo sapiens	UBA52
R-HSA-176814	Activation of APC/C and APC/C:Cdc20 mediated degradation of m	1	77	0.005	0.237008433	2.37E-01	8	15	0.0011	9606	Homo sapiens	UBA52
R-HSA-448424	Interleukin-17 signaling	1	77	0.005	0.237008433	2.37E-01	4	35	0.0026	9606	Homo sapiens	UBA52
R-HSA-1834949	Cytosolic sensors of pathogen-associated DNA	1	78	0.005	0.239691525	2.40E-01	4	50	0.0037	9606	Homo sapiens	UBA52
R-HSA-9013106	RHOC GTPase cycle	1	79	0.006	0.24236537	2.42E-01	1	6	0.0004	9606	Homo sapiens	JUP
R-HSA-975634	Retinoid metabolism and transport	1	79	0.006	0.24236537	2.42E-01	1	28	0.0021	9606	Homo sapiens	AKR1B10
R-HSA-199992	trans-Golgi Network Vesicle Budding	1	80	0.006	0.245029998	2.45E-01	2	19	0.0014	9606	Homo sapiens	HSPA8
R-HSA-167172	Transcription of the HIV genome	1	81	0.006	0.247685442	2.48E-01	12	47	0.0035	9606	Homo sapiens	UBA52
R-HSA-204998	Cell death signalling via NRAGE, NRIF and NADE	1	81	0.006	0.247685442	2.48E-01	3	17	0.0013	9606	Homo sapiens	UBA52
R-HSA-159236	Transport of Mature mRNA derived from an Intron-Containing Tra	1	81	0.006	0.247685442	2.48E-01	2	4	0.0003	9606	Homo sapiens	SRSF3
R-HSA-69473	G2/M DNA damage checkpoint	1	81	0.006	0.247685442	2.48E-01	2	12	0.0009	9606	Homo sapiens	H4C1

R-HSA-1236394	Signaling by ERBB4	1	82	0.006	0.250331732	2.50E-01	2	52	0.0038	9606	Homo sapiens	UBA52
R-HSA-8939902	Regulation of RUNX2 expression and activity	1	83	0.006	0.252968899	2.53E-01	9	32	0.0024	9606	Homo sapiens	UBA52
R-HSA-176408	Regulation of APC/C activators between G1/S and early anaphase	1	83	0.006	0.252968899	2.53E-01	2	14	0.0010	9606	Homo sapiens	UBA52
R-HSA-1169408	ISG15 antiviral mechanism	1	83	0.006	0.252968899	2.53E-01	1	16	0.0012	9606	Homo sapiens	UBA52
R-HSA-917937	Iron uptake and transport	1	83	0.006	0.252968899	2.53E-01	1	34	0.0025	9606	Homo sapiens	UBA52
R-HSA-8852135	Protein ubiquitination	1	84	0.006	0.255596974	2.56E-01	29	32	0.0024	9606	Homo sapiens	UBA52
R-HSA-1980143	Signaling by NOTCH1	1	84	0.006	0.255596974	2.56E-01	6	39	0.0029	9606	Homo sapiens	UBA52
R-HSA-69620	Cell Cycle Checkpoints	2	280	0.020	0.257173856	2.57E-01	9	56	0.0041	9606	Homo sapiens	H4C1;UBA52
R-HSA-6781827	Transcription-Coupled Nucleotide Excision Repair (TC-NER)	1	85	0.006	0.258215988	2.58E-01	13	17	0.0013	9606	Homo sapiens	UBA52
R-HSA-1234174	Cellular response to hypoxia	1	86	0.006	0.260825971	2.61E-01	4	20	0.0015	9606	Homo sapiens	UBA52
R-HSA-170834	Signaling by TGF-beta Receptor Complex	1	87	0.006	0.263426953	2.63E-01	21	88	0.0065	9606	Homo sapiens	UBA52
R-HSA-9659379	Sensory processing of sound	1	87	0.006	0.263426953	2.63E-01	2	13	0.0010	9606	Homo sapiens	ACTG1
R-HSA-674695	RNA Polymerase II Pre-transcription Events	1	88	0.006	0.266018966	2.66E-01	3	17	0.0013	9606	Homo sapiens	UBA52
R-HSA-5654738	Signaling by FGFR2	1	88	0.006	0.266018966	2.66E-01	3	46	0.0034	9606	Homo sapiens	UBA52
R-HSA-6806834	Signaling by MET	1	88	0.006	0.266018966	2.66E-01	3	51	0.0038	9606	Homo sapiens	UBA52
R-HSA-69202	Cyclin E associated events during G1/S transition	1	88	0.006	0.266018966	2.66E-01	2	20	0.0015	9606	Homo sapiens	UBA52
R-HSA-2995410	Nuclear Envelope (NE) Reassembly	1	88	0.006	0.266018966	2.66E-01	1	24	0.0018	9606	Homo sapiens	TUBB
R-HSA-6806667	Metabolism of fat-soluble vitamins	1	89	0.006	0.26860204	2.69E-01	1	34	0.0025	9606	Homo sapiens	AKR1B10
R-HSA-5688426	Deubiquitination	2	289	0.020	0.268890049	2.69E-01	55	77	0.0057	9606	Homo sapiens	H2AC19;UBA52
R-HSA-72202	Transport of Mature Transcript to Cytoplasm	1	90	0.006	0.271176204	2.71E-01	2	13	0.0010	9606	Homo sapiens	SRSF3
R-HSA-69656	Cyclin A:Cdk2-associated events at S phase entry	1	90	0.006	0.271176204	2.71E-01	2	19	0.0014	9606	Homo sapiens	UBA52
R-HSA-195253	Degradation of beta-catenin by the destruction complex	1	91	0.006	0.273741488	2.74E-01	3	23	0.0017	9606	Homo sapiens	UBA52
R-HSA-1168372	Downstream signaling events of B Cell Receptor (BCR)	1	91	0.006	0.273741488	2.74E-01	1	15	0.0011	9606	Homo sapiens	UBA52
R-HSA-174143	APC/C-mediated degradation of cell cycle proteins	1	92	0.006	0.276297924	2.76E-01	16	40	0.0029	9606	Homo sapiens	UBA52
R-HSA-453276	Regulation of mitotic cell cycle	1	92	0.006	0.276297924	2.76E-01	16	40	0.0029	9606	Homo sapiens	UBA52
R-HSA-5696399	Global Genome Nucleotide Excision Repair (GG-NER)	1	92	0.006	0.276297924	2.76E-01	14	20	0.0015	9606	Homo sapiens	UBA52
R-HSA-5632684	Hedgehog 'on' state	1	92	0.006	0.276297924	2.76E-01	5	37	0.0027	9606	Homo sapiens	UBA52
R-HSA-9013694	Signaling by NOTCH4	1	92	0.006	0.276297924	2.76E-01	2	20	0.0015	9606	Homo sapiens	UBA52
R-HSA-69052	Switching of origins to a post-replicative state	1	93	0.007	0.27884554	2.79E-01	4	9	0.0007	9606	Homo sapiens	UBA52
R-HSA-9678108	SARS-CoV-1 Infection	1	93	0.007	0.27884554	2.79E-01	1	111	0.0082	9606	Homo sapiens	UBA52
R-HSA-975871	MyD88 cascade initiated on plasma membrane	1	94	0.007	0.281384367	2.81E-01	10	58	0.0043	9606	Homo sapiens	UBA52
R-HSA-168176	Toll Like Receptor 5 (TLR5) Cascade	1	94	0.007	0.281384367	2.81E-01	10	59	0.0043	9606	Homo sapiens	UBA52
R-HSA-168142	Toll Like Receptor 10 (TLR10) Cascade	1	94	0.007	0.281384367	2.81E-01	10	59	0.0043	9606	Homo sapiens	UBA52
R-HSA-6807505	RNA polymerase II transcribes snRNA genes	1	94	0.007	0.281384367	2.81E-01	9	11	0.0008	9606	Homo sapiens	UBA52
R-HSA-1169410	Antiviral mechanism by IFN-stimulated genes	1	94	0.007	0.281384367	2.81E-01	1	31	0.0023	9606	Homo sapiens	UBA52
R-HSA-438064	Post NMDA receptor activation events	1	96	0.007	0.28643577	2.86E-01	1	39	0.0029	9606	Homo sapiens	TUBB
R-HSA-5619084	ABC transporter disorders	1	99	0.007	0.293947694	2.94E-01	3	22	0.0016	9606	Homo sapiens	UBA52
R-HSA-4086400	PCP/CE pathway	1	99	0.007	0.293947694	2.94E-01	1	24	0.0018	9606	Homo sapiens	UBA52
R-HSA-112310	Neurotransmitter release cycle	1	100	0.007	0.296434404	2.96E-01	2	37	0.0027	9606	Homo sapiens	HSPA8
R-HSA-975138	TRAF6 mediated induction of NFkB and MAP kinases upon TLR7/	1	101	0.007	0.29891253	2.99E-01	10	48	0.0035	9606	Homo sapiens	UBA52
R-HSA-168164	Toll Like Receptor 3 (TLR3) Cascade	1	102	0.007	0.301382102	3.01E-01	13	61	0.0045	9606	Homo sapiens	UBA52
R-HSA-975155	MyD88 dependent cascade initiated on endosome	1	102	0.007	0.301382102	3.01E-01	11	63	0.0046	9606	Homo sapiens	UBA52
R-HSA-2682334	EPH-Ephrin signaling	1	102	0.007	0.301382102	3.01E-01	2	56	0.0041	9606	Homo sapiens	ACTG1
R-HSA-168181	Toll Like Receptor 7/8 (TLR7/8) Cascade	1	103	0.007	0.303843148	3.04E-01	11	66	0.0049	9606	Homo sapiens	UBA52
R-HSA-5668541	TNFR2 non-canonical NF-kB pathway	1	104	0.007	0.306295697	3.06E-01	5	43	0.0032	9606	Homo sapiens	UBA52
R-HSA-400253	Circadian Clock	1	105	0.007	0.308739779	3.09E-01	2	59	0.0043	9606	Homo sapiens	UBA52
R-HSA-168138	Toll Like Receptor 9 (TLR9) Cascade	1	106	0.007	0.31117542	3.11E-01	11	68	0.0050	9606	Homo sapiens	UBA52
R-HSA-5687128	MAPK6/MAPK4 signaling	1	106	0.007	0.31117542	3.11E-01	2	40	0.0029	9606	Homo sapiens	UBA52
R-HSA-937061	TRIF(TICAM1)-mediated TLR4 signaling	1	107	0.007	0.313602651	3.14E-01	13	58	0.0043	9606	Homo sapiens	UBA52
R-HSA-166166	MyD88-independent TLR4 cascade	1	107	0.007	0.313602651	3.14E-01	13	60	0.0044	9606	Homo sapiens	UBA52
R-HSA-190236	Signaling by FGFR	1	107	0.007	0.313602651	3.14E-01	6	142	0.0105	9606	Homo sapiens	UBA52
R-HSA-6811434	COPI-dependent Golgi-to-ER retrograde traffic	1	107	0.007	0.313602651	3.14E-01	2	11	0.0008	9606	Homo sapiens	TUBB
R-HSA-6807878	COPI-mediated anterograde transport	1	107	0.007	0.313602651	3.14E-01	2	12	0.0009	9606	Homo sapiens	TUBB
R-HSA-9020702	Interleukin-1 signaling	1	109	0.008	0.318431994	3.18E-01	9	47	0.0035	9606	Homo sapiens	UBA52
R-HSA-8957275	Post-translational protein phosphorylation	1	109	0.008	0.318431994	3.18E-01	1	1	0.0001	9606	Homo sapiens	HSP90B1
R-HSA-193704	p75 NTR receptor-mediated signalling	1	110	0.008	0.320834163	3.21E-01	5	50	0.0037	9606	Homo sapiens	UBA52
R-HSA-74160	Gene expression (Transcription)	8	1855	0.130	0.321591278	3.22E-01	113	1006	0.0741	9606	Homo sapiens	HSP90AA1;PRDX1;H2AC19;SRSF3;H4C1;UBA52;LRPPRC
R-HSA-9009391	Extra-nuclear estrogen signaling	1	111	0.008	0.323228034	3.23E-01	1	38	0.0028	9606	Homo sapiens	HSP90AA1
R-HSA-9694516	SARS-CoV-2 Infection	1	112	0.008	0.325613635	3.26E-01	1	116	0.0085	9606	Homo sapiens	UBA52
R-HSA-3371453	Regulation of HSF1-mediated heat shock response	1	113	0.008	0.327990995	3.28E-01	7	14	0.0010	9606	Homo sapiens	HSPA8
R-HSA-442755	Activation of NMDA receptors and postsynaptic events	1	113	0.008	0.327990995	3.28E-01	3	71	0.0052	9606	Homo sapiens	TUBB
R-HSA-8856825	Cargo recognition for clathrin-mediated endocytosis	1	115	0.008	0.332721101	3.33E-01	1	15	0.0011	9606	Homo sapiens	UBA52
R-HSA-9006936	Signaling by TGFB family members	1	116	0.008	0.335073904	3.35E-01	21	113	0.0083	9606	Homo sapiens	UBA52
R-HSA-112315	Transmission across Chemical Synapses	2	343	0.024	0.338877553	3.39E-01	5	163	0.0120	9606	Homo sapiens	HSPA8;TUBB
R-HSA-166058	MyD88:MAL(TIRAP) cascade initiated on plasma membrane	1	118	0.008	0.339755144	3.40E-01	10	64	0.0047	9606	Homo sapiens	UBA52
R-HSA-168188	Toll Like Receptor TLR6:TLR2 Cascade	1	118	0.008	0.339755144	3.40E-01	10	66	0.0049	9606	Homo sapiens	UBA52
R-HSA-8878159	Transcriptional regulation by RUNX3	1	118	0.008	0.339755144	3.40E-01	3	47	0.0035	9606	Homo sapiens	UBA52
R-HSA-611105	Respiratory electron transport	1	118	0.008	0.339755144	3.40E-01	1	19	0.0014	9606	Homo sapiens	LRPPRC
R-HSA-5696398	Nucleotide Excision Repair	1	119	0.008	0.342083637	3.42E-01	27	37	0.0027	9606	Homo sapiens	UBA52
R-HSA-2672351	Stimuli-sensing channels	1	119	0.008	0.342083637	3.42E-01	1	28	0.0021	9606	Homo sapiens	UBA52
R-HSA-5607764	CLEC7A (Dectin-1) signaling	1	120	0.008	0.344404081	3.44E-01	6	45	0.0033	9606	Homo sapiens	UBA52
R-HSA-168179	Toll Like Receptor TLR1:TLR2 Cascade	1	121	0.008	0.346716505	3.47E-01	10	66	0.0049	9606	Homo sapiens	UBA52
R-HSA-181438	Toll Like Receptor 2 (TLR2) Cascade	1	121	0.008	0.346716505	3.47E-01	10	68	0.0050	9606	Homo sapiens	UBA52
R-HSA-9648025	EML4 and NUDC in mitotic spindle formation	1	121	0.008	0.346716505	3.47E-01	2	5	0.0004	9606	Homo sapiens	TUBB
R-HSA-382556	ABC-family proteins mediated transport	1	122	0.009	0.349020935	3.49E-01	3	27	0.0020	9606	Homo sapiens	UBA52
R-HSA-202424	Downstream TCR signaling	1	124	0.009	0.353605921	3.54E-01	3	24	0.0018	9606	Homo sapiens	UBA52
R-HSA-381426	Regulation of Insulin-like Growth Factor (IGF) transport and upta	1	127	0.009	0.360424121	3.60E-01	1	14	0.0010	9606	Homo sapiens	HSP90B1

R-HSA-69239	Synthesis of DNA	1	133	0.009	0.373849749	3.74E-01	4	26	0.0019	9606	Homo sapiens	UBA52
R-HSA-2500257	Resolution of Sister Chromatid Cohesion	1	134	0.009	0.376060339	3.76E-01	4	8	0.0006	9606	Homo sapiens	TUBB
R-HSA-9635486	Infection with Mycobacterium tuberculosis	1	135	0.009	0.37826328	3.78E-01	2	73	0.0054	9606	Homo sapiens	UBA52
R-HSA-73857	RNA Polymerase II Transcription	7	1694	0.119	0.382380365	3.82E-01	89	891	0.0656	9606	Homo sapiens	PRDX1;H2AC19;SRSF3;H4C1;UBA52;LRPPRC
R-HSA-196854	Metabolism of vitamins and cofactors	2	379	0.027	0.384563504	3.85E-01	3	206	0.0152	9606	Homo sapiens	HSP90AA1;AKR1B10
R-HSA-1430728	Metabolism	14	3646	0.255	0.397390069	3.97E-01	27	2260	0.1665	9606	Homo sapiens	HSP90AA1;AKR1B10;PKM;HSP90AB1;TALDO1;RPSA;TKT;UBA52;GAPDH;LRPPRC;RPL6
R-HSA-8878166	Transcriptional regulation by RUNX2	1	147	0.010	0.404111317	4.04E-01	9	84	0.0062	9606	Homo sapiens	UBA52
R-HSA-202403	TCR signaling	1	147	0.010	0.404111317	4.04E-01	3	52	0.0038	9606	Homo sapiens	UBA52
R-HSA-8856688	Golgi-to-ER retrograde transport	1	148	0.010	0.406217152	4.06E-01	4	18	0.0013	9606	Homo sapiens	TUBB
R-HSA-2132295	MHC class II antigen presentation	1	149	0.010	0.408315693	4.08E-01	1	26	0.0019	9606	Homo sapiens	TUBB
R-HSA-69206	G1/S Transition	1	150	0.011	0.410406965	4.10E-01	2	61	0.0045	9606	Homo sapiens	UBA52
R-HSA-166016	Toll Like Receptor 4 (TLR4) Cascade	1	151	0.011	0.412490993	4.12E-01	18	95	0.0070	9606	Homo sapiens	UBA52
R-HSA-162909	Host Interactions of HIV factors	1	151	0.011	0.412490993	4.12E-01	4	54	0.0040	9606	Homo sapiens	UBA52
R-HSA-71291	Metabolism of amino acids and derivatives	3	665	0.047	0.413932859	4.14E-01	2	285	0.0210	9606	Homo sapiens	RPSA;UBA52;RPL6
R-HSA-163200	Respiratory electron transport, ATP synthesis by chemiosmotic cc	1	153	0.011	0.416637416	4.17E-01	1	31	0.0023	9606	Homo sapiens	LRPPRC
R-HSA-9658195	Leishmania infection	2	406	0.028	0.417983979	4.18E-01	11	95	0.0070	9606	Homo sapiens	HSP90AB1;ACTG1
R-HSA-8980692	RHOA GTPase cycle	1	154	0.011	0.418699858	4.19E-01	1	6	0.0004	9606	Homo sapiens	JUP
R-HSA-5668914	Diseases of metabolism	2	409	0.029	0.421644916	4.22E-01	3	190	0.0140	9606	Homo sapiens	TALDO1;UBA52
R-HSA-9664417	Leishmania phagocytosis	1	157	0.011	0.424844406	4.25E-01	6	27	0.0020	9606	Homo sapiens	ACTG1
R-HSA-9664422	FCGR3A-mediated phagocytosis	1	157	0.011	0.424844406	4.25E-01	6	27	0.0020	9606	Homo sapiens	ACTG1
R-HSA-9664407	Parasite infection	1	157	0.011	0.424844406	4.25E-01	6	27	0.0020	9606	Homo sapiens	ACTG1
R-HSA-73887	Death Receptor Signalling	1	159	0.011	0.428905362	4.29E-01	8	93	0.0068	9606	Homo sapiens	UBA52
R-HSA-9013148	CDC42 GTPase cycle	1	159	0.011	0.428905362	4.29E-01	1	6	0.0004	9606	Homo sapiens	JUP
R-HSA-446203	Asparagine N-linked glycosylation	2	421	0.030	0.436175405	4.36E-01	6	144	0.0106	9606	Homo sapiens	TUBB;UBA52
R-HSA-199977	ER to Golgi Anterograde Transport	1	164	0.011	0.438935216	4.39E-01	2	39	0.0029	9606	Homo sapiens	TUBB
R-HSA-3858494	Beta-catenin independent WNT signaling	1	166	0.012	0.442898641	4.43E-01	1	51	0.0038	9606	Homo sapiens	UBA52
R-HSA-446652	Interleukin-1 family signaling	1	167	0.012	0.444870051	4.45E-01	9	80	0.0059	9606	Homo sapiens	UBA52
R-HSA-2187338	Visual phototransduction	1	169	0.012	0.448792383	4.49E-01	1	92	0.0068	9606	Homo sapiens	AKR1B10
R-HSA-6807070	PTEN Regulation	1	171	0.012	0.452687552	4.53E-01	8	56	0.0041	9606	Homo sapiens	UBA52
R-HSA-453279	Mitotic G1 phase and G1/S transition	1	173	0.012	0.456555742	4.57E-01	3	99	0.0073	9606	Homo sapiens	UBA52
R-HSA-1236974	ER-Phagosome pathway	1	173	0.012	0.456555742	4.57E-01	1	10	0.0007	9606	Homo sapiens	UBA52
R-HSA-2871837	FCERI mediated NF-kB activation	1	175	0.012	0.460397136	4.60E-01	3	19	0.0014	9606	Homo sapiens	UBA52
R-HSA-212436	Generic Transcription Pathway	6	1555	0.109	0.466565534	4.67E-01	62	830	0.0611	9606	Homo sapiens	PRDX1;H2AC19;H4C1;UBA52;LRPPRC
R-HSA-69242	S Phase	1	180	0.013	0.469884579	4.70E-01	8	54	0.0040	9606	Homo sapiens	UBA52
R-HSA-3108232	SUMO E3 ligases SUMOylate target proteins	1	183	0.013	0.475498371	4.75E-01	2	131	0.0096	9606	Homo sapiens	H4C1
R-HSA-983705	Signaling by the B Cell Receptor (BCR)	1	189	0.013	0.486551733	4.87E-01	1	44	0.0032	9606	Homo sapiens	UBA52
R-HSA-2990846	SUMOylation	1	192	0.013	0.491992482	4.92E-01	2	140	0.0103	9606	Homo sapiens	H4C1
R-HSA-109581	Apoptosis	1	192	0.013	0.491992482	4.92E-01	2	141	0.0104	9606	Homo sapiens	UBA52
R-HSA-597592	Post-translational protein modification	6	1598	0.112	0.493951316	4.94E-01	102	526	0.0387	9606	Homo sapiens	HSPA8;TUBB;H2AC19;H4C1;UBA52;HSP90B1
R-HSA-983231	Factors involved in megakaryocyte development and platelet pro	1	194	0.014	0.495588216	4.96E-01	2	43	0.0032	9606	Homo sapiens	TUBB
R-HSA-1236975	Antigen processing-Cross presentation	1	195	0.014	0.497376719	4.97E-01	1	23	0.0017	9606	Homo sapiens	UBA52
R-HSA-5621481	C-type lectin receptors (CLRs)	1	203	0.014	0.511462759	5.11E-01	6	68	0.0050	9606	Homo sapiens	UBA52
R-HSA-112316	Neuronal System	2	489	0.034	0.514703964	5.15E-01	5	216	0.0159	9606	Homo sapiens	HSPA8;TUBB
R-HSA-983712	Ion channel transport	1	206	0.014	0.516644663	5.17E-01	1	45	0.0033	9606	Homo sapiens	UBA52
R-HSA-9709957	Sensory Perception	2	493	0.035	0.51910494	5.19E-01	3	139	0.0102	9606	Homo sapiens	AKR1B10;ACTG1
R-HSA-6811442	Intra-Golgi and retrograde Golgi-to-ER traffic	1	219	0.015	0.538484014	5.38E-01	4	48	0.0035	9606	Homo sapiens	TUBB
R-HSA-948021	Transport to the Golgi and subsequent modification	1	219	0.015	0.538484014	5.38E-01	2	60	0.0044	9606	Homo sapiens	TUBB
R-HSA-112314	Neurotransmitter receptors and postsynaptic signal transmission	1	232	0.016	0.559355458	5.59E-01	3	109	0.0080	9606	Homo sapiens	TUBB
R-HSA-2454202	Fc epsilon receptor (FCERI) signaling	1	235	0.016	0.56403897	5.64E-01	3	65	0.0048	9606	Homo sapiens	UBA52
R-HSA-1428517	The citric acid (TCA) cycle and respiratory electron transport	1	238	0.017	0.568673687	5.69E-01	1	67	0.0049	9606	Homo sapiens	LRPPRC
R-HSA-8951664	Neddylation	1	241	0.017	0.573260107	5.73E-01	2	31	0.0023	9606	Homo sapiens	UBA52
R-HSA-5619115	Disorders of transmembrane transporters	1	278	0.019	0.62604323	6.26E-01	3	87	0.0064	9606	Homo sapiens	UBA52
R-HSA-211945	Phase I - Functionalization of compounds	1	299	0.021	0.653096612	6.53E-01	3	98	0.0072	9606	Homo sapiens	HSP90AB1
R-HSA-983168	Antigen processing: Ubiquitination & Proteasome degradation	1	315	0.022	0.672412191	6.72E-01	6	9	0.0007	9606	Homo sapiens	UBA52
R-HSA-1257604	PIP3 activates AKT signaling	1	319	0.022	0.677073956	6.77E-01	8	86	0.0063	9606	Homo sapiens	UBA52
R-HSA-8957322	Metabolism of steroids	1	328	0.023	0.68732651	6.87E-01	1	244	0.0180	9606	Homo sapiens	AKR1B10
R-HSA-1474244	Extracellular matrix organization	1	329	0.023	0.688445798	6.88E-01	1	319	0.0235	9606	Homo sapiens	TKT
R-HSA-9006925	Intracellular signaling by second messengers	1	366	0.026	0.72720943	7.27E-01	8	114	0.0084	9606	Homo sapiens	UBA52
R-HSA-162582	Signal Transduction	9	2997	0.210	0.750516292	7.51E-01	200	2454	0.1807	9606	Homo sapiens	HSP90AA1;HSP90AB1;JUP;TUBB;NF1;H2AC19;H4C1;UBA52;ACTG1
R-HSA-913531	Interferon Signaling	1	394	0.028	0.753362501	7.53E-01	1	69	0.0051	9606	Homo sapiens	UBA52
R-HSA-983169	Class I MHC mediated antigen processing & presentation	1	474	0.033	0.815280431	8.15E-01	7	48	0.0035	9606	Homo sapiens	UBA52
R-HSA-211859	Biological oxidations	1	549	0.038	0.859346533	8.59E-01	3	188	0.0138	9606	Homo sapiens	HSP90AB1
R-HSA-1280218	Adaptive Immune System	2	1005	0.070	0.875713921	8.76E-01	12	264	0.0194	9606	Homo sapiens	TUBB;UBA52
R-HSA-109582	Hemostasis	1	803	0.056	0.944736921	9.45E-01	2	334	0.0246	9606	Homo sapiens	TUBB
R-HSA-382551	Transport of small molecules	1	961	0.067	0.969367498	9.69E-01	5	443	0.0326	9606	Homo sapiens	UBA52
R-HSA-556833	Metabolism of lipids	1	1444	0.101	0.995174588	9.95E-01	1	954	0.0703	9606	Homo sapiens	AKR1B10
